# Supplementary material for: Statistical Modeling to Adjust for Time Trends in Adaptive Platform Trials Utilizing Non‐Concurrent Controls
Source: Biom J. 2025 Jun 10;67(3):e70059. doi: 10.1002/bimj.70059 (PMC12150008; doi:10.1002/bimj.70059)
Supplement: Supplementary file 1 — Supporting Information [file BIMJ-67-e70059-s002.zip › case_studies/FLAIR/NCC_FreqModels_case_study_FLAIR.html]

Statistical modeling to adjust for time trends in adaptive platform trials utilizing non-concurrent controls


Code 

- Show All Code
- Hide All Code

# Statistical modeling to adjust for time trends in adaptive platform trials utilizing non-concurrent controls

### Case study: FLAIR trial

#### Pavla Krotka, Martin Posch, Mohamed Gewily, Günter Höglinger, Marta Bofill Roig

#### 2024

# 1 Introduction

This file contains all code to reproduce the case study based on the
FLAIR trial presented in Supplementary material (Section D) of the paper
*“Statistical modeling to adjust for time trends in adaptive platform
trials utilizing non-concurrent controls”* by Pavla Krotka, Martin
Posch, Mohamed Gewily, Günter Höglinger, Marta Bofill Roig. The results
obtained from the analyses below are presented in Table S1 and Figure
S12.

# 2 Data simulation

```
# Function to simulate data from the FLAIR trial

sim_flair <- function(lambda, trend_pattern, theta = c(-log(0.44), -log(0.13))){
  
  # treatments: FCR-0, IR-1, IV-2
  
  period1 <- sample(c(rep(0, 316), rep(1, 317)))
  period2 <- sample(c(rep(0, 69), rep(1, 69), rep(2, 69)))
  period3 <- sample(c(rep(0, 194), rep(2, 191)))
  
  trt <- c(period1, period2, period3)
  
  per <- c(rep(1, length(period1)), rep(2, length(period2)), rep(3, length(period3)))
  
  per_trend <- c(rep(1, length(period1)), rep(2, length(period2)+length(period3)))
  
  j0 <- which(trt==0)
  j1 <- which(trt==1)
  j2 <- which(trt==2)
  
  if (trend_pattern=="linear") {
    trend <- linear_trend(1:length(trt), lambda = lambda, sample_size = c(1, length(trt)))
  }
  
  if (trend_pattern=="stepwise") {
    trend <- sw_trend(cj = per_trend, lambda = lambda)
  }
  
  
  means <- c()
  
  means[j0] <- 0
  means[j1] <- theta[1]
  means[j2] <- theta[2]
  
  response <- rnorm(length(trt), means, 1)
  
  # Add time trend
  
  response[j0] <- response[j0] + trend[j0]
  response[j1] <- response[j1] + trend[j1]
  response[j2] <- response[j2] + trend[j2]
  
  unit_size <- 100
  
  flair_data <- data.frame(j = 1:length(trt),
                           treatment = trt,
                           period = per,
                           response = response,
                           cal_time = rep(c(1:ceiling((length(trt)/unit_size))), each = unit_size)[1:length(trt)])
  
  flair_data
}
```

```
# Simulation of 6 settings with different time trends patterns and strengths (using same simulation runs to distinguish changes due to the added time trend)

set.seed(132)
flair_data_lin_0.25 <- sim_flair(lambda = 0.25, trend_pattern = "linear")

set.seed(132)
flair_data_lin_1.5 <- sim_flair(lambda = 1.5, trend_pattern = "linear")

set.seed(132)
flair_data_lin_5 <- sim_flair(lambda = 5, trend_pattern = "linear")

set.seed(132)
flair_data_step_0.25 <- sim_flair(lambda = 0.25, trend_pattern = "stepwise")

set.seed(132)
flair_data_step_1.5 <- sim_flair(lambda = 1.5, trend_pattern = "stepwise")

set.seed(132)
flair_data_step_5 <- sim_flair(lambda = 5, trend_pattern = "stepwise")
```

## 2.1 Sample sizes per treatment and period

```
table(flair_data_lin_0.25$treatment, flair_data_lin_0.25$period)
#>    
#>       1   2   3
#>   0 316  69 194
#>   1 317  69   0
#>   2   0  69 191
```

```
rowSums(table(flair_data_lin_0.25$treatment, flair_data_lin_0.25$period))
#>   0   1   2 
#> 579 386 260
```

## 2.2 Total sample size

```
nrow(flair_data_lin_0.25)
#> [1] 1225
```

# 3 Considered approaches

## 3.1 Regression model - period adjustment

### 3.1.1 Linear trend, \(\lambda=0.25\)

```
mod_lm_per <- lm(response ~ as.factor(treatment) + as.factor(period), flair_data_lin_0.25)
summary(mod_lm_per)
#> 
#> Call:
#> lm(formula = response ~ as.factor(treatment) + as.factor(period), 
#>     data = flair_data_lin_0.25)
#> 
#> Residuals:
#>      Min       1Q   Median       3Q      Max 
#> -3.03632 -0.67654  0.00051  0.64590  3.14989 
#> 
#> Coefficients:
#>                       Estimate Std. Error t value Pr(>|t|)    
#> (Intercept)            0.05004    0.05366   0.933  0.35119    
#> as.factor(treatment)1  0.79810    0.07122  11.206  < 2e-16 ***
#> as.factor(treatment)2  2.03655    0.08535  23.863  < 2e-16 ***
#> as.factor(period)2     0.02221    0.08591   0.258  0.79607    
#> as.factor(period)3     0.23639    0.08310   2.844  0.00452 ** 
#> ---
#> Signif. codes:  0 '***' 0.001 '**' 0.01 '*' 0.05 '.' 0.1 ' ' 1
#> 
#> Residual standard error: 1.009 on 1220 degrees of freedom
#> Multiple R-squared:  0.4001, Adjusted R-squared:  0.3981 
#> F-statistic: 203.4 on 4 and 1220 DF,  p-value: < 2.2e-16
```

### 3.1.2 Linear trend, \(\lambda=1.5\)

```
mod_lm_per <- lm(response ~ as.factor(treatment) + as.factor(period), flair_data_lin_1.5)
summary(mod_lm_per)
#> 
#> Call:
#> lm(formula = response ~ as.factor(treatment) + as.factor(period), 
#>     data = flair_data_lin_1.5)
#> 
#> Residuals:
#>      Min       1Q   Median       3Q      Max 
#> -2.96798 -0.72226 -0.00281  0.67547  3.05447 
#> 
#> Coefficients:
#>                       Estimate Std. Error t value Pr(>|t|)    
#> (Intercept)            0.36945    0.05453   6.776 1.92e-11 ***
#> as.factor(treatment)1  0.80417    0.07237  11.111  < 2e-16 ***
#> as.factor(treatment)2  2.03388    0.08673  23.452  < 2e-16 ***
#> as.factor(period)2     0.45269    0.08730   5.185 2.52e-07 ***
#> as.factor(period)3     0.97137    0.08445  11.502  < 2e-16 ***
#> ---
#> Signif. codes:  0 '***' 0.001 '**' 0.01 '*' 0.05 '.' 0.1 ' ' 1
#> 
#> Residual standard error: 1.025 on 1220 degrees of freedom
#> Multiple R-squared:  0.4975, Adjusted R-squared:  0.4958 
#> F-statistic: 301.9 on 4 and 1220 DF,  p-value: < 2.2e-16
```

### 3.1.3 Linear trend, \(\lambda=5\)

```
mod_lm_per <- lm(response ~ as.factor(treatment) + as.factor(period), flair_data_lin_5)
summary(mod_lm_per)
#> 
#> Call:
#> lm(formula = response ~ as.factor(treatment) + as.factor(period), 
#>     data = flair_data_lin_5)
#> 
#> Residuals:
#>     Min      1Q  Median      3Q     Max 
#> -3.4509 -0.8284 -0.0197  0.8270  3.9260 
#> 
#> Coefficients:
#>                       Estimate Std. Error t value Pr(>|t|)    
#> (Intercept)            1.26379    0.06261  20.184   <2e-16 ***
#> as.factor(treatment)1  0.82118    0.08311   9.881   <2e-16 ***
#> as.factor(treatment)2  2.02638    0.09959  20.348   <2e-16 ***
#> as.factor(period)2     1.65803    0.10025  16.539   <2e-16 ***
#> as.factor(period)3     3.02932    0.09697  31.238   <2e-16 ***
#> ---
#> Signif. codes:  0 '***' 0.001 '**' 0.01 '*' 0.05 '.' 0.1 ' ' 1
#> 
#> Residual standard error: 1.177 on 1220 degrees of freedom
#> Multiple R-squared:  0.698,  Adjusted R-squared:  0.697 
#> F-statistic: 704.9 on 4 and 1220 DF,  p-value: < 2.2e-16
```

### 3.1.4 Stepwise trend, \(\lambda=0.25\)

```
mod_lm_per <- lm(response ~ as.factor(treatment) + as.factor(period), flair_data_step_0.25)
summary(mod_lm_per)
#> 
#> Call:
#> lm(formula = response ~ as.factor(treatment) + as.factor(period), 
#>     data = flair_data_step_0.25)
#> 
#> Residuals:
#>     Min      1Q  Median      3Q     Max 
#> -3.0500 -0.6714  0.0054  0.6517  3.2134 
#> 
#> Coefficients:
#>                       Estimate Std. Error t value Pr(>|t|)    
#> (Intercept)           -0.01384    0.05363  -0.258   0.7964    
#> as.factor(treatment)1  0.79688    0.07118  11.195  < 2e-16 ***
#> as.factor(treatment)2  2.03709    0.08530  23.883  < 2e-16 ***
#> as.factor(period)2     0.18611    0.08586   2.168   0.0304 *  
#> as.factor(period)3     0.33939    0.08306   4.086 4.67e-05 ***
#> ---
#> Signif. codes:  0 '***' 0.001 '**' 0.01 '*' 0.05 '.' 0.1 ' ' 1
#> 
#> Residual standard error: 1.008 on 1220 degrees of freedom
#> Multiple R-squared:  0.4167, Adjusted R-squared:  0.4148 
#> F-statistic: 217.9 on 4 and 1220 DF,  p-value: < 2.2e-16
```

### 3.1.5 Stepwise trend, \(\lambda=1.5\)

```
mod_lm_per <- lm(response ~ as.factor(treatment) + as.factor(period), flair_data_step_1.5)
summary(mod_lm_per)
#> 
#> Call:
#> lm(formula = response ~ as.factor(treatment) + as.factor(period), 
#>     data = flair_data_step_1.5)
#> 
#> Residuals:
#>     Min      1Q  Median      3Q     Max 
#> -3.0500 -0.6714  0.0054  0.6517  3.2134 
#> 
#> Coefficients:
#>                       Estimate Std. Error t value Pr(>|t|)    
#> (Intercept)           -0.01384    0.05363  -0.258    0.796    
#> as.factor(treatment)1  0.79688    0.07118  11.195   <2e-16 ***
#> as.factor(treatment)2  2.03709    0.08530  23.883   <2e-16 ***
#> as.factor(period)2     1.43611    0.08586  16.726   <2e-16 ***
#> as.factor(period)3     1.58939    0.08306  19.136   <2e-16 ***
#> ---
#> Signif. codes:  0 '***' 0.001 '**' 0.01 '*' 0.05 '.' 0.1 ' ' 1
#> 
#> Residual standard error: 1.008 on 1220 degrees of freedom
#> Multiple R-squared:  0.6211, Adjusted R-squared:  0.6199 
#> F-statistic:   500 on 4 and 1220 DF,  p-value: < 2.2e-16
```

### 3.1.6 Stepwise trend, \(\lambda=5\)

```
mod_lm_per <- lm(response ~ as.factor(treatment) + as.factor(period), flair_data_step_5)
summary(mod_lm_per)
#> 
#> Call:
#> lm(formula = response ~ as.factor(treatment) + as.factor(period), 
#>     data = flair_data_step_5)
#> 
#> Residuals:
#>     Min      1Q  Median      3Q     Max 
#> -3.0500 -0.6714  0.0054  0.6517  3.2134 
#> 
#> Coefficients:
#>                       Estimate Std. Error t value Pr(>|t|)    
#> (Intercept)           -0.01384    0.05363  -0.258    0.796    
#> as.factor(treatment)1  0.79688    0.07118  11.195   <2e-16 ***
#> as.factor(treatment)2  2.03709    0.08530  23.883   <2e-16 ***
#> as.factor(period)2     4.93611    0.08586  57.488   <2e-16 ***
#> as.factor(period)3     5.08939    0.08306  61.277   <2e-16 ***
#> ---
#> Signif. codes:  0 '***' 0.001 '**' 0.01 '*' 0.05 '.' 0.1 ' ' 1
#> 
#> Residual standard error: 1.008 on 1220 degrees of freedom
#> Multiple R-squared:  0.8928, Adjusted R-squared:  0.8925 
#> F-statistic:  2541 on 4 and 1220 DF,  p-value: < 2.2e-16
```

## 3.2 Regression model - calendar time adjustment

### 3.2.1 Linear trend, \(\lambda=0.25\)

```
mod_lm_cal <- lm(response ~ as.factor(treatment) + as.factor(cal_time), flair_data_lin_0.25)
summary(mod_lm_cal)
#> 
#> Call:
#> lm(formula = response ~ as.factor(treatment) + as.factor(cal_time), 
#>     data = flair_data_lin_0.25)
#> 
#> Residuals:
#>     Min      1Q  Median      3Q     Max 
#> -2.9832 -0.6712 -0.0052  0.6585  3.1927 
#> 
#> Coefficients:
#>                        Estimate Std. Error t value Pr(>|t|)    
#> (Intercept)            0.006638   0.106247   0.062  0.95019    
#> as.factor(treatment)1  0.798660   0.071305  11.201  < 2e-16 ***
#> as.factor(treatment)2  2.043939   0.085040  24.035  < 2e-16 ***
#> as.factor(cal_time)2  -0.039510   0.142630  -0.277  0.78182    
#> as.factor(cal_time)3   0.141880   0.142585   0.995  0.31991    
#> as.factor(cal_time)4  -0.009748   0.142592  -0.068  0.94551    
#> as.factor(cal_time)5   0.098384   0.142801   0.689  0.49098    
#> as.factor(cal_time)6   0.097643   0.142592   0.685  0.49362    
#> as.factor(cal_time)7  -0.062757   0.143488  -0.437  0.66192    
#> as.factor(cal_time)8   0.186775   0.145391   1.285  0.19916    
#> as.factor(cal_time)9   0.061915   0.150376   0.412  0.68060    
#> as.factor(cal_time)10  0.169532   0.150773   1.124  0.26106    
#> as.factor(cal_time)11  0.439354   0.150979   2.910  0.00368 ** 
#> as.factor(cal_time)12  0.293503   0.151189   1.941  0.05245 .  
#> as.factor(cal_time)13  0.357780   0.229747   1.557  0.11967    
#> ---
#> Signif. codes:  0 '***' 0.001 '**' 0.01 '*' 0.05 '.' 0.1 ' ' 1
#> 
#> Residual standard error: 1.008 on 1210 degrees of freedom
#> Multiple R-squared:  0.4054, Adjusted R-squared:  0.3985 
#> F-statistic: 58.92 on 14 and 1210 DF,  p-value: < 2.2e-16
```

### 3.2.2 Linear trend, \(\lambda=1.5\)

```
mod_lm_cal <- lm(response ~ as.factor(treatment) + as.factor(cal_time), flair_data_lin_1.5)
summary(mod_lm_cal)
#> 
#> Call:
#> lm(formula = response ~ as.factor(treatment) + as.factor(cal_time), 
#>     data = flair_data_lin_1.5)
#> 
#> Residuals:
#>     Min      1Q  Median      3Q     Max 
#> -2.9513 -0.6756 -0.0152  0.6655  3.2101 
#> 
#> Coefficients:
#>                       Estimate Std. Error t value Pr(>|t|)    
#> (Intercept)            0.05645    0.10628   0.531 0.595402    
#> as.factor(treatment)1  0.80014    0.07132  11.218  < 2e-16 ***
#> as.factor(treatment)2  2.04697    0.08506  24.064  < 2e-16 ***
#> as.factor(cal_time)2   0.06246    0.14267   0.438 0.661631    
#> as.factor(cal_time)3   0.34596    0.14263   2.426 0.015426 *  
#> as.factor(cal_time)4   0.29634    0.14263   2.078 0.037949 *  
#> as.factor(cal_time)5   0.50638    0.14284   3.545 0.000407 ***
#> as.factor(cal_time)6   0.60788    0.14263   4.262 2.18e-05 ***
#> as.factor(cal_time)7   0.54893    0.14353   3.825 0.000138 ***
#> as.factor(cal_time)8   0.90027    0.14543   6.190 8.21e-10 ***
#> as.factor(cal_time)9   0.87726    0.15042   5.832 7.01e-09 ***
#> as.factor(cal_time)10  1.08714    0.15082   7.208 9.94e-13 ***
#> as.factor(cal_time)11  1.45897    0.15102   9.661  < 2e-16 ***
#> as.factor(cal_time)12  1.41513    0.15123   9.357  < 2e-16 ***
#> as.factor(cal_time)13  1.54349    0.22981   6.716 2.86e-11 ***
#> ---
#> Signif. codes:  0 '***' 0.001 '**' 0.01 '*' 0.05 '.' 0.1 ' ' 1
#> 
#> Residual standard error: 1.009 on 1210 degrees of freedom
#> Multiple R-squared:  0.5174, Adjusted R-squared:  0.5118 
#> F-statistic: 92.66 on 14 and 1210 DF,  p-value: < 2.2e-16
```

### 3.2.3 Linear trend, \(\lambda=5\)

```
mod_lm_cal <- lm(response ~ as.factor(treatment) + as.factor(cal_time), flair_data_lin_5)
summary(mod_lm_cal)
#> 
#> Call:
#> lm(formula = response ~ as.factor(treatment) + as.factor(cal_time), 
#>     data = flair_data_lin_5)
#> 
#> Residuals:
#>     Min      1Q  Median      3Q     Max 
#> -2.8621 -0.6906  0.0131  0.6751  3.2723 
#> 
#> Coefficients:
#>                       Estimate Std. Error t value Pr(>|t|)    
#> (Intercept)            0.19592    0.10684   1.834   0.0669 .  
#> as.factor(treatment)1  0.80430    0.07170  11.218  < 2e-16 ***
#> as.factor(treatment)2  2.05546    0.08551  24.037  < 2e-16 ***
#> as.factor(cal_time)2   0.34796    0.14342   2.426   0.0154 *  
#> as.factor(cal_time)3   0.91739    0.14338   6.398 2.24e-10 ***
#> as.factor(cal_time)4   1.15340    0.14338   8.044 2.05e-15 ***
#> as.factor(cal_time)5   1.64878    0.14359  11.482  < 2e-16 ***
#> as.factor(cal_time)6   2.03653    0.14338  14.203  < 2e-16 ***
#> as.factor(cal_time)7   2.26164    0.14428  15.675  < 2e-16 ***
#> as.factor(cal_time)8   2.89805    0.14620  19.823  < 2e-16 ***
#> as.factor(cal_time)9   3.16022    0.15121  20.900  < 2e-16 ***
#> as.factor(cal_time)10  3.65645    0.15161  24.118  < 2e-16 ***
#> as.factor(cal_time)11  4.31391    0.15182  28.415  < 2e-16 ***
#> as.factor(cal_time)12  4.55570    0.15203  29.966  < 2e-16 ***
#> as.factor(cal_time)13  4.86348    0.23102  21.052  < 2e-16 ***
#> ---
#> Signif. codes:  0 '***' 0.001 '**' 0.01 '*' 0.05 '.' 0.1 ' ' 1
#> 
#> Residual standard error: 1.014 on 1210 degrees of freedom
#> Multiple R-squared:  0.7777, Adjusted R-squared:  0.7752 
#> F-statistic: 302.4 on 14 and 1210 DF,  p-value: < 2.2e-16
```

### 3.2.4 Stepwise trend, \(\lambda=0.25\)

```
mod_lm_cal <- lm(response ~ as.factor(treatment) + as.factor(cal_time), flair_data_step_0.25)
summary(mod_lm_cal)
#> 
#> Call:
#> lm(formula = response ~ as.factor(treatment) + as.factor(cal_time), 
#>     data = flair_data_step_0.25)
#> 
#> Residuals:
#>     Min      1Q  Median      3Q     Max 
#> -2.9912 -0.6715  0.0007  0.6656  3.2560 
#> 
#> Coefficients:
#>                        Estimate Std. Error t value Pr(>|t|)    
#> (Intercept)           -0.001701   0.106295  -0.016  0.98723    
#> as.factor(treatment)1  0.794910   0.071337  11.143  < 2e-16 ***
#> as.factor(treatment)2  2.053678   0.085078  24.139  < 2e-16 ***
#> as.factor(cal_time)2  -0.059731   0.142694  -0.419  0.67559    
#> as.factor(cal_time)3   0.101063   0.142650   0.708  0.47879    
#> as.factor(cal_time)4  -0.070897   0.142657  -0.497  0.61929    
#> as.factor(cal_time)5   0.017164   0.142865   0.120  0.90439    
#> as.factor(cal_time)6  -0.004473   0.142657  -0.031  0.97499    
#> as.factor(cal_time)7  -0.019594   0.143553  -0.136  0.89145    
#> as.factor(cal_time)8   0.290180   0.145456   1.995  0.04627 *  
#> as.factor(cal_time)9   0.142259   0.150444   0.946  0.34455    
#> as.factor(cal_time)10  0.229422   0.150841   1.521  0.12853    
#> as.factor(cal_time)11  0.478738   0.151047   3.169  0.00157 ** 
#> as.factor(cal_time)12  0.312381   0.151257   2.065  0.03911 *  
#> as.factor(cal_time)13  0.364877   0.229851   1.587  0.11267    
#> ---
#> Signif. codes:  0 '***' 0.001 '**' 0.01 '*' 0.05 '.' 0.1 ' ' 1
#> 
#> Residual standard error: 1.009 on 1210 degrees of freedom
#> Multiple R-squared:  0.4207, Adjusted R-squared:  0.414 
#> F-statistic: 62.76 on 14 and 1210 DF,  p-value: < 2.2e-16
```

### 3.2.5 Stepwise trend, \(\lambda=1.5\)

```
mod_lm_cal <- lm(response ~ as.factor(treatment) + as.factor(cal_time), flair_data_step_1.5)
summary(mod_lm_cal)
#> 
#> Call:
#> lm(formula = response ~ as.factor(treatment) + as.factor(cal_time), 
#>     data = flair_data_step_1.5)
#> 
#> Residuals:
#>     Min      1Q  Median      3Q     Max 
#> -3.3670 -0.6642 -0.0120  0.6680  3.6186 
#> 
#> Coefficients:
#>                        Estimate Std. Error t value Pr(>|t|)    
#> (Intercept)            0.006413   0.108257   0.059    0.953    
#> as.factor(treatment)1  0.777646   0.072654  10.703  < 2e-16 ***
#> as.factor(treatment)2  2.105402   0.086648  24.298  < 2e-16 ***
#> as.factor(cal_time)2  -0.058868   0.145328  -0.405    0.685    
#> as.factor(cal_time)3   0.101063   0.145283   0.696    0.487    
#> as.factor(cal_time)4  -0.070552   0.145290  -0.486    0.627    
#> as.factor(cal_time)5   0.019063   0.145502   0.131    0.896    
#> as.factor(cal_time)6  -0.004818   0.145290  -0.033    0.974    
#> as.factor(cal_time)7   0.807905   0.146203   5.526 4.01e-08 ***
#> as.factor(cal_time)8   1.520694   0.148141  10.265  < 2e-16 ***
#> as.factor(cal_time)9   1.359319   0.153221   8.872  < 2e-16 ***
#> as.factor(cal_time)10  1.446480   0.153625   9.416  < 2e-16 ***
#> as.factor(cal_time)11  1.695279   0.153835  11.020  < 2e-16 ***
#> as.factor(cal_time)12  1.528405   0.154049   9.922  < 2e-16 ***
#> as.factor(cal_time)13  1.586073   0.234093   6.775 1.93e-11 ***
#> ---
#> Signif. codes:  0 '***' 0.001 '**' 0.01 '*' 0.05 '.' 0.1 ' ' 1
#> 
#> Residual standard error: 1.027 on 1210 degrees of freedom
#> Multiple R-squared:  0.6097, Adjusted R-squared:  0.6052 
#> F-statistic:   135 on 14 and 1210 DF,  p-value: < 2.2e-16
```

### 3.2.6 Stepwise trend, \(\lambda=5\)

```
mod_lm_cal <- lm(response ~ as.factor(treatment) + as.factor(cal_time), flair_data_step_5)
summary(mod_lm_cal)
#> 
#> Call:
#> lm(formula = response ~ as.factor(treatment) + as.factor(cal_time), 
#>     data = flair_data_step_5)
#> 
#> Residuals:
#>     Min      1Q  Median      3Q     Max 
#> -5.7067 -0.7119  0.0281  0.7365  4.6341 
#> 
#> Coefficients:
#>                        Estimate Std. Error t value Pr(>|t|)    
#> (Intercept)            0.029132   0.127374   0.229    0.819    
#> as.factor(treatment)1  0.729306   0.085484   8.532   <2e-16 ***
#> as.factor(treatment)2  2.250231   0.101949  22.072   <2e-16 ***
#> as.factor(cal_time)2  -0.056451   0.170991  -0.330    0.741    
#> as.factor(cal_time)3   0.101063   0.170937   0.591    0.554    
#> as.factor(cal_time)4  -0.069585   0.170946  -0.407    0.684    
#> as.factor(cal_time)5   0.024380   0.171196   0.142    0.887    
#> as.factor(cal_time)6  -0.005785   0.170946  -0.034    0.973    
#> as.factor(cal_time)7   3.124905   0.172020  18.166   <2e-16 ***
#> as.factor(cal_time)8   4.966133   0.174301  28.492   <2e-16 ***
#> as.factor(cal_time)9   4.767087   0.180277  26.443   <2e-16 ***
#> as.factor(cal_time)10  4.854242   0.180753  26.856   <2e-16 ***
#> as.factor(cal_time)11  5.101593   0.181000  28.186   <2e-16 ***
#> as.factor(cal_time)12  4.933271   0.181252  27.218   <2e-16 ***
#> as.factor(cal_time)13  5.005422   0.275431  18.173   <2e-16 ***
#> ---
#> Signif. codes:  0 '***' 0.001 '**' 0.01 '*' 0.05 '.' 0.1 ' ' 1
#> 
#> Residual standard error: 1.209 on 1210 degrees of freedom
#> Multiple R-squared:  0.8472, Adjusted R-squared:  0.8454 
#> F-statistic: 479.1 on 14 and 1210 DF,  p-value: < 2.2e-16
```

## 3.3 Mixed model - period adjustment

### 3.3.1 Linear trend, \(\lambda=0.25\)

```
mod_mix_per <- lmer(response ~ as.factor(treatment) + (1 | period), flair_data_lin_0.25)
summary(mod_mix_per)
#> Linear mixed model fit by REML. t-tests use Satterthwaite's method [
#> lmerModLmerTest]
#> Formula: response ~ as.factor(treatment) + (1 | period)
#>    Data: flair_data_lin_0.25
#> 
#> REML criterion at convergence: 3509.7
#> 
#> Scaled residuals: 
#>      Min       1Q   Median       3Q      Max 
#> -3.02714 -0.67085  0.00734  0.64278  3.12309 
#> 
#> Random effects:
#>  Groups   Name        Variance Std.Dev.
#>  period   (Intercept) 0.01331  0.1154  
#>  Residual             1.01721  1.0086  
#> Number of obs: 1225, groups:  period, 3
#> 
#> Fixed effects:
#>                        Estimate Std. Error        df t value Pr(>|t|)    
#> (Intercept)             0.13627    0.08036   2.51942   1.696    0.206    
#> as.factor(treatment)1   0.78140    0.07020 829.63472  11.130   <2e-16 ***
#> as.factor(treatment)2   2.05652    0.08341 529.12605  24.655   <2e-16 ***
#> ---
#> Signif. codes:  0 '***' 0.001 '**' 0.01 '*' 0.05 '.' 0.1 ' ' 1
#> 
#> Correlation of Fixed Effects:
#>             (Intr) as.()1
#> as.fctr(t)1 -0.293       
#> as.fctr(t)2 -0.320  0.173
```

### 3.3.2 Linear trend, \(\lambda=1.5\)

```
mod_mix_per <- lmer(response ~ as.factor(treatment) + (1 | period), flair_data_lin_1.5)
summary(mod_mix_per)
#> Linear mixed model fit by REML. t-tests use Satterthwaite's method [
#> lmerModLmerTest]
#> Formula: response ~ as.factor(treatment) + (1 | period)
#>    Data: flair_data_lin_1.5
#> 
#> REML criterion at convergence: 3554.1
#> 
#> Scaled residuals: 
#>      Min       1Q   Median       3Q      Max 
#> -2.90139 -0.70341  0.00446  0.65664  2.97927 
#> 
#> Random effects:
#>  Groups   Name        Variance Std.Dev.
#>  period   (Intercept) 0.233    0.4827  
#>  Residual             1.050    1.0249  
#> Number of obs: 1225, groups:  period, 3
#> 
#> Fixed effects:
#>                        Estimate Std. Error        df t value Pr(>|t|)    
#> (Intercept)           8.429e-01  2.825e-01 2.049e+00   2.984   0.0936 .  
#> as.factor(treatment)1 7.996e-01  7.230e-02 1.222e+03  11.060   <2e-16 ***
#> as.factor(treatment)2 2.041e+00  8.658e-02 1.220e+03  23.568   <2e-16 ***
#> ---
#> Signif. codes:  0 '***' 0.001 '**' 0.01 '*' 0.05 '.' 0.1 ' ' 1
#> 
#> Correlation of Fixed Effects:
#>             (Intr) as.()1
#> as.fctr(t)1 -0.083       
#> as.fctr(t)2 -0.094  0.138
```

### 3.3.3 Linear trend, \(\lambda=5\)

```
mod_mix_per <- lmer(response ~ as.factor(treatment) + (1 | period), flair_data_lin_5)
summary(mod_mix_per)
#> Linear mixed model fit by REML. t-tests use Satterthwaite's method [
#> lmerModLmerTest]
#> Formula: response ~ as.factor(treatment) + (1 | period)
#>    Data: flair_data_lin_5
#> 
#> REML criterion at convergence: 3896.1
#> 
#> Scaled residuals: 
#>     Min      1Q  Median      3Q     Max 
#> -2.9327 -0.7044 -0.0160  0.7031  3.3354 
#> 
#> Random effects:
#>  Groups   Name        Variance Std.Dev.
#>  period   (Intercept) 2.297    1.516   
#>  Residual             1.385    1.177   
#> Number of obs: 1225, groups:  period, 3
#> 
#> Fixed effects:
#>                        Estimate Std. Error        df t value Pr(>|t|)    
#> (Intercept)           2.826e+00  8.766e-01 2.007e+00   3.223   0.0839 .  
#> as.factor(treatment)1 8.193e-01  8.310e-02 1.221e+03   9.859   <2e-16 ***
#> as.factor(treatment)2 2.029e+00  9.957e-02 1.221e+03  20.381   <2e-16 ***
#> ---
#> Signif. codes:  0 '***' 0.001 '**' 0.01 '*' 0.05 '.' 0.1 ' ' 1
#> 
#> Correlation of Fixed Effects:
#>             (Intr) as.()1
#> as.fctr(t)1 -0.031       
#> as.fctr(t)2 -0.035  0.135
```

### 3.3.4 Stepwise trend, \(\lambda=0.25\)

```
mod_mix_per <- lmer(response ~ as.factor(treatment) + (1 | period), flair_data_step_0.25)
summary(mod_mix_per)
#> Linear mixed model fit by REML. t-tests use Satterthwaite's method [
#> lmerModLmerTest]
#> Formula: response ~ as.factor(treatment) + (1 | period)
#>    Data: flair_data_step_0.25
#> 
#> REML criterion at convergence: 3509.3
#> 
#> Scaled residuals: 
#>     Min      1Q  Median      3Q     Max 
#> -3.0415 -0.6676  0.0000  0.6538  3.1844 
#> 
#> Random effects:
#>  Groups   Name        Variance Std.Dev.
#>  period   (Intercept) 0.02578  0.1606  
#>  Residual             1.01601  1.0080  
#> Number of obs: 1225, groups:  period, 3
#> 
#> Fixed effects:
#>                        Estimate Std. Error        df t value Pr(>|t|)    
#> (Intercept)              0.1571     0.1032    2.3695   1.523    0.248    
#> as.factor(treatment)1    0.7847     0.0706 1076.2502  11.115   <2e-16 ***
#> as.factor(treatment)2    2.0560     0.0842  889.4259  24.419   <2e-16 ***
#> ---
#> Signif. codes:  0 '***' 0.001 '**' 0.01 '*' 0.05 '.' 0.1 ' ' 1
#> 
#> Correlation of Fixed Effects:
#>             (Intr) as.()1
#> as.fctr(t)1 -0.226       
#> as.fctr(t)2 -0.251  0.156
```

### 3.3.5 Stepwise trend, \(\lambda=1.5\)

```
mod_mix_per <- lmer(response ~ as.factor(treatment) + (1 | period), flair_data_step_1.5)
summary(mod_mix_per)
#> Linear mixed model fit by REML. t-tests use Satterthwaite's method [
#> lmerModLmerTest]
#> Formula: response ~ as.factor(treatment) + (1 | period)
#>    Data: flair_data_step_1.5
#> 
#> REML criterion at convergence: 3515.8
#> 
#> Scaled residuals: 
#>     Min      1Q  Median      3Q     Max 
#> -3.0289 -0.6645  0.0052  0.6497  3.1868 
#> 
#> Random effects:
#>  Groups   Name        Variance Std.Dev.
#>  period   (Intercept) 0.7657   0.875   
#>  Residual             1.0160   1.008   
#> Number of obs: 1225, groups:  period, 3
#> 
#> Fixed effects:
#>                        Estimate Std. Error        df t value Pr(>|t|)    
#> (Intercept)           9.933e-01  5.073e-01 2.015e+00   1.958    0.188    
#> as.factor(treatment)1 7.950e-01  7.116e-02 1.221e+03  11.172   <2e-16 ***
#> as.factor(treatment)2 2.041e+00  8.525e-02 1.222e+03  23.938   <2e-16 ***
#> ---
#> Signif. codes:  0 '***' 0.001 '**' 0.01 '*' 0.05 '.' 0.1 ' ' 1
#> 
#> Correlation of Fixed Effects:
#>             (Intr) as.()1
#> as.fctr(t)1 -0.045       
#> as.fctr(t)2 -0.052  0.136
```

### 3.3.6 Stepwise trend, \(\lambda=5\)

```
mod_mix_per <- lmer(response ~ as.factor(treatment) + (1 | period), flair_data_step_5)
summary(mod_mix_per)
#> Linear mixed model fit by REML. t-tests use Satterthwaite's method [
#> lmerModLmerTest]
#> Formula: response ~ as.factor(treatment) + (1 | period)
#>    Data: flair_data_step_5
#> 
#> REML criterion at convergence: 3520.6
#> 
#> Scaled residuals: 
#>     Min      1Q  Median      3Q     Max 
#> -3.0268 -0.6660  0.0054  0.6476  3.1876 
#> 
#> Random effects:
#>  Groups   Name        Variance Std.Dev.
#>  period   (Intercept) 8.379    2.895   
#>  Residual             1.016    1.008   
#> Number of obs: 1225, groups:  period, 3
#> 
#> Fixed effects:
#>                        Estimate Std. Error        df t value Pr(>|t|)    
#> (Intercept)           3.328e+00  1.672e+00 2.001e+00    1.99    0.185    
#> as.factor(treatment)1 7.963e-01  7.118e-02 1.220e+03   11.19   <2e-16 ***
#> as.factor(treatment)2 2.038e+00  8.529e-02 1.220e+03   23.90   <2e-16 ***
#> ---
#> Signif. codes:  0 '***' 0.001 '**' 0.01 '*' 0.05 '.' 0.1 ' ' 1
#> 
#> Correlation of Fixed Effects:
#>             (Intr) as.()1
#> as.fctr(t)1 -0.014       
#> as.fctr(t)2 -0.016  0.135
```

## 3.4 Mixed model - calendar time adjustment

### 3.4.1 Linear trend, \(\lambda=0.25\)

```
mod_mix_cal <- lmer(response ~ as.factor(treatment) + (1 | cal_time), flair_data_lin_0.25)
summary(mod_mix_cal)
#> Linear mixed model fit by REML. t-tests use Satterthwaite's method [
#> lmerModLmerTest]
#> Formula: response ~ as.factor(treatment) + (1 | cal_time)
#>    Data: flair_data_lin_0.25
#> 
#> REML criterion at convergence: 3512.5
#> 
#> Scaled residuals: 
#>     Min      1Q  Median      3Q     Max 
#> -3.0489 -0.6825 -0.0078  0.6538  3.1356 
#> 
#> Random effects:
#>  Groups   Name        Variance Std.Dev.
#>  cal_time (Intercept) 0.006691 0.0818  
#>  Residual             1.017452 1.0087  
#> Number of obs: 1225, groups:  cal_time, 13
#> 
#> Fixed effects:
#>                        Estimate Std. Error        df t value Pr(>|t|)    
#> (Intercept)             0.13170    0.04791  26.97914   2.749   0.0105 *  
#> as.factor(treatment)1   0.74263    0.06777 756.48161  10.958   <2e-16 ***
#> as.factor(treatment)2   2.11030    0.07828 403.15624  26.959   <2e-16 ***
#> ---
#> Signif. codes:  0 '***' 0.001 '**' 0.01 '*' 0.05 '.' 0.1 ' ' 1
#> 
#> Correlation of Fixed Effects:
#>             (Intr) as.()1
#> as.fctr(t)1 -0.541       
#> as.fctr(t)2 -0.475  0.285
```

### 3.4.2 Linear trend, \(\lambda=1.5\)

```
mod_mix_cal <- lmer(response ~ as.factor(treatment) + (1 | cal_time), flair_data_lin_1.5)
summary(mod_mix_cal)
#> Linear mixed model fit by REML. t-tests use Satterthwaite's method [
#> lmerModLmerTest]
#> Formula: response ~ as.factor(treatment) + (1 | cal_time)
#>    Data: flair_data_lin_1.5
#> 
#> REML criterion at convergence: 3543.7
#> 
#> Scaled residuals: 
#>      Min       1Q   Median       3Q      Max 
#> -2.95359 -0.68168 -0.00961  0.66094  3.14092 
#> 
#> Random effects:
#>  Groups   Name        Variance Std.Dev.
#>  cal_time (Intercept) 0.2416   0.4915  
#>  Residual             1.0174   1.0087  
#> Number of obs: 1225, groups:  cal_time, 13
#> 
#> Fixed effects:
#>                        Estimate Std. Error        df t value Pr(>|t|)    
#> (Intercept)           7.918e-01  1.430e-01 1.233e+01   5.536 0.000117 ***
#> as.factor(treatment)1 7.786e-01  7.100e-02 1.222e+03  10.966  < 2e-16 ***
#> as.factor(treatment)2 2.077e+00  8.448e-02 1.220e+03  24.591  < 2e-16 ***
#> ---
#> Signif. codes:  0 '***' 0.001 '**' 0.01 '*' 0.05 '.' 0.1 ' ' 1
#> 
#> Correlation of Fixed Effects:
#>             (Intr) as.()1
#> as.fctr(t)1 -0.169       
#> as.fctr(t)2 -0.154  0.154
```

### 3.4.3 Linear trend, \(\lambda=5\)

```
mod_mix_cal <- lmer(response ~ as.factor(treatment) + (1 | cal_time), flair_data_lin_5)
summary(mod_mix_cal)
#> Linear mixed model fit by REML. t-tests use Satterthwaite's method [
#> lmerModLmerTest]
#> Formula: response ~ as.factor(treatment) + (1 | cal_time)
#>    Data: flair_data_lin_5
#> 
#> REML criterion at convergence: 3583.8
#> 
#> Scaled residuals: 
#>     Min      1Q  Median      3Q     Max 
#> -2.8314 -0.6855  0.0099  0.6669  3.2159 
#> 
#> Random effects:
#>  Groups   Name        Variance Std.Dev.
#>  cal_time (Intercept) 2.569    1.603   
#>  Residual             1.028    1.014   
#> Number of obs: 1225, groups:  cal_time, 13
#> 
#> Fixed effects:
#>                        Estimate Std. Error        df t value Pr(>|t|)    
#> (Intercept)           2.641e+00  4.467e-01 1.203e+01   5.912 7.05e-05 ***
#> as.factor(treatment)1 7.975e-01  7.167e-02 1.212e+03  11.128  < 2e-16 ***
#> as.factor(treatment)2 2.065e+00  8.545e-02 1.213e+03  24.170  < 2e-16 ***
#> ---
#> Signif. codes:  0 '***' 0.001 '**' 0.01 '*' 0.05 '.' 0.1 ' ' 1
#> 
#> Correlation of Fixed Effects:
#>             (Intr) as.()1
#> as.fctr(t)1 -0.054       
#> as.fctr(t)2 -0.049  0.143
```

### 3.4.4 Stepwise trend, \(\lambda=0.25\)

```
mod_mix_cal <- lmer(response ~ as.factor(treatment) + (1 | cal_time), flair_data_step_0.25)
summary(mod_mix_cal)
#> Linear mixed model fit by REML. t-tests use Satterthwaite's method [
#> lmerModLmerTest]
#> Formula: response ~ as.factor(treatment) + (1 | cal_time)
#>    Data: flair_data_step_0.25
#> 
#> REML criterion at convergence: 3517.2
#> 
#> Scaled residuals: 
#>      Min       1Q   Median       3Q      Max 
#> -3.06888 -0.67274 -0.01447  0.66071  3.16005 
#> 
#> Random effects:
#>  Groups   Name        Variance Std.Dev.
#>  cal_time (Intercept) 0.012    0.1095  
#>  Residual             1.019    1.0094  
#> Number of obs: 1225, groups:  cal_time, 13
#> 
#> Fixed effects:
#>                        Estimate Std. Error        df t value Pr(>|t|)    
#> (Intercept)             0.12473    0.05214  20.10136   2.392   0.0266 *  
#> as.factor(treatment)1   0.73516    0.06848 831.73703  10.735   <2e-16 ***
#> as.factor(treatment)2   2.13996    0.07964 482.32726  26.870   <2e-16 ***
#> ---
#> Signif. codes:  0 '***' 0.001 '**' 0.01 '*' 0.05 '.' 0.1 ' ' 1
#> 
#> Correlation of Fixed Effects:
#>             (Intr) as.()1
#> as.fctr(t)1 -0.492       
#> as.fctr(t)2 -0.433  0.256
```

### 3.4.5 Stepwise trend, \(\lambda=1.5\)

```
mod_mix_cal <- lmer(response ~ as.factor(treatment) + (1 | cal_time), flair_data_step_1.5)
summary(mod_mix_cal)
#> Linear mixed model fit by REML. t-tests use Satterthwaite's method [
#> lmerModLmerTest]
#> Formula: response ~ as.factor(treatment) + (1 | cal_time)
#>    Data: flair_data_step_1.5
#> 
#> REML criterion at convergence: 3597.9
#> 
#> Scaled residuals: 
#>     Min      1Q  Median      3Q     Max 
#> -3.2786 -0.6388 -0.0181  0.6482  3.4981 
#> 
#> Random effects:
#>  Groups   Name        Variance Std.Dev.
#>  cal_time (Intercept) 0.5627   0.7501  
#>  Residual             1.0555   1.0274  
#> Number of obs: 1225, groups:  cal_time, 13
#> 
#> Fixed effects:
#>                        Estimate Std. Error        df t value Pr(>|t|)    
#> (Intercept)           7.661e-01  2.127e-01 1.223e+01   3.602  0.00353 ** 
#> as.factor(treatment)1 7.631e-01  7.250e-02 1.218e+03  10.525  < 2e-16 ***
#> as.factor(treatment)2 2.129e+00  8.637e-02 1.221e+03  24.649  < 2e-16 ***
#> ---
#> Signif. codes:  0 '***' 0.001 '**' 0.01 '*' 0.05 '.' 0.1 ' ' 1
#> 
#> Correlation of Fixed Effects:
#>             (Intr) as.()1
#> as.fctr(t)1 -0.115       
#> as.fctr(t)2 -0.105  0.147
```

### 3.4.6 Stepwise trend, \(\lambda=5\)

```
mod_mix_cal <- lmer(response ~ as.factor(treatment) + (1 | cal_time), flair_data_step_5)
summary(mod_mix_cal)
#> Linear mixed model fit by REML. t-tests use Satterthwaite's method [
#> lmerModLmerTest]
#> Formula: response ~ as.factor(treatment) + (1 | cal_time)
#>    Data: flair_data_step_5
#> 
#> REML criterion at convergence: 4019.7
#> 
#> Scaled residuals: 
#>     Min      1Q  Median      3Q     Max 
#> -4.7209 -0.5827  0.0264  0.6061  3.8260 
#> 
#> Random effects:
#>  Groups   Name        Variance Std.Dev.
#>  cal_time (Intercept) 6.104    2.471   
#>  Residual             1.461    1.209   
#> Number of obs: 1225, groups:  cal_time, 13
#> 
#> Fixed effects:
#>                        Estimate Std. Error        df t value Pr(>|t|)    
#> (Intercept)           2.546e+00  6.872e-01 1.203e+01   3.705  0.00299 ** 
#> as.factor(treatment)1 7.232e-01  8.546e-02 1.211e+03   8.463  < 2e-16 ***
#> as.factor(treatment)2 2.260e+00  1.019e-01 1.212e+03  22.180  < 2e-16 ***
#> ---
#> Signif. codes:  0 '***' 0.001 '**' 0.01 '*' 0.05 '.' 0.1 ' ' 1
#> 
#> Correlation of Fixed Effects:
#>             (Intr) as.()1
#> as.fctr(t)1 -0.042       
#> as.fctr(t)2 -0.038  0.142
```

## 3.5 Mixed model (AR1) - period adjustment

### 3.5.1 Linear trend, \(\lambda=0.25\)

```
mod_mix_ar1_per <- fitme(response ~ as.factor(treatment) + AR1(1 | period), flair_data_lin_0.25)

summary.HLfit(mod_mix_ar1_per)
#> formula: response ~ as.factor(treatment) + AR1(1 | period)
#> ML: Estimation of corrPars, lambda and phi by ML.
#>     Estimation of fixed effects by ML.
#> Estimation of lambda and phi by 'outer' ML, maximizing logL.
#> family: gaussian( link = identity ) 
#>  ------------ Fixed effects (beta) ------------
#>                       Estimate Cond. SE t-value
#> (Intercept)             0.1325  0.06173   2.147
#> as.factor(treatment)1   0.7709  0.06949  11.094
#> as.factor(treatment)2   2.0705  0.08206  25.232
#>  --------------- Random effects ---------------
#> Family: gaussian( link = identity ) 
#>                    --- Correlation parameters:
#>    1.ARphi 
#> -0.1826209 
#>            --- Variance parameters ('lambda'):
#> lambda = var(u) for u ~ Gaussian; 
#>    period  :  0.006827  
#> # of obs: 1225; # of groups: period, 3 
#>  -------------- Residual variance  ------------
#> phi estimate was 1.01573 
#>  ------------- Likelihood values  -------------
#>                         logLik
#> logL       (p_v(h)): -1749.646
```

```
res <- summary.HLfit(mod_mix_ar1_per, verbose = FALSE)

IC <- get_any_IC(mod_mix_ar1_per, verbose = FALSE)
IC
#>        marginal AIC:     conditional AIC:      dispersion AIC: 
#>             3511.293             3504.095             3505.293 
#>        effective df: 
#>             1220.663
```

```
eff_df <- IC["       effective df:"] # effective degrees of freedom

2*(1-pt(abs(res$beta_table["as.factor(treatment)2", "t-value"]), eff_df)) # p-value
#> [1] 0
```

### 3.5.2 Linear trend, \(\lambda=1.5\)

```
mod_mix_ar1_per <- fitme(response ~ as.factor(treatment) + AR1(1 | period), flair_data_lin_1.5)

summary.HLfit(mod_mix_ar1_per)
#> formula: response ~ as.factor(treatment) + AR1(1 | period)
#> ML: Estimation of corrPars, lambda and phi by ML.
#>     Estimation of fixed effects by ML.
#> Estimation of lambda and phi by 'outer' ML, maximizing logL.
#> family: gaussian( link = identity ) 
#>  ------------ Fixed effects (beta) ------------
#>                       Estimate Cond. SE t-value
#> (Intercept)             0.8423  0.23050   3.654
#> as.factor(treatment)1   0.7973  0.07220  11.043
#> as.factor(treatment)2   2.0440  0.08644  23.646
#>  --------------- Random effects ---------------
#> Family: gaussian( link = identity ) 
#>                    --- Correlation parameters:
#>      1.ARphi 
#> -0.002661492 
#>            --- Variance parameters ('lambda'):
#> lambda = var(u) for u ~ Gaussian; 
#>    period  :  0.1535  
#> # of obs: 1225; # of groups: period, 3 
#>  -------------- Residual variance  ------------
#> phi estimate was 1.04869 
#>  ------------- Likelihood values  -------------
#>                         logLik
#> logL       (p_v(h)): -1773.333
```

```
res <- summary.HLfit(mod_mix_ar1_per, verbose = FALSE)

IC <- get_any_IC(mod_mix_ar1_per, verbose = FALSE)
IC
#>        marginal AIC:     conditional AIC:      dispersion AIC: 
#>             3558.665             3543.595             3552.665 
#>        effective df: 
#>             1220.049
```

```
eff_df <- IC["       effective df:"] # effective degrees of freedom

2*(1-pt(abs(res$beta_table["as.factor(treatment)2", "t-value"]), eff_df)) # p-value
#> [1] 0
```

### 3.5.3 Linear trend, \(\lambda=5\)

```
mod_mix_ar1_per <- fitme(response ~ as.factor(treatment) + AR1(1 | period), flair_data_lin_5)

summary.HLfit(mod_mix_ar1_per)
#> formula: response ~ as.factor(treatment) + AR1(1 | period)
#> ML: Estimation of corrPars, lambda and phi by ML.
#>     Estimation of fixed effects by ML.
#> Estimation of lambda and phi by 'outer' ML, maximizing logL.
#> family: gaussian( link = identity ) 
#>  ------------ Fixed effects (beta) ------------
#>                       Estimate Cond. SE t-value
#> (Intercept)             2.8255  0.71306   3.962
#> as.factor(treatment)1   0.8184  0.08302   9.857
#> as.factor(treatment)2   2.0307  0.09947  20.415
#>  --------------- Random effects ---------------
#> Family: gaussian( link = identity ) 
#>                    --- Correlation parameters:
#>      1.ARphi 
#> -0.005928153 
#>            --- Variance parameters ('lambda'):
#> lambda = var(u) for u ~ Gaussian; 
#>    period  :  1.529  
#> # of obs: 1225; # of groups: period, 3 
#>  -------------- Residual variance  ------------
#> phi estimate was 1.38281 
#>  ------------- Likelihood values  -------------
#>                         logLik
#> logL       (p_v(h)): -1945.746
```

```
res <- summary.HLfit(mod_mix_ar1_per, verbose = FALSE)

IC <- get_any_IC(mod_mix_ar1_per, verbose = FALSE)
IC
#>        marginal AIC:     conditional AIC:      dispersion AIC: 
#>             3903.492             3882.441             3897.492 
#>        effective df: 
#>             1220.007
```

```
eff_df <- IC["       effective df:"] # effective degrees of freedom

2*(1-pt(abs(res$beta_table["as.factor(treatment)2", "t-value"]), eff_df)) # p-value
#> [1] 0
```

### 3.5.4 Stepwise trend, \(\lambda=0.25\)

```
mod_mix_ar1_per <- fitme(response ~ as.factor(treatment) + AR1(1 | period), flair_data_step_0.25)

summary.HLfit(mod_mix_ar1_per)
#> formula: response ~ as.factor(treatment) + AR1(1 | period)
#> ML: Estimation of corrPars, lambda and phi by ML.
#>     Estimation of fixed effects by ML.
#> Estimation of lambda and phi by 'outer' ML, maximizing logL.
#> family: gaussian( link = identity ) 
#>  ------------ Fixed effects (beta) ------------
#>                       Estimate Cond. SE t-value
#> (Intercept)             0.1547  0.08428   1.836
#> as.factor(treatment)1   0.7780  0.07022  11.079
#> as.factor(treatment)2   2.0664  0.08351  24.743
#>  --------------- Random effects ---------------
#> Family: gaussian( link = identity ) 
#>                    --- Correlation parameters:
#>      1.ARphi 
#> -0.006255264 
#>            --- Variance parameters ('lambda'):
#> lambda = var(u) for u ~ Gaussian; 
#>    period  :  0.01536  
#> # of obs: 1225; # of groups: period, 3 
#>  -------------- Residual variance  ------------
#> phi estimate was 1.01443 
#>  ------------- Likelihood values  -------------
#>                         logLik
#> logL       (p_v(h)): -1749.822
```

```
res <- summary.HLfit(mod_mix_ar1_per, verbose = FALSE)

IC <- get_any_IC(mod_mix_ar1_per, verbose = FALSE)
IC
#>        marginal AIC:     conditional AIC:      dispersion AIC: 
#>             3511.644             3502.647             3505.644 
#>        effective df: 
#>             1220.390
```

```
eff_df <- IC["       effective df:"] # effective degrees of freedom

2*(1-pt(abs(res$beta_table["as.factor(treatment)2", "t-value"]), eff_df)) # p-value
#> [1] 0
```

### 3.5.5 Stepwise trend, \(\lambda=1.5\)

```
mod_mix_ar1_per <- fitme(response ~ as.factor(treatment) + AR1(1 | period), flair_data_step_1.5)

summary.HLfit(mod_mix_ar1_per)
#> formula: response ~ as.factor(treatment) + AR1(1 | period)
#> ML: Estimation of corrPars, lambda and phi by ML.
#>     Estimation of fixed effects by ML.
#> Estimation of lambda and phi by 'outer' ML, maximizing logL.
#> family: gaussian( link = identity ) 
#>  ------------ Fixed effects (beta) ------------
#>                       Estimate Cond. SE t-value
#> (Intercept)             1.0301  0.33912   3.038
#> as.factor(treatment)1   0.7937  0.07109  11.164
#> as.factor(treatment)2   2.0428  0.08516  23.987
#>  --------------- Random effects ---------------
#> Family: gaussian( link = identity ) 
#>                    --- Correlation parameters:
#>    1.ARphi 
#> -0.2874824 
#>            --- Variance parameters ('lambda'):
#> lambda = var(u) for u ~ Gaussian; 
#>    period  :  0.5202  
#> # of obs: 1225; # of groups: period, 3 
#>  -------------- Residual variance  ------------
#> phi estimate was 1.01436 
#>  ------------- Likelihood values  -------------
#>                         logLik
#> logL       (p_v(h)): -1754.724
```

```
res <- summary.HLfit(mod_mix_ar1_per, verbose = FALSE)

IC <- get_any_IC(mod_mix_ar1_per, verbose = FALSE)
IC
#>        marginal AIC:     conditional AIC:      dispersion AIC: 
#>             3521.448             3502.862             3515.448 
#>        effective df: 
#>             1220.013
```

```
eff_df <- IC["       effective df:"] # effective degrees of freedom

2*(1-pt(abs(res$beta_table["as.factor(treatment)2", "t-value"]), eff_df)) # p-value
#> [1] 0
```

### 3.5.6 Stepwise trend, \(\lambda=5\)

```
mod_mix_ar1_per <- fitme(response ~ as.factor(treatment) + AR1(1 | period), flair_data_step_5)

summary.HLfit(mod_mix_ar1_per)
#> formula: response ~ as.factor(treatment) + AR1(1 | period)
#> ML: Estimation of corrPars, lambda and phi by ML.
#>     Estimation of fixed effects by ML.
#> Estimation of lambda and phi by 'outer' ML, maximizing logL.
#> family: gaussian( link = identity ) 
#>  ------------ Fixed effects (beta) ------------
#>                       Estimate Cond. SE t-value
#> (Intercept)             3.4950  1.05651   3.308
#> as.factor(treatment)1   0.7959  0.07112  11.191
#> as.factor(treatment)2   2.0388  0.08522  23.924
#>  --------------- Random effects ---------------
#> Family: gaussian( link = identity ) 
#>                    --- Correlation parameters:
#>    1.ARphi 
#> -0.3524192 
#>            --- Variance parameters ('lambda'):
#> lambda = var(u) for u ~ Gaussian; 
#>    period  :  5.767  
#> # of obs: 1225; # of groups: period, 3 
#>  -------------- Residual variance  ------------
#> phi estimate was 1.01436 
#>  ------------- Likelihood values  -------------
#>                         logLik
#> logL       (p_v(h)): -1758.277
```

```
res <- summary.HLfit(mod_mix_ar1_per, verbose = FALSE)

IC <- get_any_IC(mod_mix_ar1_per, verbose = FALSE)
IC
#>        marginal AIC:     conditional AIC:      dispersion AIC: 
#>             3528.554             3502.867             3522.554 
#>        effective df: 
#>             1220.001
```

```
eff_df <- IC["       effective df:"] # effective degrees of freedom

2*(1-pt(abs(res$beta_table["as.factor(treatment)2", "t-value"]), eff_df)) # p-value
#> [1] 0
```

## 3.6 Mixed model (AR1) - calendar time adjustment

### 3.6.1 Linear trend, \(\lambda=0.25\)

```
mod_mix_ar1_cal <- fitme(response ~ as.factor(treatment) + AR1(1 | cal_time), flair_data_lin_0.25)

summary.HLfit(mod_mix_ar1_cal)
#> formula: response ~ as.factor(treatment) + AR1(1 | cal_time)
#> ML: Estimation of corrPars, lambda and phi by ML.
#>     Estimation of fixed effects by ML.
#> Estimation of lambda and phi by 'outer' ML, maximizing logL.
#> family: gaussian( link = identity ) 
#>  ------------ Fixed effects (beta) ------------
#>                       Estimate Cond. SE t-value
#> (Intercept)             0.1399  0.07095   1.971
#> as.factor(treatment)1   0.7641  0.06889  11.091
#> as.factor(treatment)2   2.0779  0.08078  25.724
#>  --------------- Random effects ---------------
#> Family: gaussian( link = identity ) 
#>                    --- Correlation parameters:
#>   1.ARphi 
#> 0.7425427 
#>            --- Variance parameters ('lambda'):
#> lambda = var(u) for u ~ Gaussian; 
#>    cal_time  :  0.008488  
#> # of obs: 1225; # of groups: cal_time, 13 
#>  -------------- Residual variance  ------------
#> phi estimate was 1.01444 
#>  ------------- Likelihood values  -------------
#>                         logLik
#> logL       (p_v(h)): -1749.877
```

```
res <- summary.HLfit(mod_mix_ar1_cal, verbose = FALSE)

IC <- get_any_IC(mod_mix_ar1_cal, verbose = FALSE)
IC
#>        marginal AIC:     conditional AIC:      dispersion AIC: 
#>             3511.753             3504.095             3505.753 
#>        effective df: 
#>             1218.968
```

```
eff_df <- IC["       effective df:"] # effective degrees of freedom

2*(1-pt(abs(res$beta_table["as.factor(treatment)2", "t-value"]), eff_df)) # p-value
#> [1] 0
```

### 3.6.2 Linear trend, \(\lambda=1.5\)

```
mod_mix_ar1_cal <- fitme(response ~ as.factor(treatment) + AR1(1 | cal_time), flair_data_lin_1.5)

summary.HLfit(mod_mix_ar1_cal)
#> formula: response ~ as.factor(treatment) + AR1(1 | cal_time)
#> ML: Estimation of corrPars, lambda and phi by ML.
#>     Estimation of fixed effects by ML.
#> Estimation of lambda and phi by 'outer' ML, maximizing logL.
#> family: gaussian( link = identity ) 
#>  ------------ Fixed effects (beta) ------------
#>                       Estimate Cond. SE t-value
#> (Intercept)             0.7999  0.58222   1.374
#> as.factor(treatment)1   0.7893  0.07082  11.145
#> as.factor(treatment)2   2.0566  0.08431  24.393
#>  --------------- Random effects ---------------
#> Family: gaussian( link = identity ) 
#>                    --- Correlation parameters:
#>  1.ARphi 
#> 0.959724 
#>            --- Variance parameters ('lambda'):
#> lambda = var(u) for u ~ Gaussian; 
#>    cal_time  :  0.4155  
#> # of obs: 1225; # of groups: cal_time, 13 
#>  -------------- Residual variance  ------------
#> phi estimate was 1.01378 
#>  ------------- Likelihood values  -------------
#>                         logLik
#> logL       (p_v(h)): -1757.632
```

```
res <- summary.HLfit(mod_mix_ar1_cal, verbose = FALSE)

IC <- get_any_IC(mod_mix_ar1_cal, verbose = FALSE)
IC
#>        marginal AIC:     conditional AIC:      dispersion AIC: 
#>             3527.264             3507.928             3521.264 
#>        effective df: 
#>             1214.225
```

```
eff_df <- IC["       effective df:"] # effective degrees of freedom

2*(1-pt(abs(res$beta_table["as.factor(treatment)2", "t-value"]), eff_df)) # p-value
#> [1] 0
```

### 3.6.3 Linear trend, \(\lambda=5\)

```
mod_mix_ar1_cal <- fitme(response ~ as.factor(treatment) + AR1(1 | cal_time), flair_data_lin_5)

summary.HLfit(mod_mix_ar1_cal)
#> formula: response ~ as.factor(treatment) + AR1(1 | cal_time)
#> ML: Estimation of corrPars, lambda and phi by ML.
#>     Estimation of fixed effects by ML.
#> Estimation of lambda and phi by 'outer' ML, maximizing logL.
#> family: gaussian( link = identity ) 
#>  ------------ Fixed effects (beta) ------------
#>                       Estimate Cond. SE t-value
#> (Intercept)             2.6111  2.12112   1.231
#> as.factor(treatment)1   0.7993  0.07153  11.174
#> as.factor(treatment)2   2.0608  0.08528  24.164
#>  --------------- Random effects ---------------
#> Family: gaussian( link = identity ) 
#>                    --- Correlation parameters:
#>   1.ARphi 
#> 0.9802344 
#>            --- Variance parameters ('lambda'):
#> lambda = var(u) for u ~ Gaussian; 
#>    cal_time  :  5.027  
#> # of obs: 1225; # of groups: cal_time, 13 
#>  -------------- Residual variance  ------------
#> phi estimate was 1.02539 
#>  ------------- Likelihood values  -------------
#>                         logLik
#> logL       (p_v(h)): -1774.327
```

```
res <- summary.HLfit(mod_mix_ar1_cal, verbose = FALSE)

IC <- get_any_IC(mod_mix_ar1_cal, verbose = FALSE)
IC
#>        marginal AIC:     conditional AIC:      dispersion AIC: 
#>             3560.654             3524.922             3554.654 
#>        effective df: 
#>             1211.188
```

```
eff_df <- IC["       effective df:"] # effective degrees of freedom

2*(1-pt(abs(res$beta_table["as.factor(treatment)2", "t-value"]), eff_df)) # p-value
#> [1] 0
```

### 3.6.4 Stepwise trend, \(\lambda=0.25\)

```
mod_mix_ar1_cal <- fitme(response ~ as.factor(treatment) + AR1(1 | cal_time), flair_data_step_0.25)

summary.HLfit(mod_mix_ar1_cal)
#> formula: response ~ as.factor(treatment) + AR1(1 | cal_time)
#> ML: Estimation of corrPars, lambda and phi by ML.
#>     Estimation of fixed effects by ML.
#> Estimation of lambda and phi by 'outer' ML, maximizing logL.
#> family: gaussian( link = identity ) 
#>  ------------ Fixed effects (beta) ------------
#>                       Estimate Cond. SE t-value
#> (Intercept)             0.1392  0.10269   1.356
#> as.factor(treatment)1   0.7665  0.06955  11.022
#> as.factor(treatment)2   2.0932  0.08204  25.516
#>  --------------- Random effects ---------------
#> Family: gaussian( link = identity ) 
#>                    --- Correlation parameters:
#>   1.ARphi 
#> 0.8329124 
#>            --- Variance parameters ('lambda'):
#> lambda = var(u) for u ~ Gaussian; 
#>    cal_time  :  0.01732  
#> # of obs: 1225; # of groups: cal_time, 13 
#>  -------------- Residual variance  ------------
#> phi estimate was 1.01474 
#>  ------------- Likelihood values  -------------
#>                         logLik
#> logL       (p_v(h)): -1751.145
```

```
res <- summary.HLfit(mod_mix_ar1_cal, verbose = FALSE)

IC <- get_any_IC(mod_mix_ar1_cal, verbose = FALSE)
IC
#>        marginal AIC:     conditional AIC:      dispersion AIC: 
#>             3514.290             3505.026             3508.290 
#>        effective df: 
#>             1218.310
```

```
eff_df <- IC["       effective df:"] # effective degrees of freedom

2*(1-pt(abs(res$beta_table["as.factor(treatment)2", "t-value"]), eff_df)) # p-value
#> [1] 0
```

### 3.6.5 Stepwise trend, \(\lambda=1.5\)

```
mod_mix_ar1_cal <- fitme(response ~ as.factor(treatment) + AR1(1 | cal_time), flair_data_step_1.5)

summary.HLfit(mod_mix_ar1_cal)
#> formula: response ~ as.factor(treatment) + AR1(1 | cal_time)
#> ML: Estimation of corrPars, lambda and phi by ML.
#>     Estimation of fixed effects by ML.
#> Estimation of lambda and phi by 'outer' ML, maximizing logL.
#> family: gaussian( link = identity ) 
#>  ------------ Fixed effects (beta) ------------
#>                       Estimate Cond. SE t-value
#> (Intercept)             0.7749  0.59499   1.302
#> as.factor(treatment)1   0.7737  0.07241  10.685
#> as.factor(treatment)2   2.1217  0.08630  24.586
#>  --------------- Random effects ---------------
#> Family: gaussian( link = identity ) 
#>                    --- Correlation parameters:
#>   1.ARphi 
#> 0.9034115 
#>            --- Variance parameters ('lambda'):
#> lambda = var(u) for u ~ Gaussian; 
#>    cal_time  :  0.561  
#> # of obs: 1225; # of groups: cal_time, 13 
#>  -------------- Residual variance  ------------
#> phi estimate was 1.05292 
#>  ------------- Likelihood values  -------------
#>                         logLik
#> logL       (p_v(h)): -1785.884
```

```
res <- summary.HLfit(mod_mix_ar1_cal, verbose = FALSE)

IC <- get_any_IC(mod_mix_ar1_cal, verbose = FALSE)
IC
#>        marginal AIC:     conditional AIC:      dispersion AIC: 
#>             3583.767             3556.618             3577.767 
#>        effective df: 
#>             1211.955
```

```
eff_df <- IC["       effective df:"] # effective degrees of freedom

2*(1-pt(abs(res$beta_table["as.factor(treatment)2", "t-value"]), eff_df)) # p-value
#> [1] 0
```

### 3.6.6 Stepwise trend, \(\lambda=5\)

```
mod_mix_ar1_cal <- fitme(response ~ as.factor(treatment) + AR1(1 | cal_time), flair_data_step_5)

summary.HLfit(mod_mix_ar1_cal)
#> formula: response ~ as.factor(treatment) + AR1(1 | cal_time)
#> ML: Estimation of corrPars, lambda and phi by ML.
#>     Estimation of fixed effects by ML.
#> Estimation of lambda and phi by 'outer' ML, maximizing logL.
#> family: gaussian( link = identity ) 
#>  ------------ Fixed effects (beta) ------------
#>                       Estimate Cond. SE t-value
#> (Intercept)             2.5328  1.92235   1.318
#> as.factor(treatment)1   0.7281  0.08538   8.528
#> as.factor(treatment)2   2.2584  0.10182  22.181
#>  --------------- Random effects ---------------
#> Family: gaussian( link = identity ) 
#>                    --- Correlation parameters:
#>   1.ARphi 
#> 0.9051542 
#>            --- Variance parameters ('lambda'):
#> lambda = var(u) for u ~ Gaussian; 
#>    cal_time  :  5.889  
#> # of obs: 1225; # of groups: cal_time, 13 
#>  -------------- Residual variance  ------------
#> phi estimate was 1.45839 
#>  ------------- Likelihood values  -------------
#>                         logLik
#> logL       (p_v(h)): -1997.531
```

```
res <- summary.HLfit(mod_mix_ar1_cal, verbose = FALSE)

IC <- get_any_IC(mod_mix_ar1_cal, verbose = FALSE)
IC
#>        marginal AIC:     conditional AIC:      dispersion AIC: 
#>             4007.061             3957.303             4001.061 
#>        effective df: 
#>             1210.328
```

```
eff_df <- IC["       effective df:"] # effective degrees of freedom

2*(1-pt(abs(res$beta_table["as.factor(treatment)2", "t-value"]), eff_df)) # p-value
#> [1] 0
```

## 3.7 Splines with knotes according to periods

### 3.7.1 Linear trend, \(\lambda=0.25\)

```
period_start <- c(max(which(flair_data_lin_0.25$period==1)), max(which(flair_data_lin_0.25$period==2)))

mod_splines_per <- lm(response ~ as.factor(treatment) + bs(j, knots = period_start, degree = 3), flair_data_lin_0.25)
summary(mod_splines_per)
#> 
#> Call:
#> lm(formula = response ~ as.factor(treatment) + bs(j, knots = period_start, 
#>     degree = 3), data = flair_data_lin_0.25)
#> 
#> Residuals:
#>     Min      1Q  Median      3Q     Max 
#> -3.0522 -0.6708  0.0071  0.6573  3.2133 
#> 
#> Coefficients:
#>                                          Estimate Std. Error t value Pr(>|t|)
#> (Intercept)                              -0.01906    0.14676  -0.130   0.8967
#> as.factor(treatment)1                     0.80025    0.07096  11.277   <2e-16
#> as.factor(treatment)2                     2.03626    0.08474  24.028   <2e-16
#> bs(j, knots = period_start, degree = 3)1  0.09358    0.32444   0.288   0.7731
#> bs(j, knots = period_start, degree = 3)2  0.09472    0.19092   0.496   0.6199
#> bs(j, knots = period_start, degree = 3)3  0.05612    0.25548   0.220   0.8262
#> bs(j, knots = period_start, degree = 3)4  0.58128    0.24364   2.386   0.0172
#> bs(j, knots = period_start, degree = 3)5  0.28614    0.23568   1.214   0.2249
#>                                             
#> (Intercept)                                 
#> as.factor(treatment)1                    ***
#> as.factor(treatment)2                    ***
#> bs(j, knots = period_start, degree = 3)1    
#> bs(j, knots = period_start, degree = 3)2    
#> bs(j, knots = period_start, degree = 3)3    
#> bs(j, knots = period_start, degree = 3)4 *  
#> bs(j, knots = period_start, degree = 3)5    
#> ---
#> Signif. codes:  0 '***' 0.001 '**' 0.01 '*' 0.05 '.' 0.1 ' ' 1
#> 
#> Residual standard error: 1.008 on 1217 degrees of freedom
#> Multiple R-squared:  0.4019, Adjusted R-squared:  0.3985 
#> F-statistic: 116.8 on 7 and 1217 DF,  p-value: < 2.2e-16
```

### 3.7.2 Linear trend, \(\lambda=1.5\)

```
period_start <- c(max(which(flair_data_lin_1.5$period==1)), max(which(flair_data_lin_1.5$period==2)))

mod_splines_per <- lm(response ~ as.factor(treatment) + bs(j, knots = period_start, degree = 3), flair_data_lin_1.5)
summary(mod_splines_per)
#> 
#> Call:
#> lm(formula = response ~ as.factor(treatment) + bs(j, knots = period_start, 
#>     degree = 3), data = flair_data_lin_1.5)
#> 
#> Residuals:
#>     Min      1Q  Median      3Q     Max 
#> -3.0522 -0.6708  0.0071  0.6573  3.2133 
#> 
#> Coefficients:
#>                                          Estimate Std. Error t value Pr(>|t|)
#> (Intercept)                              -0.01906    0.14676  -0.130 0.896702
#> as.factor(treatment)1                     0.80025    0.07096  11.277  < 2e-16
#> as.factor(treatment)2                     2.03626    0.08474  24.028  < 2e-16
#> bs(j, knots = period_start, degree = 3)1  0.30855    0.32444   0.951 0.341789
#> bs(j, knots = period_start, degree = 3)2  0.59506    0.19092   3.117 0.001871
#> bs(j, knots = period_start, degree = 3)3  0.97279    0.25548   3.808 0.000147
#> bs(j, knots = period_start, degree = 3)4  1.69931    0.24364   6.975 5.02e-12
#> bs(j, knots = period_start, degree = 3)5  1.53512    0.23568   6.514 1.07e-10
#>                                             
#> (Intercept)                                 
#> as.factor(treatment)1                    ***
#> as.factor(treatment)2                    ***
#> bs(j, knots = period_start, degree = 3)1    
#> bs(j, knots = period_start, degree = 3)2 ** 
#> bs(j, knots = period_start, degree = 3)3 ***
#> bs(j, knots = period_start, degree = 3)4 ***
#> bs(j, knots = period_start, degree = 3)5 ***
#> ---
#> Signif. codes:  0 '***' 0.001 '**' 0.01 '*' 0.05 '.' 0.1 ' ' 1
#> 
#> Residual standard error: 1.008 on 1217 degrees of freedom
#> Multiple R-squared:  0.5148, Adjusted R-squared:  0.5121 
#> F-statistic: 184.5 on 7 and 1217 DF,  p-value: < 2.2e-16
```

### 3.7.3 Linear trend, \(\lambda=5\)

```
period_start <- c(max(which(flair_data_lin_5$period==1)), max(which(flair_data_lin_5$period==2)))

mod_splines_per <- lm(response ~ as.factor(treatment) + bs(j, knots = period_start, degree = 3), flair_data_lin_5)
summary(mod_splines_per)
#> 
#> Call:
#> lm(formula = response ~ as.factor(treatment) + bs(j, knots = period_start, 
#>     degree = 3), data = flair_data_lin_5)
#> 
#> Residuals:
#>     Min      1Q  Median      3Q     Max 
#> -3.0522 -0.6708  0.0071  0.6573  3.2133 
#> 
#> Coefficients:
#>                                          Estimate Std. Error t value Pr(>|t|)
#> (Intercept)                              -0.01906    0.14676  -0.130  0.89670
#> as.factor(treatment)1                     0.80025    0.07096  11.277  < 2e-16
#> as.factor(treatment)2                     2.03626    0.08474  24.028  < 2e-16
#> bs(j, knots = period_start, degree = 3)1  0.91045    0.32444   2.806  0.00509
#> bs(j, knots = period_start, degree = 3)2  1.99601    0.19092  10.455  < 2e-16
#> bs(j, knots = period_start, degree = 3)3  3.53945    0.25548  13.854  < 2e-16
#> bs(j, knots = period_start, degree = 3)4  4.82978    0.24364  19.823  < 2e-16
#> bs(j, knots = period_start, degree = 3)5  5.03227    0.23568  21.352  < 2e-16
#>                                             
#> (Intercept)                                 
#> as.factor(treatment)1                    ***
#> as.factor(treatment)2                    ***
#> bs(j, knots = period_start, degree = 3)1 ** 
#> bs(j, knots = period_start, degree = 3)2 ***
#> bs(j, knots = period_start, degree = 3)3 ***
#> bs(j, knots = period_start, degree = 3)4 ***
#> bs(j, knots = period_start, degree = 3)5 ***
#> ---
#> Signif. codes:  0 '***' 0.001 '**' 0.01 '*' 0.05 '.' 0.1 ' ' 1
#> 
#> Residual standard error: 1.008 on 1217 degrees of freedom
#> Multiple R-squared:  0.7789, Adjusted R-squared:  0.7776 
#> F-statistic: 612.4 on 7 and 1217 DF,  p-value: < 2.2e-16
```

### 3.7.4 Stepwise trend, \(\lambda=0.25\)

```
period_start <- c(max(which(flair_data_step_0.25$period==1)), max(which(flair_data_step_0.25$period==2)))

mod_splines_per <- lm(response ~ as.factor(treatment) + bs(j, knots = period_start, degree = 3), flair_data_step_0.25)
summary(mod_splines_per)
#> 
#> Call:
#> lm(formula = response ~ as.factor(treatment) + bs(j, knots = period_start, 
#>     degree = 3), data = flair_data_step_0.25)
#> 
#> Residuals:
#>     Min      1Q  Median      3Q     Max 
#> -3.0457 -0.6700  0.0086  0.6588  3.2086 
#> 
#> Coefficients:
#>                                          Estimate Std. Error t value Pr(>|t|)
#> (Intercept)                              -0.01520    0.14685  -0.104   0.9176
#> as.factor(treatment)1                     0.80092    0.07100  11.280   <2e-16
#> as.factor(treatment)2                     2.04847    0.08479  24.158   <2e-16
#> bs(j, knots = period_start, degree = 3)1  0.05756    0.32463   0.177   0.8593
#> bs(j, knots = period_start, degree = 3)2 -0.11289    0.19103  -0.591   0.5547
#> bs(j, knots = period_start, degree = 3)3  0.28288    0.25563   1.107   0.2687
#> bs(j, knots = period_start, degree = 3)4  0.48273    0.24379   1.980   0.0479
#> bs(j, knots = period_start, degree = 3)5  0.31845    0.23582   1.350   0.1771
#>                                             
#> (Intercept)                                 
#> as.factor(treatment)1                    ***
#> as.factor(treatment)2                    ***
#> bs(j, knots = period_start, degree = 3)1    
#> bs(j, knots = period_start, degree = 3)2    
#> bs(j, knots = period_start, degree = 3)3    
#> bs(j, knots = period_start, degree = 3)4 *  
#> bs(j, knots = period_start, degree = 3)5    
#> ---
#> Signif. codes:  0 '***' 0.001 '**' 0.01 '*' 0.05 '.' 0.1 ' ' 1
#> 
#> Residual standard error: 1.009 on 1217 degrees of freedom
#> Multiple R-squared:  0.4172, Adjusted R-squared:  0.4138 
#> F-statistic: 124.4 on 7 and 1217 DF,  p-value: < 2.2e-16
```

### 3.7.5 Stepwise trend, \(\lambda=1.5\)

```
period_start <- c(max(which(flair_data_step_1.5$period==1)), max(which(flair_data_step_1.5$period==2)))

mod_splines_per <- lm(response ~ as.factor(treatment) + bs(j, knots = period_start, degree = 3), flair_data_step_1.5)
summary(mod_splines_per)
#> 
#> Call:
#> lm(formula = response ~ as.factor(treatment) + bs(j, knots = period_start, 
#>     degree = 3), data = flair_data_step_1.5)
#> 
#> Residuals:
#>     Min      1Q  Median      3Q     Max 
#> -3.0881 -0.6756 -0.0047  0.6781  3.6544 
#> 
#> Coefficients:
#>                                           Estimate Std. Error t value Pr(>|t|)
#> (Intercept)                               0.004066   0.150426   0.027 0.978439
#> as.factor(treatment)1                     0.804258   0.072736  11.057  < 2e-16
#> as.factor(treatment)2                     2.109498   0.086861  24.286  < 2e-16
#> bs(j, knots = period_start, degree = 3)1  0.092412   0.332550   0.278 0.781146
#> bs(j, knots = period_start, degree = 3)2 -0.650584   0.195690  -3.325 0.000912
#> bs(j, knots = period_start, degree = 3)3  2.333375   0.261865   8.911  < 2e-16
#> bs(j, knots = period_start, degree = 3)4  1.108036   0.249731   4.437 9.95e-06
#> bs(j, knots = period_start, degree = 3)5  1.728985   0.241569   7.157 1.42e-12
#>                                             
#> (Intercept)                                 
#> as.factor(treatment)1                    ***
#> as.factor(treatment)2                    ***
#> bs(j, knots = period_start, degree = 3)1    
#> bs(j, knots = period_start, degree = 3)2 ***
#> bs(j, knots = period_start, degree = 3)3 ***
#> bs(j, knots = period_start, degree = 3)4 ***
#> bs(j, knots = period_start, degree = 3)5 ***
#> ---
#> Signif. codes:  0 '***' 0.001 '**' 0.01 '*' 0.05 '.' 0.1 ' ' 1
#> 
#> Residual standard error: 1.033 on 1217 degrees of freedom
#> Multiple R-squared:  0.6027, Adjusted R-squared:  0.6004 
#> F-statistic: 263.8 on 7 and 1217 DF,  p-value: < 2.2e-16
```

### 3.7.6 Stepwise trend, \(\lambda=5\)

```
period_start <- c(max(which(flair_data_step_5$period==1)), max(which(flair_data_step_5$period==2)))

mod_splines_per <- lm(response ~ as.factor(treatment) + bs(j, knots = period_start, degree = 3), flair_data_step_5)
summary(mod_splines_per)
#> 
#> Call:
#> lm(formula = response ~ as.factor(treatment) + bs(j, knots = period_start, 
#>     degree = 3), data = flair_data_step_5)
#> 
#> Residuals:
#>     Min      1Q  Median      3Q     Max 
#> -4.4936 -0.8149 -0.0513  0.7629  4.9914 
#> 
#> Coefficients:
#>                                          Estimate Std. Error t value Pr(>|t|)
#> (Intercept)                               0.05802    0.18423   0.315    0.753
#> as.factor(treatment)1                     0.81360    0.08908   9.133   <2e-16
#> as.factor(treatment)2                     2.28038    0.10638  21.436   <2e-16
#> bs(j, knots = period_start, degree = 3)1  0.18999    0.40728   0.466    0.641
#> bs(j, knots = period_start, degree = 3)2 -2.15614    0.23966  -8.997   <2e-16
#> bs(j, knots = period_start, degree = 3)3  8.07475    0.32071  25.178   <2e-16
#> bs(j, knots = period_start, degree = 3)4  2.85888    0.30585   9.347   <2e-16
#> bs(j, knots = period_start, degree = 3)5  5.67847    0.29585  19.194   <2e-16
#>                                             
#> (Intercept)                                 
#> as.factor(treatment)1                    ***
#> as.factor(treatment)2                    ***
#> bs(j, knots = period_start, degree = 3)1    
#> bs(j, knots = period_start, degree = 3)2 ***
#> bs(j, knots = period_start, degree = 3)3 ***
#> bs(j, knots = period_start, degree = 3)4 ***
#> bs(j, knots = period_start, degree = 3)5 ***
#> ---
#> Signif. codes:  0 '***' 0.001 '**' 0.01 '*' 0.05 '.' 0.1 ' ' 1
#> 
#> Residual standard error: 1.266 on 1217 degrees of freedom
#> Multiple R-squared:  0.8315, Adjusted R-squared:  0.8305 
#> F-statistic: 857.8 on 7 and 1217 DF,  p-value: < 2.2e-16
```

## 3.8 Splines with knotes according to calendar time

### 3.8.1 Linear trend, \(\lambda=0.25\)

```
cal_time_start <- c()
for (i in unique(flair_data_lin_0.25$cal_time)) {
  cal_time_start <- c(cal_time_start, max(flair_data_lin_0.25[flair_data_lin_0.25$cal_time==i,]$j))
}

mod_splines_cal <- lm(response ~ as.factor(treatment) + bs(j, knots = cal_time_start[1:length(cal_time_start)-1], degree = 3), flair_data_lin_0.25)
summary(mod_splines_cal)
#> 
#> Call:
#> lm(formula = response ~ as.factor(treatment) + bs(j, knots = cal_time_start[1:length(cal_time_start) - 
#>     1], degree = 3), data = flair_data_lin_0.25)
#> 
#> Residuals:
#>     Min      1Q  Median      3Q     Max 
#> -3.0288 -0.6558  0.0094  0.6575  3.1216 
#> 
#> Coefficients:
#>                                                                           Estimate
#> (Intercept)                                                                0.41887
#> as.factor(treatment)1                                                      0.79326
#> as.factor(treatment)2                                                      2.03729
#> bs(j, knots = cal_time_start[1:length(cal_time_start) - 1], degree = 3)1  -0.44703
#> bs(j, knots = cal_time_start[1:length(cal_time_start) - 1], degree = 3)2  -0.79316
#> bs(j, knots = cal_time_start[1:length(cal_time_start) - 1], degree = 3)3  -0.19610
#> bs(j, knots = cal_time_start[1:length(cal_time_start) - 1], degree = 3)4  -0.23011
#> bs(j, knots = cal_time_start[1:length(cal_time_start) - 1], degree = 3)5  -0.45288
#> bs(j, knots = cal_time_start[1:length(cal_time_start) - 1], degree = 3)6  -0.28674
#> bs(j, knots = cal_time_start[1:length(cal_time_start) - 1], degree = 3)7  -0.43958
#> bs(j, knots = cal_time_start[1:length(cal_time_start) - 1], degree = 3)8  -0.41814
#> bs(j, knots = cal_time_start[1:length(cal_time_start) - 1], degree = 3)9  -0.08340
#> bs(j, knots = cal_time_start[1:length(cal_time_start) - 1], degree = 3)10 -0.57779
#> bs(j, knots = cal_time_start[1:length(cal_time_start) - 1], degree = 3)11  0.12105
#> bs(j, knots = cal_time_start[1:length(cal_time_start) - 1], degree = 3)12 -0.14126
#> bs(j, knots = cal_time_start[1:length(cal_time_start) - 1], degree = 3)13  0.06649
#> bs(j, knots = cal_time_start[1:length(cal_time_start) - 1], degree = 3)14 -0.36663
#> bs(j, knots = cal_time_start[1:length(cal_time_start) - 1], degree = 3)15  0.37141
#>                                                                           Std. Error
#> (Intercept)                                                                  0.32094
#> as.factor(treatment)1                                                        0.07119
#> as.factor(treatment)2                                                        0.08512
#> bs(j, knots = cal_time_start[1:length(cal_time_start) - 1], degree = 3)1     0.58488
#> bs(j, knots = cal_time_start[1:length(cal_time_start) - 1], degree = 3)2     0.37197
#> bs(j, knots = cal_time_start[1:length(cal_time_start) - 1], degree = 3)3     0.44155
#> bs(j, knots = cal_time_start[1:length(cal_time_start) - 1], degree = 3)4     0.37429
#> bs(j, knots = cal_time_start[1:length(cal_time_start) - 1], degree = 3)5     0.40131
#> bs(j, knots = cal_time_start[1:length(cal_time_start) - 1], degree = 3)6     0.38446
#> bs(j, knots = cal_time_start[1:length(cal_time_start) - 1], degree = 3)7     0.39294
#> bs(j, knots = cal_time_start[1:length(cal_time_start) - 1], degree = 3)8     0.39037
#> bs(j, knots = cal_time_start[1:length(cal_time_start) - 1], degree = 3)9     0.39239
#> bs(j, knots = cal_time_start[1:length(cal_time_start) - 1], degree = 3)10    0.39858
#> bs(j, knots = cal_time_start[1:length(cal_time_start) - 1], degree = 3)11    0.40472
#> bs(j, knots = cal_time_start[1:length(cal_time_start) - 1], degree = 3)12    0.44053
#> bs(j, knots = cal_time_start[1:length(cal_time_start) - 1], degree = 3)13    0.46705
#> bs(j, knots = cal_time_start[1:length(cal_time_start) - 1], degree = 3)14    0.46645
#> bs(j, knots = cal_time_start[1:length(cal_time_start) - 1], degree = 3)15    0.62378
#>                                                                           t value
#> (Intercept)                                                                 1.305
#> as.factor(treatment)1                                                      11.142
#> as.factor(treatment)2                                                      23.933
#> bs(j, knots = cal_time_start[1:length(cal_time_start) - 1], degree = 3)1   -0.764
#> bs(j, knots = cal_time_start[1:length(cal_time_start) - 1], degree = 3)2   -2.132
#> bs(j, knots = cal_time_start[1:length(cal_time_start) - 1], degree = 3)3   -0.444
#> bs(j, knots = cal_time_start[1:length(cal_time_start) - 1], degree = 3)4   -0.615
#> bs(j, knots = cal_time_start[1:length(cal_time_start) - 1], degree = 3)5   -1.129
#> bs(j, knots = cal_time_start[1:length(cal_time_start) - 1], degree = 3)6   -0.746
#> bs(j, knots = cal_time_start[1:length(cal_time_start) - 1], degree = 3)7   -1.119
#> bs(j, knots = cal_time_start[1:length(cal_time_start) - 1], degree = 3)8   -1.071
#> bs(j, knots = cal_time_start[1:length(cal_time_start) - 1], degree = 3)9   -0.213
#> bs(j, knots = cal_time_start[1:length(cal_time_start) - 1], degree = 3)10  -1.450
#> bs(j, knots = cal_time_start[1:length(cal_time_start) - 1], degree = 3)11   0.299
#> bs(j, knots = cal_time_start[1:length(cal_time_start) - 1], degree = 3)12  -0.321
#> bs(j, knots = cal_time_start[1:length(cal_time_start) - 1], degree = 3)13   0.142
#> bs(j, knots = cal_time_start[1:length(cal_time_start) - 1], degree = 3)14  -0.786
#> bs(j, knots = cal_time_start[1:length(cal_time_start) - 1], degree = 3)15   0.595
#>                                                                           Pr(>|t|)
#> (Intercept)                                                                 0.1921
#> as.factor(treatment)1                                                       <2e-16
#> as.factor(treatment)2                                                       <2e-16
#> bs(j, knots = cal_time_start[1:length(cal_time_start) - 1], degree = 3)1    0.4448
#> bs(j, knots = cal_time_start[1:length(cal_time_start) - 1], degree = 3)2    0.0332
#> bs(j, knots = cal_time_start[1:length(cal_time_start) - 1], degree = 3)3    0.6570
#> bs(j, knots = cal_time_start[1:length(cal_time_start) - 1], degree = 3)4    0.5388
#> bs(j, knots = cal_time_start[1:length(cal_time_start) - 1], degree = 3)5    0.2593
#> bs(j, knots = cal_time_start[1:length(cal_time_start) - 1], degree = 3)6    0.4559
#> bs(j, knots = cal_time_start[1:length(cal_time_start) - 1], degree = 3)7    0.2635
#> bs(j, knots = cal_time_start[1:length(cal_time_start) - 1], degree = 3)8    0.2843
#> bs(j, knots = cal_time_start[1:length(cal_time_start) - 1], degree = 3)9    0.8317
#> bs(j, knots = cal_time_start[1:length(cal_time_start) - 1], degree = 3)10   0.1474
#> bs(j, knots = cal_time_start[1:length(cal_time_start) - 1], degree = 3)11   0.7649
#> bs(j, knots = cal_time_start[1:length(cal_time_start) - 1], degree = 3)12   0.7485
#> bs(j, knots = cal_time_start[1:length(cal_time_start) - 1], degree = 3)13   0.8868
#> bs(j, knots = cal_time_start[1:length(cal_time_start) - 1], degree = 3)14   0.4320
#> bs(j, knots = cal_time_start[1:length(cal_time_start) - 1], degree = 3)15   0.5517
#>                                                                              
#> (Intercept)                                                                  
#> as.factor(treatment)1                                                     ***
#> as.factor(treatment)2                                                     ***
#> bs(j, knots = cal_time_start[1:length(cal_time_start) - 1], degree = 3)1     
#> bs(j, knots = cal_time_start[1:length(cal_time_start) - 1], degree = 3)2  *  
#> bs(j, knots = cal_time_start[1:length(cal_time_start) - 1], degree = 3)3     
#> bs(j, knots = cal_time_start[1:length(cal_time_start) - 1], degree = 3)4     
#> bs(j, knots = cal_time_start[1:length(cal_time_start) - 1], degree = 3)5     
#> bs(j, knots = cal_time_start[1:length(cal_time_start) - 1], degree = 3)6     
#> bs(j, knots = cal_time_start[1:length(cal_time_start) - 1], degree = 3)7     
#> bs(j, knots = cal_time_start[1:length(cal_time_start) - 1], degree = 3)8     
#> bs(j, knots = cal_time_start[1:length(cal_time_start) - 1], degree = 3)9     
#> bs(j, knots = cal_time_start[1:length(cal_time_start) - 1], degree = 3)10    
#> bs(j, knots = cal_time_start[1:length(cal_time_start) - 1], degree = 3)11    
#> bs(j, knots = cal_time_start[1:length(cal_time_start) - 1], degree = 3)12    
#> bs(j, knots = cal_time_start[1:length(cal_time_start) - 1], degree = 3)13    
#> bs(j, knots = cal_time_start[1:length(cal_time_start) - 1], degree = 3)14    
#> bs(j, knots = cal_time_start[1:length(cal_time_start) - 1], degree = 3)15    
#> ---
#> Signif. codes:  0 '***' 0.001 '**' 0.01 '*' 0.05 '.' 0.1 ' ' 1
#> 
#> Residual standard error: 1.008 on 1207 degrees of freedom
#> Multiple R-squared:  0.4072, Adjusted R-squared:  0.3988 
#> F-statistic: 48.77 on 17 and 1207 DF,  p-value: < 2.2e-16
```

### 3.8.2 Linear trend, \(\lambda=1.5\)

```
cal_time_start <- c()
for (i in unique(flair_data_lin_1.5$cal_time)) {
  cal_time_start <- c(cal_time_start, max(flair_data_lin_1.5[flair_data_lin_1.5$cal_time==i,]$j))
}

mod_splines_cal <- lm(response ~ as.factor(treatment) + bs(j, knots = cal_time_start[1:length(cal_time_start)-1], degree = 3), flair_data_lin_1.5)
summary(mod_splines_cal)
#> 
#> Call:
#> lm(formula = response ~ as.factor(treatment) + bs(j, knots = cal_time_start[1:length(cal_time_start) - 
#>     1], degree = 3), data = flair_data_lin_1.5)
#> 
#> Residuals:
#>     Min      1Q  Median      3Q     Max 
#> -3.0288 -0.6558  0.0094  0.6575  3.1216 
#> 
#> Coefficients:
#>                                                                            Estimate
#> (Intercept)                                                                0.418869
#> as.factor(treatment)1                                                      0.793262
#> as.factor(treatment)2                                                      2.037289
#> bs(j, knots = cal_time_start[1:length(cal_time_start) - 1], degree = 3)1  -0.413360
#> bs(j, knots = cal_time_start[1:length(cal_time_start) - 1], degree = 3)2  -0.691799
#> bs(j, knots = cal_time_start[1:length(cal_time_start) - 1], degree = 3)3   0.006961
#> bs(j, knots = cal_time_start[1:length(cal_time_start) - 1], degree = 3)4   0.074991
#> bs(j, knots = cal_time_start[1:length(cal_time_start) - 1], degree = 3)5  -0.045742
#> bs(j, knots = cal_time_start[1:length(cal_time_start) - 1], degree = 3)6   0.222440
#> bs(j, knots = cal_time_start[1:length(cal_time_start) - 1], degree = 3)7   0.171644
#> bs(j, knots = cal_time_start[1:length(cal_time_start) - 1], degree = 3)8   0.295130
#> bs(j, knots = cal_time_start[1:length(cal_time_start) - 1], degree = 3)9   0.731908
#> bs(j, knots = cal_time_start[1:length(cal_time_start) - 1], degree = 3)10  0.339556
#> bs(j, knots = cal_time_start[1:length(cal_time_start) - 1], degree = 3)11  1.140437
#> bs(j, knots = cal_time_start[1:length(cal_time_start) - 1], degree = 3)12  0.980168
#> bs(j, knots = cal_time_start[1:length(cal_time_start) - 1], degree = 3)13  1.264450
#> bs(j, knots = cal_time_start[1:length(cal_time_start) - 1], degree = 3)14  0.873846
#> bs(j, knots = cal_time_start[1:length(cal_time_start) - 1], degree = 3)15  1.620387
#>                                                                           Std. Error
#> (Intercept)                                                                 0.320935
#> as.factor(treatment)1                                                       0.071194
#> as.factor(treatment)2                                                       0.085124
#> bs(j, knots = cal_time_start[1:length(cal_time_start) - 1], degree = 3)1    0.584882
#> bs(j, knots = cal_time_start[1:length(cal_time_start) - 1], degree = 3)2    0.371966
#> bs(j, knots = cal_time_start[1:length(cal_time_start) - 1], degree = 3)3    0.441550
#> bs(j, knots = cal_time_start[1:length(cal_time_start) - 1], degree = 3)4    0.374291
#> bs(j, knots = cal_time_start[1:length(cal_time_start) - 1], degree = 3)5    0.401307
#> bs(j, knots = cal_time_start[1:length(cal_time_start) - 1], degree = 3)6    0.384458
#> bs(j, knots = cal_time_start[1:length(cal_time_start) - 1], degree = 3)7    0.392939
#> bs(j, knots = cal_time_start[1:length(cal_time_start) - 1], degree = 3)8    0.390369
#> bs(j, knots = cal_time_start[1:length(cal_time_start) - 1], degree = 3)9    0.392392
#> bs(j, knots = cal_time_start[1:length(cal_time_start) - 1], degree = 3)10   0.398583
#> bs(j, knots = cal_time_start[1:length(cal_time_start) - 1], degree = 3)11   0.404717
#> bs(j, knots = cal_time_start[1:length(cal_time_start) - 1], degree = 3)12   0.440526
#> bs(j, knots = cal_time_start[1:length(cal_time_start) - 1], degree = 3)13   0.467050
#> bs(j, knots = cal_time_start[1:length(cal_time_start) - 1], degree = 3)14   0.466453
#> bs(j, knots = cal_time_start[1:length(cal_time_start) - 1], degree = 3)15   0.623778
#>                                                                           t value
#> (Intercept)                                                                 1.305
#> as.factor(treatment)1                                                      11.142
#> as.factor(treatment)2                                                      23.933
#> bs(j, knots = cal_time_start[1:length(cal_time_start) - 1], degree = 3)1   -0.707
#> bs(j, knots = cal_time_start[1:length(cal_time_start) - 1], degree = 3)2   -1.860
#> bs(j, knots = cal_time_start[1:length(cal_time_start) - 1], degree = 3)3    0.016
#> bs(j, knots = cal_time_start[1:length(cal_time_start) - 1], degree = 3)4    0.200
#> bs(j, knots = cal_time_start[1:length(cal_time_start) - 1], degree = 3)5   -0.114
#> bs(j, knots = cal_time_start[1:length(cal_time_start) - 1], degree = 3)6    0.579
#> bs(j, knots = cal_time_start[1:length(cal_time_start) - 1], degree = 3)7    0.437
#> bs(j, knots = cal_time_start[1:length(cal_time_start) - 1], degree = 3)8    0.756
#> bs(j, knots = cal_time_start[1:length(cal_time_start) - 1], degree = 3)9    1.865
#> bs(j, knots = cal_time_start[1:length(cal_time_start) - 1], degree = 3)10   0.852
#> bs(j, knots = cal_time_start[1:length(cal_time_start) - 1], degree = 3)11   2.818
#> bs(j, knots = cal_time_start[1:length(cal_time_start) - 1], degree = 3)12   2.225
#> bs(j, knots = cal_time_start[1:length(cal_time_start) - 1], degree = 3)13   2.707
#> bs(j, knots = cal_time_start[1:length(cal_time_start) - 1], degree = 3)14   1.873
#> bs(j, knots = cal_time_start[1:length(cal_time_start) - 1], degree = 3)15   2.598
#>                                                                           Pr(>|t|)
#> (Intercept)                                                                0.19209
#> as.factor(treatment)1                                                      < 2e-16
#> as.factor(treatment)2                                                      < 2e-16
#> bs(j, knots = cal_time_start[1:length(cal_time_start) - 1], degree = 3)1   0.47986
#> bs(j, knots = cal_time_start[1:length(cal_time_start) - 1], degree = 3)2   0.06315
#> bs(j, knots = cal_time_start[1:length(cal_time_start) - 1], degree = 3)3   0.98742
#> bs(j, knots = cal_time_start[1:length(cal_time_start) - 1], degree = 3)4   0.84124
#> bs(j, knots = cal_time_start[1:length(cal_time_start) - 1], degree = 3)5   0.90927
#> bs(j, knots = cal_time_start[1:length(cal_time_start) - 1], degree = 3)6   0.56298
#> bs(j, knots = cal_time_start[1:length(cal_time_start) - 1], degree = 3)7   0.66232
#> bs(j, knots = cal_time_start[1:length(cal_time_start) - 1], degree = 3)8   0.44978
#> bs(j, knots = cal_time_start[1:length(cal_time_start) - 1], degree = 3)9   0.06239
#> bs(j, knots = cal_time_start[1:length(cal_time_start) - 1], degree = 3)10  0.39443
#> bs(j, knots = cal_time_start[1:length(cal_time_start) - 1], degree = 3)11  0.00491
#> bs(j, knots = cal_time_start[1:length(cal_time_start) - 1], degree = 3)12  0.02627
#> bs(j, knots = cal_time_start[1:length(cal_time_start) - 1], degree = 3)13  0.00688
#> bs(j, knots = cal_time_start[1:length(cal_time_start) - 1], degree = 3)14  0.06126
#> bs(j, knots = cal_time_start[1:length(cal_time_start) - 1], degree = 3)15  0.00950
#>                                                                              
#> (Intercept)                                                                  
#> as.factor(treatment)1                                                     ***
#> as.factor(treatment)2                                                     ***
#> bs(j, knots = cal_time_start[1:length(cal_time_start) - 1], degree = 3)1     
#> bs(j, knots = cal_time_start[1:length(cal_time_start) - 1], degree = 3)2  .  
#> bs(j, knots = cal_time_start[1:length(cal_time_start) - 1], degree = 3)3     
#> bs(j, knots = cal_time_start[1:length(cal_time_start) - 1], degree = 3)4     
#> bs(j, knots = cal_time_start[1:length(cal_time_start) - 1], degree = 3)5     
#> bs(j, knots = cal_time_start[1:length(cal_time_start) - 1], degree = 3)6     
#> bs(j, knots = cal_time_start[1:length(cal_time_start) - 1], degree = 3)7     
#> bs(j, knots = cal_time_start[1:length(cal_time_start) - 1], degree = 3)8     
#> bs(j, knots = cal_time_start[1:length(cal_time_start) - 1], degree = 3)9  .  
#> bs(j, knots = cal_time_start[1:length(cal_time_start) - 1], degree = 3)10    
#> bs(j, knots = cal_time_start[1:length(cal_time_start) - 1], degree = 3)11 ** 
#> bs(j, knots = cal_time_start[1:length(cal_time_start) - 1], degree = 3)12 *  
#> bs(j, knots = cal_time_start[1:length(cal_time_start) - 1], degree = 3)13 ** 
#> bs(j, knots = cal_time_start[1:length(cal_time_start) - 1], degree = 3)14 .  
#> bs(j, knots = cal_time_start[1:length(cal_time_start) - 1], degree = 3)15 ** 
#> ---
#> Signif. codes:  0 '***' 0.001 '**' 0.01 '*' 0.05 '.' 0.1 ' ' 1
#> 
#> Residual standard error: 1.008 on 1207 degrees of freedom
#> Multiple R-squared:  0.5191, Adjusted R-squared:  0.5123 
#> F-statistic: 76.65 on 17 and 1207 DF,  p-value: < 2.2e-16
```

### 3.8.3 Linear trend, \(\lambda=5\)

```
cal_time_start <- c()
for (i in unique(flair_data_lin_5$cal_time)) {
  cal_time_start <- c(cal_time_start, max(flair_data_lin_5[flair_data_lin_5$cal_time==i,]$j))
}

mod_splines_cal <- lm(response ~ as.factor(treatment) + bs(j, knots = cal_time_start[1:length(cal_time_start)-1], degree = 3), flair_data_lin_5)
summary(mod_splines_cal)
#> 
#> Call:
#> lm(formula = response ~ as.factor(treatment) + bs(j, knots = cal_time_start[1:length(cal_time_start) - 
#>     1], degree = 3), data = flair_data_lin_5)
#> 
#> Residuals:
#>     Min      1Q  Median      3Q     Max 
#> -3.0288 -0.6558  0.0094  0.6575  3.1216 
#> 
#> Coefficients:
#>                                                                           Estimate
#> (Intercept)                                                                0.41887
#> as.factor(treatment)1                                                      0.79326
#> as.factor(treatment)2                                                      2.03729
#> bs(j, knots = cal_time_start[1:length(cal_time_start) - 1], degree = 3)1  -0.31907
#> bs(j, knots = cal_time_start[1:length(cal_time_start) - 1], degree = 3)2  -0.40799
#> bs(j, knots = cal_time_start[1:length(cal_time_start) - 1], degree = 3)3   0.57553
#> bs(j, knots = cal_time_start[1:length(cal_time_start) - 1], degree = 3)4   0.92928
#> bs(j, knots = cal_time_start[1:length(cal_time_start) - 1], degree = 3)5   1.09426
#> bs(j, knots = cal_time_start[1:length(cal_time_start) - 1], degree = 3)6   1.64815
#> bs(j, knots = cal_time_start[1:length(cal_time_start) - 1], degree = 3)7   1.88307
#> bs(j, knots = cal_time_start[1:length(cal_time_start) - 1], degree = 3)8   2.29227
#> bs(j, knots = cal_time_start[1:length(cal_time_start) - 1], degree = 3)9   3.01477
#> bs(j, knots = cal_time_start[1:length(cal_time_start) - 1], degree = 3)10  2.90813
#> bs(j, knots = cal_time_start[1:length(cal_time_start) - 1], degree = 3)11  3.99472
#> bs(j, knots = cal_time_start[1:length(cal_time_start) - 1], degree = 3)12  4.12017
#> bs(j, knots = cal_time_start[1:length(cal_time_start) - 1], degree = 3)13  4.61874
#> bs(j, knots = cal_time_start[1:length(cal_time_start) - 1], degree = 3)14  4.34718
#> bs(j, knots = cal_time_start[1:length(cal_time_start) - 1], degree = 3)15  5.11753
#>                                                                           Std. Error
#> (Intercept)                                                                  0.32094
#> as.factor(treatment)1                                                        0.07119
#> as.factor(treatment)2                                                        0.08512
#> bs(j, knots = cal_time_start[1:length(cal_time_start) - 1], degree = 3)1     0.58488
#> bs(j, knots = cal_time_start[1:length(cal_time_start) - 1], degree = 3)2     0.37197
#> bs(j, knots = cal_time_start[1:length(cal_time_start) - 1], degree = 3)3     0.44155
#> bs(j, knots = cal_time_start[1:length(cal_time_start) - 1], degree = 3)4     0.37429
#> bs(j, knots = cal_time_start[1:length(cal_time_start) - 1], degree = 3)5     0.40131
#> bs(j, knots = cal_time_start[1:length(cal_time_start) - 1], degree = 3)6     0.38446
#> bs(j, knots = cal_time_start[1:length(cal_time_start) - 1], degree = 3)7     0.39294
#> bs(j, knots = cal_time_start[1:length(cal_time_start) - 1], degree = 3)8     0.39037
#> bs(j, knots = cal_time_start[1:length(cal_time_start) - 1], degree = 3)9     0.39239
#> bs(j, knots = cal_time_start[1:length(cal_time_start) - 1], degree = 3)10    0.39858
#> bs(j, knots = cal_time_start[1:length(cal_time_start) - 1], degree = 3)11    0.40472
#> bs(j, knots = cal_time_start[1:length(cal_time_start) - 1], degree = 3)12    0.44053
#> bs(j, knots = cal_time_start[1:length(cal_time_start) - 1], degree = 3)13    0.46705
#> bs(j, knots = cal_time_start[1:length(cal_time_start) - 1], degree = 3)14    0.46645
#> bs(j, knots = cal_time_start[1:length(cal_time_start) - 1], degree = 3)15    0.62378
#>                                                                           t value
#> (Intercept)                                                                 1.305
#> as.factor(treatment)1                                                      11.142
#> as.factor(treatment)2                                                      23.933
#> bs(j, knots = cal_time_start[1:length(cal_time_start) - 1], degree = 3)1   -0.546
#> bs(j, knots = cal_time_start[1:length(cal_time_start) - 1], degree = 3)2   -1.097
#> bs(j, knots = cal_time_start[1:length(cal_time_start) - 1], degree = 3)3    1.303
#> bs(j, knots = cal_time_start[1:length(cal_time_start) - 1], degree = 3)4    2.483
#> bs(j, knots = cal_time_start[1:length(cal_time_start) - 1], degree = 3)5    2.727
#> bs(j, knots = cal_time_start[1:length(cal_time_start) - 1], degree = 3)6    4.287
#> bs(j, knots = cal_time_start[1:length(cal_time_start) - 1], degree = 3)7    4.792
#> bs(j, knots = cal_time_start[1:length(cal_time_start) - 1], degree = 3)8    5.872
#> bs(j, knots = cal_time_start[1:length(cal_time_start) - 1], degree = 3)9    7.683
#> bs(j, knots = cal_time_start[1:length(cal_time_start) - 1], degree = 3)10   7.296
#> bs(j, knots = cal_time_start[1:length(cal_time_start) - 1], degree = 3)11   9.870
#> bs(j, knots = cal_time_start[1:length(cal_time_start) - 1], degree = 3)12   9.353
#> bs(j, knots = cal_time_start[1:length(cal_time_start) - 1], degree = 3)13   9.889
#> bs(j, knots = cal_time_start[1:length(cal_time_start) - 1], degree = 3)14   9.320
#> bs(j, knots = cal_time_start[1:length(cal_time_start) - 1], degree = 3)15   8.204
#>                                                                           Pr(>|t|)
#> (Intercept)                                                                0.19209
#> as.factor(treatment)1                                                      < 2e-16
#> as.factor(treatment)2                                                      < 2e-16
#> bs(j, knots = cal_time_start[1:length(cal_time_start) - 1], degree = 3)1   0.58549
#> bs(j, knots = cal_time_start[1:length(cal_time_start) - 1], degree = 3)2   0.27293
#> bs(j, knots = cal_time_start[1:length(cal_time_start) - 1], degree = 3)3   0.19267
#> bs(j, knots = cal_time_start[1:length(cal_time_start) - 1], degree = 3)4   0.01317
#> bs(j, knots = cal_time_start[1:length(cal_time_start) - 1], degree = 3)5   0.00649
#> bs(j, knots = cal_time_start[1:length(cal_time_start) - 1], degree = 3)6  1.96e-05
#> bs(j, knots = cal_time_start[1:length(cal_time_start) - 1], degree = 3)7  1.85e-06
#> bs(j, knots = cal_time_start[1:length(cal_time_start) - 1], degree = 3)8  5.56e-09
#> bs(j, knots = cal_time_start[1:length(cal_time_start) - 1], degree = 3)9  3.20e-14
#> bs(j, knots = cal_time_start[1:length(cal_time_start) - 1], degree = 3)10 5.35e-13
#> bs(j, knots = cal_time_start[1:length(cal_time_start) - 1], degree = 3)11  < 2e-16
#> bs(j, knots = cal_time_start[1:length(cal_time_start) - 1], degree = 3)12  < 2e-16
#> bs(j, knots = cal_time_start[1:length(cal_time_start) - 1], degree = 3)13  < 2e-16
#> bs(j, knots = cal_time_start[1:length(cal_time_start) - 1], degree = 3)14  < 2e-16
#> bs(j, knots = cal_time_start[1:length(cal_time_start) - 1], degree = 3)15 5.90e-16
#>                                                                              
#> (Intercept)                                                                  
#> as.factor(treatment)1                                                     ***
#> as.factor(treatment)2                                                     ***
#> bs(j, knots = cal_time_start[1:length(cal_time_start) - 1], degree = 3)1     
#> bs(j, knots = cal_time_start[1:length(cal_time_start) - 1], degree = 3)2     
#> bs(j, knots = cal_time_start[1:length(cal_time_start) - 1], degree = 3)3     
#> bs(j, knots = cal_time_start[1:length(cal_time_start) - 1], degree = 3)4  *  
#> bs(j, knots = cal_time_start[1:length(cal_time_start) - 1], degree = 3)5  ** 
#> bs(j, knots = cal_time_start[1:length(cal_time_start) - 1], degree = 3)6  ***
#> bs(j, knots = cal_time_start[1:length(cal_time_start) - 1], degree = 3)7  ***
#> bs(j, knots = cal_time_start[1:length(cal_time_start) - 1], degree = 3)8  ***
#> bs(j, knots = cal_time_start[1:length(cal_time_start) - 1], degree = 3)9  ***
#> bs(j, knots = cal_time_start[1:length(cal_time_start) - 1], degree = 3)10 ***
#> bs(j, knots = cal_time_start[1:length(cal_time_start) - 1], degree = 3)11 ***
#> bs(j, knots = cal_time_start[1:length(cal_time_start) - 1], degree = 3)12 ***
#> bs(j, knots = cal_time_start[1:length(cal_time_start) - 1], degree = 3)13 ***
#> bs(j, knots = cal_time_start[1:length(cal_time_start) - 1], degree = 3)14 ***
#> bs(j, knots = cal_time_start[1:length(cal_time_start) - 1], degree = 3)15 ***
#> ---
#> Signif. codes:  0 '***' 0.001 '**' 0.01 '*' 0.05 '.' 0.1 ' ' 1
#> 
#> Residual standard error: 1.008 on 1207 degrees of freedom
#> Multiple R-squared:  0.7808, Adjusted R-squared:  0.7778 
#> F-statistic:   253 on 17 and 1207 DF,  p-value: < 2.2e-16
```

### 3.8.4 Stepwise trend, \(\lambda=0.25\)

```
cal_time_start <- c()
for (i in unique(flair_data_step_0.25$cal_time)) {
  cal_time_start <- c(cal_time_start, max(flair_data_step_0.25[flair_data_step_0.25$cal_time==i,]$j))
}

mod_splines_cal <- lm(response ~ as.factor(treatment) + bs(j, knots = cal_time_start[1:length(cal_time_start)-1], degree = 3), flair_data_step_0.25)
summary(mod_splines_cal)
#> 
#> Call:
#> lm(formula = response ~ as.factor(treatment) + bs(j, knots = cal_time_start[1:length(cal_time_start) - 
#>     1], degree = 3), data = flair_data_step_0.25)
#> 
#> Residuals:
#>      Min       1Q   Median       3Q      Max 
#> -3.02884 -0.65636  0.00689  0.65787  3.14651 
#> 
#> Coefficients:
#>                                                                           Estimate
#> (Intercept)                                                                0.42001
#> as.factor(treatment)1                                                      0.79156
#> as.factor(treatment)2                                                      2.04181
#> bs(j, knots = cal_time_start[1:length(cal_time_start) - 1], degree = 3)1  -0.45472
#> bs(j, knots = cal_time_start[1:length(cal_time_start) - 1], degree = 3)2  -0.81291
#> bs(j, knots = cal_time_start[1:length(cal_time_start) - 1], degree = 3)3  -0.23801
#> bs(j, knots = cal_time_start[1:length(cal_time_start) - 1], degree = 3)4  -0.28980
#> bs(j, knots = cal_time_start[1:length(cal_time_start) - 1], degree = 3)5  -0.53798
#> bs(j, knots = cal_time_start[1:length(cal_time_start) - 1], degree = 3)6  -0.37920
#> bs(j, knots = cal_time_start[1:length(cal_time_start) - 1], degree = 3)7  -0.60658
#> bs(j, knots = cal_time_start[1:length(cal_time_start) - 1], degree = 3)8  -0.19746
#> bs(j, knots = cal_time_start[1:length(cal_time_start) - 1], degree = 3)9  -0.05516
#> bs(j, knots = cal_time_start[1:length(cal_time_start) - 1], degree = 3)10 -0.48481
#> bs(j, knots = cal_time_start[1:length(cal_time_start) - 1], degree = 3)11  0.14630
#> bs(j, knots = cal_time_start[1:length(cal_time_start) - 1], degree = 3)12 -0.10611
#> bs(j, knots = cal_time_start[1:length(cal_time_start) - 1], degree = 3)13  0.06487
#> bs(j, knots = cal_time_start[1:length(cal_time_start) - 1], degree = 3)14 -0.36351
#> bs(j, knots = cal_time_start[1:length(cal_time_start) - 1], degree = 3)15  0.36669
#>                                                                           Std. Error
#> (Intercept)                                                                  0.32084
#> as.factor(treatment)1                                                        0.07117
#> as.factor(treatment)2                                                        0.08510
#> bs(j, knots = cal_time_start[1:length(cal_time_start) - 1], degree = 3)1     0.58470
#> bs(j, knots = cal_time_start[1:length(cal_time_start) - 1], degree = 3)2     0.37185
#> bs(j, knots = cal_time_start[1:length(cal_time_start) - 1], degree = 3)3     0.44141
#> bs(j, knots = cal_time_start[1:length(cal_time_start) - 1], degree = 3)4     0.37418
#> bs(j, knots = cal_time_start[1:length(cal_time_start) - 1], degree = 3)5     0.40118
#> bs(j, knots = cal_time_start[1:length(cal_time_start) - 1], degree = 3)6     0.38434
#> bs(j, knots = cal_time_start[1:length(cal_time_start) - 1], degree = 3)7     0.39282
#> bs(j, knots = cal_time_start[1:length(cal_time_start) - 1], degree = 3)8     0.39025
#> bs(j, knots = cal_time_start[1:length(cal_time_start) - 1], degree = 3)9     0.39227
#> bs(j, knots = cal_time_start[1:length(cal_time_start) - 1], degree = 3)10    0.39846
#> bs(j, knots = cal_time_start[1:length(cal_time_start) - 1], degree = 3)11    0.40459
#> bs(j, knots = cal_time_start[1:length(cal_time_start) - 1], degree = 3)12    0.44039
#> bs(j, knots = cal_time_start[1:length(cal_time_start) - 1], degree = 3)13    0.46691
#> bs(j, knots = cal_time_start[1:length(cal_time_start) - 1], degree = 3)14    0.46631
#> bs(j, knots = cal_time_start[1:length(cal_time_start) - 1], degree = 3)15    0.62359
#>                                                                           t value
#> (Intercept)                                                                 1.309
#> as.factor(treatment)1                                                      11.122
#> as.factor(treatment)2                                                      23.994
#> bs(j, knots = cal_time_start[1:length(cal_time_start) - 1], degree = 3)1   -0.778
#> bs(j, knots = cal_time_start[1:length(cal_time_start) - 1], degree = 3)2   -2.186
#> bs(j, knots = cal_time_start[1:length(cal_time_start) - 1], degree = 3)3   -0.539
#> bs(j, knots = cal_time_start[1:length(cal_time_start) - 1], degree = 3)4   -0.775
#> bs(j, knots = cal_time_start[1:length(cal_time_start) - 1], degree = 3)5   -1.341
#> bs(j, knots = cal_time_start[1:length(cal_time_start) - 1], degree = 3)6   -0.987
#> bs(j, knots = cal_time_start[1:length(cal_time_start) - 1], degree = 3)7   -1.544
#> bs(j, knots = cal_time_start[1:length(cal_time_start) - 1], degree = 3)8   -0.506
#> bs(j, knots = cal_time_start[1:length(cal_time_start) - 1], degree = 3)9   -0.141
#> bs(j, knots = cal_time_start[1:length(cal_time_start) - 1], degree = 3)10  -1.217
#> bs(j, knots = cal_time_start[1:length(cal_time_start) - 1], degree = 3)11   0.362
#> bs(j, knots = cal_time_start[1:length(cal_time_start) - 1], degree = 3)12  -0.241
#> bs(j, knots = cal_time_start[1:length(cal_time_start) - 1], degree = 3)13   0.139
#> bs(j, knots = cal_time_start[1:length(cal_time_start) - 1], degree = 3)14  -0.780
#> bs(j, knots = cal_time_start[1:length(cal_time_start) - 1], degree = 3)15   0.588
#>                                                                           Pr(>|t|)
#> (Intercept)                                                                  0.191
#> as.factor(treatment)1                                                       <2e-16
#> as.factor(treatment)2                                                       <2e-16
#> bs(j, knots = cal_time_start[1:length(cal_time_start) - 1], degree = 3)1     0.437
#> bs(j, knots = cal_time_start[1:length(cal_time_start) - 1], degree = 3)2     0.029
#> bs(j, knots = cal_time_start[1:length(cal_time_start) - 1], degree = 3)3     0.590
#> bs(j, knots = cal_time_start[1:length(cal_time_start) - 1], degree = 3)4     0.439
#> bs(j, knots = cal_time_start[1:length(cal_time_start) - 1], degree = 3)5     0.180
#> bs(j, knots = cal_time_start[1:length(cal_time_start) - 1], degree = 3)6     0.324
#> bs(j, knots = cal_time_start[1:length(cal_time_start) - 1], degree = 3)7     0.123
#> bs(j, knots = cal_time_start[1:length(cal_time_start) - 1], degree = 3)8     0.613
#> bs(j, knots = cal_time_start[1:length(cal_time_start) - 1], degree = 3)9     0.888
#> bs(j, knots = cal_time_start[1:length(cal_time_start) - 1], degree = 3)10    0.224
#> bs(j, knots = cal_time_start[1:length(cal_time_start) - 1], degree = 3)11    0.718
#> bs(j, knots = cal_time_start[1:length(cal_time_start) - 1], degree = 3)12    0.810
#> bs(j, knots = cal_time_start[1:length(cal_time_start) - 1], degree = 3)13    0.890
#> bs(j, knots = cal_time_start[1:length(cal_time_start) - 1], degree = 3)14    0.436
#> bs(j, knots = cal_time_start[1:length(cal_time_start) - 1], degree = 3)15    0.557
#>                                                                              
#> (Intercept)                                                                  
#> as.factor(treatment)1                                                     ***
#> as.factor(treatment)2                                                     ***
#> bs(j, knots = cal_time_start[1:length(cal_time_start) - 1], degree = 3)1     
#> bs(j, knots = cal_time_start[1:length(cal_time_start) - 1], degree = 3)2  *  
#> bs(j, knots = cal_time_start[1:length(cal_time_start) - 1], degree = 3)3     
#> bs(j, knots = cal_time_start[1:length(cal_time_start) - 1], degree = 3)4     
#> bs(j, knots = cal_time_start[1:length(cal_time_start) - 1], degree = 3)5     
#> bs(j, knots = cal_time_start[1:length(cal_time_start) - 1], degree = 3)6     
#> bs(j, knots = cal_time_start[1:length(cal_time_start) - 1], degree = 3)7     
#> bs(j, knots = cal_time_start[1:length(cal_time_start) - 1], degree = 3)8     
#> bs(j, knots = cal_time_start[1:length(cal_time_start) - 1], degree = 3)9     
#> bs(j, knots = cal_time_start[1:length(cal_time_start) - 1], degree = 3)10    
#> bs(j, knots = cal_time_start[1:length(cal_time_start) - 1], degree = 3)11    
#> bs(j, knots = cal_time_start[1:length(cal_time_start) - 1], degree = 3)12    
#> bs(j, knots = cal_time_start[1:length(cal_time_start) - 1], degree = 3)13    
#> bs(j, knots = cal_time_start[1:length(cal_time_start) - 1], degree = 3)14    
#> bs(j, knots = cal_time_start[1:length(cal_time_start) - 1], degree = 3)15    
#> ---
#> Signif. codes:  0 '***' 0.001 '**' 0.01 '*' 0.05 '.' 0.1 ' ' 1
#> 
#> Residual standard error: 1.008 on 1207 degrees of freedom
#> Multiple R-squared:  0.4233, Adjusted R-squared:  0.4152 
#> F-statistic: 52.12 on 17 and 1207 DF,  p-value: < 2.2e-16
```

### 3.8.5 Stepwise trend, \(\lambda=1.5\)

```
cal_time_start <- c()
for (i in unique(flair_data_step_1.5$cal_time)) {
  cal_time_start <- c(cal_time_start, max(flair_data_step_1.5[flair_data_step_1.5$cal_time==i,]$j))
}

mod_splines_cal <- lm(response ~ as.factor(treatment) + bs(j, knots = cal_time_start[1:length(cal_time_start)-1], degree = 3), flair_data_step_1.5)
summary(mod_splines_cal)
#> 
#> Call:
#> lm(formula = response ~ as.factor(treatment) + bs(j, knots = cal_time_start[1:length(cal_time_start) - 
#>     1], degree = 3), data = flair_data_step_1.5)
#> 
#> Residuals:
#>     Min      1Q  Median      3Q     Max 
#> -3.0289 -0.6477  0.0046  0.6635  3.2712 
#> 
#> Coefficients:
#>                                                                           Estimate
#> (Intercept)                                                                0.42573
#> as.factor(treatment)1                                                      0.78305
#> as.factor(treatment)2                                                      2.06441
#> bs(j, knots = cal_time_start[1:length(cal_time_start) - 1], degree = 3)1  -0.45949
#> bs(j, knots = cal_time_start[1:length(cal_time_start) - 1], degree = 3)2  -0.81029
#> bs(j, knots = cal_time_start[1:length(cal_time_start) - 1], degree = 3)3  -0.24451
#> bs(j, knots = cal_time_start[1:length(cal_time_start) - 1], degree = 3)4  -0.28316
#> bs(j, knots = cal_time_start[1:length(cal_time_start) - 1], degree = 3)5  -0.55632
#> bs(j, knots = cal_time_start[1:length(cal_time_start) - 1], degree = 3)6  -0.33229
#> bs(j, knots = cal_time_start[1:length(cal_time_start) - 1], degree = 3)7  -0.83034
#> bs(j, knots = cal_time_start[1:length(cal_time_start) - 1], degree = 3)8   1.61916
#> bs(j, knots = cal_time_start[1:length(cal_time_start) - 1], degree = 3)9   0.90131
#> bs(j, knots = cal_time_start[1:length(cal_time_start) - 1], degree = 3)10  0.89743
#> bs(j, knots = cal_time_start[1:length(cal_time_start) - 1], degree = 3)11  1.29193
#> bs(j, knots = cal_time_start[1:length(cal_time_start) - 1], degree = 3)12  1.19109
#> bs(j, knots = cal_time_start[1:length(cal_time_start) - 1], degree = 3)13  1.25474
#> bs(j, knots = cal_time_start[1:length(cal_time_start) - 1], degree = 3)14  0.89258
#> bs(j, knots = cal_time_start[1:length(cal_time_start) - 1], degree = 3)15  1.59207
#>                                                                           Std. Error
#> (Intercept)                                                                  0.32259
#> as.factor(treatment)1                                                        0.07156
#> as.factor(treatment)2                                                        0.08556
#> bs(j, knots = cal_time_start[1:length(cal_time_start) - 1], degree = 3)1     0.58790
#> bs(j, knots = cal_time_start[1:length(cal_time_start) - 1], degree = 3)2     0.37389
#> bs(j, knots = cal_time_start[1:length(cal_time_start) - 1], degree = 3)3     0.44383
#> bs(j, knots = cal_time_start[1:length(cal_time_start) - 1], degree = 3)4     0.37622
#> bs(j, knots = cal_time_start[1:length(cal_time_start) - 1], degree = 3)5     0.40338
#> bs(j, knots = cal_time_start[1:length(cal_time_start) - 1], degree = 3)6     0.38644
#> bs(j, knots = cal_time_start[1:length(cal_time_start) - 1], degree = 3)7     0.39497
#> bs(j, knots = cal_time_start[1:length(cal_time_start) - 1], degree = 3)8     0.39238
#> bs(j, knots = cal_time_start[1:length(cal_time_start) - 1], degree = 3)9     0.39442
#> bs(j, knots = cal_time_start[1:length(cal_time_start) - 1], degree = 3)10    0.40064
#> bs(j, knots = cal_time_start[1:length(cal_time_start) - 1], degree = 3)11    0.40681
#> bs(j, knots = cal_time_start[1:length(cal_time_start) - 1], degree = 3)12    0.44280
#> bs(j, knots = cal_time_start[1:length(cal_time_start) - 1], degree = 3)13    0.46946
#> bs(j, knots = cal_time_start[1:length(cal_time_start) - 1], degree = 3)14    0.46886
#> bs(j, knots = cal_time_start[1:length(cal_time_start) - 1], degree = 3)15    0.62700
#>                                                                           t value
#> (Intercept)                                                                 1.320
#> as.factor(treatment)1                                                      10.942
#> as.factor(treatment)2                                                      24.127
#> bs(j, knots = cal_time_start[1:length(cal_time_start) - 1], degree = 3)1   -0.782
#> bs(j, knots = cal_time_start[1:length(cal_time_start) - 1], degree = 3)2   -2.167
#> bs(j, knots = cal_time_start[1:length(cal_time_start) - 1], degree = 3)3   -0.551
#> bs(j, knots = cal_time_start[1:length(cal_time_start) - 1], degree = 3)4   -0.753
#> bs(j, knots = cal_time_start[1:length(cal_time_start) - 1], degree = 3)5   -1.379
#> bs(j, knots = cal_time_start[1:length(cal_time_start) - 1], degree = 3)6   -0.860
#> bs(j, knots = cal_time_start[1:length(cal_time_start) - 1], degree = 3)7   -2.102
#> bs(j, knots = cal_time_start[1:length(cal_time_start) - 1], degree = 3)8    4.126
#> bs(j, knots = cal_time_start[1:length(cal_time_start) - 1], degree = 3)9    2.285
#> bs(j, knots = cal_time_start[1:length(cal_time_start) - 1], degree = 3)10   2.240
#> bs(j, knots = cal_time_start[1:length(cal_time_start) - 1], degree = 3)11   3.176
#> bs(j, knots = cal_time_start[1:length(cal_time_start) - 1], degree = 3)12   2.690
#> bs(j, knots = cal_time_start[1:length(cal_time_start) - 1], degree = 3)13   2.673
#> bs(j, knots = cal_time_start[1:length(cal_time_start) - 1], degree = 3)14   1.904
#> bs(j, knots = cal_time_start[1:length(cal_time_start) - 1], degree = 3)15   2.539
#>                                                                           Pr(>|t|)
#> (Intercept)                                                                0.18718
#> as.factor(treatment)1                                                      < 2e-16
#> as.factor(treatment)2                                                      < 2e-16
#> bs(j, knots = cal_time_start[1:length(cal_time_start) - 1], degree = 3)1   0.43461
#> bs(j, knots = cal_time_start[1:length(cal_time_start) - 1], degree = 3)2   0.03041
#> bs(j, knots = cal_time_start[1:length(cal_time_start) - 1], degree = 3)3   0.58180
#> bs(j, knots = cal_time_start[1:length(cal_time_start) - 1], degree = 3)4   0.45181
#> bs(j, knots = cal_time_start[1:length(cal_time_start) - 1], degree = 3)5   0.16811
#> bs(j, knots = cal_time_start[1:length(cal_time_start) - 1], degree = 3)6   0.39003
#> bs(j, knots = cal_time_start[1:length(cal_time_start) - 1], degree = 3)7   0.03573
#> bs(j, knots = cal_time_start[1:length(cal_time_start) - 1], degree = 3)8  3.94e-05
#> bs(j, knots = cal_time_start[1:length(cal_time_start) - 1], degree = 3)9   0.02248
#> bs(j, knots = cal_time_start[1:length(cal_time_start) - 1], degree = 3)10  0.02527
#> bs(j, knots = cal_time_start[1:length(cal_time_start) - 1], degree = 3)11  0.00153
#> bs(j, knots = cal_time_start[1:length(cal_time_start) - 1], degree = 3)12  0.00725
#> bs(j, knots = cal_time_start[1:length(cal_time_start) - 1], degree = 3)13  0.00763
#> bs(j, knots = cal_time_start[1:length(cal_time_start) - 1], degree = 3)14  0.05718
#> bs(j, knots = cal_time_start[1:length(cal_time_start) - 1], degree = 3)15  0.01124
#>                                                                              
#> (Intercept)                                                                  
#> as.factor(treatment)1                                                     ***
#> as.factor(treatment)2                                                     ***
#> bs(j, knots = cal_time_start[1:length(cal_time_start) - 1], degree = 3)1     
#> bs(j, knots = cal_time_start[1:length(cal_time_start) - 1], degree = 3)2  *  
#> bs(j, knots = cal_time_start[1:length(cal_time_start) - 1], degree = 3)3     
#> bs(j, knots = cal_time_start[1:length(cal_time_start) - 1], degree = 3)4     
#> bs(j, knots = cal_time_start[1:length(cal_time_start) - 1], degree = 3)5     
#> bs(j, knots = cal_time_start[1:length(cal_time_start) - 1], degree = 3)6     
#> bs(j, knots = cal_time_start[1:length(cal_time_start) - 1], degree = 3)7  *  
#> bs(j, knots = cal_time_start[1:length(cal_time_start) - 1], degree = 3)8  ***
#> bs(j, knots = cal_time_start[1:length(cal_time_start) - 1], degree = 3)9  *  
#> bs(j, knots = cal_time_start[1:length(cal_time_start) - 1], degree = 3)10 *  
#> bs(j, knots = cal_time_start[1:length(cal_time_start) - 1], degree = 3)11 ** 
#> bs(j, knots = cal_time_start[1:length(cal_time_start) - 1], degree = 3)12 ** 
#> bs(j, knots = cal_time_start[1:length(cal_time_start) - 1], degree = 3)13 ** 
#> bs(j, knots = cal_time_start[1:length(cal_time_start) - 1], degree = 3)14 .  
#> bs(j, knots = cal_time_start[1:length(cal_time_start) - 1], degree = 3)15 *  
#> ---
#> Signif. codes:  0 '***' 0.001 '**' 0.01 '*' 0.05 '.' 0.1 ' ' 1
#> 
#> Residual standard error: 1.013 on 1207 degrees of freedom
#> Multiple R-squared:  0.6213, Adjusted R-squared:  0.616 
#> F-statistic: 116.5 on 17 and 1207 DF,  p-value: < 2.2e-16
```

### 3.8.6 Stepwise trend, \(\lambda=5\)

```
cal_time_start <- c()
for (i in unique(flair_data_step_5$cal_time)) {
  cal_time_start <- c(cal_time_start, max(flair_data_step_5[flair_data_step_5$cal_time==i,]$j))
}

mod_splines_cal <- lm(response ~ as.factor(treatment) + bs(j, knots = cal_time_start[1:length(cal_time_start)-1], degree = 3), flair_data_step_5)
summary(mod_splines_cal)
#> 
#> Call:
#> lm(formula = response ~ as.factor(treatment) + bs(j, knots = cal_time_start[1:length(cal_time_start) - 
#>     1], degree = 3), data = flair_data_step_5)
#> 
#> Residuals:
#>     Min      1Q  Median      3Q     Max 
#> -4.3677 -0.7099  0.0062  0.6869  3.8956 
#> 
#> Coefficients:
#>                                                                           Estimate
#> (Intercept)                                                                0.44175
#> as.factor(treatment)1                                                      0.75922
#> as.factor(treatment)2                                                      2.12769
#> bs(j, knots = cal_time_start[1:length(cal_time_start) - 1], degree = 3)1  -0.47285
#> bs(j, knots = cal_time_start[1:length(cal_time_start) - 1], degree = 3)2  -0.80296
#> bs(j, knots = cal_time_start[1:length(cal_time_start) - 1], degree = 3)3  -0.26270
#> bs(j, knots = cal_time_start[1:length(cal_time_start) - 1], degree = 3)4  -0.26457
#> bs(j, knots = cal_time_start[1:length(cal_time_start) - 1], degree = 3)5  -0.60766
#> bs(j, knots = cal_time_start[1:length(cal_time_start) - 1], degree = 3)6  -0.20095
#> bs(j, knots = cal_time_start[1:length(cal_time_start) - 1], degree = 3)7  -1.45687
#> bs(j, knots = cal_time_start[1:length(cal_time_start) - 1], degree = 3)8   6.70571
#> bs(j, knots = cal_time_start[1:length(cal_time_start) - 1], degree = 3)9   3.57943
#> bs(j, knots = cal_time_start[1:length(cal_time_start) - 1], degree = 3)10  4.76769
#> bs(j, knots = cal_time_start[1:length(cal_time_start) - 1], degree = 3)11  4.49970
#> bs(j, knots = cal_time_start[1:length(cal_time_start) - 1], degree = 3)12  4.82323
#> bs(j, knots = cal_time_start[1:length(cal_time_start) - 1], degree = 3)13  4.58637
#> bs(j, knots = cal_time_start[1:length(cal_time_start) - 1], degree = 3)14  4.40963
#> bs(j, knots = cal_time_start[1:length(cal_time_start) - 1], degree = 3)15  5.02314
#>                                                                           Std. Error
#> (Intercept)                                                                  0.34650
#> as.factor(treatment)1                                                        0.07686
#> as.factor(treatment)2                                                        0.09190
#> bs(j, knots = cal_time_start[1:length(cal_time_start) - 1], degree = 3)1     0.63147
#> bs(j, knots = cal_time_start[1:length(cal_time_start) - 1], degree = 3)2     0.40159
#> bs(j, knots = cal_time_start[1:length(cal_time_start) - 1], degree = 3)3     0.47672
#> bs(j, knots = cal_time_start[1:length(cal_time_start) - 1], degree = 3)4     0.40410
#> bs(j, knots = cal_time_start[1:length(cal_time_start) - 1], degree = 3)5     0.43327
#> bs(j, knots = cal_time_start[1:length(cal_time_start) - 1], degree = 3)6     0.41508
#> bs(j, knots = cal_time_start[1:length(cal_time_start) - 1], degree = 3)7     0.42424
#> bs(j, knots = cal_time_start[1:length(cal_time_start) - 1], degree = 3)8     0.42146
#> bs(j, knots = cal_time_start[1:length(cal_time_start) - 1], degree = 3)9     0.42364
#> bs(j, knots = cal_time_start[1:length(cal_time_start) - 1], degree = 3)10    0.43033
#> bs(j, knots = cal_time_start[1:length(cal_time_start) - 1], degree = 3)11    0.43695
#> bs(j, knots = cal_time_start[1:length(cal_time_start) - 1], degree = 3)12    0.47561
#> bs(j, knots = cal_time_start[1:length(cal_time_start) - 1], degree = 3)13    0.50425
#> bs(j, knots = cal_time_start[1:length(cal_time_start) - 1], degree = 3)14    0.50360
#> bs(j, knots = cal_time_start[1:length(cal_time_start) - 1], degree = 3)15    0.67346
#>                                                                           t value
#> (Intercept)                                                                 1.275
#> as.factor(treatment)1                                                       9.877
#> as.factor(treatment)2                                                      23.151
#> bs(j, knots = cal_time_start[1:length(cal_time_start) - 1], degree = 3)1   -0.749
#> bs(j, knots = cal_time_start[1:length(cal_time_start) - 1], degree = 3)2   -1.999
#> bs(j, knots = cal_time_start[1:length(cal_time_start) - 1], degree = 3)3   -0.551
#> bs(j, knots = cal_time_start[1:length(cal_time_start) - 1], degree = 3)4   -0.655
#> bs(j, knots = cal_time_start[1:length(cal_time_start) - 1], degree = 3)5   -1.403
#> bs(j, knots = cal_time_start[1:length(cal_time_start) - 1], degree = 3)6   -0.484
#> bs(j, knots = cal_time_start[1:length(cal_time_start) - 1], degree = 3)7   -3.434
#> bs(j, knots = cal_time_start[1:length(cal_time_start) - 1], degree = 3)8   15.911
#> bs(j, knots = cal_time_start[1:length(cal_time_start) - 1], degree = 3)9    8.449
#> bs(j, knots = cal_time_start[1:length(cal_time_start) - 1], degree = 3)10  11.079
#> bs(j, knots = cal_time_start[1:length(cal_time_start) - 1], degree = 3)11  10.298
#> bs(j, knots = cal_time_start[1:length(cal_time_start) - 1], degree = 3)12  10.141
#> bs(j, knots = cal_time_start[1:length(cal_time_start) - 1], degree = 3)13   9.095
#> bs(j, knots = cal_time_start[1:length(cal_time_start) - 1], degree = 3)14   8.756
#> bs(j, knots = cal_time_start[1:length(cal_time_start) - 1], degree = 3)15   7.459
#>                                                                           Pr(>|t|)
#> (Intercept)                                                               0.202593
#> as.factor(treatment)1                                                      < 2e-16
#> as.factor(treatment)2                                                      < 2e-16
#> bs(j, knots = cal_time_start[1:length(cal_time_start) - 1], degree = 3)1  0.454111
#> bs(j, knots = cal_time_start[1:length(cal_time_start) - 1], degree = 3)2  0.045783
#> bs(j, knots = cal_time_start[1:length(cal_time_start) - 1], degree = 3)3  0.581693
#> bs(j, knots = cal_time_start[1:length(cal_time_start) - 1], degree = 3)4  0.512774
#> bs(j, knots = cal_time_start[1:length(cal_time_start) - 1], degree = 3)5  0.161023
#> bs(j, knots = cal_time_start[1:length(cal_time_start) - 1], degree = 3)6  0.628393
#> bs(j, knots = cal_time_start[1:length(cal_time_start) - 1], degree = 3)7  0.000615
#> bs(j, knots = cal_time_start[1:length(cal_time_start) - 1], degree = 3)8   < 2e-16
#> bs(j, knots = cal_time_start[1:length(cal_time_start) - 1], degree = 3)9   < 2e-16
#> bs(j, knots = cal_time_start[1:length(cal_time_start) - 1], degree = 3)10  < 2e-16
#> bs(j, knots = cal_time_start[1:length(cal_time_start) - 1], degree = 3)11  < 2e-16
#> bs(j, knots = cal_time_start[1:length(cal_time_start) - 1], degree = 3)12  < 2e-16
#> bs(j, knots = cal_time_start[1:length(cal_time_start) - 1], degree = 3)13  < 2e-16
#> bs(j, knots = cal_time_start[1:length(cal_time_start) - 1], degree = 3)14  < 2e-16
#> bs(j, knots = cal_time_start[1:length(cal_time_start) - 1], degree = 3)15 1.66e-13
#>                                                                              
#> (Intercept)                                                                  
#> as.factor(treatment)1                                                     ***
#> as.factor(treatment)2                                                     ***
#> bs(j, knots = cal_time_start[1:length(cal_time_start) - 1], degree = 3)1     
#> bs(j, knots = cal_time_start[1:length(cal_time_start) - 1], degree = 3)2  *  
#> bs(j, knots = cal_time_start[1:length(cal_time_start) - 1], degree = 3)3     
#> bs(j, knots = cal_time_start[1:length(cal_time_start) - 1], degree = 3)4     
#> bs(j, knots = cal_time_start[1:length(cal_time_start) - 1], degree = 3)5     
#> bs(j, knots = cal_time_start[1:length(cal_time_start) - 1], degree = 3)6     
#> bs(j, knots = cal_time_start[1:length(cal_time_start) - 1], degree = 3)7  ***
#> bs(j, knots = cal_time_start[1:length(cal_time_start) - 1], degree = 3)8  ***
#> bs(j, knots = cal_time_start[1:length(cal_time_start) - 1], degree = 3)9  ***
#> bs(j, knots = cal_time_start[1:length(cal_time_start) - 1], degree = 3)10 ***
#> bs(j, knots = cal_time_start[1:length(cal_time_start) - 1], degree = 3)11 ***
#> bs(j, knots = cal_time_start[1:length(cal_time_start) - 1], degree = 3)12 ***
#> bs(j, knots = cal_time_start[1:length(cal_time_start) - 1], degree = 3)13 ***
#> bs(j, knots = cal_time_start[1:length(cal_time_start) - 1], degree = 3)14 ***
#> bs(j, knots = cal_time_start[1:length(cal_time_start) - 1], degree = 3)15 ***
#> ---
#> Signif. codes:  0 '***' 0.001 '**' 0.01 '*' 0.05 '.' 0.1 ' ' 1
#> 
#> Residual standard error: 1.088 on 1207 degrees of freedom
#> Multiple R-squared:  0.8764, Adjusted R-squared:  0.8747 
#> F-statistic: 503.6 on 17 and 1207 DF,  p-value: < 2.2e-16
```

## 3.9 Pooled analysis

### 3.9.1 Linear trend, \(\lambda=0.25\)

```
mod_pool <- lm(response ~ as.factor(treatment), flair_data_lin_0.25 %>% filter(treatment %in% c(0, 2)))
summary(mod_pool)
#> 
#> Call:
#> lm(formula = response ~ as.factor(treatment), data = flair_data_lin_0.25 %>% 
#>     filter(treatment %in% c(0, 2)))
#> 
#> Residuals:
#>      Min       1Q   Median       3Q      Max 
#> -3.11817 -0.68686 -0.01985  0.66849  2.91151 
#> 
#> Coefficients:
#>                       Estimate Std. Error t value Pr(>|t|)    
#> (Intercept)            0.13189    0.04136   3.189  0.00148 ** 
#> as.factor(treatment)2  2.13425    0.07429  28.728  < 2e-16 ***
#> ---
#> Signif. codes:  0 '***' 0.001 '**' 0.01 '*' 0.05 '.' 0.1 ' ' 1
#> 
#> Residual standard error: 0.9952 on 837 degrees of freedom
#> Multiple R-squared:  0.4965, Adjusted R-squared:  0.4959 
#> F-statistic: 825.3 on 1 and 837 DF,  p-value: < 2.2e-16
```

### 3.9.2 Linear trend, \(\lambda=1.5\)

```
mod_pool <- lm(response ~ as.factor(treatment), flair_data_lin_1.5 %>% filter(treatment %in% c(0, 2)))
summary(mod_pool)
#> 
#> Call:
#> lm(formula = response ~ as.factor(treatment), data = flair_data_lin_1.5 %>% 
#>     filter(treatment %in% c(0, 2)))
#> 
#> Residuals:
#>     Min      1Q  Median      3Q     Max 
#> -3.3474 -0.7641  0.0193  0.7119  3.0049 
#> 
#> Coefficients:
#>                       Estimate Std. Error t value Pr(>|t|)    
#> (Intercept)            0.74887    0.04509   16.61   <2e-16 ***
#> as.factor(treatment)2  2.48818    0.08100   30.72   <2e-16 ***
#> ---
#> Signif. codes:  0 '***' 0.001 '**' 0.01 '*' 0.05 '.' 0.1 ' ' 1
#> 
#> Residual standard error: 1.085 on 837 degrees of freedom
#> Multiple R-squared:  0.5299, Adjusted R-squared:  0.5294 
#> F-statistic: 943.6 on 1 and 837 DF,  p-value: < 2.2e-16
```

### 3.9.3 Linear trend, \(\lambda=5\)

```
mod_pool <- lm(response ~ as.factor(treatment), flair_data_lin_5 %>% filter(treatment %in% c(0, 2)))
summary(mod_pool)
#> 
#> Call:
#> lm(formula = response ~ as.factor(treatment), data = flair_data_lin_5 %>% 
#>     filter(treatment %in% c(0, 2)))
#> 
#> Residuals:
#>    Min     1Q Median     3Q    Max 
#> -4.433 -1.283 -0.021  1.222  4.260 
#> 
#> Coefficients:
#>                       Estimate Std. Error t value Pr(>|t|)    
#> (Intercept)             2.4764     0.0698   35.48   <2e-16 ***
#> as.factor(treatment)2   3.4792     0.1254   27.75   <2e-16 ***
#> ---
#> Signif. codes:  0 '***' 0.001 '**' 0.01 '*' 0.05 '.' 0.1 ' ' 1
#> 
#> Residual standard error: 1.68 on 837 degrees of freedom
#> Multiple R-squared:  0.4791, Adjusted R-squared:  0.4785 
#> F-statistic: 769.9 on 1 and 837 DF,  p-value: < 2.2e-16
```

### 3.9.4 Stepwise trend, \(\lambda=0.25\)

```
mod_pool <- lm(response ~ as.factor(treatment), flair_data_step_0.25 %>% filter(treatment %in% c(0, 2)))
summary(mod_pool)
#> 
#> Call:
#> lm(formula = response ~ as.factor(treatment), data = flair_data_step_0.25 %>% 
#>     filter(treatment %in% c(0, 2)))
#> 
#> Residuals:
#>     Min      1Q  Median      3Q     Max 
#> -3.1859 -0.6869 -0.0140  0.6748  2.9664 
#> 
#> Coefficients:
#>                       Estimate Std. Error t value Pr(>|t|)    
#> (Intercept)            0.12206    0.04151   2.941  0.00337 ** 
#> as.factor(treatment)2  2.19991    0.07456  29.504  < 2e-16 ***
#> ---
#> Signif. codes:  0 '***' 0.001 '**' 0.01 '*' 0.05 '.' 0.1 ' ' 1
#> 
#> Residual standard error: 0.9988 on 837 degrees of freedom
#> Multiple R-squared:  0.5098, Adjusted R-squared:  0.5092 
#> F-statistic: 870.5 on 1 and 837 DF,  p-value: < 2.2e-16
```

### 3.9.5 Stepwise trend, \(\lambda=1.5\)

```
mod_pool <- lm(response ~ as.factor(treatment), flair_data_step_1.5 %>% filter(treatment %in% c(0, 2)))
summary(mod_pool)
#> 
#> Call:
#> lm(formula = response ~ as.factor(treatment), data = flair_data_step_1.5 %>% 
#>     filter(treatment %in% c(0, 2)))
#> 
#> Residuals:
#>     Min      1Q  Median      3Q     Max 
#> -3.7537 -0.7912 -0.0173  0.7513  3.2452 
#> 
#> Coefficients:
#>                       Estimate Std. Error t value Pr(>|t|)    
#> (Intercept)            0.68985    0.04945   13.95   <2e-16 ***
#> as.factor(treatment)2  2.88212    0.08883   32.45   <2e-16 ***
#> ---
#> Signif. codes:  0 '***' 0.001 '**' 0.01 '*' 0.05 '.' 0.1 ' ' 1
#> 
#> Residual standard error: 1.19 on 837 degrees of freedom
#> Multiple R-squared:  0.5571, Adjusted R-squared:  0.5565 
#> F-statistic:  1053 on 1 and 837 DF,  p-value: < 2.2e-16
```

### 3.9.6 Stepwise trend, \(\lambda=5\)

```
mod_pool <- lm(response ~ as.factor(treatment), flair_data_step_5 %>% filter(treatment %in% c(0, 2)))
summary(mod_pool)
#> 
#> Call:
#> lm(formula = response ~ as.factor(treatment), data = flair_data_step_5 %>% 
#>     filter(treatment %in% c(0, 2)))
#> 
#> Residuals:
#>     Min      1Q  Median      3Q     Max 
#> -5.3435 -1.9648 -0.2527  1.9767  5.1553 
#> 
#> Coefficients:
#>                       Estimate Std. Error t value Pr(>|t|)    
#> (Intercept)            2.27966    0.09691   23.52   <2e-16 ***
#> as.factor(treatment)2  4.79231    0.17409   27.53   <2e-16 ***
#> ---
#> Signif. codes:  0 '***' 0.001 '**' 0.01 '*' 0.05 '.' 0.1 ' ' 1
#> 
#> Residual standard error: 2.332 on 837 degrees of freedom
#> Multiple R-squared:  0.4752, Adjusted R-squared:  0.4745 
#> F-statistic: 757.8 on 1 and 837 DF,  p-value: < 2.2e-16
```

## 3.10 Separate analysis

### 3.10.1 Linear trend, \(\lambda=0.25\)

```
mod_sep <- lm(response ~ as.factor(treatment), flair_data_lin_0.25 %>% filter(treatment %in% c(0, 2), period %in% c(2, 3)))
summary(mod_sep)
#> 
#> Call:
#> lm(formula = response ~ as.factor(treatment), data = flair_data_lin_0.25 %>% 
#>     filter(treatment %in% c(0, 2), period %in% c(2, 3)))
#> 
#> Residuals:
#>      Min       1Q   Median       3Q      Max 
#> -2.61471 -0.71663  0.01876  0.66833  2.91151 
#> 
#> Coefficients:
#>                       Estimate Std. Error t value Pr(>|t|)    
#> (Intercept)            0.25883    0.06303   4.106 4.67e-05 ***
#> as.factor(treatment)2  2.00731    0.08940  22.453  < 2e-16 ***
#> ---
#> Signif. codes:  0 '***' 0.001 '**' 0.01 '*' 0.05 '.' 0.1 ' ' 1
#> 
#> Residual standard error: 1.022 on 521 degrees of freedom
#> Multiple R-squared:  0.4918, Adjusted R-squared:  0.4908 
#> F-statistic: 504.1 on 1 and 521 DF,  p-value: < 2.2e-16
```

### 3.10.2 Linear trend, \(\lambda=1.5\)

```
mod_sep <- lm(response ~ as.factor(treatment), flair_data_lin_1.5 %>% filter(treatment %in% c(0, 2), period %in% c(2, 3)))
summary(mod_sep)
#> 
#> Call:
#> lm(formula = response ~ as.factor(treatment), data = flair_data_lin_1.5 %>% 
#>     filter(treatment %in% c(0, 2), period %in% c(2, 3)))
#> 
#> Residuals:
#>      Min       1Q   Median       3Q      Max 
#> -2.69503 -0.70965  0.01201  0.69187  3.00489 
#> 
#> Coefficients:
#>                       Estimate Std. Error t value Pr(>|t|)    
#> (Intercept)            1.23609    0.06487   19.05   <2e-16 ***
#> as.factor(treatment)2  2.00096    0.09201   21.75   <2e-16 ***
#> ---
#> Signif. codes:  0 '***' 0.001 '**' 0.01 '*' 0.05 '.' 0.1 ' ' 1
#> 
#> Residual standard error: 1.052 on 521 degrees of freedom
#> Multiple R-squared:  0.4758, Adjusted R-squared:  0.4748 
#> F-statistic:   473 on 1 and 521 DF,  p-value: < 2.2e-16
```

### 3.10.3 Linear trend, \(\lambda=5\)

```
mod_sep <- lm(response ~ as.factor(treatment), flair_data_lin_5 %>% filter(treatment %in% c(0, 2), period %in% c(2, 3)))
summary(mod_sep)
#> 
#> Call:
#> lm(formula = response ~ as.factor(treatment), data = flair_data_lin_5 %>% 
#>     filter(treatment %in% c(0, 2), period %in% c(2, 3)))
#> 
#> Residuals:
#>     Min      1Q  Median      3Q     Max 
#> -3.0383 -0.8657 -0.0107  0.8936  3.2664 
#> 
#> Coefficients:
#>                       Estimate Std. Error t value Pr(>|t|)    
#> (Intercept)            3.97241    0.07744   51.29   <2e-16 ***
#> as.factor(treatment)2  1.98317    0.10984   18.05   <2e-16 ***
#> ---
#> Signif. codes:  0 '***' 0.001 '**' 0.01 '*' 0.05 '.' 0.1 ' ' 1
#> 
#> Residual standard error: 1.256 on 521 degrees of freedom
#> Multiple R-squared:  0.3849, Adjusted R-squared:  0.3837 
#> F-statistic:   326 on 1 and 521 DF,  p-value: < 2.2e-16
```

### 3.10.4 Stepwise trend, \(\lambda=0.25\)

```
mod_sep <- lm(response ~ as.factor(treatment), flair_data_step_0.25 %>% filter(treatment %in% c(0, 2), period %in% c(2, 3)))
summary(mod_sep)
#> 
#> Call:
#> lm(formula = response ~ as.factor(treatment), data = flair_data_step_0.25 %>% 
#>     filter(treatment %in% c(0, 2), period %in% c(2, 3)))
#> 
#> Residuals:
#>      Min       1Q   Median       3Q      Max 
#> -2.59865 -0.71648  0.01151  0.66612  2.96638 
#> 
#> Coefficients:
#>                       Estimate Std. Error t value Pr(>|t|)    
#> (Intercept)            0.31338    0.06286   4.985 8.44e-07 ***
#> as.factor(treatment)2  2.00858    0.08916  22.528  < 2e-16 ***
#> ---
#> Signif. codes:  0 '***' 0.001 '**' 0.01 '*' 0.05 '.' 0.1 ' ' 1
#> 
#> Residual standard error: 1.019 on 521 degrees of freedom
#> Multiple R-squared:  0.4934, Adjusted R-squared:  0.4925 
#> F-statistic: 507.5 on 1 and 521 DF,  p-value: < 2.2e-16
```

### 3.10.5 Stepwise trend, \(\lambda=1.5\)

```
mod_sep <- lm(response ~ as.factor(treatment), flair_data_step_1.5 %>% filter(treatment %in% c(0, 2), period %in% c(2, 3)))
summary(mod_sep)
#> 
#> Call:
#> lm(formula = response ~ as.factor(treatment), data = flair_data_step_1.5 %>% 
#>     filter(treatment %in% c(0, 2), period %in% c(2, 3)))
#> 
#> Residuals:
#>      Min       1Q   Median       3Q      Max 
#> -2.59865 -0.71648  0.01151  0.66612  2.96638 
#> 
#> Coefficients:
#>                       Estimate Std. Error t value Pr(>|t|)    
#> (Intercept)            1.56338    0.06286   24.87   <2e-16 ***
#> as.factor(treatment)2  2.00858    0.08916   22.53   <2e-16 ***
#> ---
#> Signif. codes:  0 '***' 0.001 '**' 0.01 '*' 0.05 '.' 0.1 ' ' 1
#> 
#> Residual standard error: 1.019 on 521 degrees of freedom
#> Multiple R-squared:  0.4934, Adjusted R-squared:  0.4925 
#> F-statistic: 507.5 on 1 and 521 DF,  p-value: < 2.2e-16
```

### 3.10.6 Stepwise trend, \(\lambda=5\)

```
mod_sep <- lm(response ~ as.factor(treatment), flair_data_step_5 %>% filter(treatment %in% c(0, 2), period %in% c(2, 3)))
summary(mod_sep)
#> 
#> Call:
#> lm(formula = response ~ as.factor(treatment), data = flair_data_step_5 %>% 
#>     filter(treatment %in% c(0, 2), period %in% c(2, 3)))
#> 
#> Residuals:
#>      Min       1Q   Median       3Q      Max 
#> -2.59865 -0.71648  0.01151  0.66612  2.96638 
#> 
#> Coefficients:
#>                       Estimate Std. Error t value Pr(>|t|)    
#> (Intercept)            5.06338    0.06286   80.55   <2e-16 ***
#> as.factor(treatment)2  2.00858    0.08916   22.53   <2e-16 ***
#> ---
#> Signif. codes:  0 '***' 0.001 '**' 0.01 '*' 0.05 '.' 0.1 ' ' 1
#> 
#> Residual standard error: 1.019 on 521 degrees of freedom
#> Multiple R-squared:  0.4934, Adjusted R-squared:  0.4925 
#> F-statistic: 507.5 on 1 and 521 DF,  p-value: < 2.2e-16
```

# 4 Bias simulations

```
n_sim <- 10000    
set.seed(123)

res_fix_step_0.25 <- c()
for (i in 1:n_sim) {
  res_fix_step_0.25 <- c(res_fix_step_0.25, fixmodel_cont(sim_flair(lambda = 0.25, trend_pattern = "stepwise"), 2)$treat_effect - (-log(0.13)))
  if (i %in% seq(1000, 10000, by=1000)) { # keep track of the progress
    print(i) 
  }
}
#> [1] 1000
#> [1] 2000
#> [1] 3000
#> [1] 4000
#> [1] 5000
#> [1] 6000
#> [1] 7000
#> [1] 8000
#> [1] 9000
#> [1] 10000
```

```
t.test(res_fix_step_0.25)
#> 
#>  One Sample t-test
#> 
#> data:  res_fix_step_0.25
#> t = 0.32332, df = 9999, p-value = 0.7465
#> alternative hypothesis: true mean is not equal to 0
#> 95 percent confidence interval:
#>  -0.001390240  0.001939441
#> sample estimates:
#>    mean of x 
#> 0.0002746004
```

```
res_splines_step_0.25 <- c()
for (i in 1:n_sim) {
  res_splines_step_0.25 <- c(res_splines_step_0.25, splines_cont(sim_flair(lambda = 0.25, trend_pattern = "stepwise"), 2)$treat_effect - (-log(0.13)))
  if (i %in% seq(1000, 10000, by=1000)) { # keep track of the progress
    print(i) 
  }
}
#> [1] 1000
#> [1] 2000
#> [1] 3000
#> [1] 4000
#> [1] 5000
#> [1] 6000
#> [1] 7000
#> [1] 8000
#> [1] 9000
#> [1] 10000
```

```
t.test(res_splines_step_0.25)
#> 
#>  One Sample t-test
#> 
#> data:  res_splines_step_0.25
#> t = 17.438, df = 9999, p-value < 2.2e-16
#> alternative hypothesis: true mean is not equal to 0
#> 95 percent confidence interval:
#>  0.01298202 0.01627029
#> sample estimates:
#>  mean of x 
#> 0.01462616
```

```
res_mix_step_0.25 <- c()
for (i in 1:n_sim) {
  res_mix_step_0.25 <- c(res_mix_step_0.25, mixmodel_cont(sim_flair(lambda = 0.25, trend_pattern = "stepwise"), 2)$treat_effect - (-log(0.13)))
  if (i %in% seq(1000, 10000, by=1000)) { # keep track of the progress
    print(i) 
  }
}
#> [1] 1000
#> [1] 2000
#> [1] 3000
#> [1] 4000
#> [1] 5000
#> [1] 6000
#> [1] 7000
#> [1] 8000
#> [1] 9000
#> [1] 10000
```

```
t.test(res_mix_step_0.25)
#> 
#>  One Sample t-test
#> 
#> data:  res_mix_step_0.25
#> t = 22.687, df = 9999, p-value < 2.2e-16
#> alternative hypothesis: true mean is not equal to 0
#> 95 percent confidence interval:
#>  0.01792641 0.02131705
#> sample estimates:
#>  mean of x 
#> 0.01962173
```

```
res_mix_ar1_step_0.25 <- c()
for (i in 1:n_sim) {
  res_mix_ar1_step_0.25 <- c(res_mix_ar1_step_0.25, mixmodel_AR1_cont(sim_flair(lambda = 0.25, trend_pattern = "stepwise"), 2)$treat_effect - (-log(0.13)))
  if (i %in% seq(1000, 10000, by=1000)) { # keep track of the progress
    print(i) 
  }
}
#> [1] 1000
#> [1] 2000
#> [1] 3000
#> [1] 4000
#> [1] 5000
#> [1] 6000
#> [1] 7000
#> [1] 8000
#> [1] 9000
#> [1] 10000
```

```
t.test(res_mix_ar1_step_0.25)
#> 
#>  One Sample t-test
#> 
#> data:  res_mix_ar1_step_0.25
#> t = 41.027, df = 9999, p-value < 2.2e-16
#> alternative hypothesis: true mean is not equal to 0
#> 95 percent confidence interval:
#>  0.03510793 0.03863108
#> sample estimates:
#>  mean of x 
#> 0.03686951
```

## 4.1 Figure S12

```
bias_res <- data.frame(model = c(rep("Fixed effect model", n_sim),
                                 rep("Spline regression", n_sim),
                                 rep("Mixed model", n_sim),
                                 rep("Mixed model (AR1)", n_sim)),
                       bias = c(res_fix_step_0.25,
                                res_splines_step_0.25,
                                res_mix_step_0.25,
                                res_mix_ar1_step_0.25))
  
  
ggplot(bias_res) +
  geom_histogram(aes(bias), color="darkred", fill="darkred", alpha=0.6, bins = 40) +
  facet_wrap(~ model, nrow=2) +
  labs(x = "Estimation error", y = "Count") +
  geom_vline(xintercept = 0, color = "black") +
  theme_bw(base_size = 13.8)
```

```
ggsave("figures/FLAIR_bias.png", width = 7, height = 6)
ggsave("figures/FLAIR_bias.pdf", width = 7, height = 6)
ggsave("figures/FLAIR_bias.tiff", width = 7, height = 6)
```
