# Supplementary material for: Statistical Modeling to Adjust for Time Trends in Adaptive Platform Trials Utilizing Non‐Concurrent Controls
Source: Biom J. 2025 Jun 10;67(3):e70059. doi: 10.1002/bimj.70059 (PMC12150008; doi:10.1002/bimj.70059)
Supplement: Supplementary file 1 — Supporting Information [file BIMJ-67-e70059-s002.zip › case_studies/PSP/NCC_FreqModels_case_study_PSP.html]

Statistical modeling to adjust for time trends in adaptive platform trials utilizing non-concurrent controls


Code 

- Show All Code
- Hide All Code

# Statistical modeling to adjust for time trends in adaptive platform trials utilizing non-concurrent controls

### Case study: PSP data

#### Pavla Krotka, Martin Posch, Mohamed Gewily, Günter Höglinger, Marta Bofill Roig

#### 2024

# 1 Introduction

This file contains all code to reproduce the case study using PSP
data presented in Section 5 of the paper *“Statistical modeling to
adjust for time trends in adaptive platform trials utilizing
non-concurrent controls”* by Pavla Krotka, Martin Posch, Mohamed
Gewily, Günter Höglinger, Marta Bofill Roig. The results obtained from
the analyses below are presented in Table 2.

# 2 Data preparation

```
improve_data <- read_csv("final_baseline.csv")
```

```
data_mod <- improve_data %>%
  filter(Study=="Abbvie", Item_number==35, Visit %in% c(1, 11, 14, 18)) %>%
  select(ID, response = DV, TRT, baseline_date, TIME, Treatment_number, Visit, Item_number) %>%
  mutate(treatment = as.factor(case_when(Treatment_number==1 ~ 1,
                                         Treatment_number==2 ~ 2,
                                         Treatment_number==3 ~ 0))) %>%
  arrange(baseline_date) %>%
  mutate(recruitment = as.numeric(baseline_date-min(baseline_date)+1),
         period = ifelse(baseline_date<as.Date("2018-06-01"), 1, 2),
         treatment_mod = as.factor(ifelse((period==1 & treatment==2), NA, as.numeric(treatment)-1)))

data_mod$response <- as.numeric(data_mod$response)
data_mod$TRT <- factor(data_mod$TRT, levels = c("Abbvie-Placebo", "Abbvie-ABBV-8E12 2000 MG", "Abbvie-ABBV-8E12 4000 MG"))
data_mod$period <- as.factor(data_mod$period)

data_mod_wide <- data_mod %>%
  select(-TIME) %>%
  pivot_wider(names_from = Visit, names_prefix = "response_visit_", values_from = response) %>%
  group_by(ID) %>%
  mutate(baseline_change_11 = response_visit_11-response_visit_1,
         baseline_change_14 = response_visit_14-response_visit_1,
         baseline_change_18 = response_visit_18-response_visit_1,
         cal_time = factor(ceiling(recruitment/90), levels = c(1:9)))

data_case_study <- data_mod_wide %>%
  select(ID, recruitment, baseline_date, response_visit_1, treatment = treatment_mod, period, cal_time) %>%
  na.omit()
```

# 3 Abbvie trial data - baseline measurement

## 3.1 Sample sizes per treatment arm

```
table(data_case_study$treatment)
#> 
#>   0   1   2 
#> 123 126  66
```

## 3.2 Sample size per period

```
table(data_case_study$period)
#> 
#>   1   2 
#> 116 199
```

## 3.3 Sample size per treatment arm and period

```
table(data_case_study$treatment, data_case_study$period)
#>    
#>      1  2
#>   0 55 68
#>   1 61 65
#>   2  0 66
```

## 3.4 Sample sizes per calendar time interval

```
table(data_case_study$cal_time)
#> 
#>  1  2  3  4  5  6  7  8  9 
#>  4 10 23 29 34 36 76 96  7
```

## 3.5 Sample sizes per treatment arm and calendar time interval

```
table(data_case_study$treatment, data_case_study$cal_time)
#>    
#>      1  2  3  4  5  6  7  8  9
#>   0  2  4 12 13 19 14 22 34  3
#>   1  2  6 11 16 15 15 29 29  3
#>   2  0  0  0  0  0  7 25 33  1
```

## 3.6 Time trend

```
test_trend <- lm(response_visit_1 ~ treatment + recruitment, data_case_study)
summary(test_trend)
#> 
#> Call:
#> lm(formula = response_visit_1 ~ treatment + recruitment, data = data_case_study)
#> 
#> Residuals:
#>     Min      1Q  Median      3Q     Max 
#> -24.008  -8.193  -0.149   7.505  33.548 
#> 
#> Coefficients:
#>              Estimate Std. Error t value Pr(>|t|)    
#> (Intercept) 41.115989   2.281091  18.025   <2e-16 ***
#> treatment1  -0.328929   1.480965  -0.222   0.8244    
#> treatment2  -0.515427   1.864973  -0.276   0.7824    
#> recruitment -0.008892   0.004119  -2.159   0.0316 *  
#> ---
#> Signif. codes:  0 '***' 0.001 '**' 0.01 '*' 0.05 '.' 0.1 ' ' 1
#> 
#> Residual standard error: 11.68 on 311 degrees of freedom
#> Multiple R-squared:  0.01782,    Adjusted R-squared:  0.008345 
#> F-statistic: 1.881 on 3 and 311 DF,  p-value: 0.1327
```

# 4 Figure 13

```
per2_start <- max(data_case_study[data_case_study$period==1,]$recruitment)

ggplot(data_case_study) +
  geom_point(aes(recruitment, response_visit_1, color = treatment)) +
  geom_line(aes(recruitment, fitted(test_trend), color = treatment), size = 1) +
  labs(x = "Trial duration in days", y = "PSPRS score at baseline", color = "Treatment:") +
  scale_color_viridis(discrete = T) +
  geom_vline(xintercept = 90*c(0:9), linetype = "dashed", color = "gray40") +
  geom_vline(xintercept = per2_start, linetype = "dashed", color = "darkred", size = 1) +
  theme_bw() +
  theme(legend.position = "bottom")
```

```
ggsave("figures/casestudy_trialdata.png", width = 7, height = 5)
ggsave("figures/casestudy_trialdata.pdf", width = 7, height = 5)
ggsave("figures/casestudy_trialdata.tiff", width = 7, height = 5)
```

# 5 Analysis approaches

## 5.1 Regression model - period adjustment

```
# Regression model - period adjustment
mod_lm_per <- lm(response_visit_1 ~ treatment + period, data_case_study)
summary(mod_lm_per)
#> 
#> Call:
#> lm(formula = response_visit_1 ~ treatment + period, data = data_case_study)
#> 
#> Residuals:
#>     Min      1Q  Median      3Q     Max 
#> -25.838  -8.052  -0.058   7.162  33.955 
#> 
#> Coefficients:
#>             Estimate Std. Error t value Pr(>|t|)    
#> (Intercept) 38.83764    1.33129  29.173   <2e-16 ***
#> treatment1  -0.37978    1.47707  -0.257   0.7973    
#> treatment2  -0.01233    1.89614  -0.007   0.9948    
#> period2     -3.77986    1.48041  -2.553   0.0111 *  
#> ---
#> Signif. codes:  0 '***' 0.001 '**' 0.01 '*' 0.05 '.' 0.1 ' ' 1
#> 
#> Residual standard error: 11.65 on 311 degrees of freedom
#> Multiple R-squared:  0.02357,    Adjusted R-squared:  0.01415 
#> F-statistic: 2.502 on 3 and 311 DF,  p-value: 0.05939
```

```
mod_lm_per_est <- summary(mod_lm_per)$coefficients["treatment2", "Estimate"]
mod_lm_per_se <- summary(mod_lm_per)$coefficients["treatment2", "Std. Error"]
mod_lm_per_pval <- summary(mod_lm_per)$coefficients["treatment2", "Pr(>|t|)"]
```

## 5.2 Regression model - calendar time adjustment

```
# Regression model - calendar time adjustment
mod_lm_cal <- lm(response_visit_1 ~ treatment + cal_time, data_case_study)
summary(mod_lm_cal)
#> 
#> Call:
#> lm(formula = response_visit_1 ~ treatment + cal_time, data = data_case_study)
#> 
#> Residuals:
#>     Min      1Q  Median      3Q     Max 
#> -25.925  -8.314  -0.404   7.524  34.409 
#> 
#> Coefficients:
#>             Estimate Std. Error t value Pr(>|t|)    
#> (Intercept) 33.87792    5.88910   5.753 2.14e-08 ***
#> treatment1  -0.25584    1.48737  -0.172    0.864    
#> treatment2  -0.06815    1.90023  -0.036    0.971    
#> cal_time2    3.87558    6.91389   0.561    0.576    
#> cal_time3    7.33139    6.32968   1.158    0.248    
#> cal_time4    4.88392    6.23231   0.784    0.434    
#> cal_time5    2.99966    6.17666   0.486    0.628    
#> cal_time6    5.04749    6.16732   0.818    0.414    
#> cal_time7    0.78159    6.02152   0.130    0.897    
#> cal_time8    0.59779    5.99255   0.100    0.921    
#> cal_time9    6.95575    7.32751   0.949    0.343    
#> ---
#> Signif. codes:  0 '***' 0.001 '**' 0.01 '*' 0.05 '.' 0.1 ' ' 1
#> 
#> Residual standard error: 11.68 on 304 degrees of freedom
#> Multiple R-squared:  0.03916,    Adjusted R-squared:  0.007554 
#> F-statistic: 1.239 on 10 and 304 DF,  p-value: 0.2653
```

```
mod_lm_cal_est <- summary(mod_lm_cal)$coefficients["treatment2", "Estimate"]
mod_lm_cal_se <- summary(mod_lm_cal)$coefficients["treatment2", "Std. Error"]
mod_lm_cal_pval <- summary(mod_lm_cal)$coefficients["treatment2", "Pr(>|t|)"]
```

## 5.3 Mixed model - calendar time adjustment

```
# Mixed model - calendar time adjustment
mod_mix_cal <- lmer(response_visit_1 ~ treatment + (1 | cal_time), data_case_study)
summary(mod_mix_cal)
#> Linear mixed model fit by REML. t-tests use Satterthwaite's method [
#> lmerModLmerTest]
#> Formula: response_visit_1 ~ treatment + (1 | cal_time)
#>    Data: data_case_study
#> 
#> REML criterion at convergence: 2435.2
#> 
#> Scaled residuals: 
#>      Min       1Q   Median       3Q      Max 
#> -2.14161 -0.72289 -0.04943  0.64452  2.92516 
#> 
#> Random effects:
#>  Groups   Name        Variance Std.Dev.
#>  cal_time (Intercept)   2.73    1.652  
#>  Residual             135.85   11.655  
#> Number of obs: 315, groups:  cal_time, 9
#> 
#> Fixed effects:
#>             Estimate Std. Error       df t value Pr(>|t|)    
#> (Intercept)  37.1470     1.2303  19.7350  30.194   <2e-16 ***
#> treatment1   -0.2531     1.4802 308.6543  -0.171    0.864    
#> treatment2   -0.9028     1.8326 276.7394  -0.493    0.623    
#> ---
#> Signif. codes:  0 '***' 0.001 '**' 0.01 '*' 0.05 '.' 0.1 ' ' 1
#> 
#> Correlation of Fixed Effects:
#>            (Intr) trtmn1
#> treatment1 -0.611       
#> treatment2 -0.458  0.410
```

```
mod_mix_cal_est <- summary(mod_mix_cal)$coefficients["treatment2", "Estimate"]
mod_mix_cal_se <- summary(mod_mix_cal)$coefficients["treatment2", "Std. Error"]
mod_mix_cal_pval <- summary(mod_mix_cal)$coefficients["treatment2", "Pr(>|t|)"]
```

## 5.4 Mixed model (AR1) - calendar time adjustment

```
# Mixed model - calendar time adjustment
mod_mix_ar1_cal <- fitme(response_visit_1 ~ treatment + AR1(1 | cal_time), data_case_study)

summary.HLfit(mod_mix_ar1_cal)
#> formula: response_visit_1 ~ treatment + AR1(1 | cal_time)
#> ML: Estimation of corrPars, lambda and phi by ML.
#>     Estimation of fixed effects by ML.
#> Estimation of lambda and phi by 'outer' ML, maximizing logL.
#> family: gaussian( link = identity ) 
#>  ------------ Fixed effects (beta) ------------
#>             Estimate Cond. SE t-value
#> (Intercept)  37.0623    1.242 29.8304
#> treatment1   -0.2495    1.474 -0.1692
#> treatment2   -0.9730    1.822 -0.5339
#>  --------------- Random effects ---------------
#> Family: gaussian( link = identity ) 
#>                    --- Correlation parameters:
#>   1.ARphi 
#> 0.2939867 
#>            --- Variance parameters ('lambda'):
#> lambda = var(u) for u ~ Gaussian; 
#>    cal_time  :  1.805  
#> # of obs: 315; # of groups: cal_time, 9 
#>  -------------- Residual variance  ------------
#> phi estimate was 134.986 
#>  ------------- Likelihood values  -------------
#>                         logLik
#> logL       (p_v(h)): -1221.074
```

```
res <- summary.HLfit(mod_mix_ar1_cal, verbose = FALSE)

IC <- get_any_IC(mod_mix_ar1_cal, verbose = FALSE)
IC
#>        marginal AIC:     conditional AIC:      dispersion AIC: 
#>            2454.1472            2448.3970            2448.1472 
#>        effective df: 
#>             310.1366
```

```
eff_df <- IC["       effective df:"] # effective degrees of freedom

2*(1-pt(abs(res$beta_table["treatment2", "t-value"]), eff_df)) # p-value
#> [1] 0.5937707
```

```
mod_mix_ar1_cal_est <- summary(mod_mix_ar1_cal, verbose = F)$beta_table["treatment2", "Estimate"]
mod_mix_ar1_cal_se <- summary(mod_mix_ar1_cal, verbose = F)$beta_table["treatment2", "Cond. SE"]
mod_mix_ar1_cal_pval <- 2*(1-pt(abs(res$beta_table["treatment2", "t-value"]), eff_df))
```

## 5.5 Spline regression - period adjustment

```
# Spline regression - period adjustment
per2_start <- max(data_case_study[data_case_study$period==1,]$recruitment)

mod_splines_per <- lm(response_visit_1 ~ treatment + bs(recruitment, knots = per2_start, degree = 3), data_case_study)
summary(mod_splines_per)
#> 
#> Call:
#> lm(formula = response_visit_1 ~ treatment + bs(recruitment, knots = per2_start, 
#>     degree = 3), data = data_case_study)
#> 
#> Residuals:
#>     Min      1Q  Median      3Q     Max 
#> -25.199  -8.174   0.082   7.206  34.545 
#> 
#> Coefficients:
#>                                                  Estimate Std. Error t value
#> (Intercept)                                       33.2030     6.4710   5.131
#> treatment1                                        -0.3345     1.4747  -0.227
#> treatment2                                         0.1784     1.8853   0.095
#> bs(recruitment, knots = per2_start, degree = 3)1   9.7351    10.9143   0.892
#> bs(recruitment, knots = per2_start, degree = 3)2   6.8196     7.0646   0.965
#> bs(recruitment, knots = per2_start, degree = 3)3  -2.1067     7.6734  -0.275
#> bs(recruitment, knots = per2_start, degree = 3)4   6.5296     6.9232   0.943
#>                                                  Pr(>|t|)    
#> (Intercept)                                      5.11e-07 ***
#> treatment1                                          0.821    
#> treatment2                                          0.925    
#> bs(recruitment, knots = per2_start, degree = 3)1    0.373    
#> bs(recruitment, knots = per2_start, degree = 3)2    0.335    
#> bs(recruitment, knots = per2_start, degree = 3)3    0.784    
#> bs(recruitment, knots = per2_start, degree = 3)4    0.346    
#> ---
#> Signif. codes:  0 '***' 0.001 '**' 0.01 '*' 0.05 '.' 0.1 ' ' 1
#> 
#> Residual standard error: 11.63 on 308 degrees of freedom
#> Multiple R-squared:  0.0356, Adjusted R-squared:  0.01681 
#> F-statistic: 1.895 on 6 and 308 DF,  p-value: 0.08135
```

```
mod_splines_per_est <- summary(mod_splines_per)$coefficients["treatment2", "Estimate"]
mod_splines_per_se <- summary(mod_splines_per)$coefficients["treatment2", "Std. Error"]
mod_splines_per_pval <- summary(mod_splines_per)$coefficients["treatment2", "Pr(>|t|)"]
```

## 5.6 Spline regression - calendar time adjustment

```
# Spline regression - calendar time adjustment
cal_time_start <- c()
for (i in unique(data_case_study$cal_time)) {
  cal_time_start <- c(cal_time_start, max(data_case_study[data_case_study$cal_time==i,]$recruitment))
}

cal_time_start <- cal_time_start[-length(cal_time_start)]

mod_splines_cal <- lm(response_visit_1 ~ treatment + bs(recruitment, knots = cal_time_start, degree = 3), data_case_study)
summary(mod_splines_cal)
#> 
#> Call:
#> lm(formula = response_visit_1 ~ treatment + bs(recruitment, knots = cal_time_start, 
#>     degree = 3), data = data_case_study)
#> 
#> Residuals:
#>     Min      1Q  Median      3Q     Max 
#> -26.864  -8.112  -0.304   8.051  34.125 
#> 
#> Coefficients:
#>                                                       Estimate Std. Error
#> (Intercept)                                            45.5599    10.2810
#> treatment1                                              0.2437     1.4710
#> treatment2                                              0.6239     1.8794
#> bs(recruitment, knots = cal_time_start, degree = 3)1  -43.4325    24.0912
#> bs(recruitment, knots = cal_time_start, degree = 3)2   17.1026    20.3063
#> bs(recruitment, knots = cal_time_start, degree = 3)3  -19.4255    14.1212
#> bs(recruitment, knots = cal_time_start, degree = 3)4    5.8126    11.0683
#> bs(recruitment, knots = cal_time_start, degree = 3)5  -17.4643    11.4435
#> bs(recruitment, knots = cal_time_start, degree = 3)6   -1.5275    11.0710
#> bs(recruitment, knots = cal_time_start, degree = 3)7  -14.4121    11.1524
#> bs(recruitment, knots = cal_time_start, degree = 3)8   -8.0005    11.0124
#> bs(recruitment, knots = cal_time_start, degree = 3)9  -18.1903    11.3156
#> bs(recruitment, knots = cal_time_start, degree = 3)10   6.2756    11.9817
#> bs(recruitment, knots = cal_time_start, degree = 3)11 -23.1058    14.0617
#>                                                       t value Pr(>|t|)    
#> (Intercept)                                             4.431 1.31e-05 ***
#> treatment1                                              0.166   0.8685    
#> treatment2                                              0.332   0.7401    
#> bs(recruitment, knots = cal_time_start, degree = 3)1   -1.803   0.0724 .  
#> bs(recruitment, knots = cal_time_start, degree = 3)2    0.842   0.4003    
#> bs(recruitment, knots = cal_time_start, degree = 3)3   -1.376   0.1700    
#> bs(recruitment, knots = cal_time_start, degree = 3)4    0.525   0.5999    
#> bs(recruitment, knots = cal_time_start, degree = 3)5   -1.526   0.1280    
#> bs(recruitment, knots = cal_time_start, degree = 3)6   -0.138   0.8904    
#> bs(recruitment, knots = cal_time_start, degree = 3)7   -1.292   0.1973    
#> bs(recruitment, knots = cal_time_start, degree = 3)8   -0.726   0.4681    
#> bs(recruitment, knots = cal_time_start, degree = 3)9   -1.608   0.1090    
#> bs(recruitment, knots = cal_time_start, degree = 3)10   0.524   0.6008    
#> bs(recruitment, knots = cal_time_start, degree = 3)11  -1.643   0.1014    
#> ---
#> Signif. codes:  0 '***' 0.001 '**' 0.01 '*' 0.05 '.' 0.1 ' ' 1
#> 
#> Residual standard error: 11.49 on 301 degrees of freedom
#> Multiple R-squared:  0.08037,    Adjusted R-squared:  0.04065 
#> F-statistic: 2.023 on 13 and 301 DF,  p-value: 0.0188
```

```
mod_splines_cal_est <- summary(mod_splines_cal)$coefficients["treatment2", "Estimate"]
mod_splines_cal_se <- summary(mod_splines_cal)$coefficients["treatment2", "Std. Error"]
mod_splines_cal_pval <- summary(mod_splines_cal)$coefficients["treatment2", "Pr(>|t|)"]
```

## 5.7 Pooled analysis

```
# Pooled analysis
mod_pool <- lm(response_visit_1 ~ treatment, data_case_study %>% filter(treatment %in% c(0, 2)))
summary(mod_pool)
#> 
#> Call:
#> lm(formula = response_visit_1 ~ treatment, data = data_case_study %>% 
#>     filter(treatment %in% c(0, 2)))
#> 
#> Residuals:
#>     Min      1Q  Median      3Q     Max 
#> -24.748  -7.748  -0.748   6.955  33.955 
#> 
#> Coefficients:
#>             Estimate Std. Error t value Pr(>|t|)    
#> (Intercept)   36.748      1.043  35.221   <2e-16 ***
#> treatment2    -1.703      1.766  -0.964    0.336    
#> ---
#> Signif. codes:  0 '***' 0.001 '**' 0.01 '*' 0.05 '.' 0.1 ' ' 1
#> 
#> Residual standard error: 11.57 on 187 degrees of freedom
#> Multiple R-squared:  0.004948,   Adjusted R-squared:  -0.0003733 
#> F-statistic: 0.9298 on 1 and 187 DF,  p-value: 0.3361
```

```
mod_pool_est <- summary(mod_pool)$coefficients["treatment2", "Estimate"]
mod_pool_se <- summary(mod_pool)$coefficients["treatment2", "Std. Error"]
mod_pool_pval <- summary(mod_pool)$coefficients["treatment2", "Pr(>|t|)"]
```

## 5.8 Separate analysis

```
# Separate analysis
mod_sep <- lm(response_visit_1 ~ treatment, data_case_study %>% filter(treatment %in% c(0, 2), period==2))
summary(mod_sep)
#> 
#> Call:
#> lm(formula = response_visit_1 ~ treatment, data = data_case_study %>% 
#>     filter(treatment %in% c(0, 2), period == 2))
#> 
#> Residuals:
#>     Min      1Q  Median      3Q     Max 
#> -23.456  -8.353  -1.045   6.397  33.955 
#> 
#> Coefficients:
#>             Estimate Std. Error t value Pr(>|t|)    
#> (Intercept)  35.4559     1.4263  24.859   <2e-16 ***
#> treatment2   -0.4104     2.0323  -0.202     0.84    
#> ---
#> Signif. codes:  0 '***' 0.001 '**' 0.01 '*' 0.05 '.' 0.1 ' ' 1
#> 
#> Residual standard error: 11.76 on 132 degrees of freedom
#> Multiple R-squared:  0.0003089,  Adjusted R-squared:  -0.007265 
#> F-statistic: 0.04079 on 1 and 132 DF,  p-value: 0.8403
```

```
mod_sep_est <- summary(mod_sep)$coefficients["treatment2", "Estimate"]
mod_sep_se <- summary(mod_sep)$coefficients["treatment2", "Std. Error"]
mod_sep_pval <- summary(mod_sep)$coefficients["treatment2", "Pr(>|t|)"]
```

# 6 TABLE 2

```
table_2 <- data.frame(`Analysis approach` = c("Fixed effect model", "Fixed effect model", "Mixed model", 
                                              "Mixed model (AR1)", "Spline regression", "Spline regression", "Pooled analysis", "Separate analysis"),
                      Adjustment = c("Periods", rep("Calendar time units", 3), "Periods", "Calendar time units", rep("-", 2)),
                      `Effect estimate` = round(c(mod_lm_per_est, mod_lm_cal_est, mod_mix_cal_est, mod_mix_ar1_cal_est, mod_splines_per_est, mod_splines_cal_est, mod_pool_est, mod_sep_est), 3),
                      `Std. error` = round(c(mod_lm_per_se, mod_lm_cal_se, mod_mix_cal_se, mod_mix_ar1_cal_se, mod_splines_per_se, mod_splines_cal_se, mod_pool_se, mod_sep_se), 3),
                      `p-value` = round(c(mod_lm_per_pval, mod_lm_cal_pval, mod_mix_cal_pval, mod_mix_ar1_cal_pval, mod_splines_per_pval, mod_splines_cal_pval, mod_pool_pval, mod_sep_pval), 3),
                      
                      check.names = F)

kable(table_2, booktabs = T) %>%
  kable_styling(bootstrap_options = "striped")
```

| Analysis approach | Adjustment | Effect estimate | Std. error | p-value |
| --- | --- | --- | --- | --- |
| Fixed effect model | Periods | -0.012 | 1.896 | 0.995 |
| Fixed effect model | Calendar time units | -0.068 | 1.900 | 0.971 |
| Mixed model | Calendar time units | -0.903 | 1.833 | 0.623 |
| Mixed model (AR1) | Calendar time units | -0.973 | 1.822 | 0.594 |
| Spline regression | Periods | 0.178 | 1.885 | 0.925 |
| Spline regression | Calendar time units | 0.624 | 1.879 | 0.740 |
| Pooled analysis |  | -1.703 | 1.766 | 0.336 |
| Separate analysis |  | -0.410 | 2.032 | 0.840 |
