# Supplementary material for: Statistical Modeling to Adjust for Time Trends in Adaptive Platform Trials Utilizing Non‐Concurrent Controls
Source: Biom J. 2025 Jun 10;67(3):e70059. doi: 10.1002/bimj.70059 (PMC12150008; doi:10.1002/bimj.70059)
Supplement: Supplementary file 1 — Supporting Information [file BIMJ-67-e70059-s002.zip › case_studies/PSP/NCC_FreqModels_case_study_PSP_synthetic.html]

Statistical modeling to adjust for time trends in adaptive platform trials utilizing non-concurrent controls


Code 

- Show All Code
- Hide All Code

# Statistical modeling to adjust for time trends in adaptive platform trials utilizing non-concurrent controls

### Case study: PSP data (synthetic)

#### Pavla Krotka, Martin Posch, Mohamed Gewily, Günter Höglinger, Marta Bofill Roig

#### 2024

# 1 Introduction

This file contains all code to generate synthetic data in order to
mimic the case study using PSP data presented in Section 5 of the paper
*“Statistical modeling to adjust for time trends in adaptive platform
trials utilizing non-concurrent controls”* by Pavla Krotka, Martin
Posch, Mohamed Gewily, Günter Höglinger, Marta Bofill Roig.

# 2 Generation of synthetic data

```
# Generate random values from the Beta distribution for patient recruitment
set.seed(22)
beta_values <- rbeta(315, 3, 1.5)

# Scale the values to the desired range (1 to 742)
recruitment <- round(1 + beta_values * (742 - 1))

# Generate randomized allocations and patient responses
set.seed(13)
period_1 <- sample(c(rep(0, 55), rep(1, 61)))
period_2 <- sample(c(rep(0, 68), rep(1, 65), rep(2, 66)))

resp_1 <- rnorm(n = 116, mean = 38.6, sd = 11)
resp_2 <- rnorm(n = 199, mean = 34.9, sd = 11)

# Create dataframe with trial data
data_case_study <- data.frame(recruitment = sort(recruitment),
                              response_visit_1 = c(resp_1, resp_2),
                              treatment = as.factor(c(period_1, period_2)),
                              period = c(rep(1, 116), rep(2, 199)),
                              cal_time = factor(ceiling(recruitment/90), levels = c(1:9)))
```

# 3 Abbvie trial data - baseline measurement

## 3.1 Sample sizes per treatment arm

```
table(data_case_study$treatment)
#> 
#>   0   1   2 
#> 123 126  66
```

## 3.2 Sample size per period

```
table(data_case_study$period)
#> 
#>   1   2 
#> 116 199
```

## 3.3 Sample size per treatment arm and period

```
table(data_case_study$treatment, data_case_study$period)
#>    
#>      1  2
#>   0 55 68
#>   1 61 65
#>   2  0 66
```

## 3.4 Sample sizes per calendar time interval

```
table(data_case_study$cal_time)
#> 
#>  1  2  3  4  5  6  7  8  9 
#>  2  5 20 31 45 55 92 60  5
```

## 3.5 Sample sizes per treatment arm and calendar time interval

```
table(data_case_study$treatment, data_case_study$cal_time)
#>    
#>      1  2  3  4  5  6  7  8  9
#>   0  1  1  8 10 21 20 40 19  3
#>   1  1  2  4 13 17 26 33 28  2
#>   2  0  2  8  8  7  9 19 13  0
```

## 3.6 Time trend

```
test_trend <- lm(response_visit_1 ~ treatment + recruitment, data_case_study)
summary(test_trend)
#> 
#> Call:
#> lm(formula = response_visit_1 ~ treatment + recruitment, data = data_case_study)
#> 
#> Residuals:
#>      Min       1Q   Median       3Q      Max 
#> -28.6493  -7.4321   0.1725   7.9982  27.5761 
#> 
#> Coefficients:
#>              Estimate Std. Error t value Pr(>|t|)    
#> (Intercept) 39.786970   2.332452  17.058   <2e-16 ***
#> treatment1  -0.008569   1.344504  -0.006    0.995    
#> treatment2  -0.358047   1.695764  -0.211    0.833    
#> recruitment -0.006927   0.004358  -1.589    0.113    
#> ---
#> Signif. codes:  0 '***' 0.001 '**' 0.01 '*' 0.05 '.' 0.1 ' ' 1
#> 
#> Residual standard error: 10.6 on 311 degrees of freedom
#> Multiple R-squared:  0.01023,    Adjusted R-squared:  0.000682 
#> F-statistic: 1.071 on 3 and 311 DF,  p-value: 0.3614
```

# 4 Figure 13

```
per2_start <- max(data_case_study[data_case_study$period==1,]$recruitment)

ggplot(data_case_study) +
  geom_point(aes(recruitment, response_visit_1, color = treatment)) +
  geom_line(aes(recruitment, fitted(test_trend), color = treatment), size = 1) +
  labs(x = "Trial duration in days", y = "PSPRS score at baseline", color = "Treatment:") +
  scale_color_viridis(discrete = T) +
  geom_vline(xintercept = 90*c(0:9), linetype = "dashed", color = "gray40") +
  geom_vline(xintercept = per2_start, linetype = "dashed", color = "darkred", size = 1) +
  theme_bw() +
  theme(legend.position = "bottom")
```

# 5 Analysis approaches

## 5.1 Regression model - period adjustment

```
# Regression model - period adjustment
mod_lm_per <- lm(response_visit_1 ~ treatment + period, data_case_study)
summary(mod_lm_per)
#> 
#> Call:
#> lm(formula = response_visit_1 ~ treatment + period, data = data_case_study)
#> 
#> Residuals:
#>      Min       1Q   Median       3Q      Max 
#> -27.9700  -7.6277   0.0616   8.0190  27.4062 
#> 
#> Coefficients:
#>             Estimate Std. Error t value Pr(>|t|)    
#> (Intercept) 38.81247    2.30429  16.844   <2e-16 ***
#> treatment1   0.02766    1.34673   0.021    0.984    
#> treatment2  -0.47708    1.72883  -0.276    0.783    
#> period      -1.55035    1.34978  -1.149    0.252    
#> ---
#> Signif. codes:  0 '***' 0.001 '**' 0.01 '*' 0.05 '.' 0.1 ' ' 1
#> 
#> Residual standard error: 10.62 on 311 degrees of freedom
#> Multiple R-squared:  0.006404,   Adjusted R-squared:  -0.00318 
#> F-statistic: 0.6682 on 3 and 311 DF,  p-value: 0.5721
```

```
mod_lm_per_est <- summary(mod_lm_per)$coefficients["treatment2", "Estimate"]
mod_lm_per_se <- summary(mod_lm_per)$coefficients["treatment2", "Std. Error"]
mod_lm_per_pval <- summary(mod_lm_per)$coefficients["treatment2", "Pr(>|t|)"]
```

## 5.2 Regression model - calendar time adjustment

```
# Regression model - calendar time adjustment
mod_lm_cal <- lm(response_visit_1 ~ treatment + cal_time, data_case_study)
summary(mod_lm_cal)
#> 
#> Call:
#> lm(formula = response_visit_1 ~ treatment + cal_time, data = data_case_study)
#> 
#> Residuals:
#>      Min       1Q   Median       3Q      Max 
#> -28.9888  -7.0507   0.3178   7.8311  26.7973 
#> 
#> Coefficients:
#>              Estimate Std. Error t value Pr(>|t|)    
#> (Intercept) 42.589184   7.575933   5.622 4.28e-08 ***
#> treatment1  -0.007604   1.364498  -0.006    0.996    
#> treatment2  -0.922229   1.649923  -0.559    0.577    
#> cal_time2   -0.939717   8.948747  -0.105    0.916    
#> cal_time3   -9.139326   7.937254  -1.151    0.250    
#> cal_time4   -8.511960   7.794644  -1.092    0.276    
#> cal_time5   -6.111671   7.714756  -0.792    0.429    
#> cal_time6   -5.743061   7.685398  -0.747    0.455    
#> cal_time7   -5.856114   7.633175  -0.767    0.444    
#> cal_time8   -5.823379   7.677450  -0.759    0.449    
#> cal_time9   -4.461112   8.928585  -0.500    0.618    
#> ---
#> Signif. codes:  0 '***' 0.001 '**' 0.01 '*' 0.05 '.' 0.1 ' ' 1
#> 
#> Residual standard error: 10.67 on 304 degrees of freedom
#> Multiple R-squared:  0.01905,    Adjusted R-squared:  -0.01322 
#> F-statistic: 0.5904 on 10 and 304 DF,  p-value: 0.8216
```

```
mod_lm_cal_est <- summary(mod_lm_cal)$coefficients["treatment2", "Estimate"]
mod_lm_cal_se <- summary(mod_lm_cal)$coefficients["treatment2", "Std. Error"]
mod_lm_cal_pval <- summary(mod_lm_cal)$coefficients["treatment2", "Pr(>|t|)"]
```

## 5.3 Mixed model - calendar time adjustment

```
# Mixed model - calendar time adjustment
mod_mix_cal <- lmer(response_visit_1 ~ treatment + (1 | cal_time), data_case_study)
summary(mod_mix_cal)
#> Linear mixed model fit by REML. t-tests use Satterthwaite's method [
#> lmerModLmerTest]
#> Formula: response_visit_1 ~ treatment + (1 | cal_time)
#>    Data: data_case_study
#> 
#> REML criterion at convergence: 2373.8
#> 
#> Scaled residuals: 
#>      Min       1Q   Median       3Q      Max 
#> -2.70365 -0.71805  0.03123  0.76417  2.57992 
#> 
#> Random effects:
#>  Groups   Name        Variance Std.Dev.
#>  cal_time (Intercept)   0.0     0.00   
#>  Residual             112.8    10.62   
#> Number of obs: 315, groups:  cal_time, 9
#> 
#> Fixed effects:
#>              Estimate Std. Error        df t value Pr(>|t|)    
#> (Intercept)  36.40502    0.95784 312.00000  38.008   <2e-16 ***
#> treatment1    0.08498    1.34650 312.00000   0.063    0.950    
#> treatment2   -1.17032    1.62088 312.00000  -0.722    0.471    
#> ---
#> Signif. codes:  0 '***' 0.001 '**' 0.01 '*' 0.05 '.' 0.1 ' ' 1
#> 
#> Correlation of Fixed Effects:
#>            (Intr) trtmn1
#> treatment1 -0.711       
#> treatment2 -0.591  0.420
#> optimizer (nloptwrap) convergence code: 0 (OK)
#> boundary (singular) fit: see help('isSingular')
```

```
mod_mix_cal_est <- summary(mod_mix_cal)$coefficients["treatment2", "Estimate"]
mod_mix_cal_se <- summary(mod_mix_cal)$coefficients["treatment2", "Std. Error"]
mod_mix_cal_pval <- summary(mod_mix_cal)$coefficients["treatment2", "Pr(>|t|)"]
```

## 5.4 Mixed model (AR1) - calendar time adjustment

```
# Mixed model - calendar time adjustment
mod_mix_ar1_cal <- fitme(response_visit_1 ~ treatment + AR1(1 | cal_time), data_case_study)

summary.HLfit(mod_mix_ar1_cal)
#> formula: response_visit_1 ~ treatment + AR1(1 | cal_time)
#> ML: Estimation of corrPars, lambda and phi by ML.
#>     Estimation of fixed effects by ML.
#> Estimation of lambda and phi by 'outer' ML, maximizing logL.
#> family: gaussian( link = identity ) 
#>  ------------ Fixed effects (beta) ------------
#>             Estimate Cond. SE  t-value
#> (Intercept) 36.40477   0.9537 38.17397
#> treatment1   0.08495   1.3401  0.06339
#> treatment2  -1.17017   1.6131 -0.72540
#>  --------------- Random effects ---------------
#> Family: gaussian( link = identity ) 
#>                    --- Correlation parameters:
#>   1.ARphi 
#> 0.3433296 
#>            --- Variance parameters ('lambda'):
#> lambda = var(u) for u ~ Gaussian; 
#>    cal_time  :  0.002235  
#> # of obs: 315; # of groups: cal_time, 9 
#>  -------------- Residual variance  ------------
#> phi estimate was 111.771 
#>  ------------- Likelihood values  -------------
#>                         logLik
#> logL       (p_v(h)): -1189.809
```

```
res <- summary.HLfit(mod_mix_ar1_cal, verbose = FALSE)

IC <- get_any_IC(mod_mix_ar1_cal, verbose = FALSE)
IC
#>        marginal AIC:     conditional AIC:      dispersion AIC: 
#>            2391.6189            2387.6187            2385.6189 
#>        effective df: 
#>             311.9958
```

```
eff_df <- IC["       effective df:"] # effective degrees of freedom

2*(1-pt(abs(res$beta_table["treatment2", "t-value"]), eff_df)) # p-value
#> [1] 0.4687523
```

```
mod_mix_ar1_cal_est <- summary(mod_mix_ar1_cal, verbose = F)$beta_table["treatment2", "Estimate"]
mod_mix_ar1_cal_se <- summary(mod_mix_ar1_cal, verbose = F)$beta_table["treatment2", "Cond. SE"]
mod_mix_ar1_cal_pval <- 2*(1-pt(abs(res$beta_table["treatment2", "t-value"]), eff_df))
```

## 5.5 Spline regression - period adjustment

```
# Spline regression - period adjustment
per2_start <- max(data_case_study[data_case_study$period==1,]$recruitment)

mod_splines_per <- lm(response_visit_1 ~ treatment + bs(recruitment, knots = per2_start, degree = 3), data_case_study)
summary(mod_splines_per)
#> 
#> Call:
#> lm(formula = response_visit_1 ~ treatment + bs(recruitment, knots = per2_start, 
#>     degree = 3), data = data_case_study)
#> 
#> Residuals:
#>      Min       1Q   Median       3Q      Max 
#> -27.6957  -7.4862  -0.0262   7.9596  27.0661 
#> 
#> Coefficients:
#>                                                   Estimate Std. Error t value
#> (Intercept)                                       43.93461    6.81961   6.442
#> treatment1                                        -0.09562    1.35127  -0.071
#> treatment2                                        -0.68341    1.71679  -0.398
#> bs(recruitment, knots = per2_start, degree = 3)1  -3.64694   11.08940  -0.329
#> bs(recruitment, knots = per2_start, degree = 3)2 -11.62174    6.64485  -1.749
#> bs(recruitment, knots = per2_start, degree = 3)3  -6.19509    8.12920  -0.762
#> bs(recruitment, knots = per2_start, degree = 3)4  -9.65718    7.06458  -1.367
#>                                                  Pr(>|t|)    
#> (Intercept)                                      4.52e-10 ***
#> treatment1                                         0.9436    
#> treatment2                                         0.6909    
#> bs(recruitment, knots = per2_start, degree = 3)1   0.7425    
#> bs(recruitment, knots = per2_start, degree = 3)2   0.0813 .  
#> bs(recruitment, knots = per2_start, degree = 3)3   0.4466    
#> bs(recruitment, knots = per2_start, degree = 3)4   0.1726    
#> ---
#> Signif. codes:  0 '***' 0.001 '**' 0.01 '*' 0.05 '.' 0.1 ' ' 1
#> 
#> Residual standard error: 10.61 on 308 degrees of freedom
#> Multiple R-squared:  0.0171, Adjusted R-squared:  -0.002051 
#> F-statistic: 0.8929 on 6 and 308 DF,  p-value: 0.5003
```

```
mod_splines_per_est <- summary(mod_splines_per)$coefficients["treatment2", "Estimate"]
mod_splines_per_se <- summary(mod_splines_per)$coefficients["treatment2", "Std. Error"]
mod_splines_per_pval <- summary(mod_splines_per)$coefficients["treatment2", "Pr(>|t|)"]
```

## 5.6 Spline regression - calendar time adjustment

```
# Spline regression - calendar time adjustment
cal_time_start <- c()
for (i in unique(data_case_study$cal_time)) {
  cal_time_start <- c(cal_time_start, max(data_case_study[data_case_study$cal_time==i,]$recruitment))
}

cal_time_start <- cal_time_start[-length(cal_time_start)]

mod_splines_cal <- lm(response_visit_1 ~ treatment + bs(recruitment, knots = cal_time_start, degree = 3), data_case_study)
summary(mod_splines_cal)
#> 
#> Call:
#> lm(formula = response_visit_1 ~ treatment + bs(recruitment, knots = cal_time_start, 
#>     degree = 3), data = data_case_study)
#> 
#> Residuals:
#>      Min       1Q   Median       3Q      Max 
#> -28.0959  -7.5474   0.0984   8.3199  26.8806 
#> 
#> Coefficients: (1 not defined because of singularities)
#>                                                       Estimate Std. Error
#> (Intercept)                                            46.7726     7.5397
#> treatment1                                             -0.1678     1.3654
#> treatment2                                             -0.4718     1.7345
#> bs(recruitment, knots = cal_time_start, degree = 3)1   -8.5930    11.0737
#> bs(recruitment, knots = cal_time_start, degree = 3)2   -9.1646     7.7771
#> bs(recruitment, knots = cal_time_start, degree = 3)3  -12.8293     8.7615
#> bs(recruitment, knots = cal_time_start, degree = 3)4   -8.8127     7.7043
#> bs(recruitment, knots = cal_time_start, degree = 3)5  -17.0795     9.5498
#> bs(recruitment, knots = cal_time_start, degree = 3)6  -12.1660    17.7100
#> bs(recruitment, knots = cal_time_start, degree = 3)7   -5.2338    20.3361
#> bs(recruitment, knots = cal_time_start, degree = 3)8  -26.9448    32.7349
#> bs(recruitment, knots = cal_time_start, degree = 3)9    7.3511    22.7286
#> bs(recruitment, knots = cal_time_start, degree = 3)10 -19.5005    13.0572
#> bs(recruitment, knots = cal_time_start, degree = 3)11       NA         NA
#>                                                       t value Pr(>|t|)    
#> (Intercept)                                             6.203 1.82e-09 ***
#> treatment1                                             -0.123   0.9023    
#> treatment2                                             -0.272   0.7858    
#> bs(recruitment, knots = cal_time_start, degree = 3)1   -0.776   0.4384    
#> bs(recruitment, knots = cal_time_start, degree = 3)2   -1.178   0.2396    
#> bs(recruitment, knots = cal_time_start, degree = 3)3   -1.464   0.1442    
#> bs(recruitment, knots = cal_time_start, degree = 3)4   -1.144   0.2536    
#> bs(recruitment, knots = cal_time_start, degree = 3)5   -1.788   0.0747 .  
#> bs(recruitment, knots = cal_time_start, degree = 3)6   -0.687   0.4926    
#> bs(recruitment, knots = cal_time_start, degree = 3)7   -0.257   0.7971    
#> bs(recruitment, knots = cal_time_start, degree = 3)8   -0.823   0.4111    
#> bs(recruitment, knots = cal_time_start, degree = 3)9    0.323   0.7466    
#> bs(recruitment, knots = cal_time_start, degree = 3)10  -1.493   0.1364    
#> bs(recruitment, knots = cal_time_start, degree = 3)11      NA       NA    
#> ---
#> Signif. codes:  0 '***' 0.001 '**' 0.01 '*' 0.05 '.' 0.1 ' ' 1
#> 
#> Residual standard error: 10.67 on 302 degrees of freedom
#> Multiple R-squared:  0.02618,    Adjusted R-squared:  -0.01252 
#> F-statistic: 0.6765 on 12 and 302 DF,  p-value: 0.7739
```

```
mod_splines_cal_est <- summary(mod_splines_cal)$coefficients["treatment2", "Estimate"]
mod_splines_cal_se <- summary(mod_splines_cal)$coefficients["treatment2", "Std. Error"]
mod_splines_cal_pval <- summary(mod_splines_cal)$coefficients["treatment2", "Pr(>|t|)"]
```

## 5.7 Pooled analysis

```
# Pooled analysis
mod_pool <- lm(response_visit_1 ~ treatment, data_case_study %>% filter(treatment %in% c(0, 2)))
summary(mod_pool)
#> 
#> Call:
#> lm(formula = response_visit_1 ~ treatment, data = data_case_study %>% 
#>     filter(treatment %in% c(0, 2)))
#> 
#> Residuals:
#>      Min       1Q   Median       3Q      Max 
#> -27.1110  -7.8935  -0.2381   8.2338  27.4062 
#> 
#> Coefficients:
#>             Estimate Std. Error t value Pr(>|t|)    
#> (Intercept)  36.4050     0.9812  37.102   <2e-16 ***
#> treatment2   -1.1703     1.6604  -0.705    0.482    
#> ---
#> Signif. codes:  0 '***' 0.001 '**' 0.01 '*' 0.05 '.' 0.1 ' ' 1
#> 
#> Residual standard error: 10.88 on 187 degrees of freedom
#> Multiple R-squared:  0.00265,    Adjusted R-squared:  -0.002684 
#> F-statistic: 0.4968 on 1 and 187 DF,  p-value: 0.4818
```

```
mod_pool_est <- summary(mod_pool)$coefficients["treatment2", "Estimate"]
mod_pool_se <- summary(mod_pool)$coefficients["treatment2", "Std. Error"]
mod_pool_pval <- summary(mod_pool)$coefficients["treatment2", "Pr(>|t|)"]
```

## 5.8 Separate analysis

```
# Separate analysis
mod_sep <- lm(response_visit_1 ~ treatment, data_case_study %>% filter(treatment %in% c(0, 2), period==2))
summary(mod_sep)
#> 
#> Call:
#> lm(formula = response_visit_1 ~ treatment, data = data_case_study %>% 
#>     filter(treatment %in% c(0, 2), period == 2))
#> 
#> Residuals:
#>      Min       1Q   Median       3Q      Max 
#> -27.1110  -7.6332  -0.2785   7.7252  27.4062 
#> 
#> Coefficients:
#>             Estimate Std. Error t value Pr(>|t|)    
#> (Intercept)  35.6482     1.3473  26.459   <2e-16 ***
#> treatment2   -0.4135     1.9197  -0.215     0.83    
#> ---
#> Signif. codes:  0 '***' 0.001 '**' 0.01 '*' 0.05 '.' 0.1 ' ' 1
#> 
#> Residual standard error: 11.11 on 132 degrees of freedom
#> Multiple R-squared:  0.0003513,  Adjusted R-squared:  -0.007222 
#> F-statistic: 0.04639 on 1 and 132 DF,  p-value: 0.8298
```

```
mod_sep_est <- summary(mod_sep)$coefficients["treatment2", "Estimate"]
mod_sep_se <- summary(mod_sep)$coefficients["treatment2", "Std. Error"]
mod_sep_pval <- summary(mod_sep)$coefficients["treatment2", "Pr(>|t|)"]
```

# 6 TABLE 2

```
table_2 <- data.frame(`Analysis approach` = c("Fixed effect model", "Fixed effect model", "Mixed model", 
                                              "Mixed model (AR1)", "Spline regression", "Spline regression", "Pooled analysis", "Separate analysis"),
                      Adjustment = c("Periods", rep("Calendar time units", 3), "Periods", "Calendar time units", rep("-", 2)),
                      `Effect estimate` = round(c(mod_lm_per_est, mod_lm_cal_est, mod_mix_cal_est, mod_mix_ar1_cal_est, mod_splines_per_est, mod_splines_cal_est, mod_pool_est, mod_sep_est), 3),
                      `Std. error` = round(c(mod_lm_per_se, mod_lm_cal_se, mod_mix_cal_se, mod_mix_ar1_cal_se, mod_splines_per_se, mod_splines_cal_se, mod_pool_se, mod_sep_se), 3),
                      `p-value` = round(c(mod_lm_per_pval, mod_lm_cal_pval, mod_mix_cal_pval, mod_mix_ar1_cal_pval, mod_splines_per_pval, mod_splines_cal_pval, mod_pool_pval, mod_sep_pval), 3),
                      
                      check.names = F)

kable(table_2, booktabs = T) %>%
  kable_styling(bootstrap_options = "striped")
```

| Analysis approach | Adjustment | Effect estimate | Std. error | p-value |
| --- | --- | --- | --- | --- |
| Fixed effect model | Periods | -0.477 | 1.729 | 0.783 |
| Fixed effect model | Calendar time units | -0.922 | 1.650 | 0.577 |
| Mixed model | Calendar time units | -1.170 | 1.621 | 0.471 |
| Mixed model (AR1) | Calendar time units | -1.170 | 1.613 | 0.469 |
| Spline regression | Periods | -0.683 | 1.717 | 0.691 |
| Spline regression | Calendar time units | -0.472 | 1.735 | 0.786 |
| Pooled analysis |  | -1.170 | 1.660 | 0.482 |
| Separate analysis |  | -0.413 | 1.920 | 0.830 |
