# Supplementary material for: Statistical Modeling to Adjust for Time Trends in Adaptive Platform Trials Utilizing Non‐Concurrent Controls
Source: Biom J. 2025 Jun 10;67(3):e70059. doi: 10.1002/bimj.70059 (PMC12150008; doi:10.1002/bimj.70059)
Supplement: Supplementary file 1 — Supporting Information [file BIMJ-67-e70059-s002.zip › simulations/figures/splines_alpha_pow_d_trend.pdf]

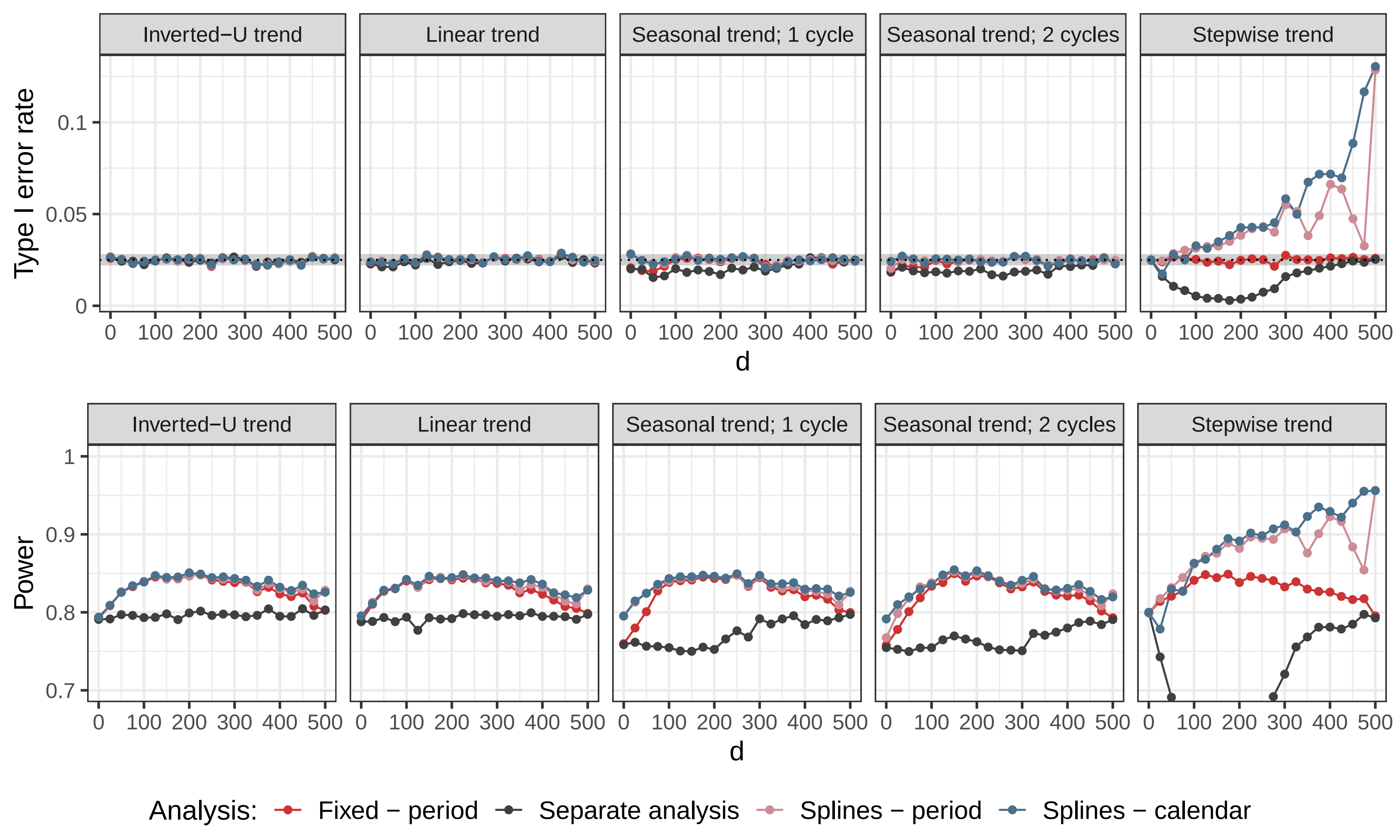

Inverted−U trend
Linear trend
Seasonal trend; 1 cycle
Seasonal trend; 2 cycles
Stepwise trend
Type I error rate
0.1
0.05
0
0
100
200
300
400
500
d
Power
1
0.9
0.8
0.7
Analysis:
Fixed − period
Separate analysis
Splines − period
Splines − calendar
