# Supplementary material for: Statistical Modeling to Adjust for Time Trends in Adaptive Platform Trials Utilizing Non‐Concurrent Controls
Source: Biom J. 2025 Jun 10;67(3):e70059. doi: 10.1002/bimj.70059 (PMC12150008; doi:10.1002/bimj.70059)
Supplement: Supplementary file 1 — Supporting Information [file BIMJ-67-e70059-s002.zip › simulations/NCC_FreqModels_figures.html]

Statistical modeling to adjust for time trends in adaptive platform trials utilizing non-concurrent controls


Code 

- Show All Code
- Hide All Code

# Statistical modeling to adjust for time trends in adaptive platform trials utilizing non-concurrent controls

### Data only until the evaluated arm leaves the trial

#### Pavla Krotka, Martin Posch, Mohamed Gewily, Günter Höglinger, Marta Bofill Roig

#### 2024

```
# Load in all files from "results"

csv_names <- list.files("results/", pattern="*.csv")

for (i in 1:length(csv_names)){
  assign(str_extract(csv_names[i], "^([^\\.])+"), read_csv(paste0("results/", csv_names[i])) %>%
           mutate(trend = ifelse(trend=="seasonal" & "n_wave" %in% names(.), ifelse(n_wave==1, "seasonal_1", "seasonal_2"), trend)))
}
```

```
# Names for trend patterns
trend_names <- c(linear = "Linear trend", stepwise_2 = "Stepwise trend", inv_u = "Inverted-U trend", seasonal_1 = "Seasonal trend; 1 cycle", seasonal_2 = "Seasonal trend; 2 cycles")

# Names for splines degrees
degree_names <- c(`1` = "Linear splines", `2` = "Quadratic splines", `3` = "Cubic splines")

# Names for trend casees
trend_case_names <- c(eq = paste("{{lambda[0]==lambda[1]}=={lambda[2]==lambda[3]}}==lambda[4]"), 
                      diff_1 = paste("{{lambda[0]==lambda[2]}=={lambda[3]==lambda[4]}}==0"), 
                      diff_12 = paste("{lambda[0]==lambda[3]}=={lambda[4]==0}~~lambda[1]==lambda[2]"), 
                      diff_124 = paste("{lambda[0]==lambda[3]}==0~~{lambda[1]==lambda[2]}==lambda[4]"),
                      diff_124_add = paste("{lambda[0]==lambda[3]}==0~~{lambda[2]==2*lambda[1]}~~{lambda[4]==3*lambda[1]}"))

# Color palette
my_palette = c("fixmodel" = "brown3",
               "fixmodel_cal" = "dodgerblue3",
               "poolmodel" = "mediumseagreen",
               "sepmodel" = "gray25",
               "mixmodel" = "deepskyblue2",
               "mixmodel_cal" = "goldenrod2",
               "mixmodel_AR1" = "lightsalmon4",
               "mixmodel_AR1_cal" = "mediumorchid3",
               "mixmodel_int" = "blue3",
               "mixmodel_int_cal" = "tan1",
               "splines" = "lightpink3",
               "splines_cal" = "skyblue4")

#show_col(my_palette)

# Prediction interval for T1E
p <- 0.025
alpha <- 0.05
nsim <- 10000
sd <- sqrt(p*(1-p)/nsim)
z.alpha <- qnorm(1-alpha/2,0,1)
pred_int <- c(p-z.alpha*sd,p+z.alpha*sd)
#pred_int
```

# Introduction

This file contains all code to reproduce the figures presented in
Section 4 and the Supplementary material of the paper *“Statistical
modeling to adjust for time trends in adaptive platform trials utilizing
non-concurrent controls”* by Pavla Krotka, Martin Posch, Mohamed
Gewily, Günter Höglinger, and Marta Bofill Roig.

# Figure 3 - Time trend patterns

```
# Time trend patterns

data_lin <- datasim_cont(num_arms = 4, n_arm = 250, d = 250*c(0:3), theta=rep(0, 4), lambda = rep(0.15, 5), sigma = 1, trend = "linear", full = T)$Data

data_step <- datasim_cont(num_arms = 4, n_arm = 250, d = 250*c(0:3), theta=rep(0, 4), lambda = rep(0.15, 5), sigma = 1, trend = "stepwise_2", full = T)$Data

data_inv_u_pos <- datasim_cont(num_arms = 4, n_arm = 250, d = 250*c(0:3), theta=rep(0, 4), lambda = rep(0.15, 5), sigma = 1, trend = "inv_u", N_peak = 764, full = T)$Data

data_seasonal_1 <- datasim_cont(num_arms = 4, n_arm = 250, d = 250*c(0:3), theta=rep(0, 4), lambda = rep(0.15, 5), sigma = 1, trend = "seasonal", n_wave = 1, full = T)$Data

data_seasonal_2 <- datasim_cont(num_arms = 4, n_arm = 250, d = 250*c(0:3), theta=rep(0, 4), lambda = rep(0.15, 5), sigma = 1, trend = "seasonal", n_wave = 2, full = T)$Data

data_trend <- rbind(data_lin %>% mutate(shape = "Linear trend"),
                    data_step %>% mutate(shape = "Stepwise trend"),
                    data_inv_u_pos %>% mutate(shape = "Inverted-U trend"),
                    data_seasonal_1 %>% mutate(shape = "Seasonal trend; 1 cycle"),
                    data_seasonal_2 %>% mutate(shape = "Seasonal trend; 2 cycles"))

ggplot(data_trend) +
  geom_point(aes(x=j, y=means), color="darkred") +
  theme_bw(base_size = 13.8) +
  facet_grid(.~shape) +
  labs(x="Patient recruitment", y=TeX("Mean response under $H_0$")) +
  scale_y_continuous(breaks = seq(-0.15, 0.45, by=0.15), labels = seq(-0.15, 0.45, by=0.15))
```

```
ggsave("figures/trend_patterns.png", width = 10, height = 4)
ggsave("figures/trend_patterns.pdf", width = 10, height = 4)
ggsave("figures/trend_patterns.tiff", width = 10, height = 4)
```

# SETTING 1 - 10 treatment arms, equal time trends

## Plots from Section 4

### Figure 4

- Evaluated arm: 5
- \(d=400\)
- \(n=250\) per experimental
  treatment arm
- T1E and power with respect to the strength of the time trend \(\lambda\)

```
ggarrange(ggplot(results_setting_1_alpha %>% filter(study_arm==5 & d2==400)) +
            annotate("rect", xmin = -Inf, xmax = Inf, ymin = pred_int[1], ymax = pred_int[2], fill = "gray54", alpha = 0.4) +
            geom_line(aes(lambda0, reject_h0, color=model)) +
            geom_point(aes(lambda0, reject_h0, color=model)) +
            labs(x=TeX("$\\lambda$"), y="Type I error rate", color="Analysis approach:") +
            geom_hline(aes(yintercept = 0.025), linetype = "dotted") +
            facet_grid(~ trend,
                       labeller = labeller(trend  = as_labeller(trend_names))) +
            scale_x_continuous(labels = function(x) sub(".0+$", "", x)) +
            scale_y_continuous(labels = function(x) sub(".0+$", "", x)) +
            #coord_cartesian(ylim = c(0, 0.3)) +
            scale_color_manual(labels = c("Fixed - period", "Pooled analysis", "Separate analysis"), values = my_palette, limits = force) +
            theme_bw(base_size = 13.8) +
            theme(legend.text=element_text(size = 13)),
          
          ggplot(results_setting_1_pow %>% filter(study_arm==5 & d2==400)) +
            geom_line(aes(lambda0, reject_h0, color=model)) +
            geom_point(aes(lambda0, reject_h0, color=model)) +
            labs(x=TeX("$\\lambda$"), y="Power", color="Analysis approach:") +
            facet_grid(~ trend,
                       labeller = labeller(trend  = as_labeller(trend_names))) +
            scale_x_continuous(labels = function(x) sub(".0+$", "", x)) +
            scale_y_continuous(labels = function(x) sub(".0+$", "", x)) +
            coord_cartesian(ylim = c(0.70, 1)) +
            scale_color_manual(labels = c("Fixed - period", "Pooled analysis", "Separate analysis"), values = my_palette, limits = force) +
            theme_bw(base_size = 13.8) +
            theme(legend.text=element_text(size = 13)),
          
          common.legend = T, legend = "bottom") + 
  bgcolor("white") + 
  border("white")
```

```
ggsave("figures/fixmodel_alpha_pow_lambda.png", width = 10, height = 5)
ggsave("figures/fixmodel_alpha_pow_lambda.pdf", width = 10, height = 5)
ggsave("figures/fixmodel_alpha_pow_lambda.tiff", width = 10, height = 5)
```

### Figure 5

- Evaluated arm: 5
- \(\lambda=0.5\)
- \(n=250\) per experimental
  treatment arm
- T1E and power with respect to the timing of adding the treatment
  arms

```
ggarrange(ggplot(results_setting_1_alpha %>% filter(study_arm==5 & lambda0==0.5)) +
            annotate("rect", xmin = -Inf, xmax = Inf, ymin = pred_int[1], ymax = pred_int[2], fill = "gray54", alpha = 0.4) +
            geom_line(aes(d2, reject_h0, color=model)) +
            geom_point(aes(d2, reject_h0, color=model)) +
            labs(x=TeX("$d$"), y="Type I error rate", color="Analysis approach:") +
            geom_hline(aes(yintercept = 0.025), linetype = "dotted") +
            facet_grid(~ trend,
                       labeller = labeller(trend  = as_labeller(trend_names))) +
            scale_y_continuous(labels = function(x) sub(".0+$", "", x)) +
            #coord_cartesian(ylim = c(0, 0.3)) +
            scale_color_manual(labels = c("Fixed - period", "Pooled analysis", "Separate analysis"), values = my_palette, limits = force) +
            theme_bw(base_size = 13.8) +
            theme(legend.text=element_text(size = 13)),
          
          ggplot(results_setting_1_pow %>% filter(study_arm==5 & lambda0==0.5)) +
            geom_line(aes(d2, reject_h0, color=model)) +
            geom_point(aes(d2, reject_h0, color=model)) +
            labs(x=TeX("$d$"), y="Power", color="Analysis approach:") +
            facet_grid(~ trend,
                       labeller = labeller(trend  = as_labeller(trend_names))) +
            scale_y_continuous(labels = function(x) sub(".0+$", "", x)) +
            coord_cartesian(ylim = c(0.70, 1)) +
            scale_color_manual(labels = c("Fixed - period", "Pooled analysis", "Separate analysis"), values = my_palette, limits = force) +
            theme_bw(base_size = 13.8) +
            theme(legend.text=element_text(size = 13)),
          
          common.legend = T, legend = "bottom") + 
  bgcolor("white") + 
  border("white")
```

```
ggsave("figures/fixmodel_alpha_pow_d.png", width = 10, height = 5)
ggsave("figures/fixmodel_alpha_pow_d.pdf", width = 10, height = 5)
ggsave("figures/fixmodel_alpha_pow_d.tiff", width = 10, height = 5)
```

### Figure 6

- Evaluated arm: 5
- \(\lambda=0.5\)
- Degree of B-spline: 3 (cubic)
- Calendar time unit size: 450
- \(n=250\) per experimental
  treatment arm
- \(N\_p\) for inverted-U time trend
  placed to the middle of the trial
- T1E and power with respect to the timing of adding the treatment
  arms under different time trend patterns

```
ggarrange(ggplot(results_setting_1_splines_alpha %>% filter(study_arm==5 & lambda0==0.5 & unit_size==450 & bs_degree==3)) +
            annotate("rect", xmin = -Inf, xmax = Inf, ymin = pred_int[1], ymax = pred_int[2], fill = "gray54", alpha = 0.4) +
            geom_line(aes(d2, reject_h0, color=model)) +
            geom_point(aes(d2, reject_h0, color=model)) +
            labs(x="d", y="Type I error rate", color="Analysis:") +
            geom_hline(aes(yintercept = 0.025), linetype = "dotted") +
            facet_grid( ~ trend,
                        labeller = labeller(trend  = as_labeller(trend_names))) +
            #scale_x_continuous(labels = function(x) sub(".0+$", "", x)) +
            scale_y_continuous(labels = function(x) sub(".0+$", "", x)) +
            #coord_cartesian(ylim = c(0, 0.05)) +
            scale_color_manual(labels = c("Fixed - period", "Separate analysis", "Splines - period", "Splines - calendar"), values = my_palette, limits = force) +
            theme_bw(base_size = 13.8) +
            theme(legend.text=element_text(size = 13)),
          
          ggplot(results_setting_1_splines_pow %>% filter(study_arm==5 & lambda0==0.5 & unit_size==450 & bs_degree==3)) +
            geom_line(aes(d2, reject_h0, color=model)) +
            geom_point(aes(d2, reject_h0, color=model)) +
            labs(x="d", y="Power", color="Analysis:") +
            facet_grid( ~ trend,
                        labeller = labeller(trend  = as_labeller(trend_names))) +
            #scale_x_continuous(labels = function(x) sub(".0+$", "", x)) +
            scale_y_continuous(labels = function(x) sub(".0+$", "", x)) +
            coord_cartesian(ylim = c(0.70, 1)) +
            scale_color_manual(labels = c("Fixed - period", "Separate analysis", "Splines - period", "Splines - calendar"), values = my_palette, limits = force) +
            theme_bw(base_size = 13.8) +
            theme(legend.text=element_text(size = 13)),
          
          common.legend = T, legend = "bottom", ncol = 1) + 
  bgcolor("white") + 
  border("white")
```

```
ggsave("figures/splines_alpha_pow_d_trend.png", width = 10, height = 6)
ggsave("figures/splines_alpha_pow_d_trend.pdf", width = 10, height = 6)
ggsave("figures/splines_alpha_pow_d_trend.tiff", width = 10, height = 6)
```

### Figure 7

- Evaluated arm: 5
- \(d=500\)
- Degree of B-spline: 3 (cubic)
- Calendar time unit size: 450
- \(n=250\) per experimental
  treatment arm
- \(N\_p=2500\) for inverted-U time
  trend (corresponding to the middle of the trial)
- T1E and power with respect to the strength of the time trend \(\lambda\) under different time trend
  patterns

```
ggarrange(ggplot(results_setting_1_splines_alpha %>% filter(study_arm==5 & d2==500 & unit_size==450 & bs_degree==3)) +
            annotate("rect", xmin = -Inf, xmax = Inf, ymin = pred_int[1], ymax = pred_int[2], fill = "gray54", alpha = 0.4) +
            geom_line(aes(lambda0, reject_h0, color=model)) +
            geom_point(aes(lambda0, reject_h0, color=model)) +
            labs(x=TeX("$\\lambda$"), y="Type I error rate", color="Analysis:") +
            geom_hline(aes(yintercept = 0.025), linetype = "dotted") +
            facet_grid( ~ trend,
                        labeller = labeller(trend  = as_labeller(trend_names))) +
            scale_x_continuous(labels = function(x) sub(".0+$", "", x)) +
            scale_y_continuous(labels = function(x) sub(".0+$", "", x)) +
            coord_cartesian(ylim = c(0, 0.05)) +
            scale_color_manual(labels = c("Fixed - period", "Separate analysis", "Splines - period", "Splines - calendar"), values = my_palette, limits = force) +
            theme_bw(base_size = 13.8) +
            theme(legend.text=element_text(size = 13)),
          
          ggplot(results_setting_1_splines_pow %>% filter(study_arm==5 & d2==500 & unit_size==450 & bs_degree==3)) +
            geom_line(aes(lambda0, reject_h0, color=model)) +
            geom_point(aes(lambda0, reject_h0, color=model)) +
            labs(x=TeX("$\\lambda$"), y="Power", color="Analysis:") +
            facet_grid( ~ trend,
                        labeller = labeller(trend  = as_labeller(trend_names))) +
            scale_x_continuous(labels = function(x) sub(".0+$", "", x)) +
            scale_y_continuous(labels = function(x) sub(".0+$", "", x)) +
            coord_cartesian(ylim = c(0.70, 1)) +
            scale_color_manual(labels = c("Fixed - period", "Separate analysis", "Splines - period", "Splines - calendar"), values = my_palette, limits = force) +
            theme_bw(base_size = 13.8) +
            theme(legend.text=element_text(size = 13)),
          
          common.legend = T, legend = "bottom", ncol = 1) + 
  bgcolor("white") + 
  border("white")
```

```
ggsave("figures/splines_alpha_pow_lambda_trend.png", width = 10, height = 6)
ggsave("figures/splines_alpha_pow_lambda_trend.pdf", width = 10, height = 6)
ggsave("figures/splines_alpha_pow_lambda_trend.tiff", width = 10, height = 6)
```

## Plots from the Supplementary Material

### Figure S1

- \(d=400\)
- \(n=250\) per experimental
  treatment arm
- Rows - Evaluated arm
- T1E and power with respect to the strength of the time trend \(\lambda\)

```
ggarrange(ggplot(results_setting_1_alpha %>% filter(d2==400)) +
            annotate("rect", xmin = -Inf, xmax = Inf, ymin = pred_int[1], ymax = pred_int[2], fill = "gray54", alpha = 0.4) +
            geom_line(aes(lambda0, reject_h0, color=model)) +
            geom_point(aes(lambda0, reject_h0, color=model)) +
            labs(x=TeX("$\\lambda$"), y="Type I error rate", color="Analysis approach:") +
            geom_hline(aes(yintercept = 0.025), linetype = "dotted") +
            facet_grid(study_arm ~ trend,
                       labeller = labeller(trend  = as_labeller(trend_names))) +
            scale_x_continuous(labels = function(x) sub(".0+$", "", x)) +
            scale_y_continuous(labels = function(x) sub(".0+$", "", x)) +
            #coord_cartesian(ylim = c(0, 0.3)) +
            scale_color_manual(labels = c("Fixed - period", "Pooled analysis", "Separate analysis"), values = my_palette, limits = force) +
            theme_bw(base_size = 13.8) +
            theme(legend.text=element_text(size = 13)),
          
          ggplot(results_setting_1_pow %>% filter(d2==400)) +
            geom_line(aes(lambda0, reject_h0, color=model)) +
            geom_point(aes(lambda0, reject_h0, color=model)) +
            labs(x=TeX("$\\lambda$"), y="Power", color="Analysis approach:") +
            facet_grid(study_arm ~ trend,
                       labeller = labeller(trend  = as_labeller(trend_names))) +
            scale_x_continuous(labels = function(x) sub(".0+$", "", x)) +
            scale_y_continuous(labels = function(x) sub(".0+$", "", x)) +
            coord_cartesian(ylim = c(0.70, 1)) +
            scale_color_manual(labels = c("Fixed - period", "Pooled analysis", "Separate analysis"), values = my_palette, limits = force) +
            theme_bw(base_size = 13.8) +
            theme(legend.text=element_text(size = 13)),
          
          common.legend = T, legend = "bottom") + 
  bgcolor("white") + 
  border("white")
```

```
ggsave("figures/fixmodel_alpha_pow_lambda_all_arms.png", width = 10, height = 15)
ggsave("figures/fixmodel_alpha_pow_lambda_all_arms.pdf", width = 10, height = 15)
ggsave("figures/fixmodel_alpha_pow_lambda_all_arms.tiff", width = 10, height = 15)
```

### Figure S2

- \(\lambda=0.5\)
- \(n=250\) per experimental
  treatment arm
- Rows - Evaluated arm
- T1E and power with respect to the timing of adding the treatment
  arms

```
ggarrange(ggplot(results_setting_1_alpha %>% filter(lambda0==0.5)) +
            annotate("rect", xmin = -Inf, xmax = Inf, ymin = pred_int[1], ymax = pred_int[2], fill = "gray54", alpha = 0.4) +
            geom_line(aes(d2, reject_h0, color=model)) +
            geom_point(aes(d2, reject_h0, color=model)) +
            labs(x=TeX("$d$"), y="Type I error rate", color="Analysis approach:") +
            geom_hline(aes(yintercept = 0.025), linetype = "dotted") +
            facet_grid(study_arm ~ trend,
                       labeller = labeller(trend  = as_labeller(trend_names))) +
            scale_y_continuous(labels = function(x) sub(".0+$", "", x)) +
            # coord_cartesian(ylim = c(0, 0.3)) +
            scale_color_manual(labels = c("Fixed - period", "Pooled analysis", "Separate analysis"), values = my_palette, limits = force) +
            theme_bw(base_size = 13.8) +
            theme(legend.text=element_text(size = 13)),
          
          ggplot(results_setting_1_pow %>% filter(lambda0==0.5)) +
            geom_line(aes(d2, reject_h0, color=model)) +
            geom_point(aes(d2, reject_h0, color=model)) +
            labs(x=TeX("$d$"), y="Power", color="Analysis approach:") +
            facet_grid(study_arm ~ trend,
                       labeller = labeller(trend  = as_labeller(trend_names))) +
            scale_y_continuous(labels = function(x) sub(".0+$", "", x)) +
            coord_cartesian(ylim = c(0.70, 1)) +
            scale_color_manual(labels = c("Fixed - period", "Pooled analysis", "Separate analysis"), values = my_palette, limits = force) +
            theme_bw(base_size = 13.8) +
            theme(legend.text=element_text(size = 13)),
          
          common.legend = T, legend = "bottom") + 
  bgcolor("white") + 
  border("white")
```

```
ggsave("figures/fixmodel_alpha_pow_d_all_arms.png", width = 10, height = 15)
ggsave("figures/fixmodel_alpha_pow_d_all_arms.pdf", width = 10, height = 15)
ggsave("figures/fixmodel_alpha_pow_d_all_arms.tiff", width = 10, height = 15)
```

### Figure S3

- \(\lambda=0.5\)
- \(d=200\)
- \(n=250\) per experimental
  treatment arm
- T1E and power with respect to the index of the evaluated arm

```
ggarrange(ggplot(results_setting_1_alpha %>% filter(d2==200 & lambda0==0.5)) +
            annotate("rect", xmin = -Inf, xmax = Inf, ymin = pred_int[1], ymax = pred_int[2], fill = "gray54", alpha = 0.4) +
            geom_line(aes(study_arm, reject_h0, color=model)) +
            geom_point(aes(study_arm, reject_h0, color=model)) +
            labs(x="Study arm", y="Type I error rate", color="Analysis approach:") +
            geom_hline(aes(yintercept = 0.025), linetype = "dotted") +
            facet_grid(~ trend,
                       labeller = labeller(trend  = as_labeller(trend_names))) +
            #coord_cartesian(ylim = c(0, 0.3)) +
            scale_color_manual(labels = c("Fixed - period", "Pooled analysis", "Separate analysis"), values = my_palette, limits = force) +
            scale_x_continuous(breaks = c(1:10), labels = c(1:10)) +
            scale_y_continuous(labels = function(x) sub(".0+$", "", x)) +
            theme_bw(base_size = 13.8) +
            theme(legend.text=element_text(size = 13)),
          
          ggplot(results_setting_1_pow %>% filter(d2==200 & lambda0==0.5)) +
            geom_line(aes(study_arm, reject_h0, color=model)) +
            geom_point(aes(study_arm, reject_h0, color=model)) +
            labs(x="Study arm", y="Power", color="Analysis approach:") +
            facet_grid(~ trend,
                       labeller = labeller(trend  = as_labeller(trend_names))) +
            coord_cartesian(ylim = c(0.70, 1)) +
            scale_color_manual(labels = c("Fixed - period", "Pooled analysis", "Separate analysis"), values = my_palette, limits = force) +
            scale_x_continuous(breaks = c(1:10), labels = c(1:10)) +
            scale_y_continuous(labels = function(x) sub(".0+$", "", x)) +
            theme_bw(base_size = 13.8) +
            theme(legend.text=element_text(size = 13)),
          
          common.legend = T, legend = "bottom") + 
  bgcolor("white") + 
  border("white")
```

```
ggsave("figures/fixmodel_alpha_pow_study_arm.png", width = 10, height = 5)
ggsave("figures/fixmodel_alpha_pow_study_arm.pdf", width = 10, height = 5)
ggsave("figures/fixmodel_alpha_pow_study_arm.tiff", width = 10, height = 5)
```

### Figure S4

- Evaluated arm: 5
- \(\lambda=0.5\)
- Calendar time unit size: 450
- \(n=250\) per experimental
  treatment arm
- \(N\_p\) for inverted-U time trend
  placed to the middle of the trial
- Rows - Degrees of B-splines
- T1E and power with respect to the strength of the time trend \(\lambda\) under different time trend
  patterns

```
ggarrange(ggplot(results_setting_1_splines_alpha %>% filter(study_arm==5 & lambda0==0.5 & unit_size==450)) +
            annotate("rect", xmin = -Inf, xmax = Inf, ymin = pred_int[1], ymax = pred_int[2], fill = "gray54", alpha = 0.4) +
            geom_line(aes(d2, reject_h0, color=model)) +
            geom_point(aes(d2, reject_h0, color=model)) +
            labs(x="d", y="Type I error rate", color="Analysis:") +
            geom_hline(aes(yintercept = 0.025), linetype = "dotted") +
            facet_grid(bs_degree ~ trend,
                       labeller = labeller(trend  = as_labeller(trend_names),
                                           bs_degree = as_labeller(degree_names))) +
            #scale_x_continuous(labels = function(x) sub(".0+$", "", x)) +
            scale_y_continuous(labels = function(x) sub(".0+$", "", x)) +
            coord_cartesian(ylim = c(0, 0.05)) +
            scale_color_manual(labels = c("Fixed - period", "Separate analysis", "Splines - period", "Splines - calendar"), values = my_palette, limits = force) +
            theme_bw(base_size = 13.8) +
            theme(legend.text=element_text(size = 13)),
          
          ggplot(results_setting_1_splines_pow %>% filter(study_arm==5 & lambda0==0.5 & unit_size==450)) +
            geom_line(aes(d2, reject_h0, color=model)) +
            geom_point(aes(d2, reject_h0, color=model)) +
            labs(x="d", y="Power", color="Analysis:") +
            facet_grid(bs_degree ~ trend,
                       labeller = labeller(trend  = as_labeller(trend_names),
                                           bs_degree = as_labeller(degree_names))) +
            #scale_x_continuous(labels = function(x) sub(".0+$", "", x)) +
            scale_y_continuous(labels = function(x) sub(".0+$", "", x)) +
            coord_cartesian(ylim = c(0.70, 1)) +
            scale_color_manual(labels = c("Fixed - period", "Separate analysis", "Splines - period", "Splines - calendar"), values = my_palette, limits = force) +
            theme_bw(base_size = 13.8) +
            theme(legend.text=element_text(size = 13)),
          
          common.legend = T, legend = "bottom", ncol = 1) + 
  bgcolor("white") + 
  border("white")
```

```
ggsave("figures/splines_alpha_pow_d_trend_all_degrees.png", width = 10, height = 12)
ggsave("figures/splines_alpha_pow_d_trend_all_degrees.pdf", width = 10, height = 12)
ggsave("figures/splines_alpha_pow_d_trend_all_degrees.tiff", width = 10, height = 12)
```

### Figure S5

- Evaluated arm: 5
- \(d=500\)
- Calendar time unit size: 450
- \(n=250\) per experimental
  treatment arm
- \(N\_p=2500\) for inverted-U time
  trend (corresponding to the middle of the trial)
- Rows - Degrees of B-splines
- T1E and power with respect to the strength of the time trend \(\lambda\) under different time trend
  patterns

```
ggarrange(ggplot(results_setting_1_splines_alpha %>% filter(study_arm==5 & d2==500 & unit_size==450)) +
            annotate("rect", xmin = -Inf, xmax = Inf, ymin = pred_int[1], ymax = pred_int[2], fill = "gray54", alpha = 0.4) +
            geom_line(aes(lambda0, reject_h0, color=model)) +
            geom_point(aes(lambda0, reject_h0, color=model)) +
            labs(x=TeX("$\\lambda$"), y="Type I error rate", color="Analysis:") +
            geom_hline(aes(yintercept = 0.025), linetype = "dotted") +
            facet_grid(bs_degree ~ trend,
                       labeller = labeller(trend  = as_labeller(trend_names),
                                           bs_degree = as_labeller(degree_names))) +
            scale_x_continuous(labels = function(x) sub(".0+$", "", x)) +
            scale_y_continuous(labels = function(x) sub(".0+$", "", x)) +
            coord_cartesian(ylim = c(0, 0.05)) +
            scale_color_manual(labels = c("Fixed - period", "Separate analysis", "Splines - period", "Splines - calendar"), values = my_palette, limits = force) +
            theme_bw(base_size = 13.8) +
            theme(legend.text=element_text(size = 13)),
          
          ggplot(results_setting_1_splines_pow %>% filter(study_arm==5 & d2==500 & unit_size==450)) +
            geom_line(aes(lambda0, reject_h0, color=model)) +
            geom_point(aes(lambda0, reject_h0, color=model)) +
            labs(x=TeX("$\\lambda$"), y="Power", color="Analysis:") +
            facet_grid(bs_degree ~ trend,
                       labeller = labeller(trend  = as_labeller(trend_names),
                                           bs_degree = as_labeller(degree_names))) +
            scale_x_continuous(labels = function(x) sub(".0+$", "", x)) +
            scale_y_continuous(labels = function(x) sub(".0+$", "", x)) +
            coord_cartesian(ylim = c(0.70, 1)) +
            scale_color_manual(labels = c("Fixed - period", "Separate analysis", "Splines - period", "Splines - calendar"), values = my_palette, limits = force) +
            theme_bw(base_size = 13.8) +
            theme(legend.text=element_text(size = 13)),
          
          common.legend = T, legend = "bottom", ncol = 1) + 
  bgcolor("white") + 
  border("white")
```

```
ggsave("figures/splines_alpha_pow_lambda_trend_all_degrees.png", width = 10, height = 12)
ggsave("figures/splines_alpha_pow_lambda_trend_all_degrees.pdf", width = 10, height = 12)
ggsave("figures/splines_alpha_pow_lambda_trend_all_degrees.tiff", width = 10, height = 12)
```

### Figure S6

- Degree of B-spline: 3 (cubic)
- Calendar time unit size: 450
- \(\lambda = 0.5\)
- \(n=250\) per experimental
  treatment arm
- \(N\_p\) for inverted-U time trend
  placed to the middle of the trial
- Rows - Evaluated arm
- T1E and power with respect to the strength of the time trend \(\lambda\) under different time trend
  patterns

```
ggarrange(ggplot(results_setting_1_splines_alpha %>% filter(bs_degree==3, lambda0==0.5, unit_size==450)) +
            annotate("rect", xmin = -Inf, xmax = Inf, ymin = pred_int[1], ymax = pred_int[2], fill = "gray54", alpha = 0.4) +
            geom_line(aes(d2, reject_h0, color = as.factor(model))) +
            geom_point(aes(d2, reject_h0, color = as.factor(model))) +
            labs(x="d", y="Type I error rate", color="Analysis approach:") +
            scale_color_manual(labels = c("Fixed - period", "Separate analysis", "Splines - period", "Splines - calendar"), values = my_palette, limits = force) +
            geom_hline(aes(yintercept = 0.025), linetype = "dotted") +
            #coord_cartesian(ylim = c(0, 0.1)) +
            facet_grid(study_arm ~ trend,
                       labeller = labeller(trend = as_labeller(trend_names))) +
            #scale_x_continuous(labels = function(x) sub(".0+$", "", x)) +
            scale_y_continuous(labels = function(x) sub(".0+$", "", x)) +
            theme_bw(base_size = 13.8) +
            theme(legend.text=element_text(size = 13)),
          
          ggplot(results_setting_1_splines_pow %>% filter(bs_degree==3, lambda0==0.5, unit_size==450)) +
            geom_line(aes(d2, reject_h0, color = as.factor(model))) +
            geom_point(aes(d2, reject_h0, color = as.factor(model))) +
            labs(x="d", y="Power", color="Analysis approach:") +
            scale_color_manual(labels = c("Fixed - period", "Separate analysis", "Splines - period", "Splines - calendar"), values = my_palette, limits = force) +
            coord_cartesian(ylim = c(0.70, 1)) +
            facet_grid(study_arm ~ trend,
                       labeller = labeller(trend  = as_labeller(trend_names))) +
            #scale_x_continuous(labels = function(x) sub(".0+$", "", x)) +
            scale_y_continuous(labels = function(x) sub(".0+$", "", x)) +
            theme_bw(base_size = 13.8) +
            theme(legend.text=element_text(size = 13)),
          
          common.legend = T, legend = "bottom",
          nrow = 2) +
  bgcolor("white") +
  border("white")
```

```
ggsave("figures/splines_alpha_pow_d_trend_all_arms.png", width = 10, height = 15)
ggsave("figures/splines_alpha_pow_d_trend_all_arms.pdf", width = 10, height = 15)
ggsave("figures/splines_alpha_pow_d_trend_all_arms.tiff", width = 10, height = 15)
```

### Figure S7

- Degree of B-spline: 3 (cubic)
- Calendar time unit size: 450
- \(d=500\)
- \(n=250\) per experimental
  treatment arm
- \(N\_p=2500\) for inverted-U time
  trend (corresponding to the middle of the trial)
- Rows - Evaluated arm
- T1E and power with respect to the strength of the time trend \(\lambda\) under different time trend
  patterns

```
ggarrange(ggplot(results_setting_1_splines_alpha %>% filter(bs_degree==3, d2==500, unit_size==450)) +
            annotate("rect", xmin = -Inf, xmax = Inf, ymin = pred_int[1], ymax = pred_int[2], fill = "gray54", alpha = 0.4) +
            geom_line(aes(lambda1, reject_h0, color = as.factor(model))) +
            geom_point(aes(lambda1, reject_h0, color = as.factor(model))) +
            labs(x=TeX("$\\lambda$"), y="Type I error rate", color="Analysis approach:") +
            scale_color_manual(labels = c("Fixed - period", "Separate analysis", "Splines - period", "Splines - calendar"), values = my_palette, limits = force) +
            geom_hline(aes(yintercept = 0.025), linetype = "dotted") +
            #coord_cartesian(ylim = c(0, 0.1)) +
            facet_grid(study_arm ~ trend,
                       labeller = labeller(trend = as_labeller(trend_names))) +
            scale_x_continuous(labels = function(x) sub(".0+$", "", x)) +
            scale_y_continuous(labels = function(x) sub(".0+$", "", x)) +
            theme_bw(base_size = 13.8) +
            theme(legend.text=element_text(size = 13)),
          
          ggplot(results_setting_1_splines_pow %>% filter(bs_degree==3, d2==500, unit_size==450)) +
            geom_line(aes(lambda1, reject_h0, color = as.factor(model))) +
            geom_point(aes(lambda1, reject_h0, color = as.factor(model))) +
            labs(x=TeX("$\\lambda$"), y="Power", color="Analysis approach:") +
            scale_color_manual(labels = c("Fixed - period", "Separate analysis", "Splines - period", "Splines - calendar"), values = my_palette, limits = force) +
            coord_cartesian(ylim = c(0.70, 1)) +
            facet_grid(study_arm ~ trend,
                       labeller = labeller(trend  = as_labeller(trend_names))) +
            scale_x_continuous(labels = function(x) sub(".0+$", "", x)) +
            scale_y_continuous(labels = function(x) sub(".0+$", "", x)) +
            theme_bw(base_size = 13.8) +
            theme(legend.text=element_text(size = 13)),
          
          common.legend = T, legend = "bottom",
          nrow = 2) +
  bgcolor("white") +
  border("white")
```

```
ggsave("figures/splines_alpha_pow_lambda_trend_all_arms.png", width = 10, height = 15)
ggsave("figures/splines_alpha_pow_lambda_trend_all_arms.pdf", width = 10, height = 15)
ggsave("figures/splines_alpha_pow_lambda_trend_all_arms.tiff", width = 10, height = 15)
```

# SETTING 2 - 4 treatment arms, equal time trends

## Plots from Section 4

### Figure 8

- Evaluated arm: 3
- \(\lambda=0.125\)
- \(n=250\) per experimental
  treatment arm
- \(N\_p=750\) for inverted-U time
  trend (corresponding approximately to the middle of the trial)
- T1E and power with respect to the size of the calendar time unit
  under different time trend patterns

```
ggarrange(ggplot(results_setting_2_alpha %>% filter(study_arm==3, lambda1==0.125)) +
            annotate("rect", xmin = -Inf, xmax = Inf, ymin = pred_int[1], ymax = pred_int[2], fill = "gray54", alpha = 0.4) +
            geom_line(aes(unit_size, reject_h0, color = as.factor(model))) +
            geom_point(aes(unit_size, reject_h0, color = as.factor(model)), size = 1) +
            labs(x="Calendar unit size", y="Type I error rate", color="Analysis approach:") +
            scale_color_manual(labels = c("Fixed - period", "Fixed - calendar", "Separate analysis"), values = my_palette, limits = force) +
            geom_hline(aes(yintercept = 0.025), linetype = "dotted") +
            #coord_cartesian(ylim = c(0, 0.1)) +
            facet_grid(~ trend,
                       labeller = labeller(trend  = as_labeller(trend_names))) +
            scale_y_continuous(labels = function(x) sub(".0+$", "", x)) +
            theme_bw(base_size = 13.8) +
            theme(legend.text=element_text(size = 13)),
          
          ggplot(results_setting_2_pow %>% filter(study_arm==3, lambda1==0.125)) +
            geom_line(aes(unit_size, reject_h0, color = as.factor(model))) +
            geom_point(aes(unit_size, reject_h0, color = as.factor(model)), size = 1) +
            labs(x="Calendar unit size", y="Power", color="Analysis approach:") +
            scale_color_manual(labels = c("Fixed - period", "Fixed - calendar", "Separate analysis"), values = my_palette, limits = force) +
            coord_cartesian(ylim = c(0.70, 1)) +
            facet_grid(~ trend,
                       labeller = labeller(trend  = as_labeller(trend_names))) +
            scale_y_continuous(labels = function(x) sub(".0+$", "", x)) +
            theme_bw(base_size = 13.8) +
            theme(legend.text=element_text(size = 13)),
          
          common.legend = T, legend = "bottom",
          nrow = 2) + 
  bgcolor("white") + 
  border("white")
```

```
ggsave("figures/fixmodel_cal_alpha_pow_unit.png", width = 10, height = 6)
ggsave("figures/fixmodel_cal_alpha_pow_unit.pdf", width = 10, height = 6)
ggsave("figures/fixmodel_cal_alpha_pow_unit.tiff", width = 10, height = 6)
```

### Figure 9

- Evaluated arm: 3
- Calendar time unit size: 100
- \(n=250\) per experimental
  treatment arm
- \(N\_p=750\) for inverted-U time
  trend (corresponding approximately to the middle of the trial)
- T1E and power with respect to the strength of the time trend \(\lambda\) under different time trend
  patterns

```
ggarrange(ggplot(results_setting_2_alpha %>% filter(study_arm==3, unit_size==100)) +
            annotate("rect", xmin = -Inf, xmax = Inf, ymin = pred_int[1], ymax = pred_int[2], fill = "gray54", alpha = 0.4) +
            geom_line(aes(lambda1, reject_h0, color = as.factor(model))) +
            geom_point(aes(lambda1, reject_h0, color = as.factor(model))) +
            labs(x=TeX("$\\lambda$"), y="Type I error rate", color="Analysis approach:") +
            scale_color_manual(labels = c("Fixed - period", "Fixed - calendar", "Separate analysis"), values = my_palette, limits = force) +
            geom_hline(aes(yintercept = 0.025), linetype = "dotted") +
            #coord_cartesian(ylim = c(0, 0.1)) +
            facet_grid( ~ trend,
                        labeller = labeller(trend  = as_labeller(trend_names))) +
            scale_x_continuous(labels = function(x) sub(".0+$", "", x)) +
            scale_y_continuous(labels = function(x) sub(".0+$", "", x)) +
            theme_bw(base_size = 13.8) +
            theme(legend.text=element_text(size = 13)),
          
          ggplot(results_setting_2_pow %>% filter(study_arm==3, unit_size==100)) +
            geom_line(aes(lambda1, reject_h0, color = as.factor(model))) +
            geom_point(aes(lambda1, reject_h0, color = as.factor(model))) +
            labs(x=TeX("$\\lambda$"), y="Power", color="Analysis approach:") +
            scale_color_manual(labels = c("Fixed - period", "Fixed - calendar", "Separate analysis"), values = my_palette, limits = force) +
            coord_cartesian(ylim = c(0.70, 1)) +
            facet_grid( ~ trend,
                        labeller = labeller(trend  = as_labeller(trend_names))) +
            scale_x_continuous(labels = function(x) sub(".0+$", "", x)) +
            scale_y_continuous(labels = function(x) sub(".0+$", "", x)) +
            theme_bw(base_size = 13.8) +
            theme(legend.text=element_text(size = 13)),
          
          common.legend = T, legend = "bottom",
          nrow = 2) + 
  bgcolor("white") + 
  border("white")
```

```
ggsave("figures/fixmodel_cal_alpha_pow_lambda.png", width = 10, height = 6)
ggsave("figures/fixmodel_cal_alpha_pow_lambda.pdf", width = 10, height = 6)
ggsave("figures/fixmodel_cal_alpha_pow_lambda.tiff", width = 10, height = 6)
```

### Figure 10

- Evaluated arm: 3
- Calendar time unit size: 100
- \(n=250\) per experimental
  treatment arm
- \(N\_p=750\) for inverted-U time
  trend (corresponding approximately to the middle of the trial)
- T1E and power with respect to the strength of the time trend \(\lambda\) under different time trend
  patterns

```
ggarrange(ggplot(results_setting_2_mix_alpha %>% filter(study_arm==3, unit_size==100)) +
            annotate("rect", xmin = -Inf, xmax = Inf, ymin = pred_int[1], ymax = pred_int[2], fill = "gray54", alpha = 0.4) +
            geom_line(aes(lambda1, reject_h0, color = as.factor(model))) +
            geom_point(aes(lambda1, reject_h0, color = as.factor(model))) +
            labs(x=TeX("$\\lambda$"), y="Type I error rate", color="Analysis approach:") +
            scale_color_manual(labels = c("Fixed - period", "Mixed - period", "Mixed (AR1) - period", 
                                          "Mixed (AR1) - calendar", "Mixed - calendar", "Separate analysis"),
                               values = my_palette, limits = force) +
            geom_hline(aes(yintercept = 0.025), linetype = "dotted") +
            #coord_cartesian(ylim = c(0, 0.1)) +
            facet_grid(~ trend,
                       labeller = labeller(trend  = as_labeller(trend_names))) +
            scale_x_continuous(labels = function(x) sub(".0+$", "", x)) +
            scale_y_continuous(labels = function(x) sub(".0+$", "", x)) +
            theme_bw(base_size = 13.8) +
            theme(legend.text=element_text(size = 13)),
          
          ggplot(results_setting_2_mix_pow %>% filter(study_arm==3, unit_size==100)) +
            geom_line(aes(lambda1, reject_h0, color = as.factor(model))) +
            geom_point(aes(lambda1, reject_h0, color = as.factor(model))) +
            labs(x=TeX("$\\lambda$"), y="Power", color="Analysis approach:") +
            scale_color_manual(labels = c("Fixed - period", "Mixed - period", "Mixed (AR1) - period", 
                                          "Mixed (AR1) - calendar", "Mixed - calendar", "Separate analysis"),
                               values = my_palette, limits = force) +
            coord_cartesian(ylim = c(0.70, 1)) +
            facet_grid(~ trend,
                       labeller = labeller(trend  = as_labeller(trend_names))) +
            scale_x_continuous(labels = function(x) sub(".0+$", "", x)) +
            scale_y_continuous(labels = function(x) sub(".0+$", "", x)) +
            theme_bw(base_size = 13.8) +
            theme(legend.text=element_text(size = 13)),
          
          common.legend = T, legend = "bottom",
          nrow = 2) + 
  bgcolor("white") + 
  border("white")
```

```
ggsave("figures/mixmodel_alpha_pow_lambda.png", width = 10, height = 6)
ggsave("figures/mixmodel_alpha_pow_lambda.pdf", width = 10, height = 6)
ggsave("figures/mixmodel_alpha_pow_lambda.tiff", width = 10, height = 6)
```

## Plots from the Supplementary Material

### Figure S8

- \(\lambda=0.125\)
- \(n=250\) per experimental
  treatment arm
- \(N\_p=750\) for inverted-U time
  trend (corresponding approximately to the middle of the trial)
- Rows - Evaluated arm
- T1E and power with respect to the size of the calendar time unit
  under different time trend patterns

```
ggarrange(ggplot(results_setting_2_alpha %>% filter(lambda1==0.125)) +
            annotate("rect", xmin = -Inf, xmax = Inf, ymin = pred_int[1], ymax = pred_int[2], fill = "gray54", alpha = 0.4) +
            geom_line(aes(unit_size, reject_h0, color = as.factor(model))) +
            geom_point(aes(unit_size, reject_h0, color = as.factor(model)), size = 1) +
            labs(x="Calendar unit size", y="Type I error rate", color="Analysis approach:") +
            scale_color_manual(labels = c("Fixed - period", "Fixed - calendar", "Separate analysis"), values = my_palette, limits = force) +
            geom_hline(aes(yintercept = 0.025), linetype = "dotted") +
            #coord_cartesian(ylim = c(0, 0.1)) +
            facet_grid(study_arm ~ trend,
                       labeller = labeller(trend  = as_labeller(trend_names))) +
            scale_y_continuous(labels = function(x) sub(".0+$", "", x)) +
            theme_bw(base_size = 13.8) +
            theme(legend.text=element_text(size = 13)),
          
          ggplot(results_setting_2_pow %>% filter(lambda1==0.125)) +
            geom_line(aes(unit_size, reject_h0, color = as.factor(model))) +
            geom_point(aes(unit_size, reject_h0, color = as.factor(model)), size = 1) +
            labs(x="Calendar unit size", y="Power", color="Analysis approach:") +
            scale_color_manual(labels = c("Fixed - period", "Fixed - calendar", "Separate analysis"), values = my_palette, limits = force) +
            coord_cartesian(ylim = c(0.70, 1)) +
            facet_grid(study_arm ~ trend,
                       labeller = labeller(trend  = as_labeller(trend_names))) +
            scale_y_continuous(labels = function(x) sub(".0+$", "", x)) +
            theme_bw(base_size = 13.8) +
            theme(legend.text=element_text(size = 13)),
          
          common.legend = T, legend = "bottom",
          nrow = 2) + 
  bgcolor("white") + 
  border("white")
```

```
ggsave("figures/fixmodel_cal_alpha_pow_unit_all_arms.png", width = 10, height = 12)
ggsave("figures/fixmodel_cal_alpha_pow_unit_all_arms.pdf", width = 10, height = 12)
ggsave("figures/fixmodel_cal_alpha_pow_unit_all_arms.tiff", width = 10, height = 12)
```

### Figure S9

- Calendar time unit size: 100
- \(n=250\) per experimental
  treatment arm
- \(N\_p=750\) for inverted-U time
  trend (corresponding approximately to the middle of the trial)
- Rows - Evaluated arm
- T1E and power with respect to the strength of the time trend \(\lambda\) under different time trend
  patterns

```
ggarrange(ggplot(results_setting_2_alpha %>% filter(unit_size==100)) +
            annotate("rect", xmin = -Inf, xmax = Inf, ymin = pred_int[1], ymax = pred_int[2], fill = "gray54", alpha = 0.4) +
            geom_line(aes(lambda1, reject_h0, color = as.factor(model))) +
            geom_point(aes(lambda1, reject_h0, color = as.factor(model))) +
            labs(x=TeX("$\\lambda$"), y="Type I error rate", color="Analysis approach:") +
            scale_color_manual(labels = c("Fixed - period", "Fixed - calendar", "Separate analysis"), values = my_palette, limits = force) +
            geom_hline(aes(yintercept = 0.025), linetype = "dotted") +
            #coord_cartesian(ylim = c(0, 0.1)) +
            facet_grid(study_arm ~ trend,
                       labeller = labeller(trend  = as_labeller(trend_names))) +
            scale_x_continuous(labels = function(x) sub(".0+$", "", x)) +
            scale_y_continuous(labels = function(x) sub(".0+$", "", x)) +
            theme_bw(base_size = 13.8) +
            theme(legend.text=element_text(size = 13)),
          
          ggplot(results_setting_2_pow %>% filter(unit_size==100)) +
            geom_line(aes(lambda1, reject_h0, color = as.factor(model))) +
            geom_point(aes(lambda1, reject_h0, color = as.factor(model))) +
            labs(x=TeX("$\\lambda$"), y="Power", color="Analysis approach:") +
            scale_color_manual(labels = c("Fixed - period", "Fixed - calendar", "Separate analysis"), values = my_palette, limits = force) +
            coord_cartesian(ylim = c(0.70, 1)) +
            facet_grid(study_arm ~ trend,
                       labeller = labeller(trend  = as_labeller(trend_names))) +
            scale_x_continuous(labels = function(x) sub(".0+$", "", x)) +
            scale_y_continuous(labels = function(x) sub(".0+$", "", x)) +
            theme_bw(base_size = 13.8) +
            theme(legend.text=element_text(size = 13)),
          
          common.legend = T, legend = "bottom",
          nrow = 2) + 
  bgcolor("white") + 
  border("white")
```

```
ggsave("figures/fixmodel_cal_alpha_pow_lambda_all_arms.png", width = 10, height = 12)
ggsave("figures/fixmodel_cal_alpha_pow_lambda_all_arms.pdf", width = 10, height = 12)
ggsave("figures/fixmodel_cal_alpha_pow_lambda_all_arms.tiff", width = 10, height = 12)
```

### Figure S10

- Calendar time unit size: 100
- \(n=250\) per experimental
  treatment arm
- \(N\_p=750\) for inverted-U time
  trend (corresponding approximately to the middle of the trial)
- Rows - Evaluated arm
- T1E and power with respect to the strength of the time trend \(\lambda\) under different time trend
  patterns

```
ggarrange(ggplot(results_setting_2_mix_alpha %>% filter(unit_size==100)) +
            annotate("rect", xmin = -Inf, xmax = Inf, ymin = pred_int[1], ymax = pred_int[2], fill = "gray54", alpha = 0.4) +
            geom_line(aes(lambda1, reject_h0, color = as.factor(model))) +
            geom_point(aes(lambda1, reject_h0, color = as.factor(model))) +
            labs(x=TeX("$\\lambda$"), y="Type I error rate", color="Analysis approach:") +
            scale_color_manual(labels = c("Fixed - period", "Mixed - period", "Mixed (AR1) - period", 
                                          "Mixed (AR1) - calendar", "Mixed - calendar", "Separate analysis"),
                               values = my_palette, limits = force) +
            geom_hline(aes(yintercept = 0.025), linetype = "dotted") +
            #coord_cartesian(ylim = c(0, 0.1)) +
            facet_grid(study_arm ~ trend,
                       labeller = labeller(trend  = as_labeller(trend_names))) +
            scale_x_continuous(labels = function(x) sub(".0+$", "", x)) +
            scale_y_continuous(labels = function(x) sub(".0+$", "", x)) +
            theme_bw(base_size = 13.8) +
            theme(legend.text=element_text(size = 13)),
          
          ggplot(results_setting_2_mix_pow %>% filter(unit_size==100)) +
            geom_line(aes(lambda1, reject_h0, color = as.factor(model))) +
            geom_point(aes(lambda1, reject_h0, color = as.factor(model))) +
            labs(x=TeX("$\\lambda$"), y="Power", color="Analysis approach:") +
            scale_color_manual(labels = c("Fixed - period", "Mixed - period", "Mixed (AR1) - period", 
                                          "Mixed (AR1) - calendar", "Mixed - calendar", "Separate analysis"),
                               values = my_palette, limits = force) +
            coord_cartesian(ylim = c(0.70, 1)) +
            facet_grid(study_arm ~ trend,
                       labeller = labeller(trend  = as_labeller(trend_names))) +
            scale_x_continuous(labels = function(x) sub(".0+$", "", x)) +
            scale_y_continuous(labels = function(x) sub(".0+$", "", x)) +
            theme_bw(base_size = 13.8) +
            theme(legend.text=element_text(size = 13)),
          
          common.legend = T, legend = "bottom",
          nrow = 2) + 
  bgcolor("white") + 
  border("white")
```

```
ggsave("figures/mixmodel_alpha_pow_lambda_all_arms.png", width = 10, height = 12)
ggsave("figures/mixmodel_alpha_pow_lambda_all_arms.pdf", width = 10, height = 12)
ggsave("figures/mixmodel_alpha_pow_lambda_all_arms.tiff", width = 10, height = 12)
```

# SETTING 3 - 4 treatment arms, different time trends

## Plots from Section 4

### Figure 11

- Evaluated arm: 3
- Calendar time unit size: 100
- \(n=250\) per experimental
  treatment arm
- \(N\_p=750\) for inverted-U time
  trend (corresponding approximately to the middle of the trial)
- T1E and power with respect to the strength of the time trend \(\lambda\_1\) under different time trend
  cases

```
ggarrange(ggplot(rbind(results_setting_3_alpha, results_setting_3_alpha_add) %>% filter(study_arm==3, unit_size==100)) +
            annotate("rect", xmin = -Inf, xmax = Inf, ymin = pred_int[1], ymax = pred_int[2], fill = "gray54", alpha = 0.4) +
            geom_line(aes(lambda1, reject_h0, color = as.factor(model))) +
            geom_point(aes(lambda1, reject_h0, color = as.factor(model))) +
            labs(x=TeX("$\\lambda_1$"), y="Type I error rate", color="Analysis approach:") +
            scale_color_manual(labels = c("Fixed - period", "Mixed w. inter. - period", "Mixed w. inter. - calendar", "Separate analysis"),
                               values = my_palette, limits = force) +
            geom_hline(aes(yintercept = 0.025), linetype = "dotted") +
            #coord_cartesian(ylim = c(0, 0.1)) +
            facet_grid(~ trend_case,
                       labeller = labeller(trend_case = as_labeller(trend_case_names, label_parsed))) +
            scale_x_continuous(labels = function(x) sub(".0+$", "", x)) +
            scale_y_continuous(labels = function(x) sub(".0+$", "", x)) +
            theme_bw(base_size = 13.8) +
            theme(legend.text=element_text(size = 13),
                  strip.text = element_text(size = 9.5)),
          
          ggplot(rbind(results_setting_3_pow, results_setting_3_pow_add) %>% filter(study_arm==3, unit_size==100)) +
            geom_line(aes(lambda1, reject_h0, color = as.factor(model))) +
            geom_point(aes(lambda1, reject_h0, color = as.factor(model))) +
            labs(x=TeX("$\\lambda_1$"), y="Power", color="Analysis approach:") +
            scale_color_manual(labels = c("Fixed - period", "Mixed w. inter. - period", "Mixed w. inter. - calendar", "Separate analysis"),
                               values = my_palette, limits = force) +
            coord_cartesian(ylim = c(0.70, 1)) +
            facet_grid(~ trend_case,
                       labeller = labeller(trend_case = as_labeller(trend_case_names, label_parsed))) +
            scale_x_continuous(labels = function(x) sub(".0+$", "", x)) +
            scale_y_continuous(labels = function(x) sub(".0+$", "", x)) +
            theme_bw(base_size = 13.8) +
            theme(legend.text=element_text(size = 13),
                  strip.text = element_text(size = 9.5)),
          
          common.legend = T, legend = "bottom",
          nrow = 2) + 
  bgcolor("white") + 
  border("white")
```

```
ggsave("figures/mixint_alpha_pow_lambda.png", width = 10, height = 6)
ggsave("figures/mixint_alpha_pow_lambda.pdf", width = 10, height = 6)
ggsave("figures/mixint_alpha_pow_lambda.tiff", width = 10, height = 6)
```
