# Supplementary figures and images for: Statistical Modeling to Adjust for Time Trends in Adaptive Platform Trials Utilizing Non‐Concurrent Controls
Source: Biom J. 2025 Jun 10;67(3):e70059. doi: 10.1002/bimj.70059 (PMC12150008; doi:10.1002/bimj.70059)

Count

Fixed effect model

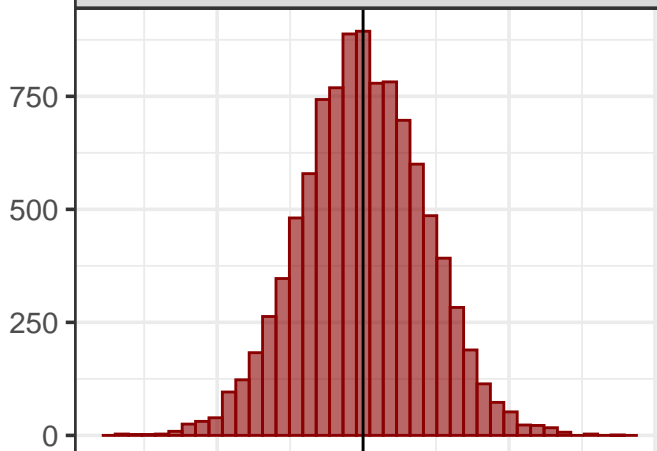

Mixed model

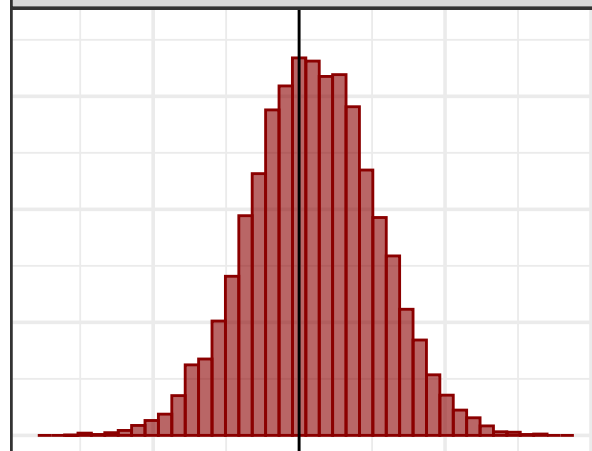

Mixed model (AR1)

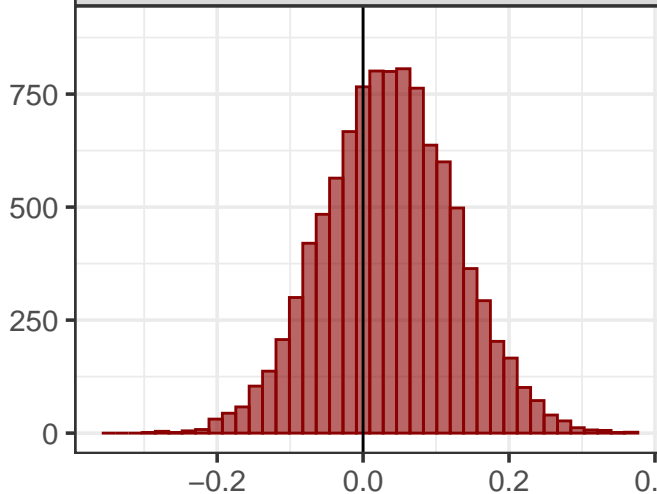

Spline regression

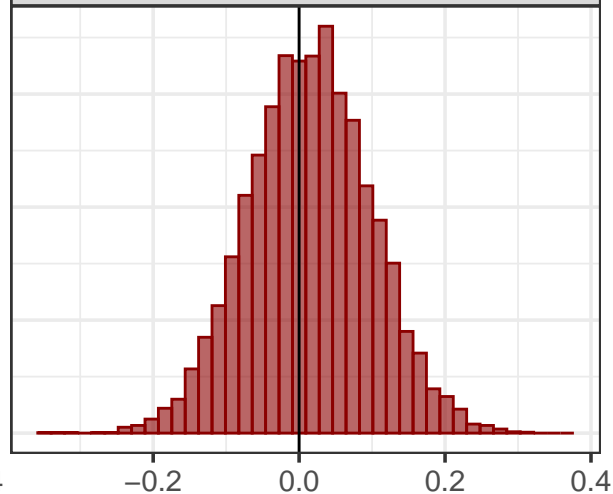

Estimation error

Supplement: Supplementary file 1 — Supporting Information [file BIMJ-67-e70059-s002.zip › case_studies/FLAIR/figures/FLAIR_bias.pdf]

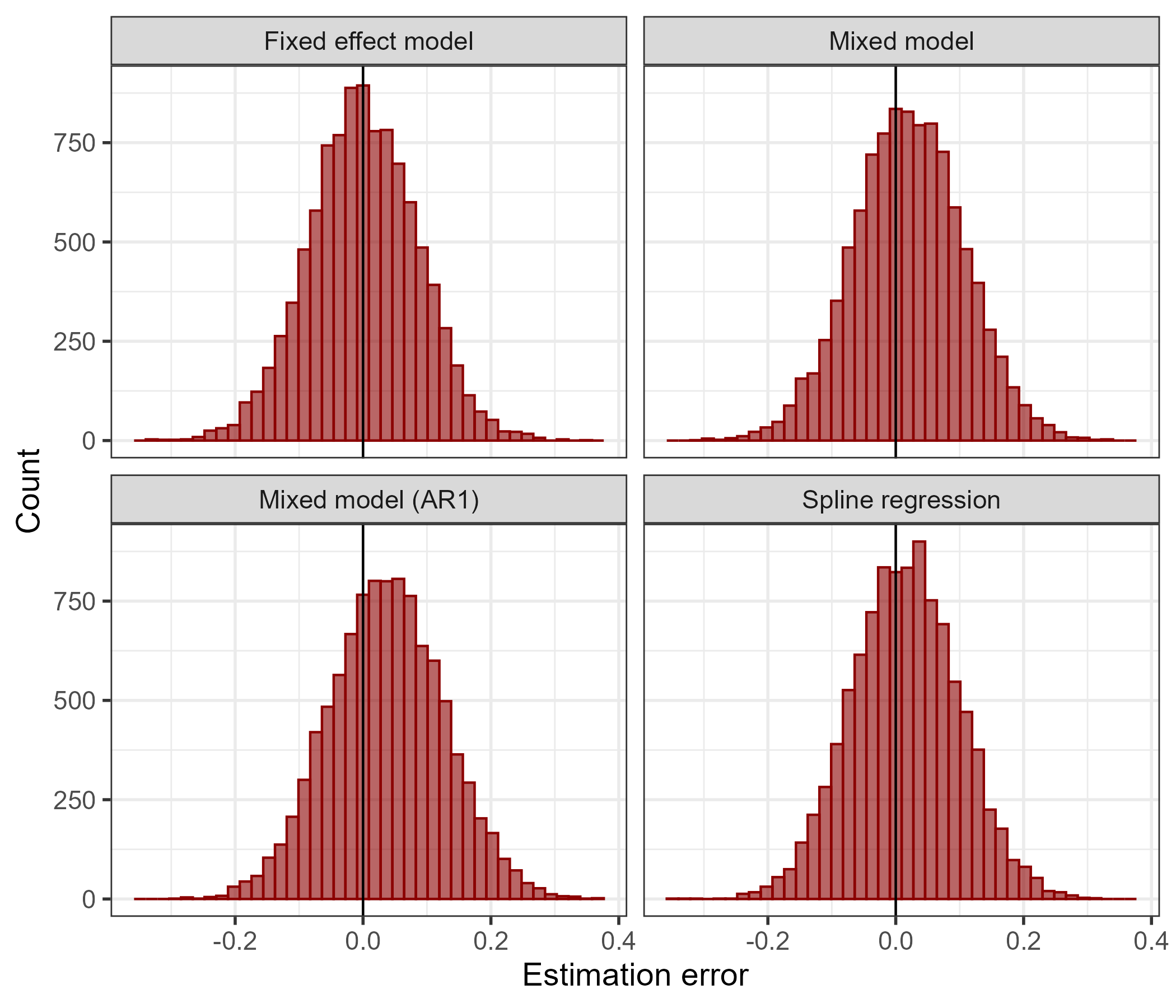

Supplement: Supplementary file 1 — Supporting Information [file BIMJ-67-e70059-s002.zip › case_studies/FLAIR/figures/FLAIR_bias.png]

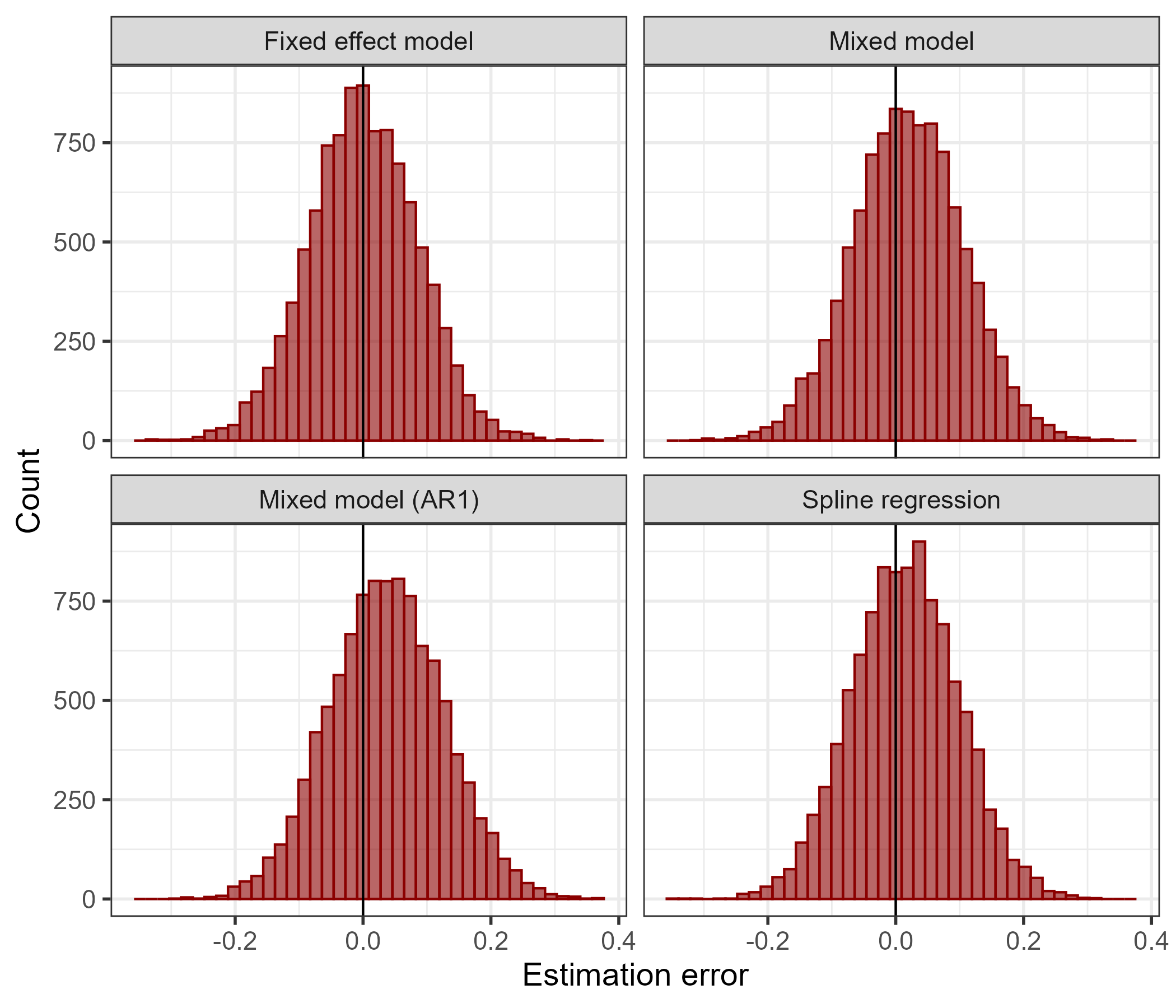

Supplement: Supplementary file 1 — Supporting Information [file BIMJ-67-e70059-s002.zip › case_studies/FLAIR/figures/FLAIR_bias.tiff]

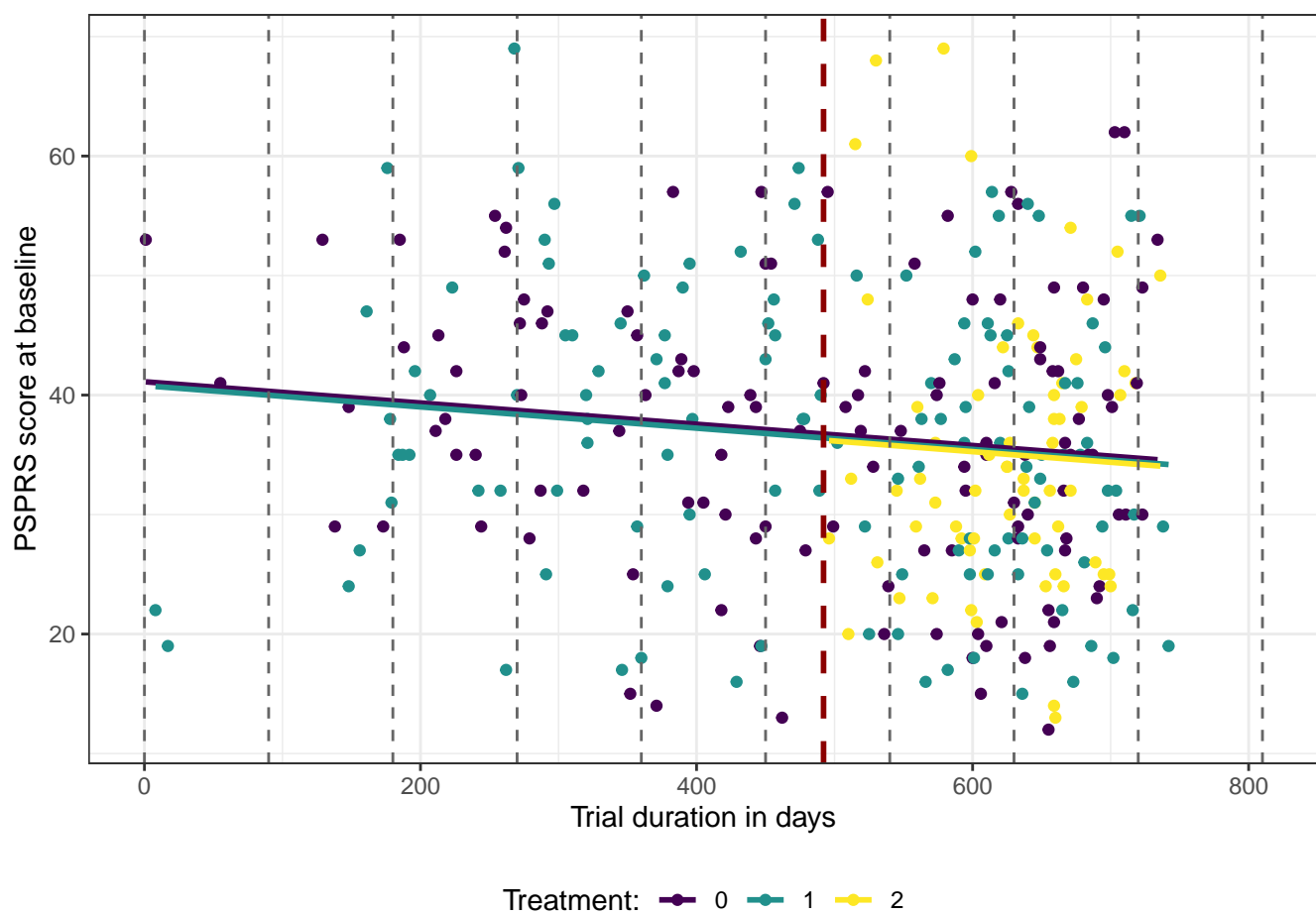

Supplement: Supplementary file 1 — Supporting Information [file BIMJ-67-e70059-s002.zip › case_studies/PSP/figures/casestudy_trialdata.pdf]

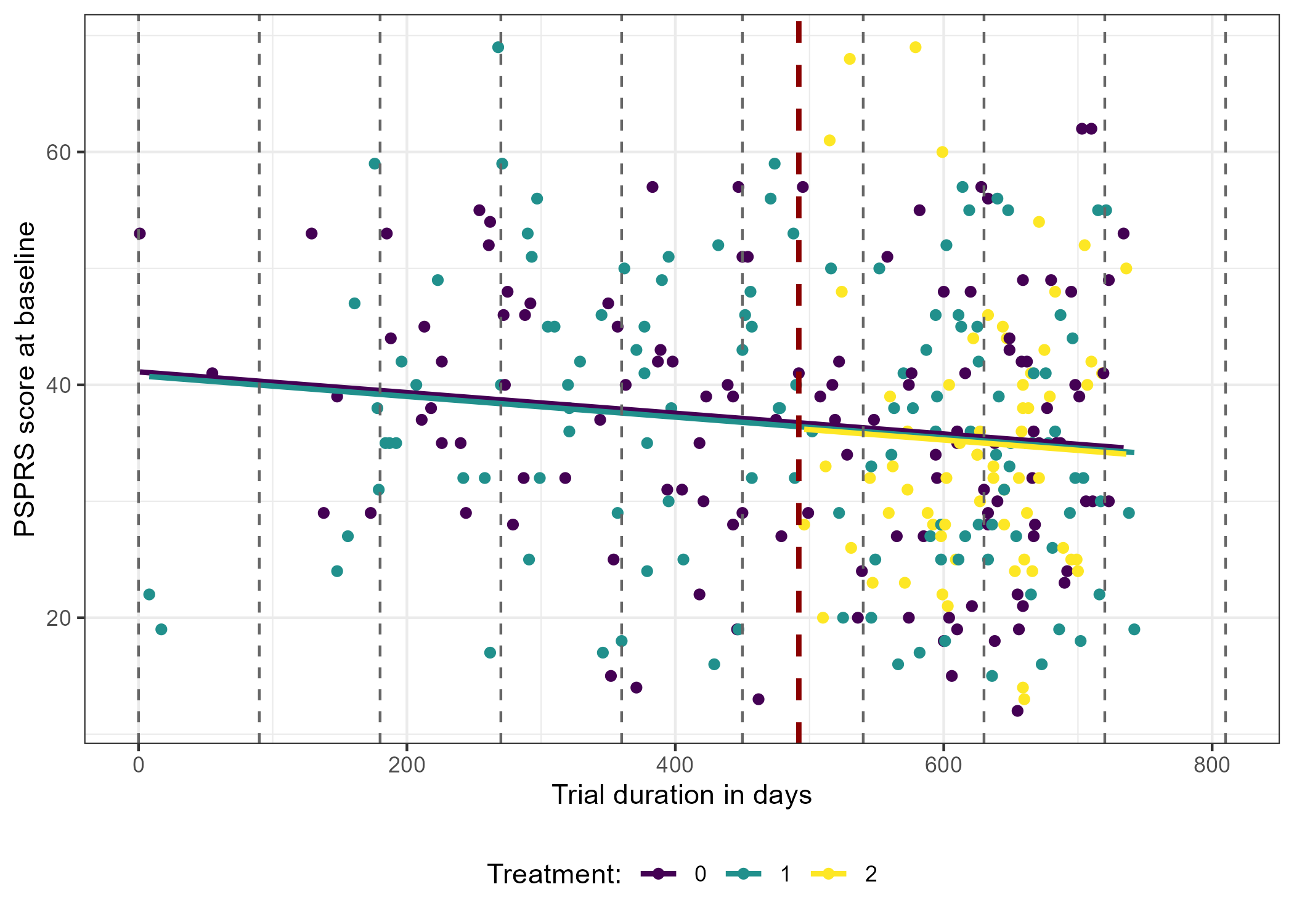

Supplement: Supplementary file 1 — Supporting Information [file BIMJ-67-e70059-s002.zip › case_studies/PSP/figures/casestudy_trialdata.png]

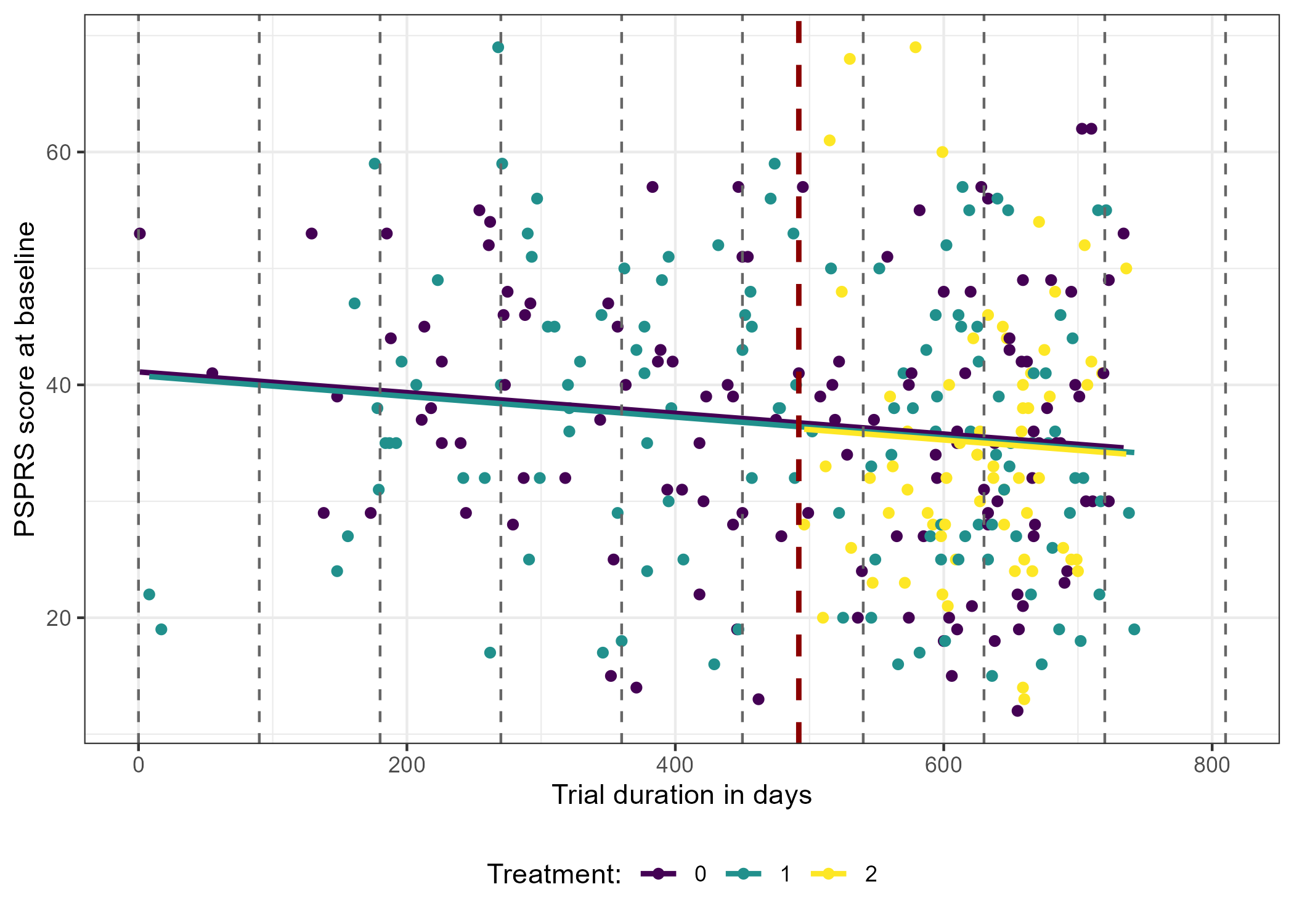

Supplement: Supplementary file 1 — Supporting Information [file BIMJ-67-e70059-s002.zip › case_studies/PSP/figures/casestudy_trialdata.tiff]

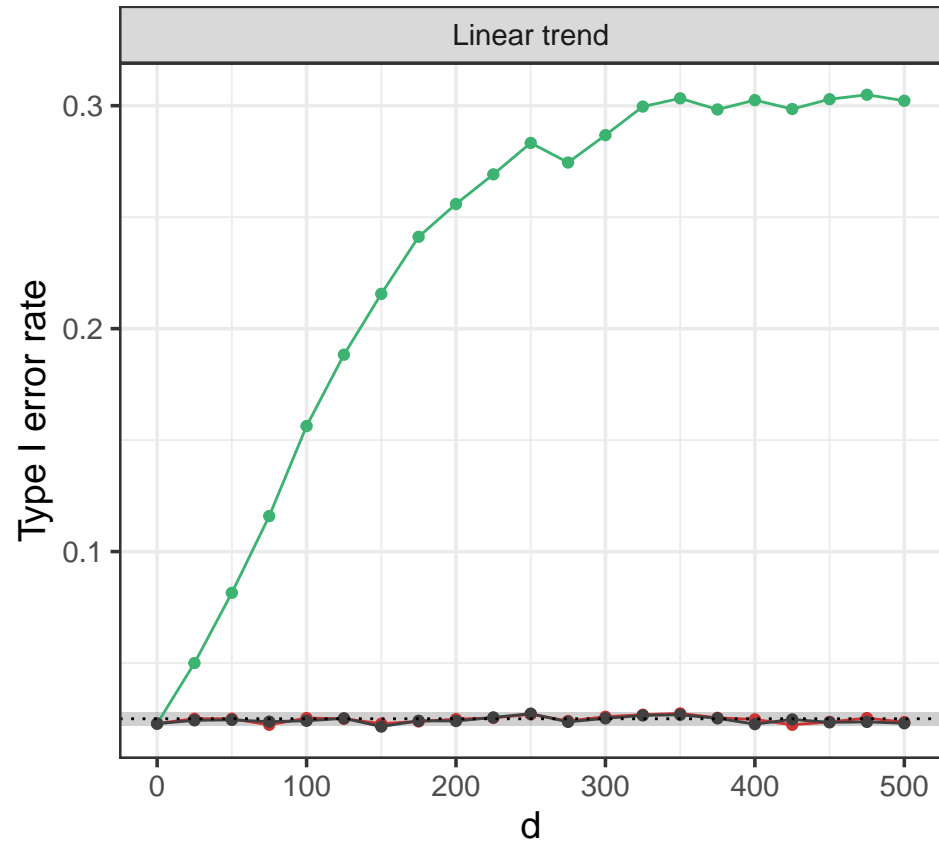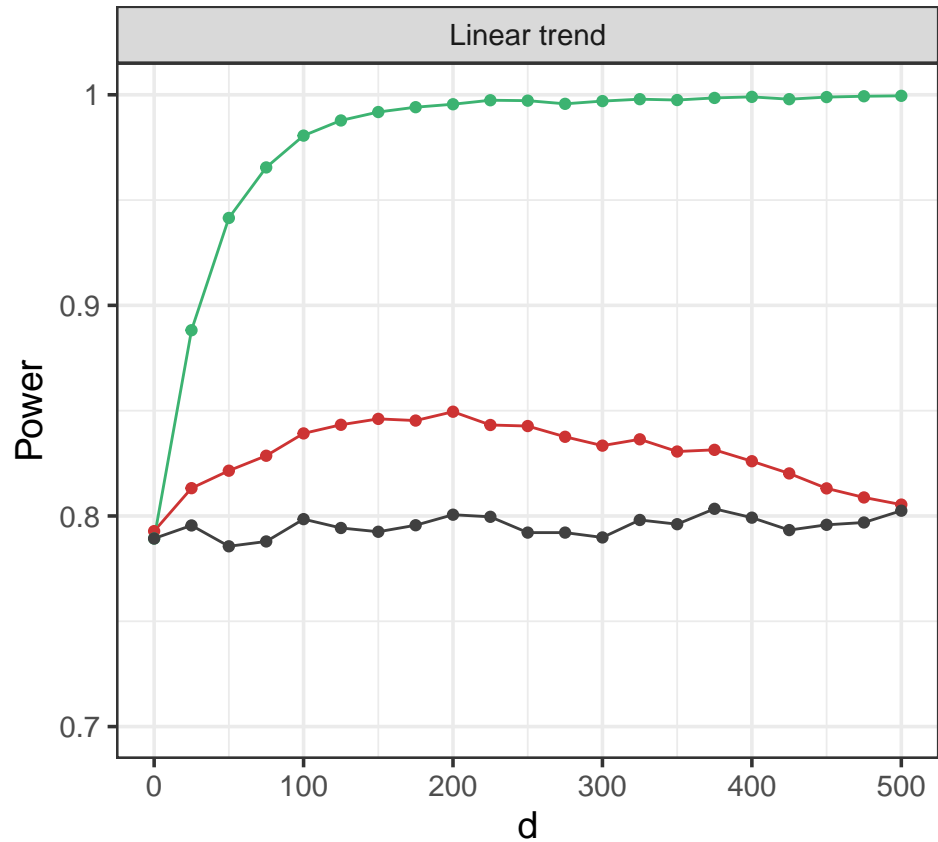

Analysis approach: —●— Fixed - period —●— Pooled analysis —●— Separate analysis

Supplement: Supplementary file 1 — Supporting Information [file BIMJ-67-e70059-s002.zip › simulations/figures/fixmodel_alpha_pow_d.pdf]

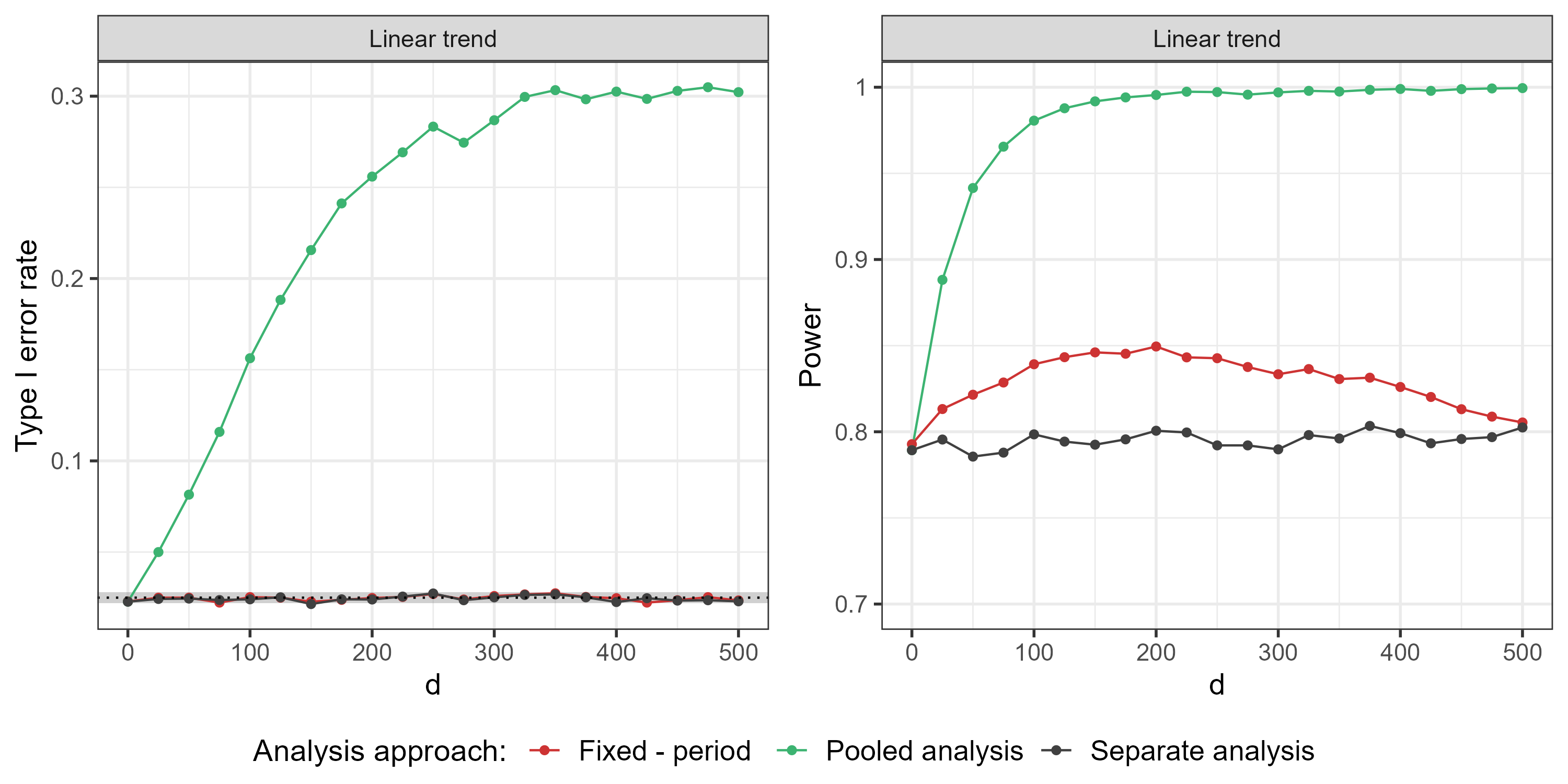

Supplement: Supplementary file 1 — Supporting Information [file BIMJ-67-e70059-s002.zip › simulations/figures/fixmodel_alpha_pow_d.png]

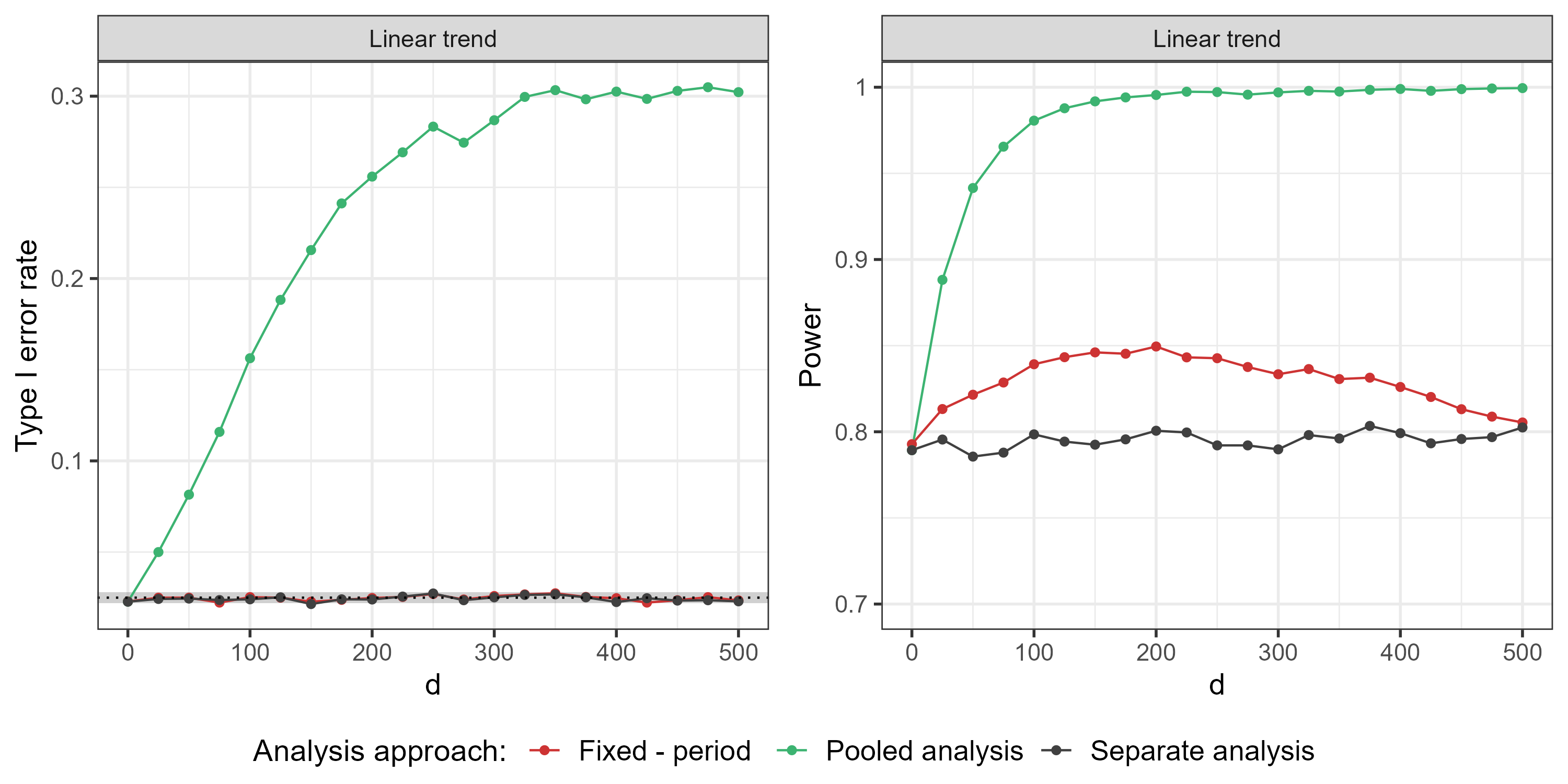

Supplement: Supplementary file 1 — Supporting Information [file BIMJ-67-e70059-s002.zip › simulations/figures/fixmodel_alpha_pow_d.tiff]

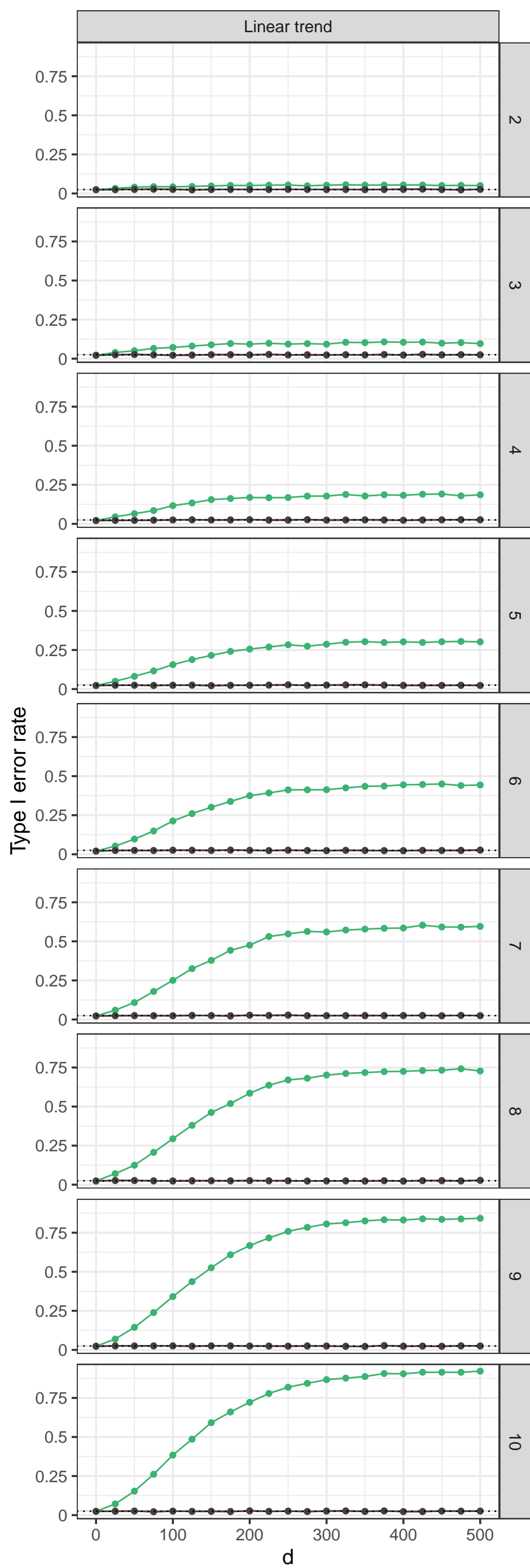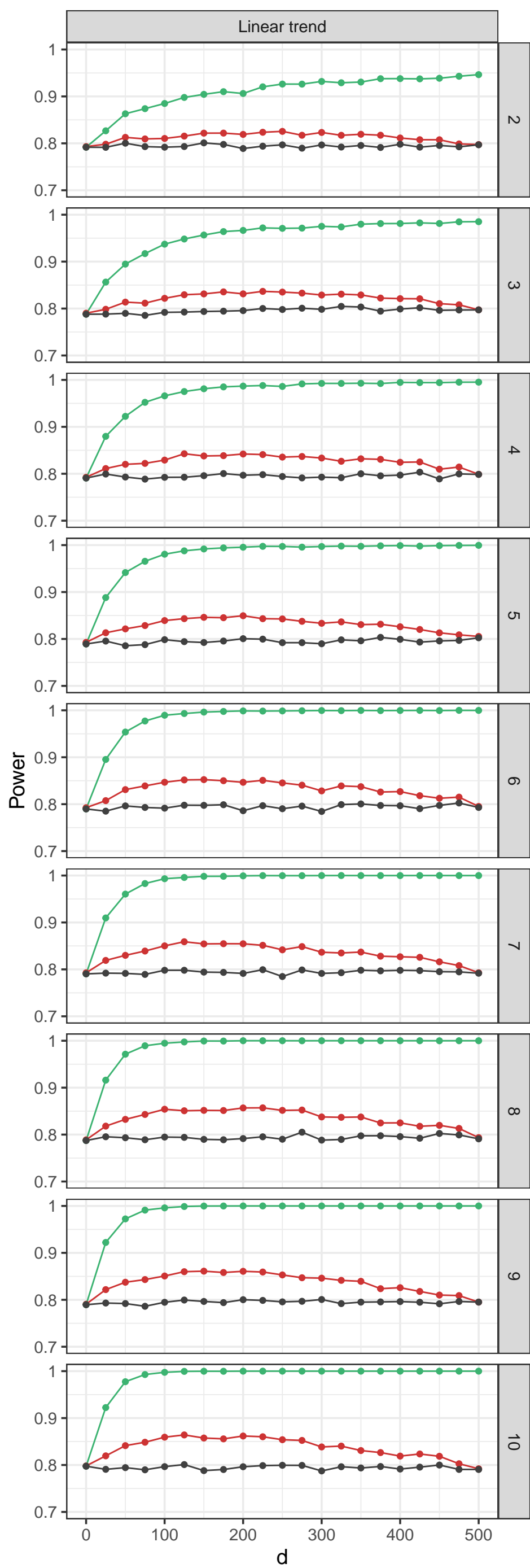

Analysis approach: Fixed – period Pooled analysis Separate analysis

Supplement: Supplementary file 1 — Supporting Information [file BIMJ-67-e70059-s002.zip › simulations/figures/fixmodel_alpha_pow_d_all_arms.pdf]

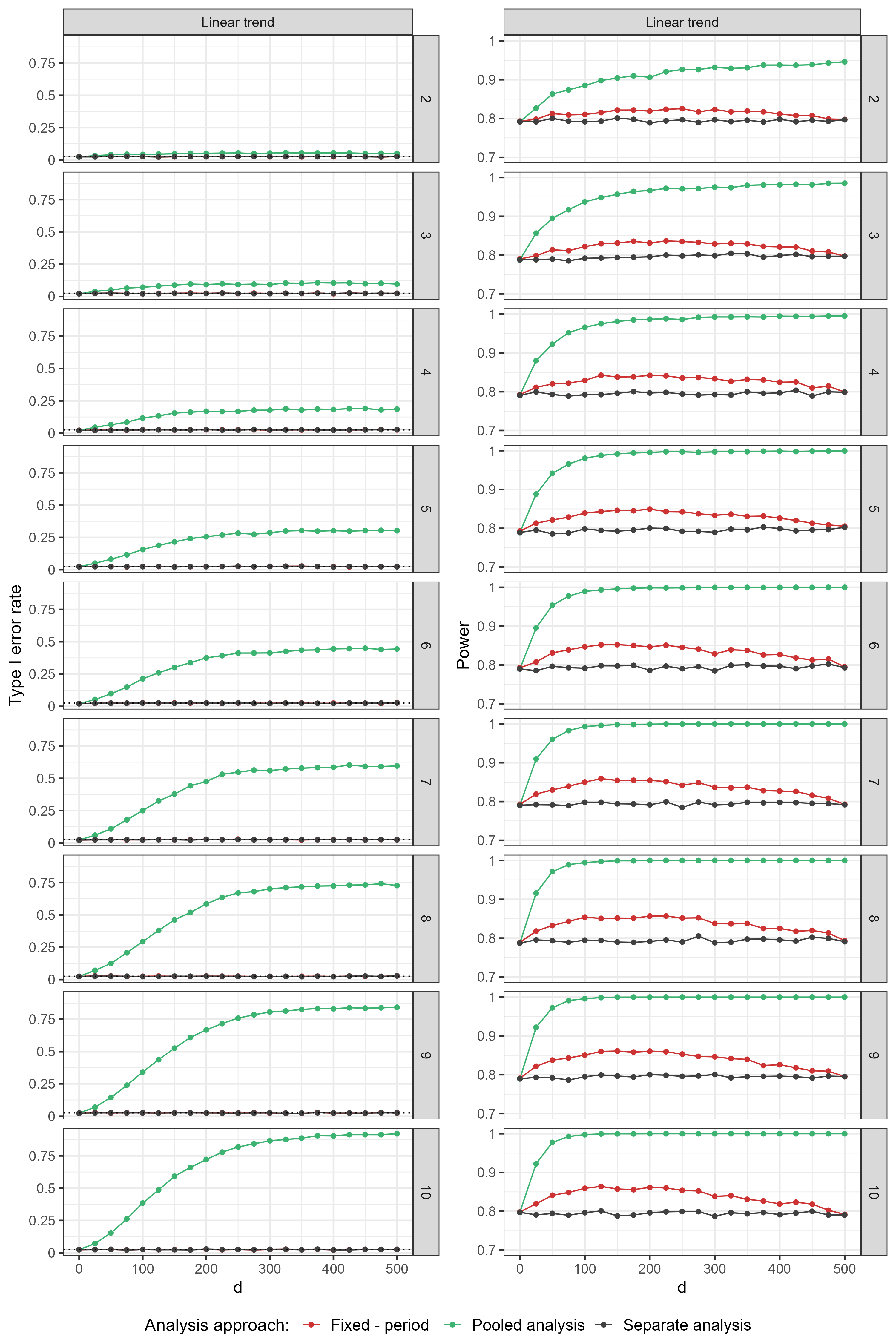

Supplement: Supplementary file 1 — Supporting Information [file BIMJ-67-e70059-s002.zip › simulations/figures/fixmodel_alpha_pow_d_all_arms.png]

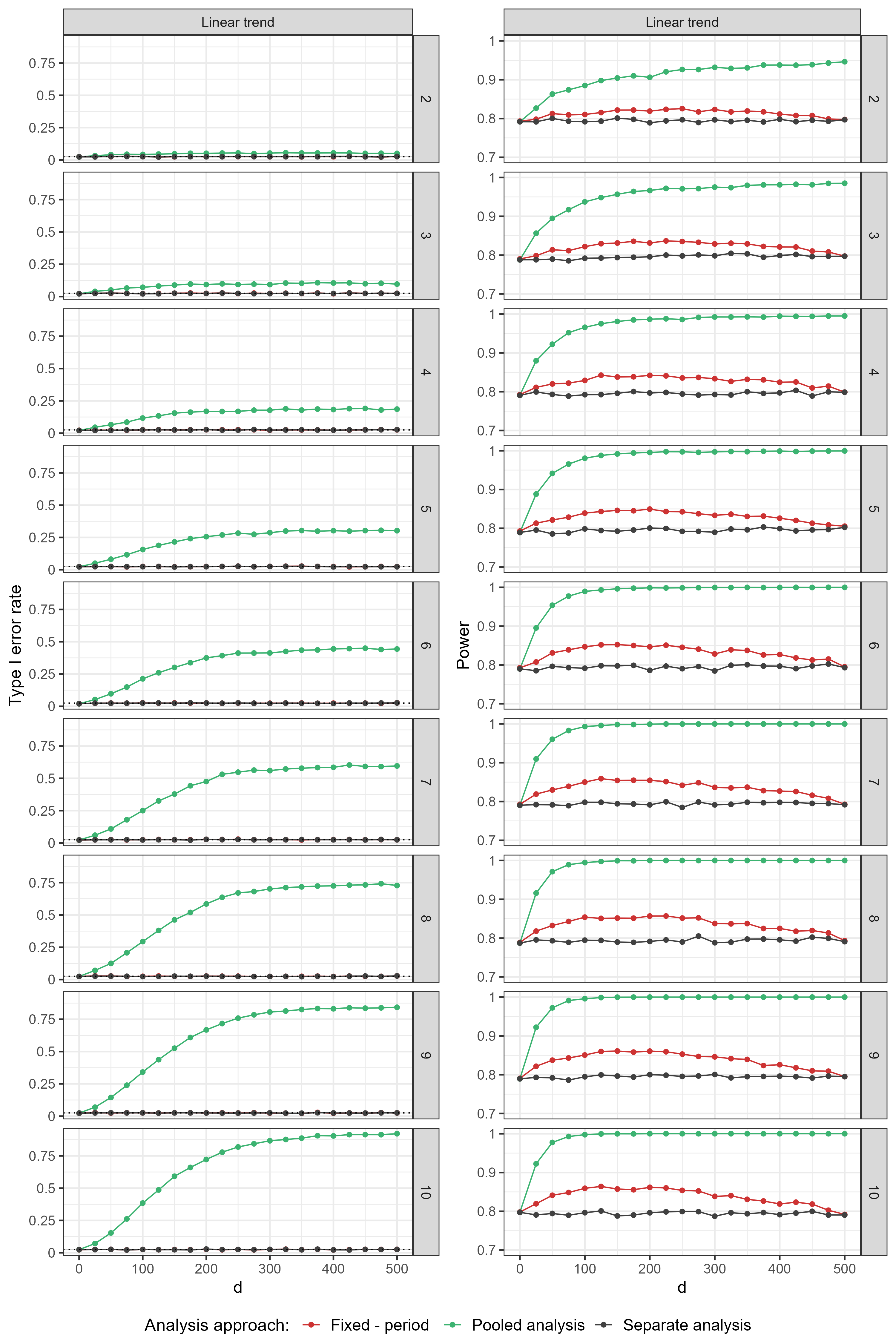

Supplement: Supplementary file 1 — Supporting Information [file BIMJ-67-e70059-s002.zip › simulations/figures/fixmodel_alpha_pow_d_all_arms.tiff]

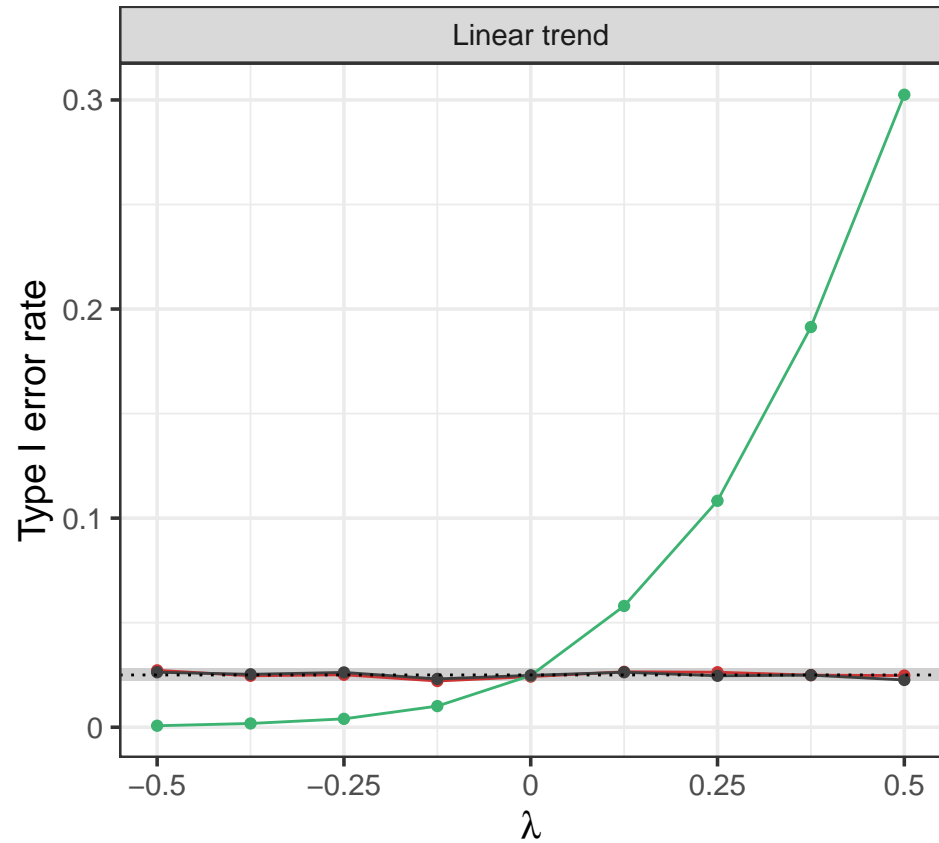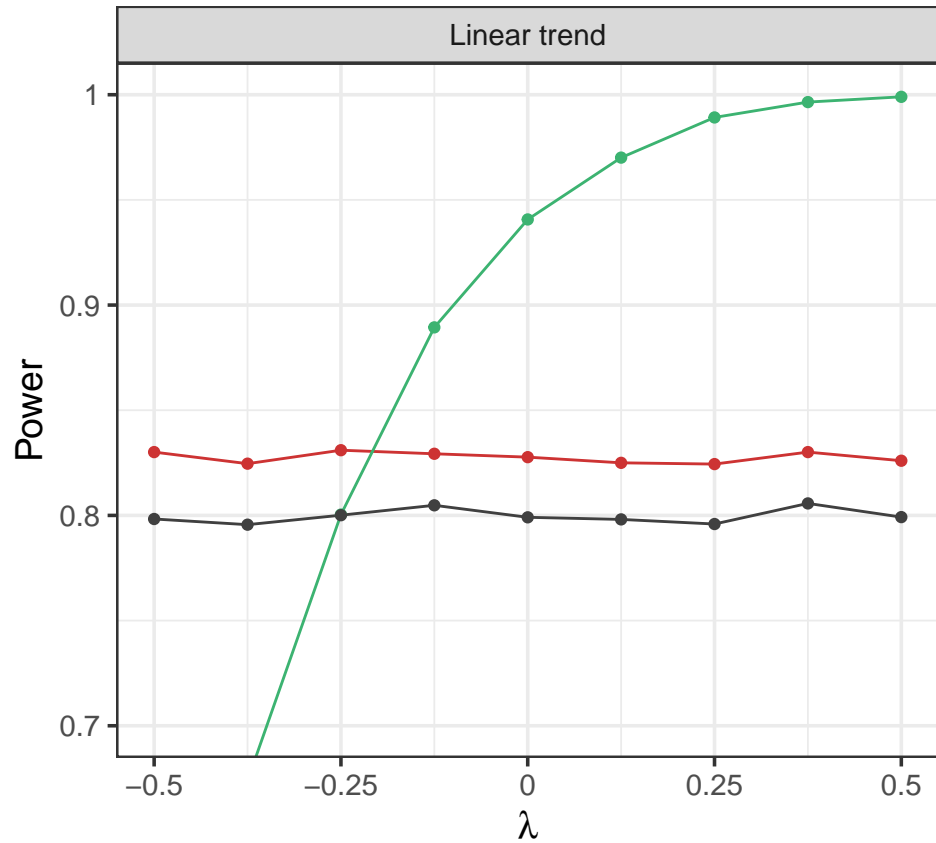

Analysis approach: —●— Fixed – period —●— Pooled analysis —●— Separate analysis

Supplement: Supplementary file 1 — Supporting Information [file BIMJ-67-e70059-s002.zip › simulations/figures/fixmodel_alpha_pow_lambda.pdf]

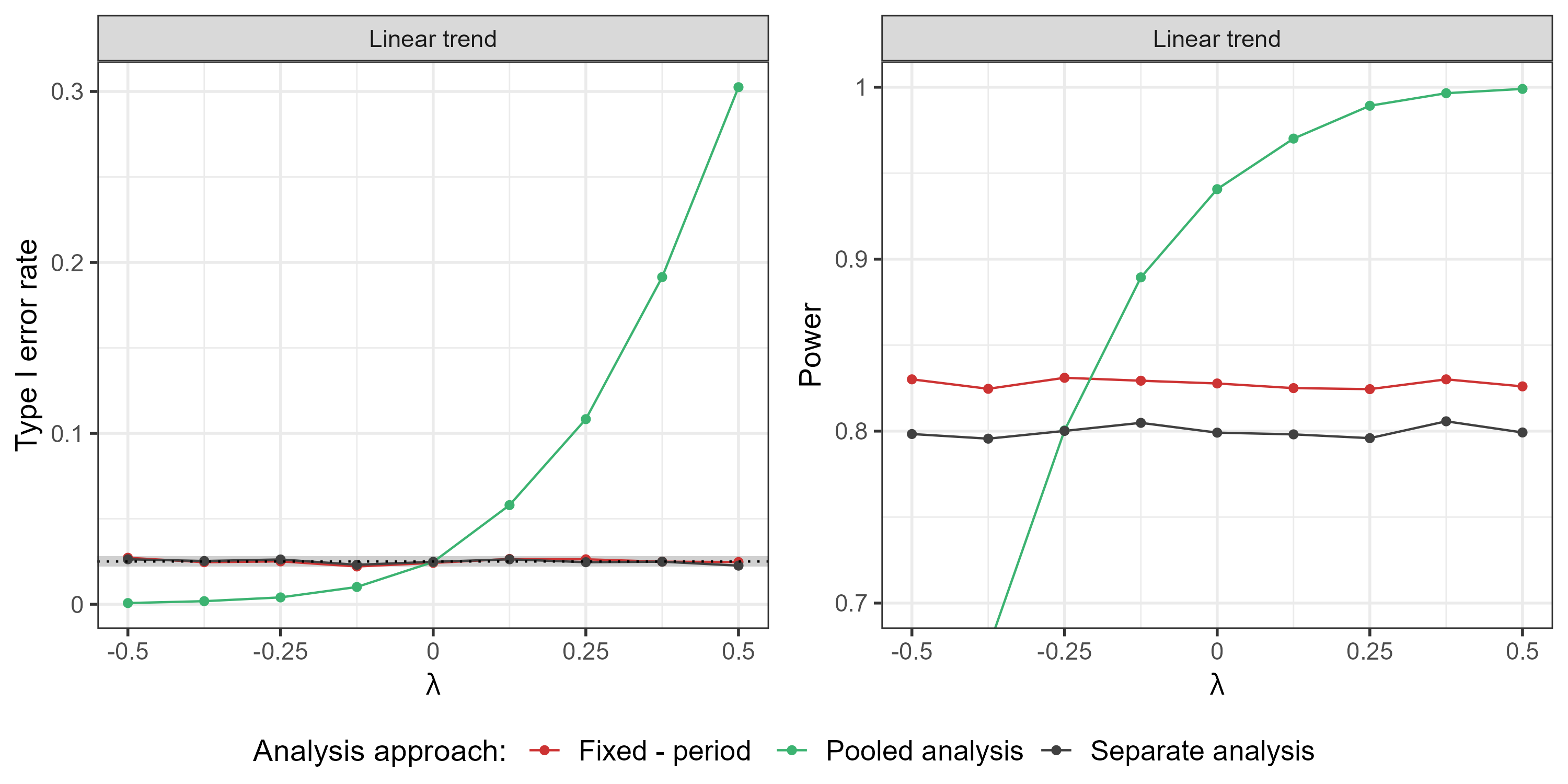

Supplement: Supplementary file 1 — Supporting Information [file BIMJ-67-e70059-s002.zip › simulations/figures/fixmodel_alpha_pow_lambda.png]

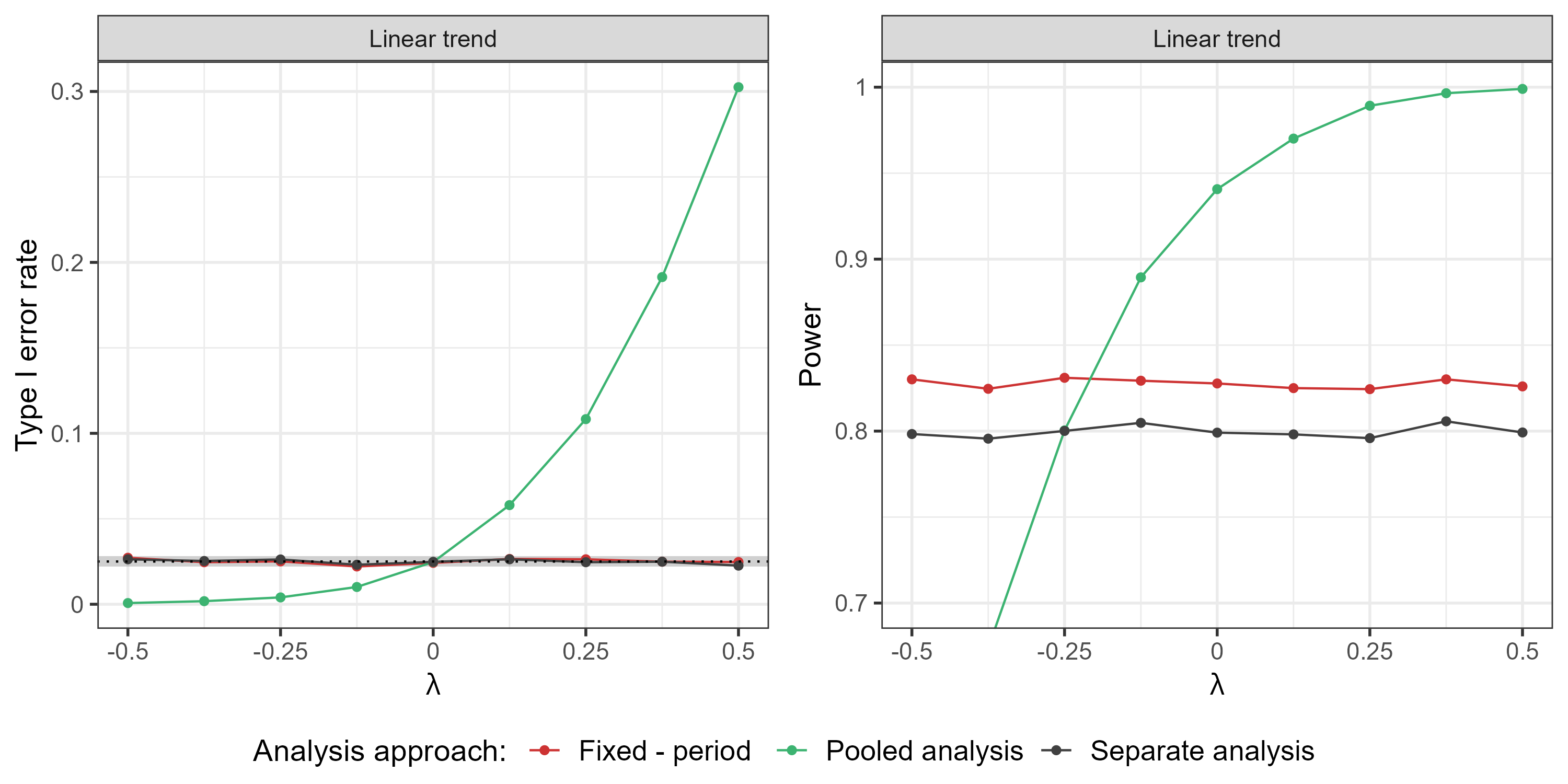

Supplement: Supplementary file 1 — Supporting Information [file BIMJ-67-e70059-s002.zip › simulations/figures/fixmodel_alpha_pow_lambda.tiff]

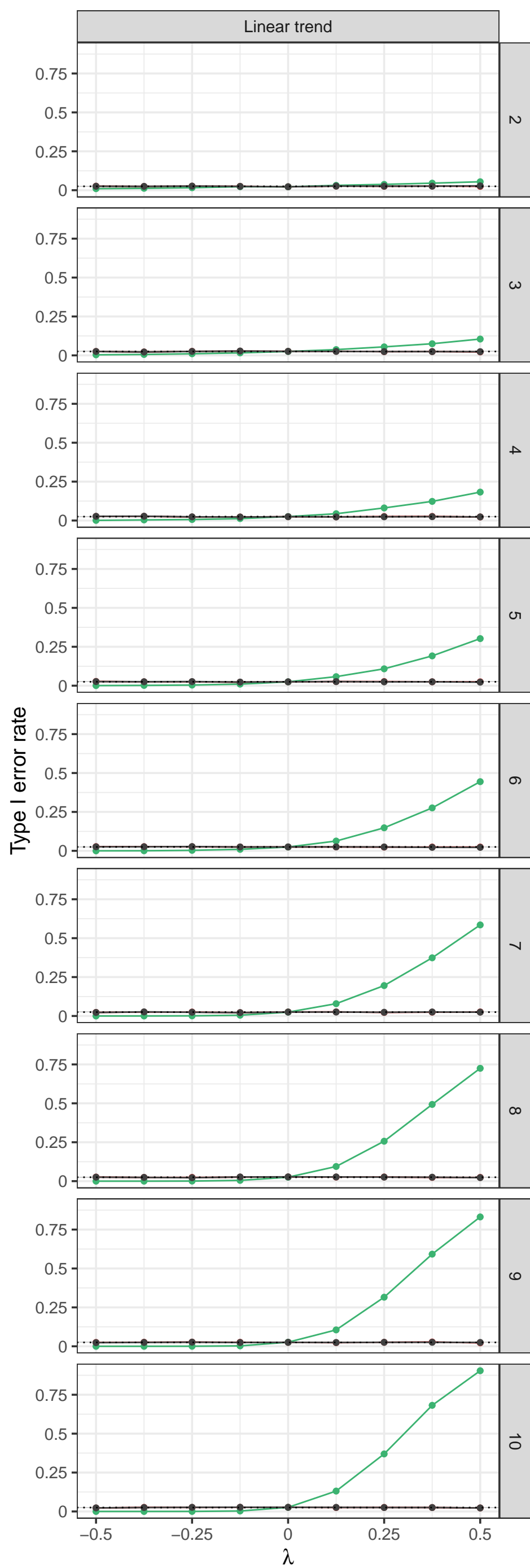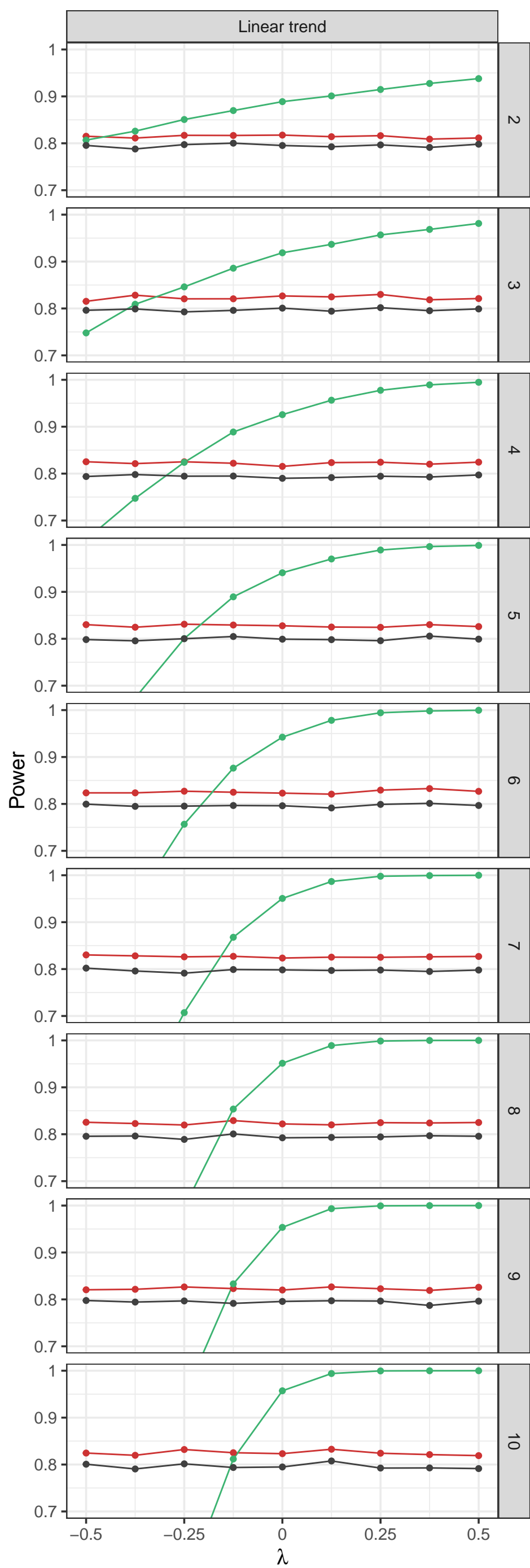

Analysis approach: Fixed – period Pooled analysis Separate analysis

Supplement: Supplementary file 1 — Supporting Information [file BIMJ-67-e70059-s002.zip › simulations/figures/fixmodel_alpha_pow_lambda_all_arms.pdf]

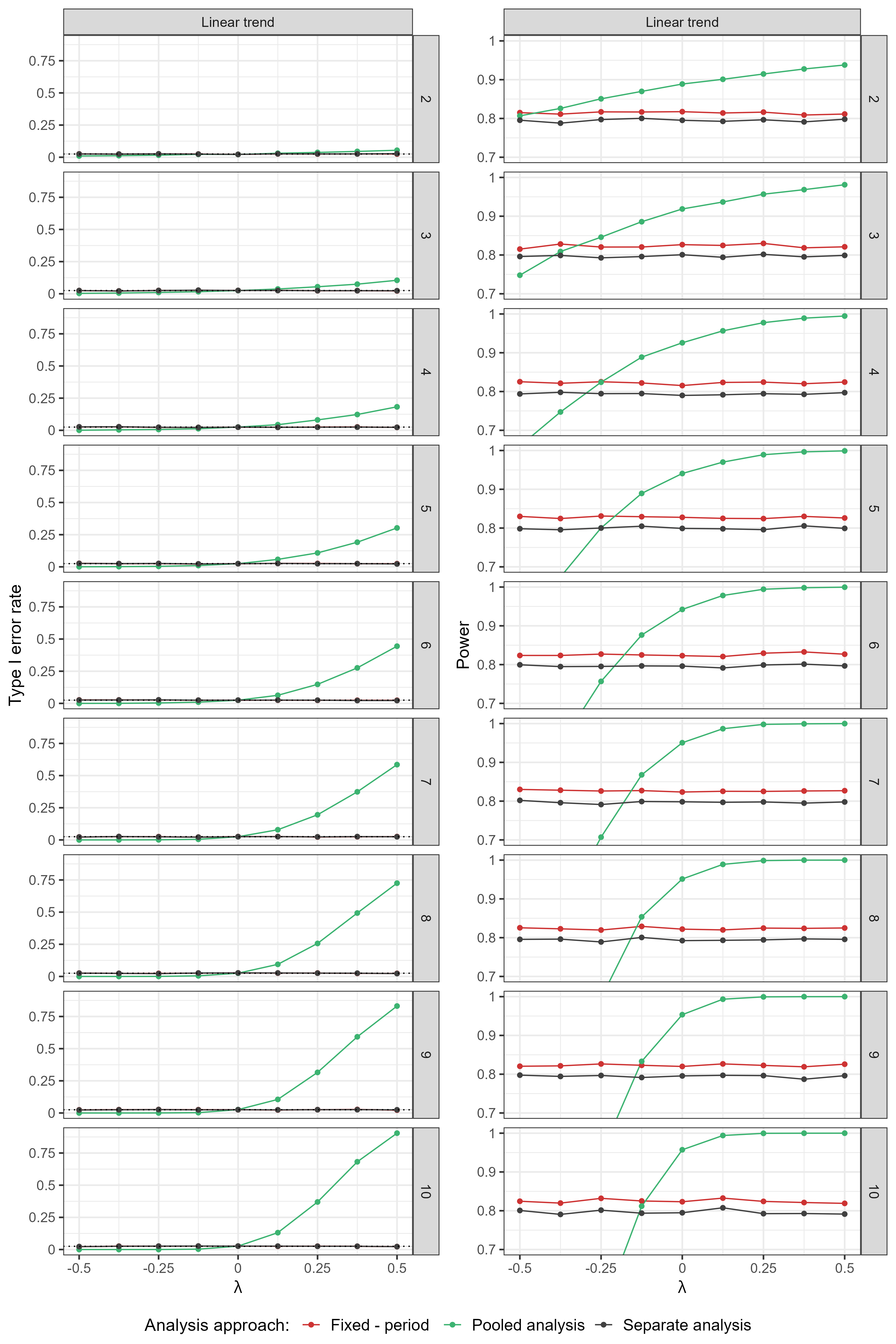

Supplement: Supplementary file 1 — Supporting Information [file BIMJ-67-e70059-s002.zip › simulations/figures/fixmodel_alpha_pow_lambda_all_arms.png]

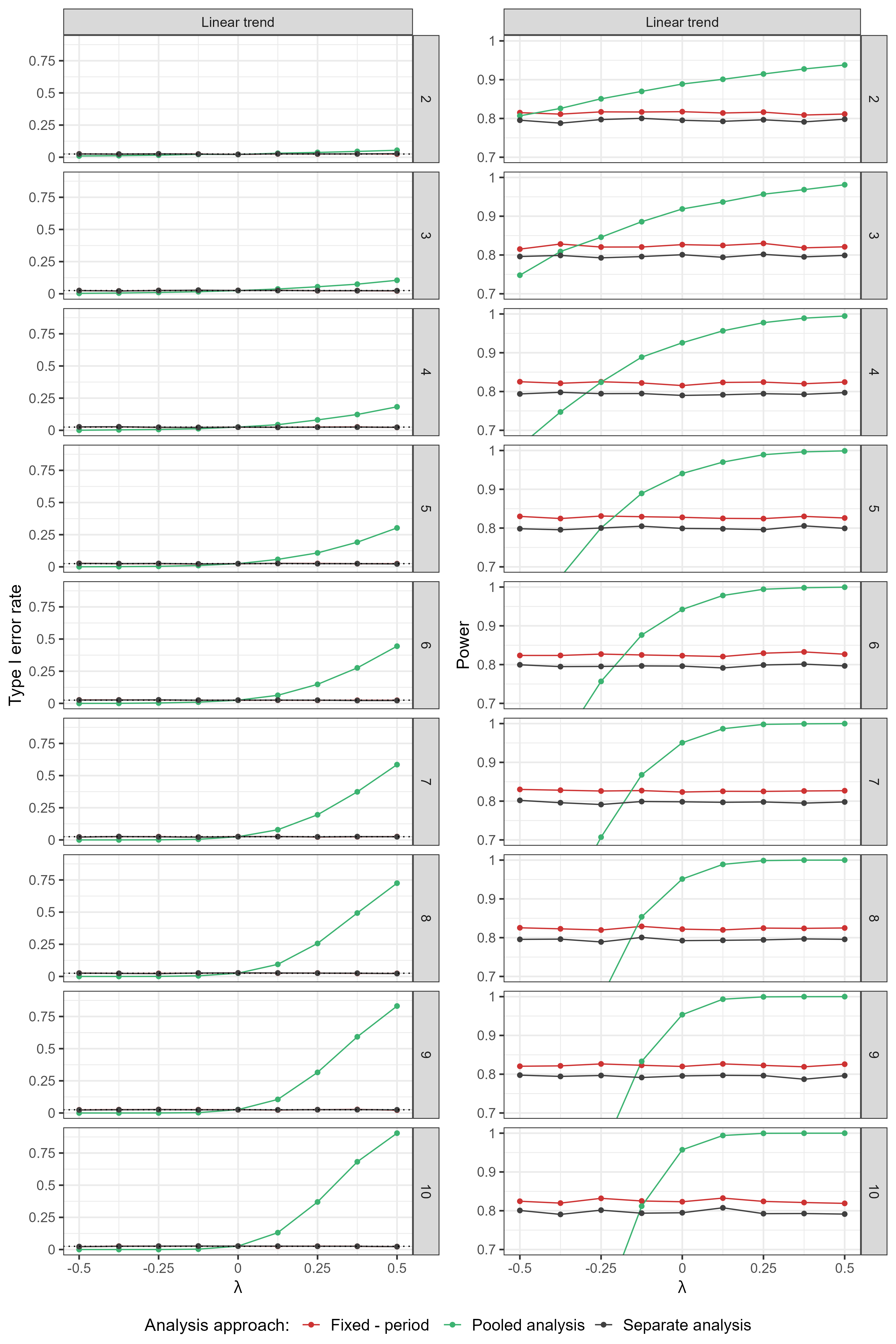

Supplement: Supplementary file 1 — Supporting Information [file BIMJ-67-e70059-s002.zip › simulations/figures/fixmodel_alpha_pow_lambda_all_arms.tiff]

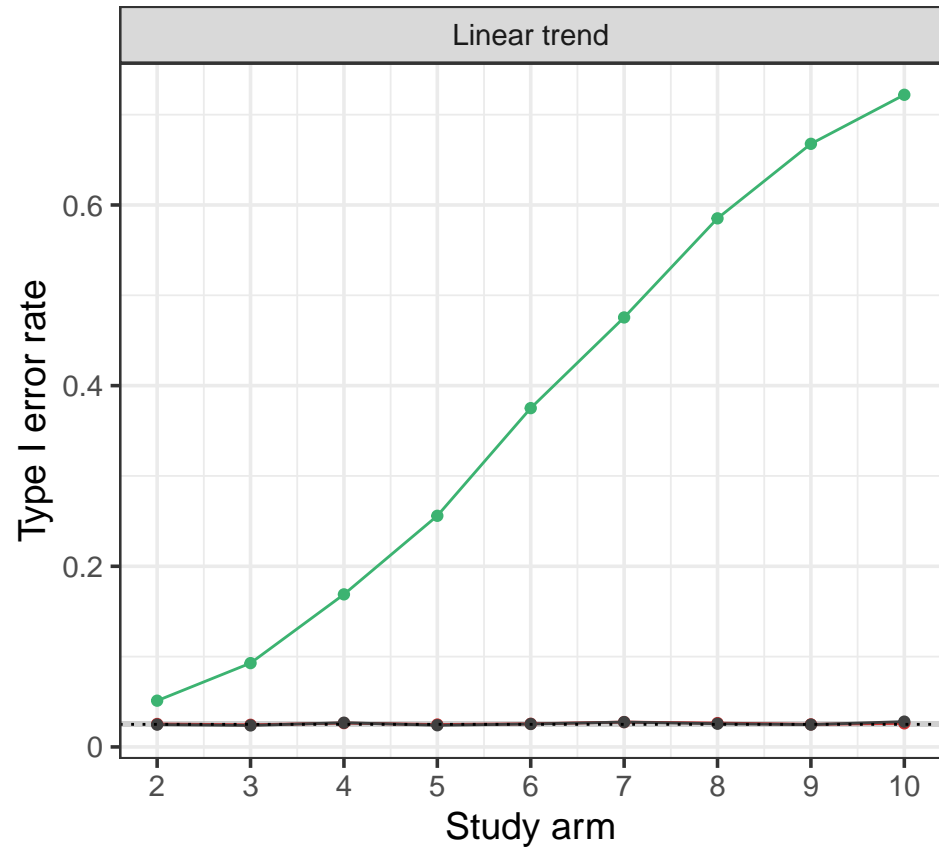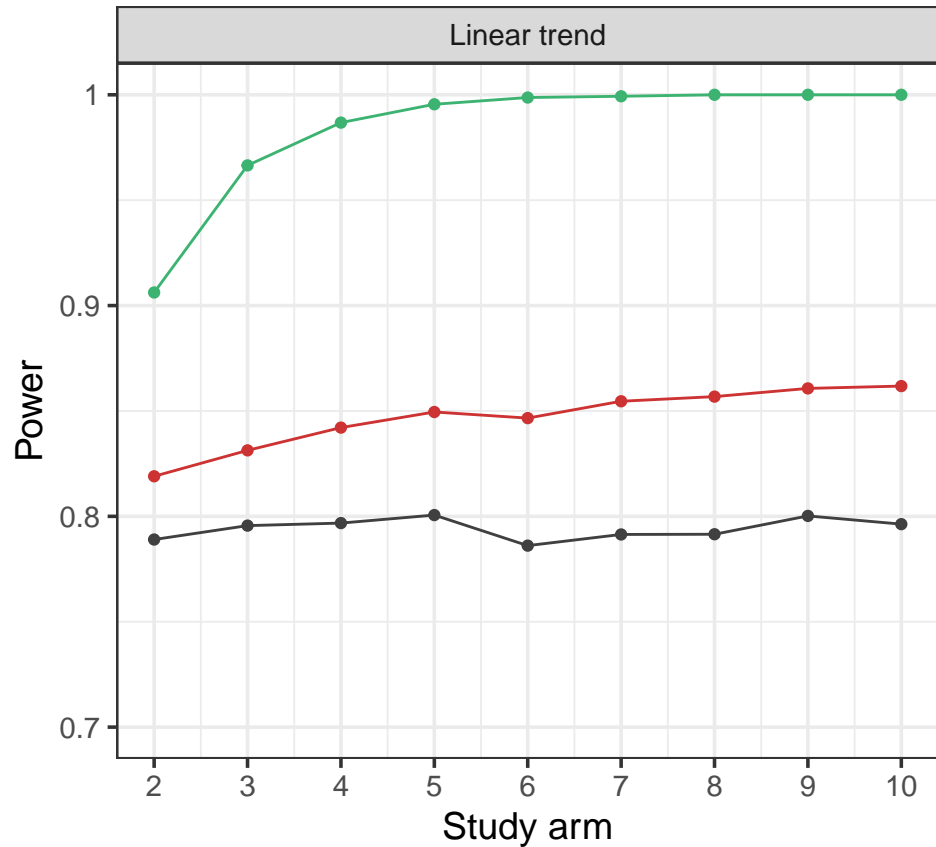

Analysis approach: Fixed - period Pooled analysis Separate analysis

Supplement: Supplementary file 1 — Supporting Information [file BIMJ-67-e70059-s002.zip › simulations/figures/fixmodel_alpha_pow_study_arm.pdf]

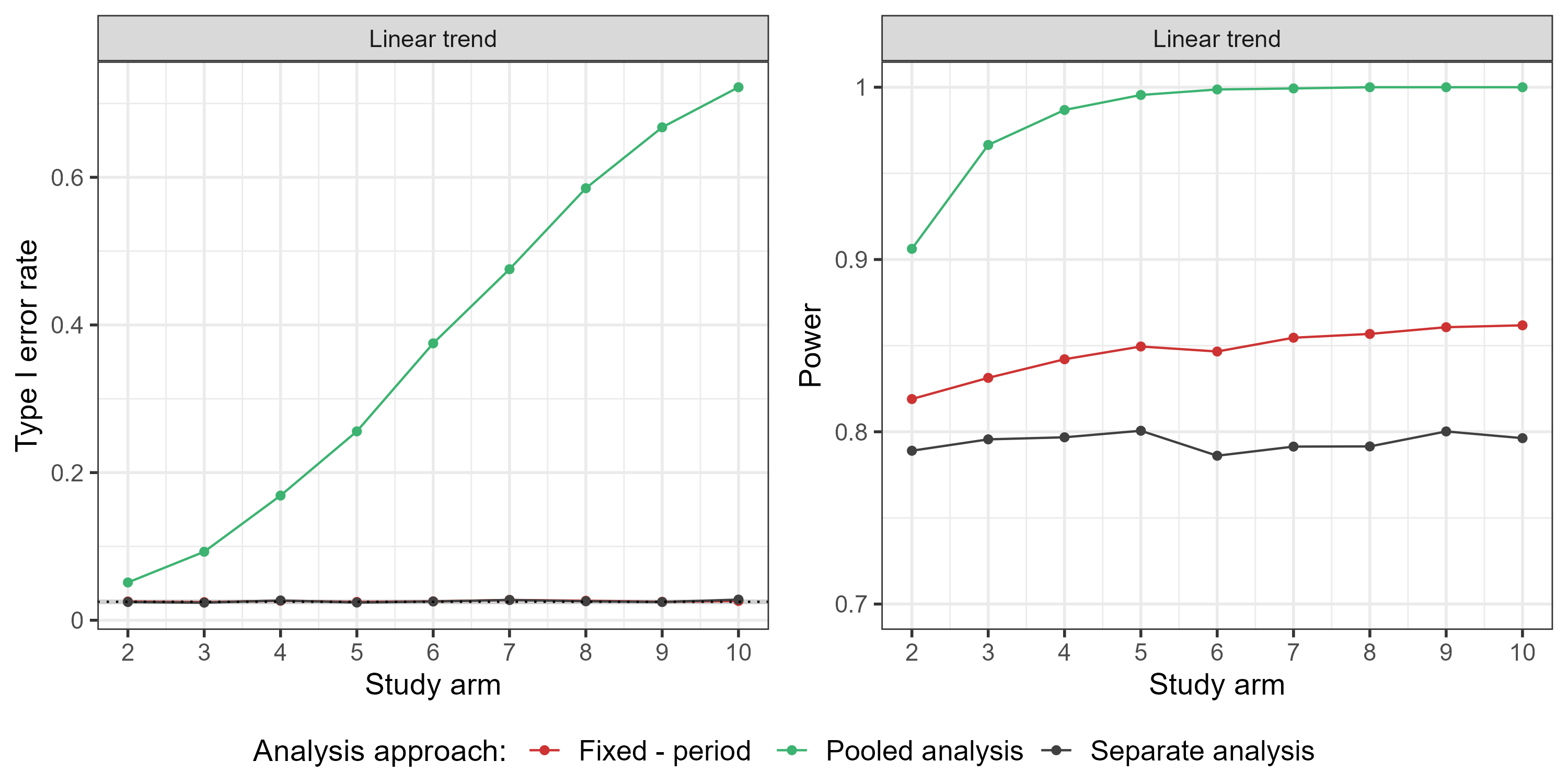

Supplement: Supplementary file 1 — Supporting Information [file BIMJ-67-e70059-s002.zip › simulations/figures/fixmodel_alpha_pow_study_arm.png]

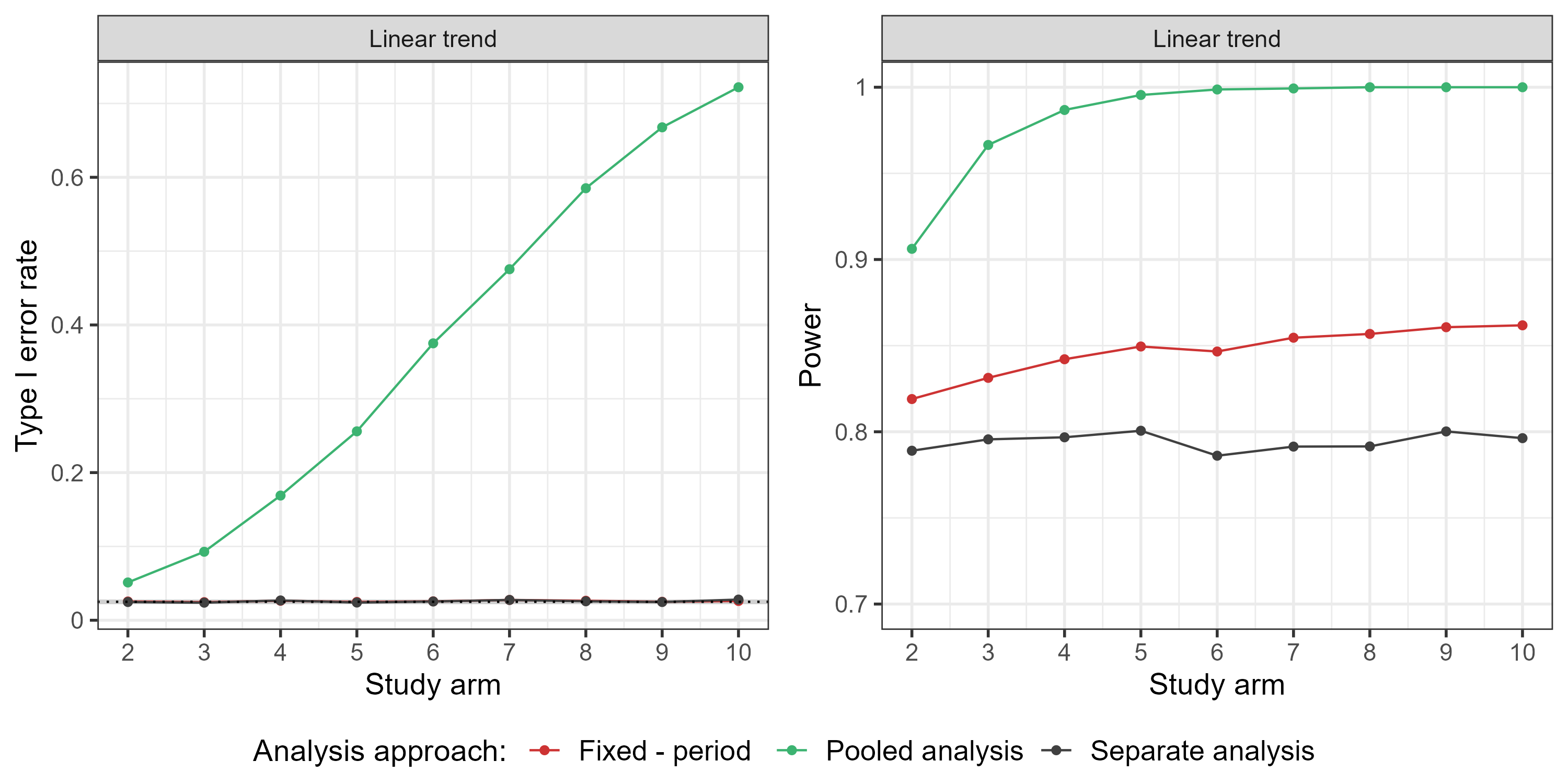

Supplement: Supplementary file 1 — Supporting Information [file BIMJ-67-e70059-s002.zip › simulations/figures/fixmodel_alpha_pow_study_arm.tiff]

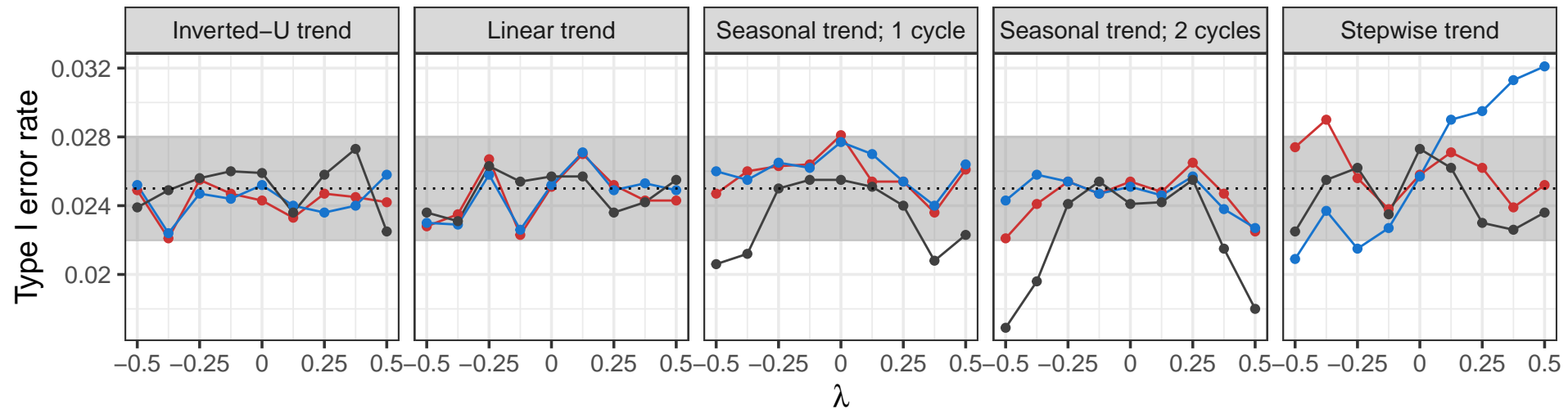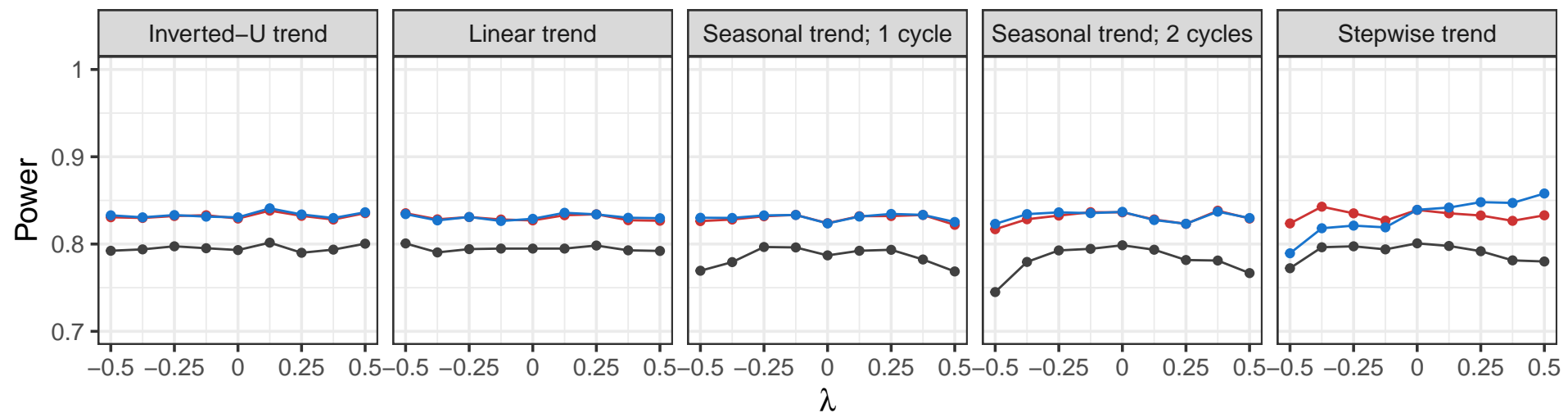

Analysis approach: —●— Fixed - period —●— Fixed - calendar —●— Separate analysis

Supplement: Supplementary file 1 — Supporting Information [file BIMJ-67-e70059-s002.zip › simulations/figures/fixmodel_cal_alpha_pow_lambda.pdf]

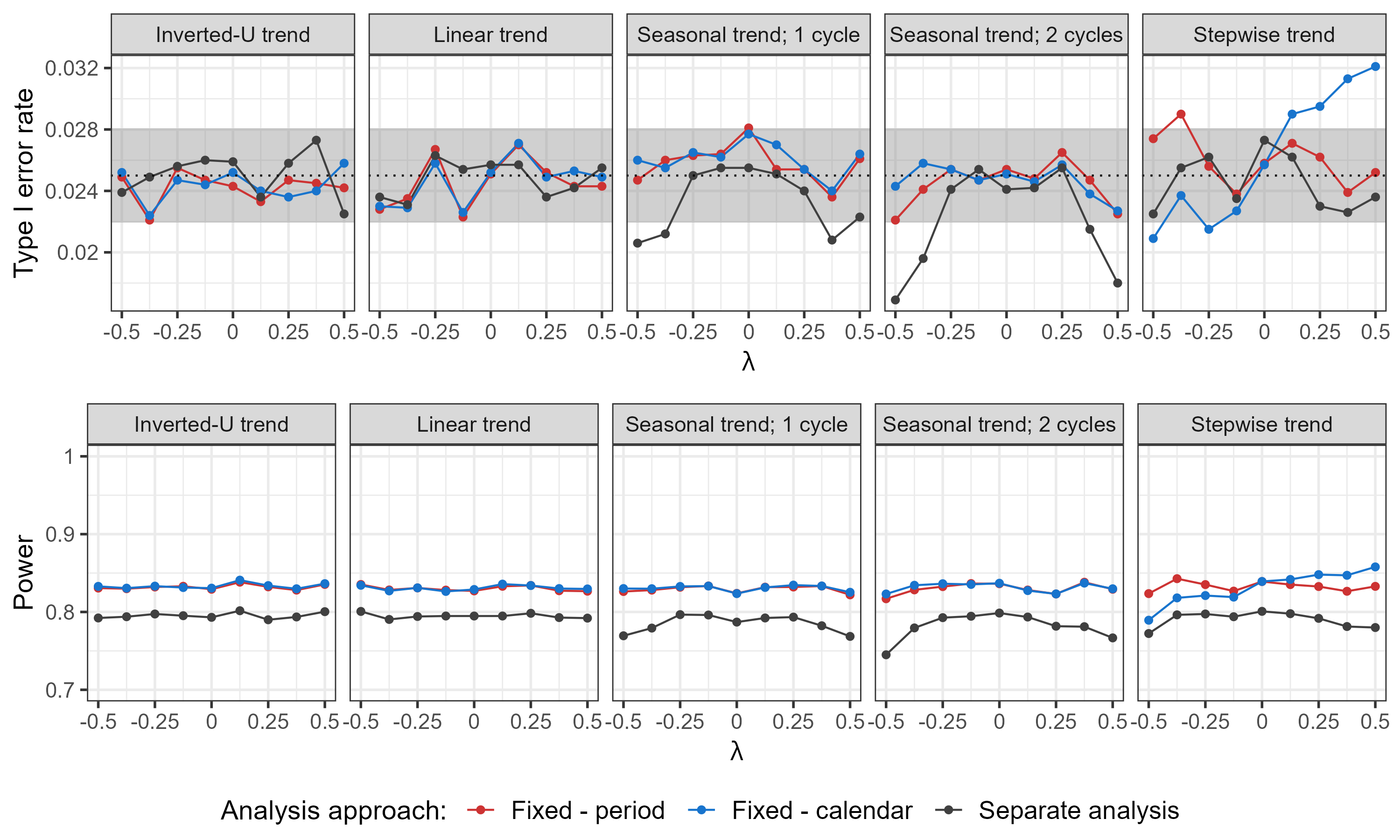

Supplement: Supplementary file 1 — Supporting Information [file BIMJ-67-e70059-s002.zip › simulations/figures/fixmodel_cal_alpha_pow_lambda.png]

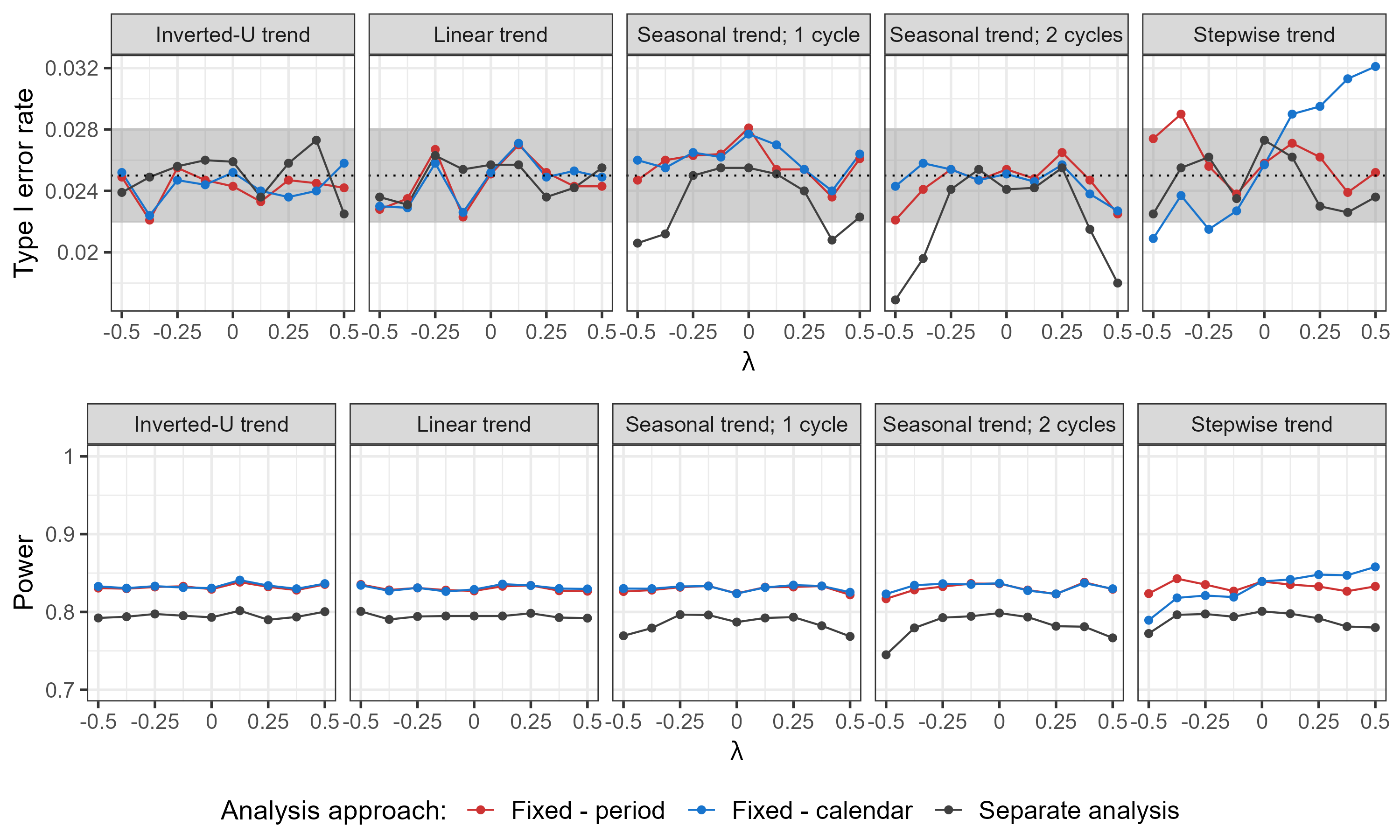

Supplement: Supplementary file 1 — Supporting Information [file BIMJ-67-e70059-s002.zip › simulations/figures/fixmodel_cal_alpha_pow_lambda.tiff]

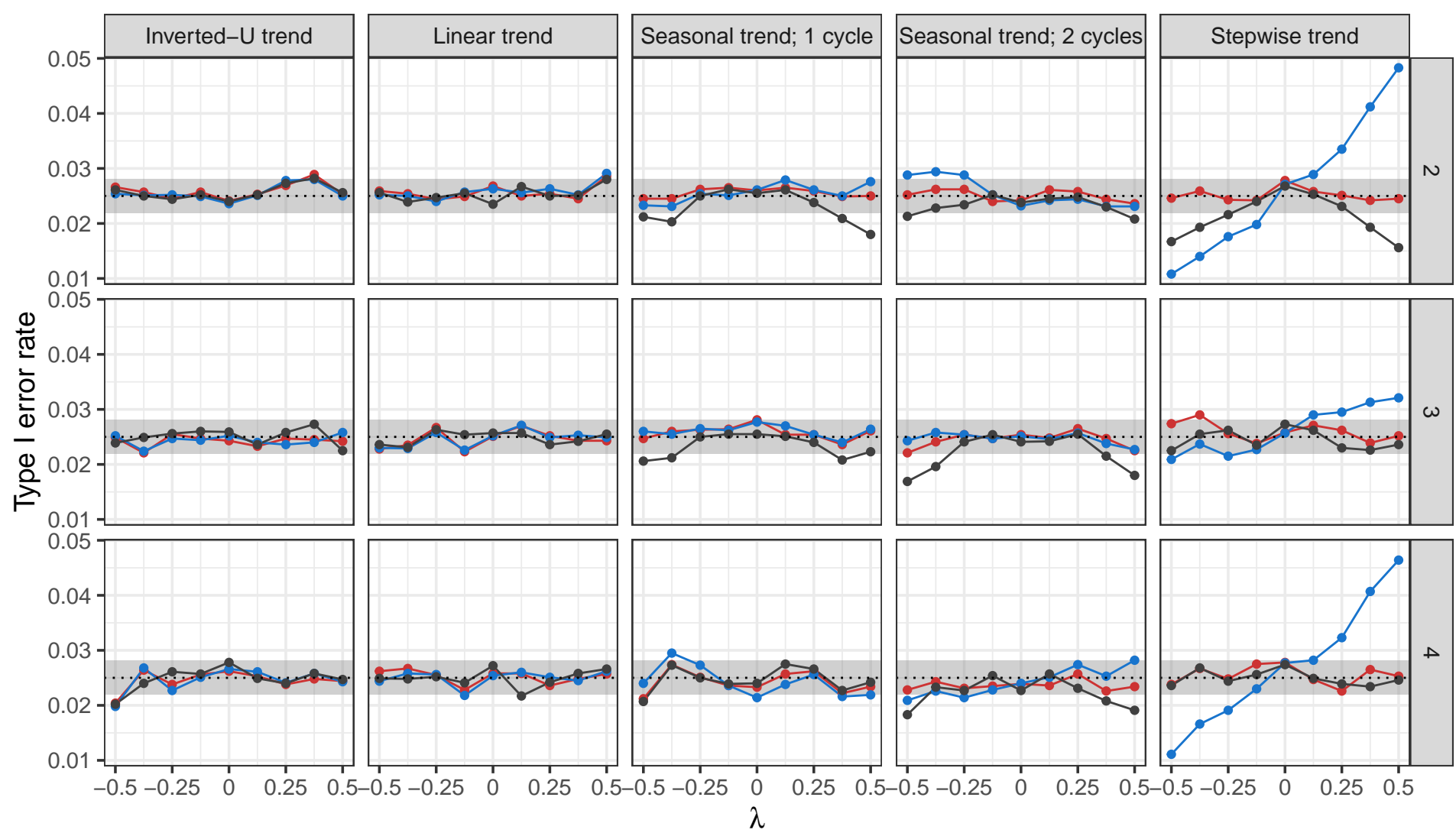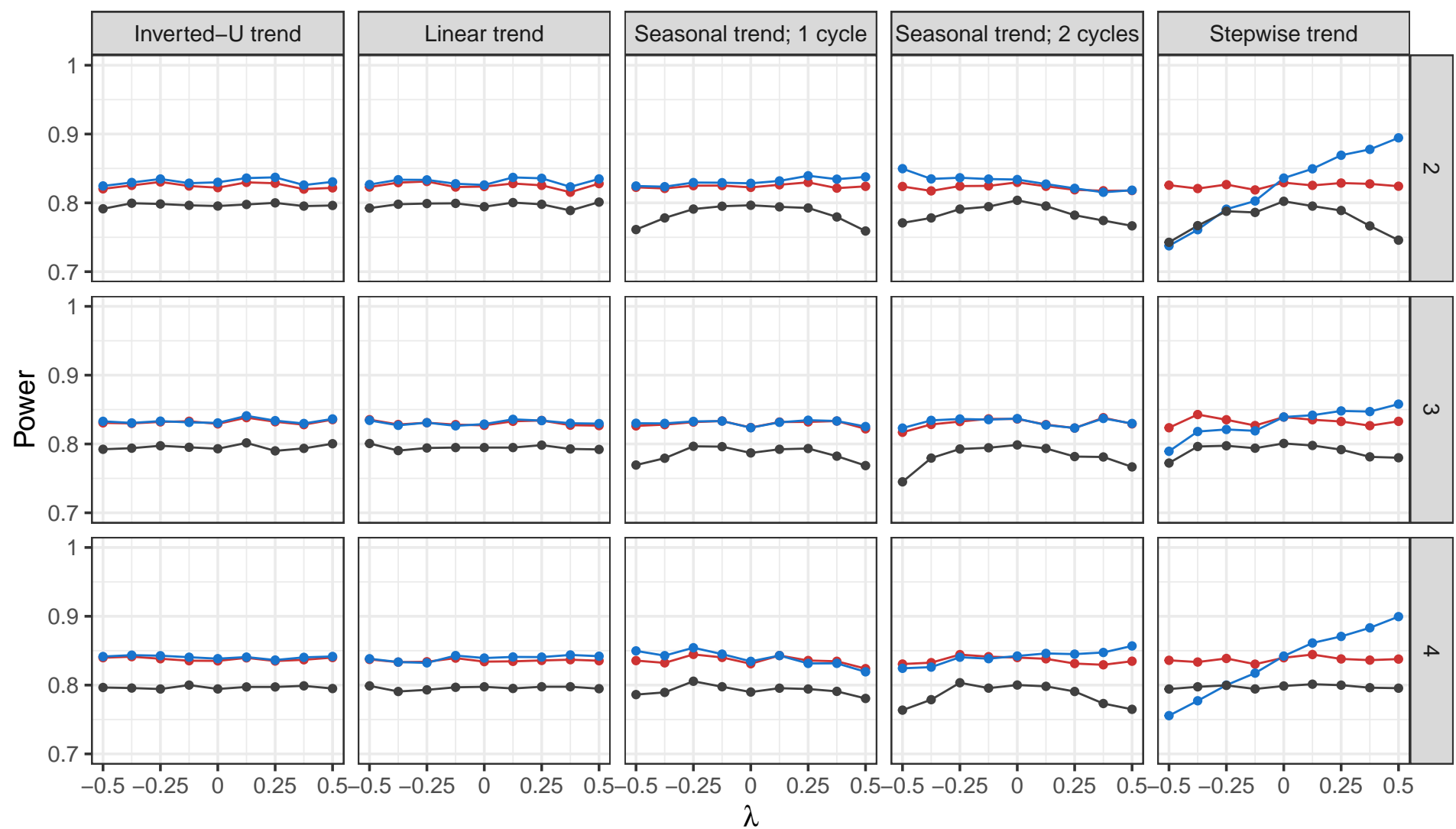

Analysis approach: —●— Fixed – period —●— Fixed – calendar —●— Separate analysis

Supplement: Supplementary file 1 — Supporting Information [file BIMJ-67-e70059-s002.zip › simulations/figures/fixmodel_cal_alpha_pow_lambda_all_arms.pdf]

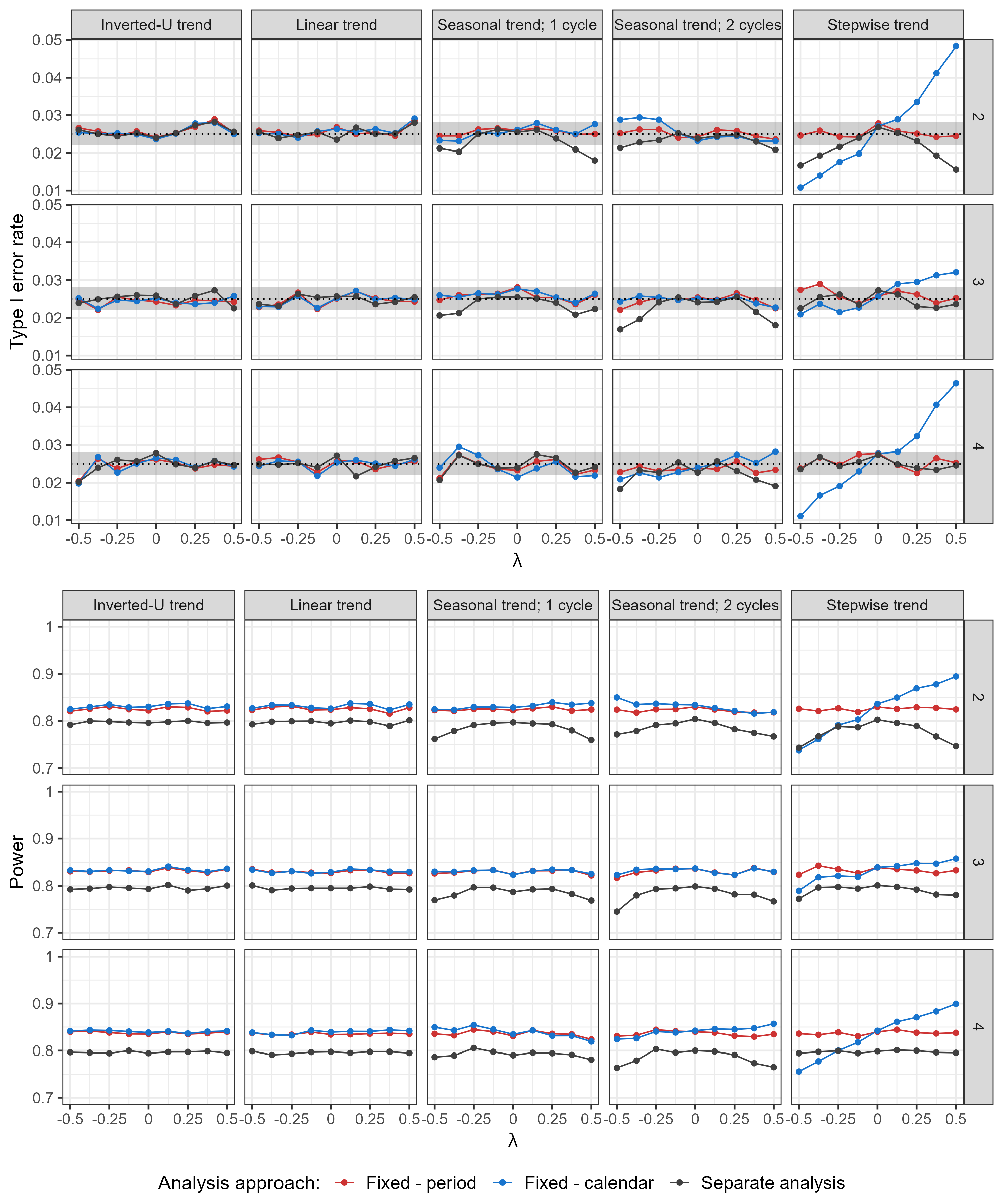

Supplement: Supplementary file 1 — Supporting Information [file BIMJ-67-e70059-s002.zip › simulations/figures/fixmodel_cal_alpha_pow_lambda_all_arms.png]

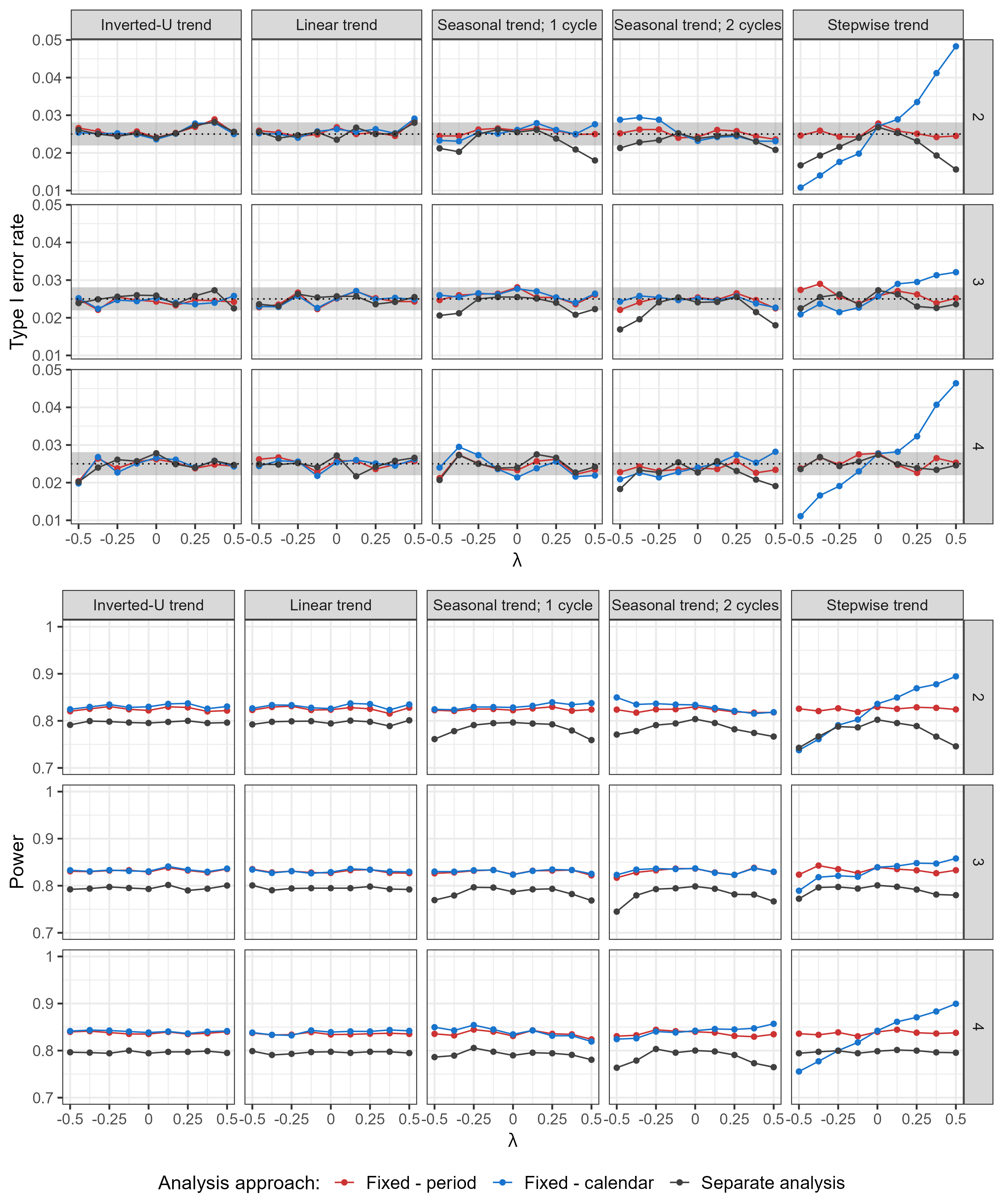

Supplement: Supplementary file 1 — Supporting Information [file BIMJ-67-e70059-s002.zip › simulations/figures/fixmodel_cal_alpha_pow_lambda_all_arms.tiff]

Type I error rate

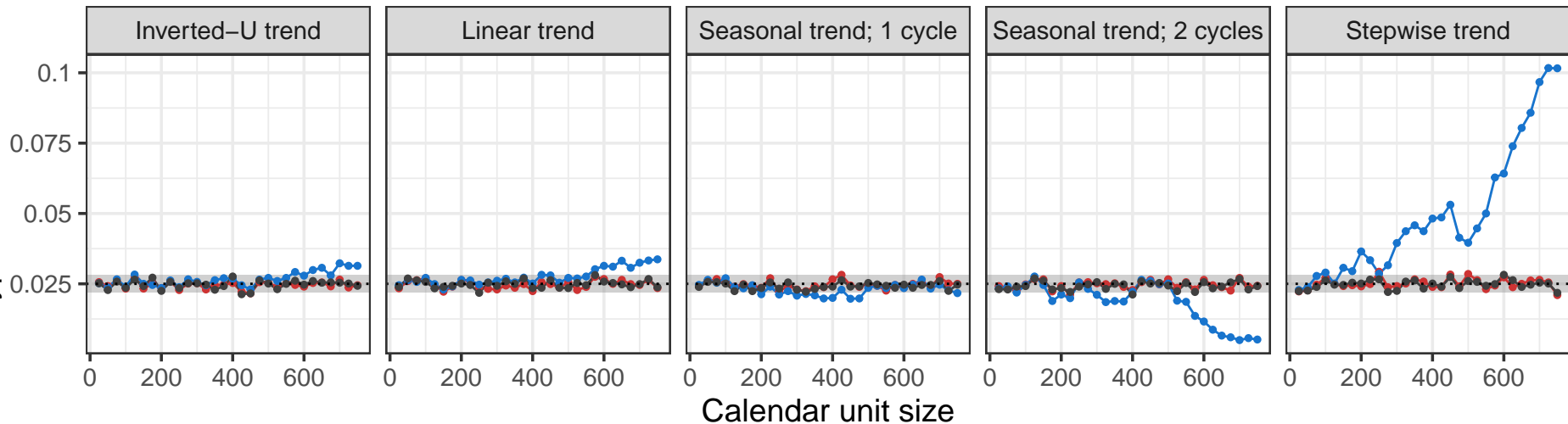

Power

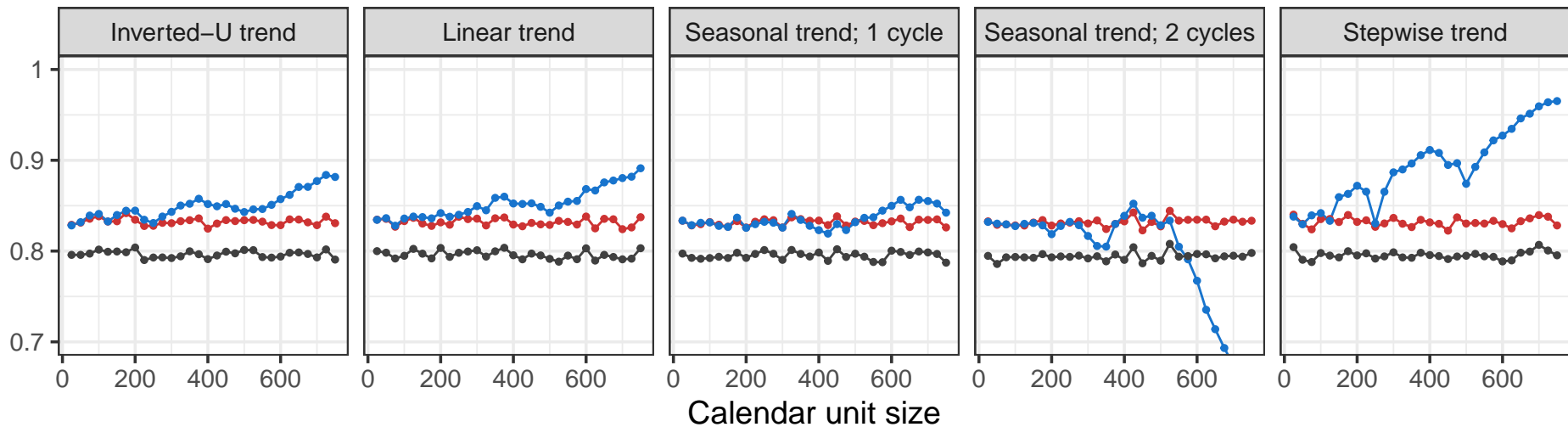

Analysis approach: — Fixed - period — Fixed - calendar — Separate analysis

Supplement: Supplementary file 1 — Supporting Information [file BIMJ-67-e70059-s002.zip › simulations/figures/fixmodel_cal_alpha_pow_unit.pdf]

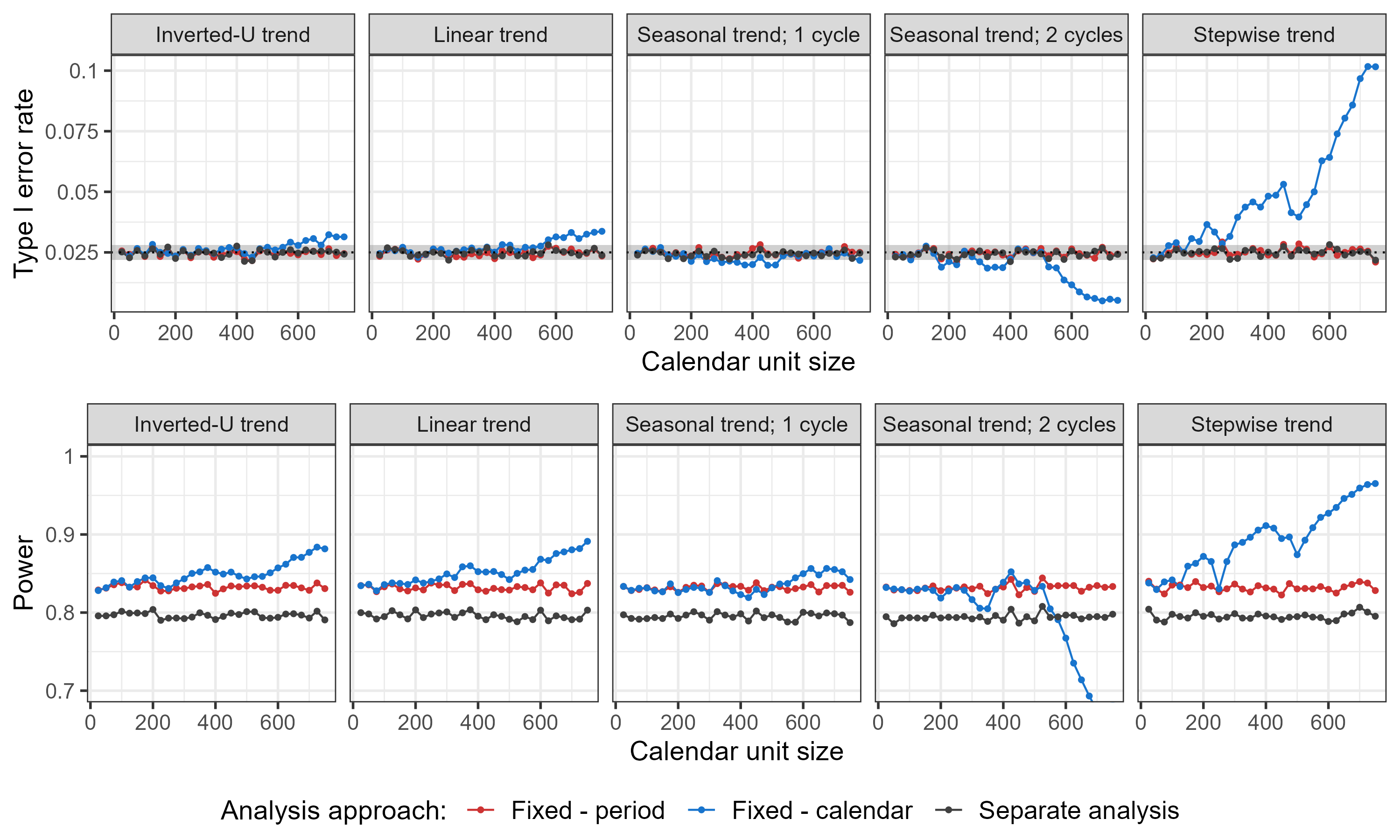

Supplement: Supplementary file 1 — Supporting Information [file BIMJ-67-e70059-s002.zip › simulations/figures/fixmodel_cal_alpha_pow_unit.png]

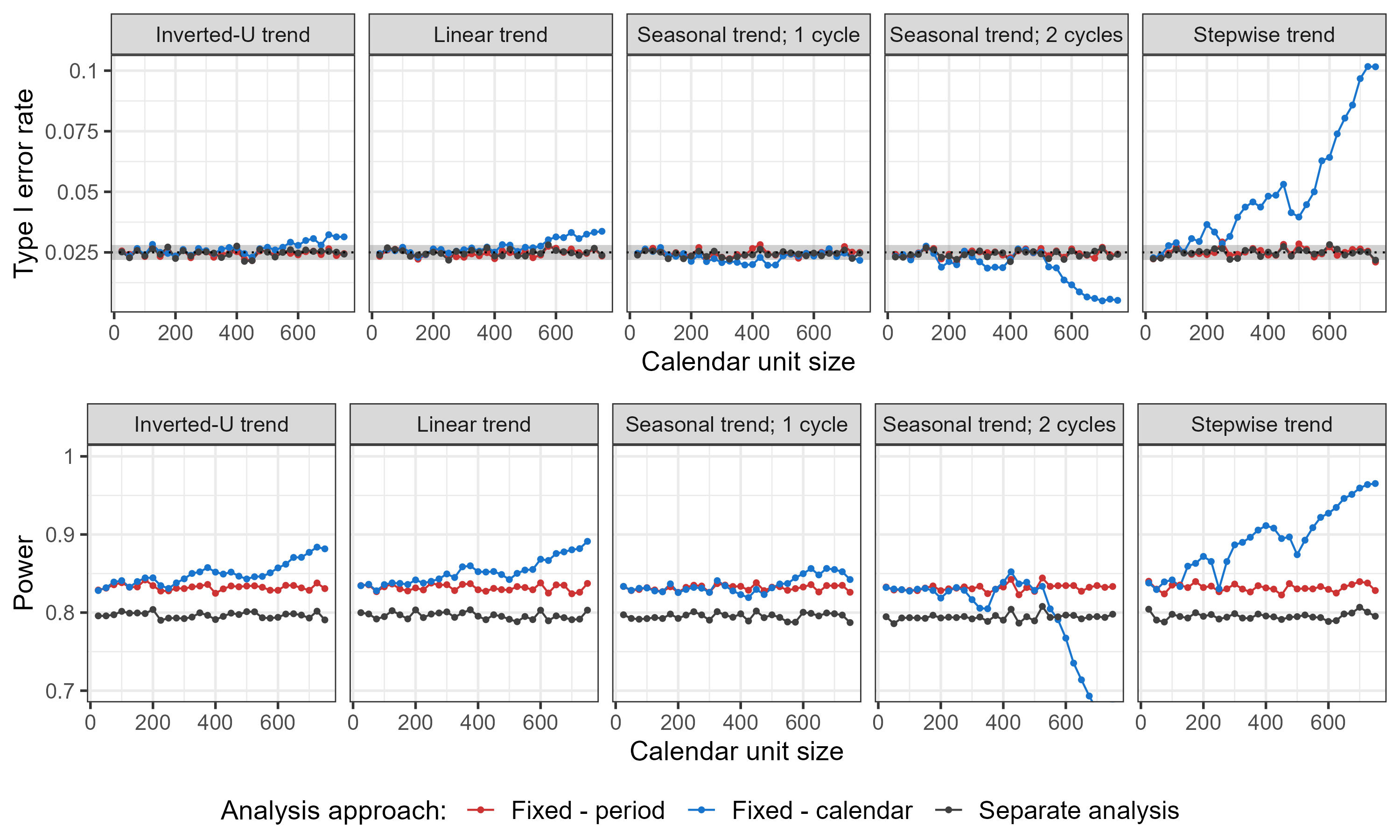

Supplement: Supplementary file 1 — Supporting Information [file BIMJ-67-e70059-s002.zip › simulations/figures/fixmodel_cal_alpha_pow_unit.tiff]

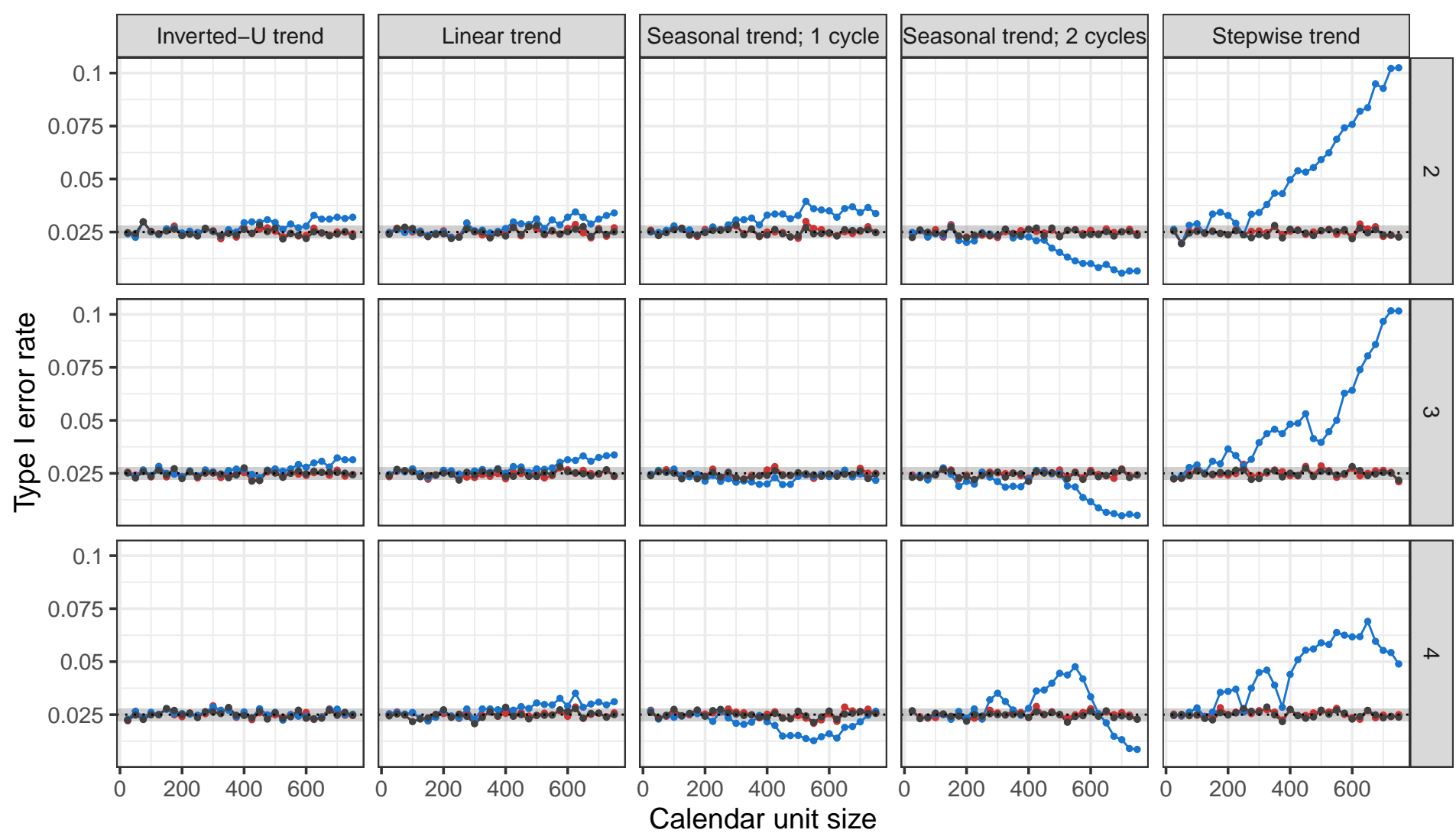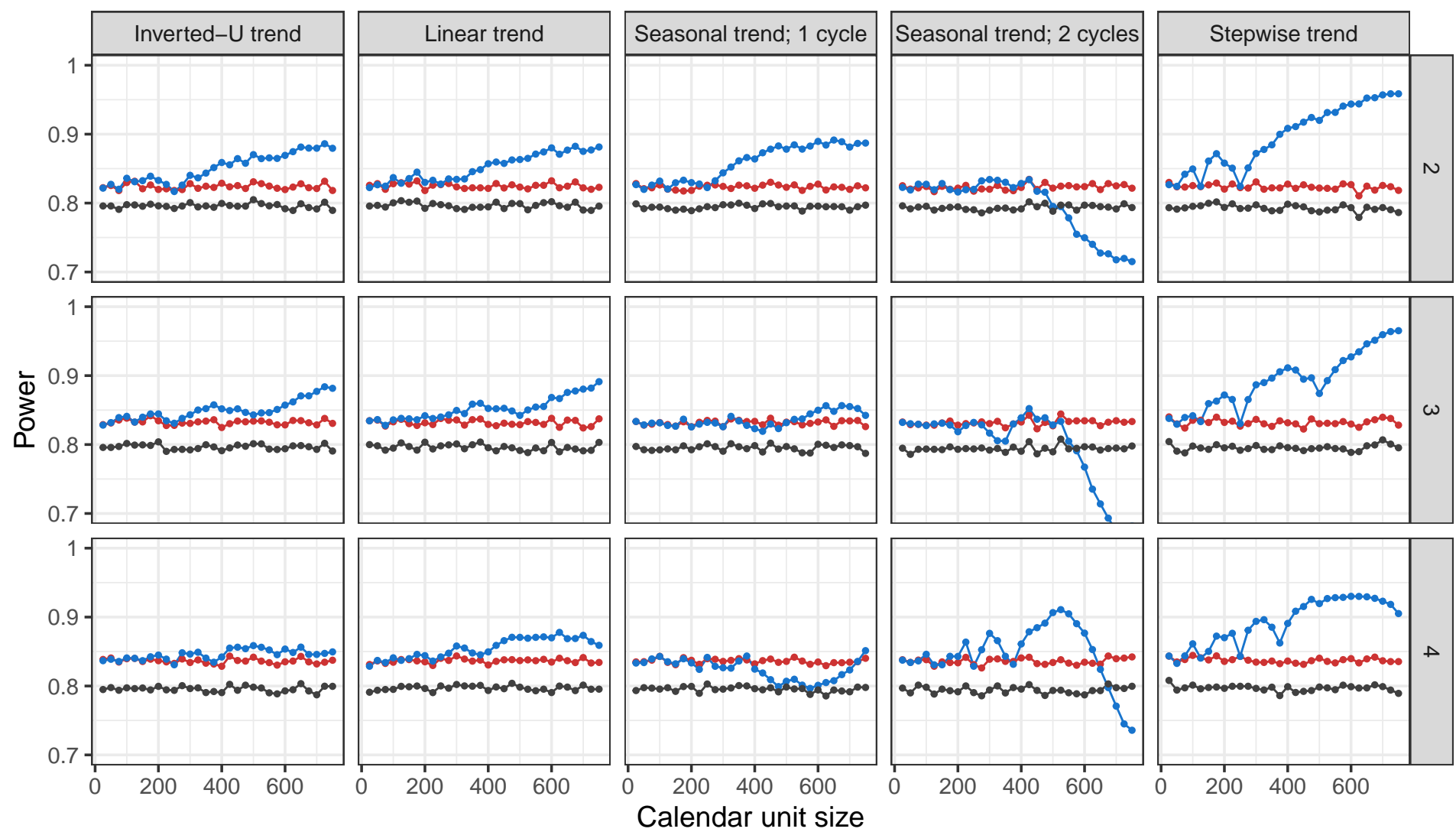

Analysis approach: —●— Fixed – period —●— Fixed – calendar —●— Separate analysis

Supplement: Supplementary file 1 — Supporting Information [file BIMJ-67-e70059-s002.zip › simulations/figures/fixmodel_cal_alpha_pow_unit_all_arms.pdf]

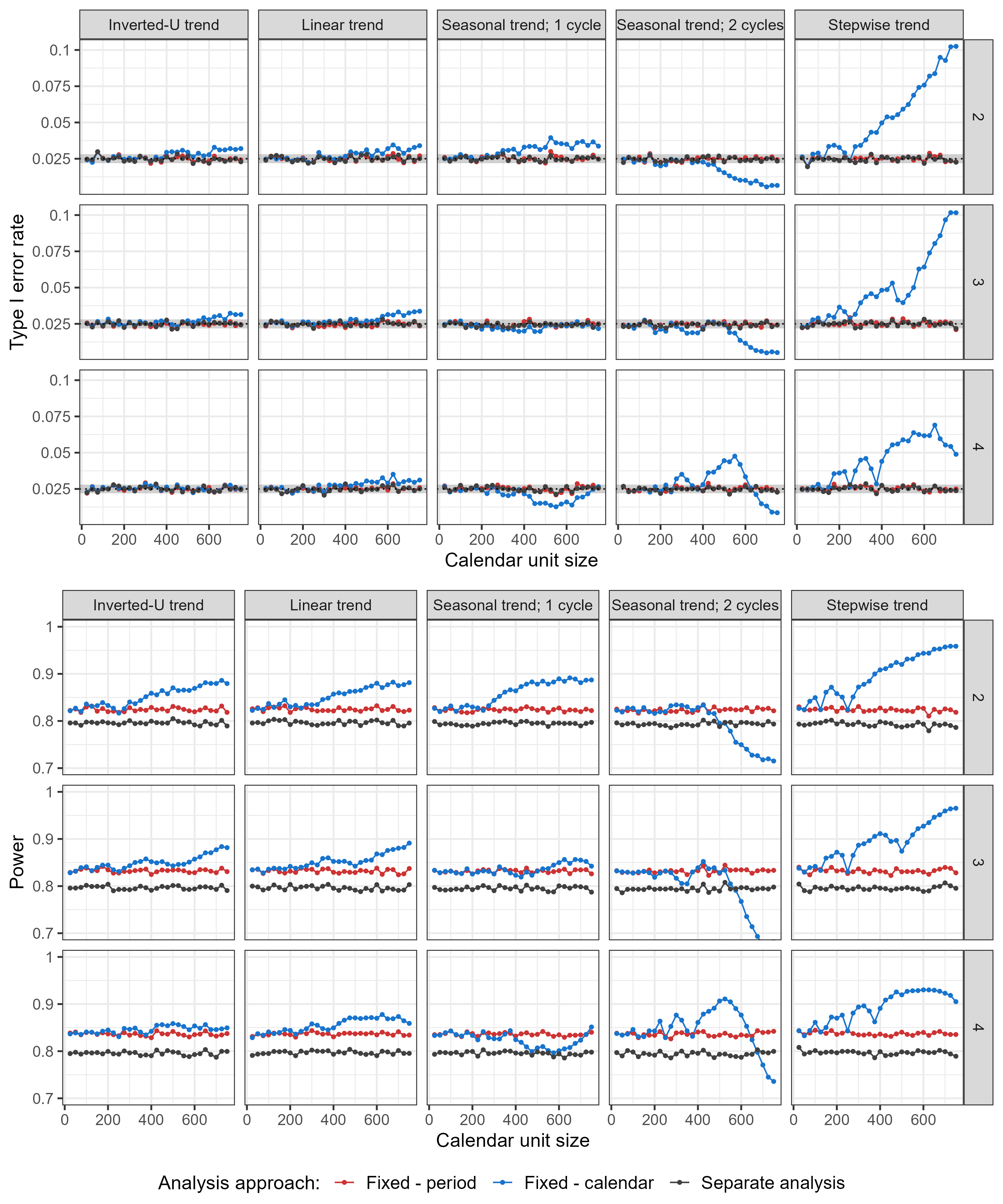

Supplement: Supplementary file 1 — Supporting Information [file BIMJ-67-e70059-s002.zip › simulations/figures/fixmodel_cal_alpha_pow_unit_all_arms.png]

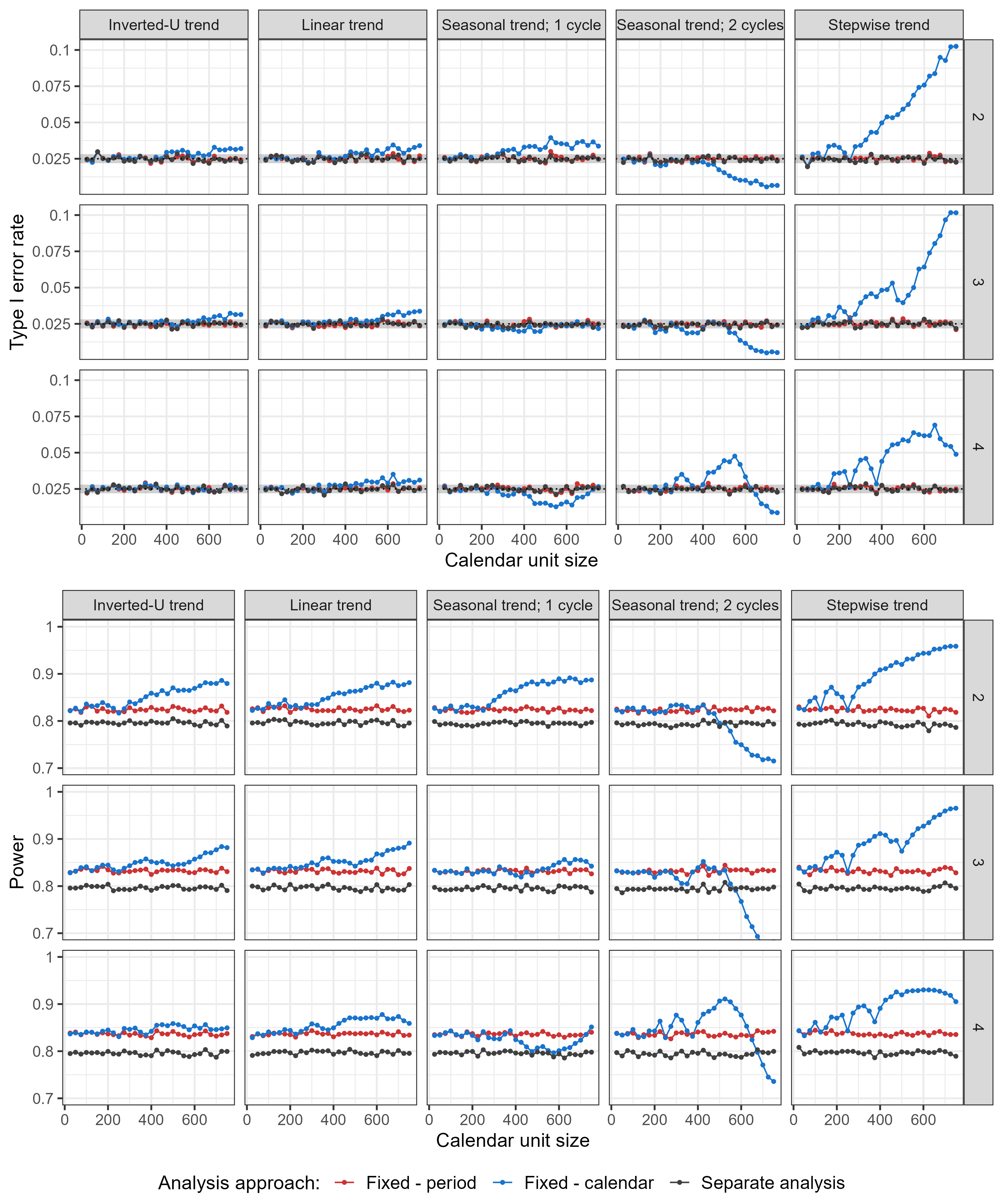

Supplement: Supplementary file 1 — Supporting Information [file BIMJ-67-e70059-s002.zip › simulations/figures/fixmodel_cal_alpha_pow_unit_all_arms.tiff]

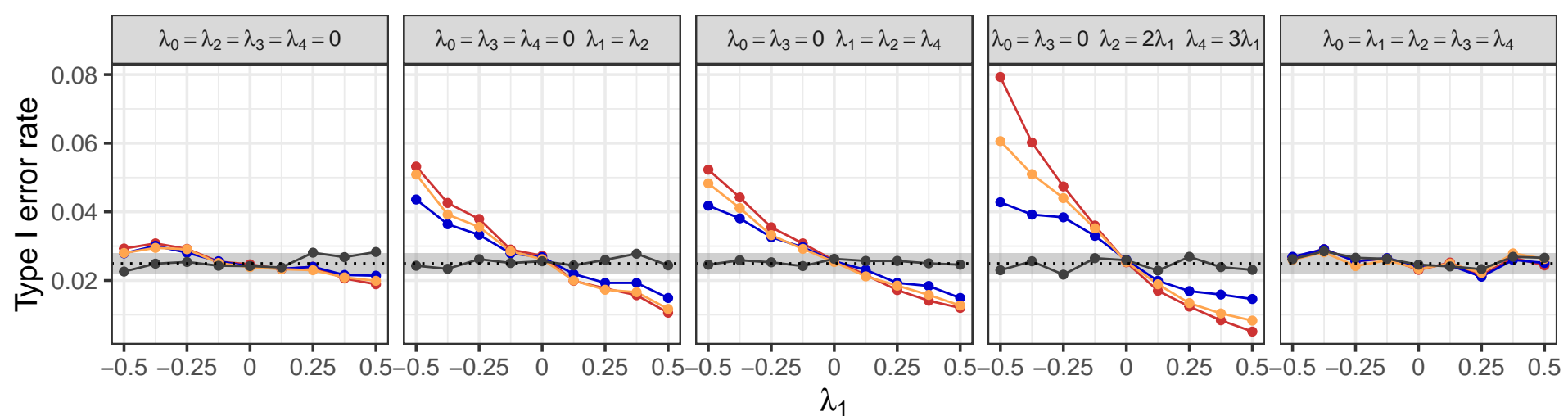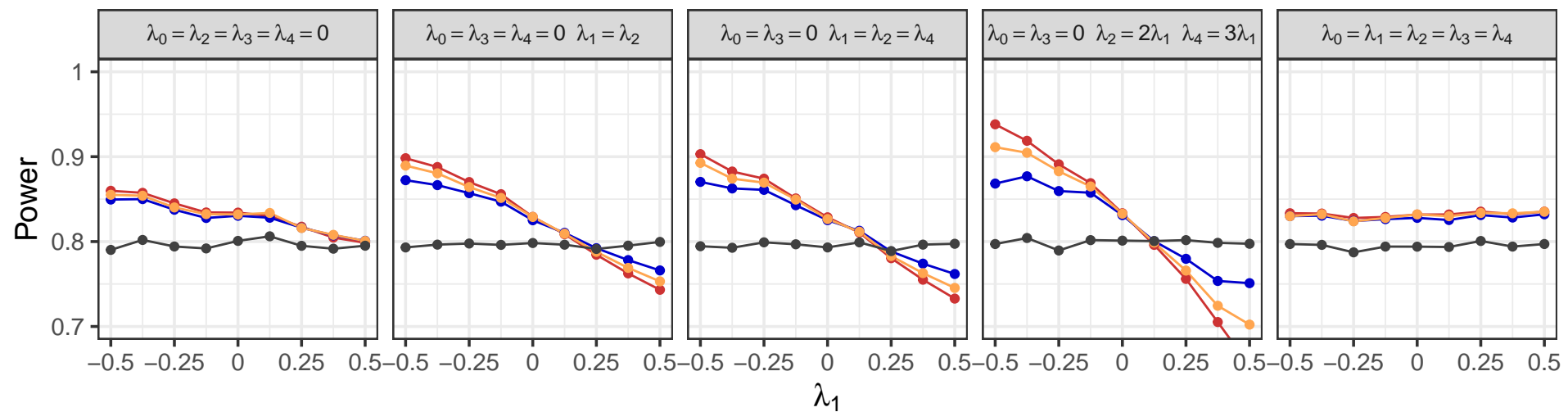

Analysis approach: Fixed – period Mixed w. inter. – period Mixed w. inter. – calendar Separate analysis

Supplement: Supplementary file 1 — Supporting Information [file BIMJ-67-e70059-s002.zip › simulations/figures/mixint_alpha_pow_lambda.pdf]

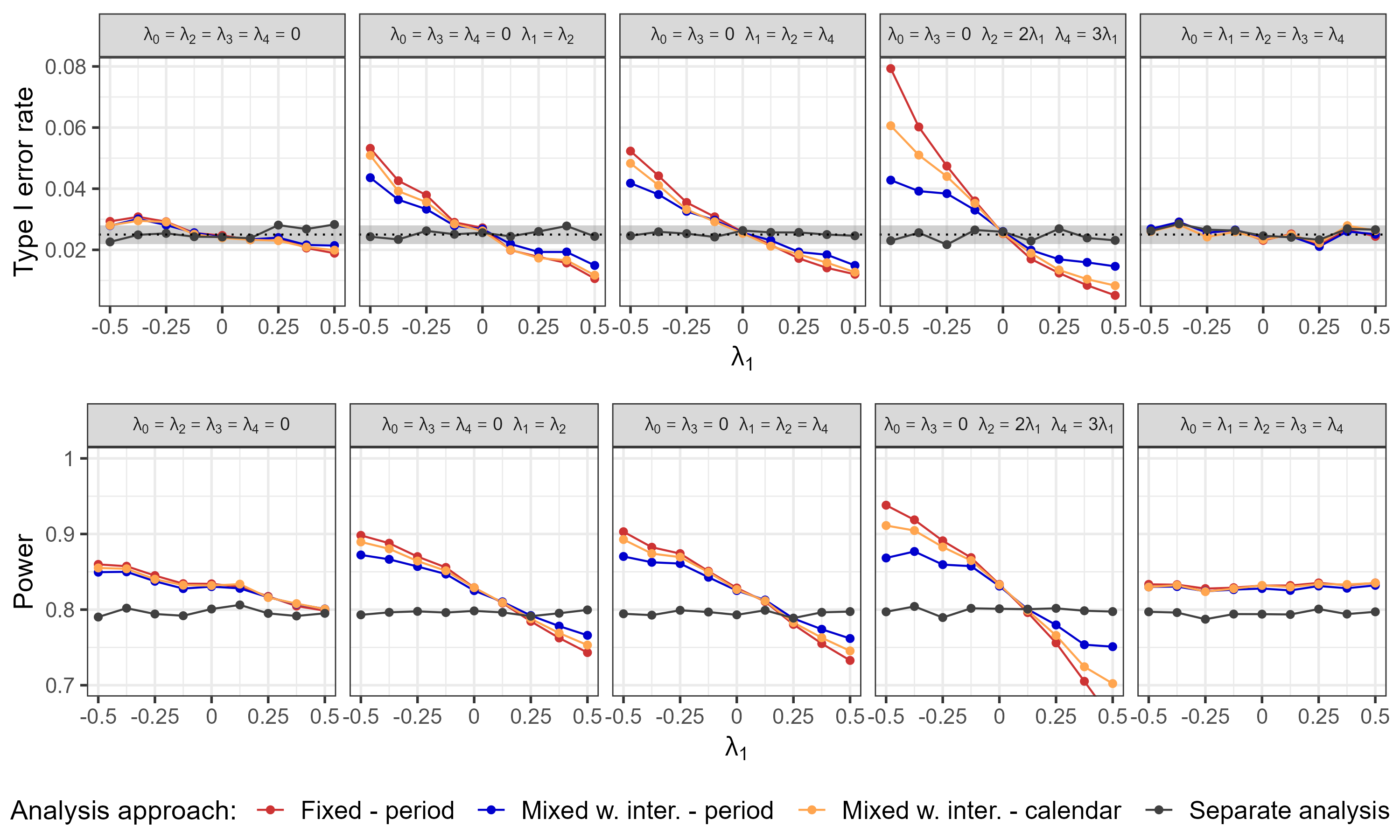

Supplement: Supplementary file 1 — Supporting Information [file BIMJ-67-e70059-s002.zip › simulations/figures/mixint_alpha_pow_lambda.png]

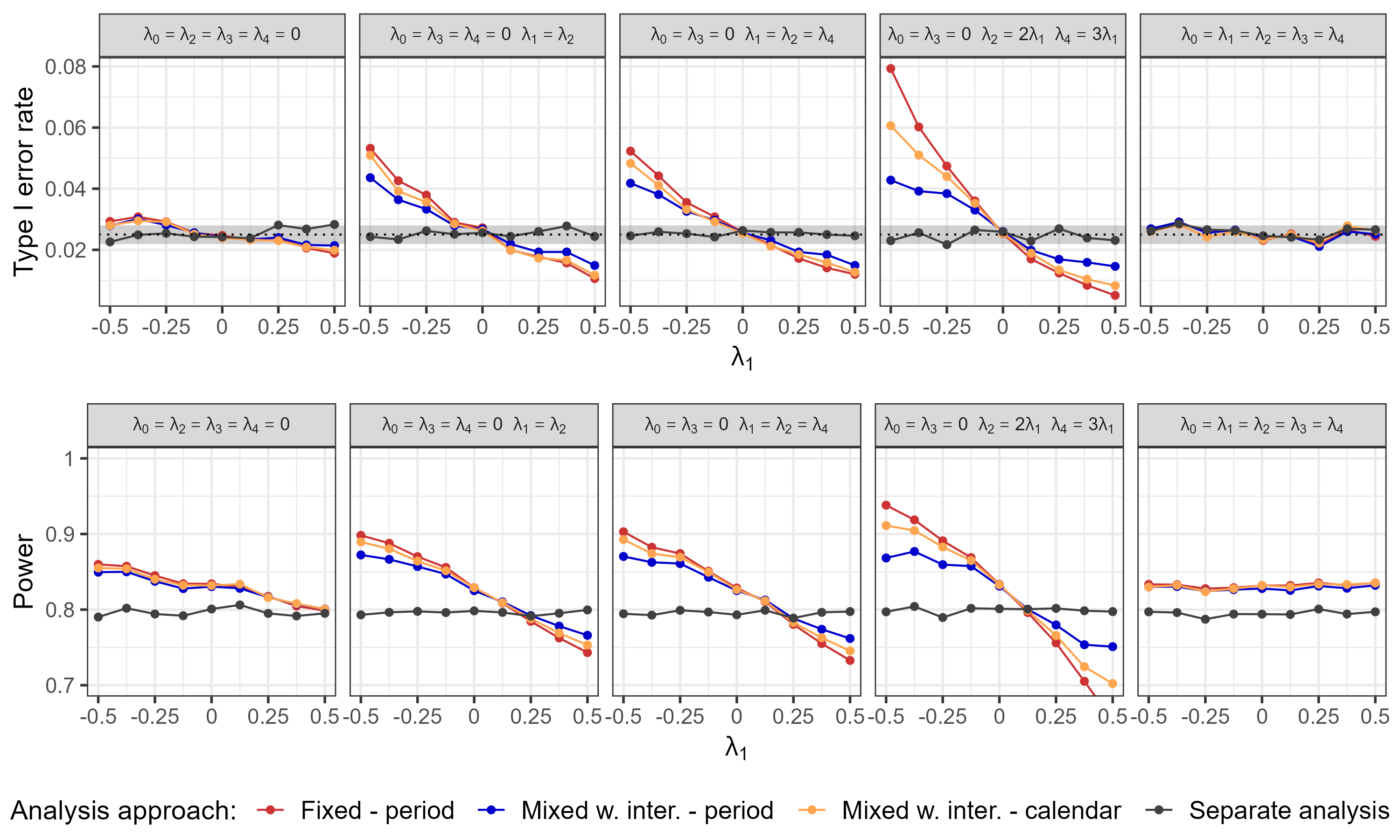

Supplement: Supplementary file 1 — Supporting Information [file BIMJ-67-e70059-s002.zip › simulations/figures/mixint_alpha_pow_lambda.tiff]

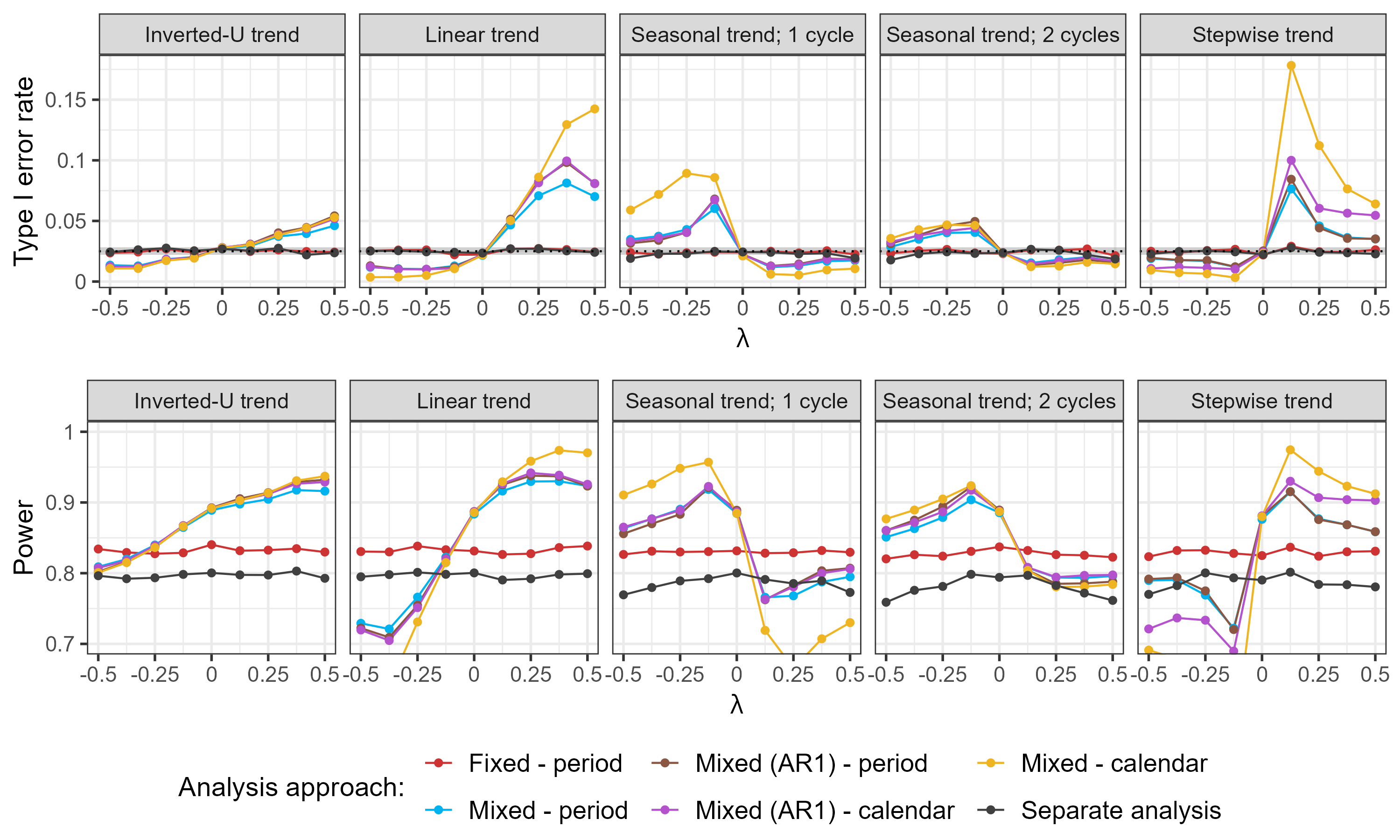

Supplement: Supplementary file 1 — Supporting Information [file BIMJ-67-e70059-s002.zip › simulations/figures/mixmodel_alpha_pow_lambda.png]

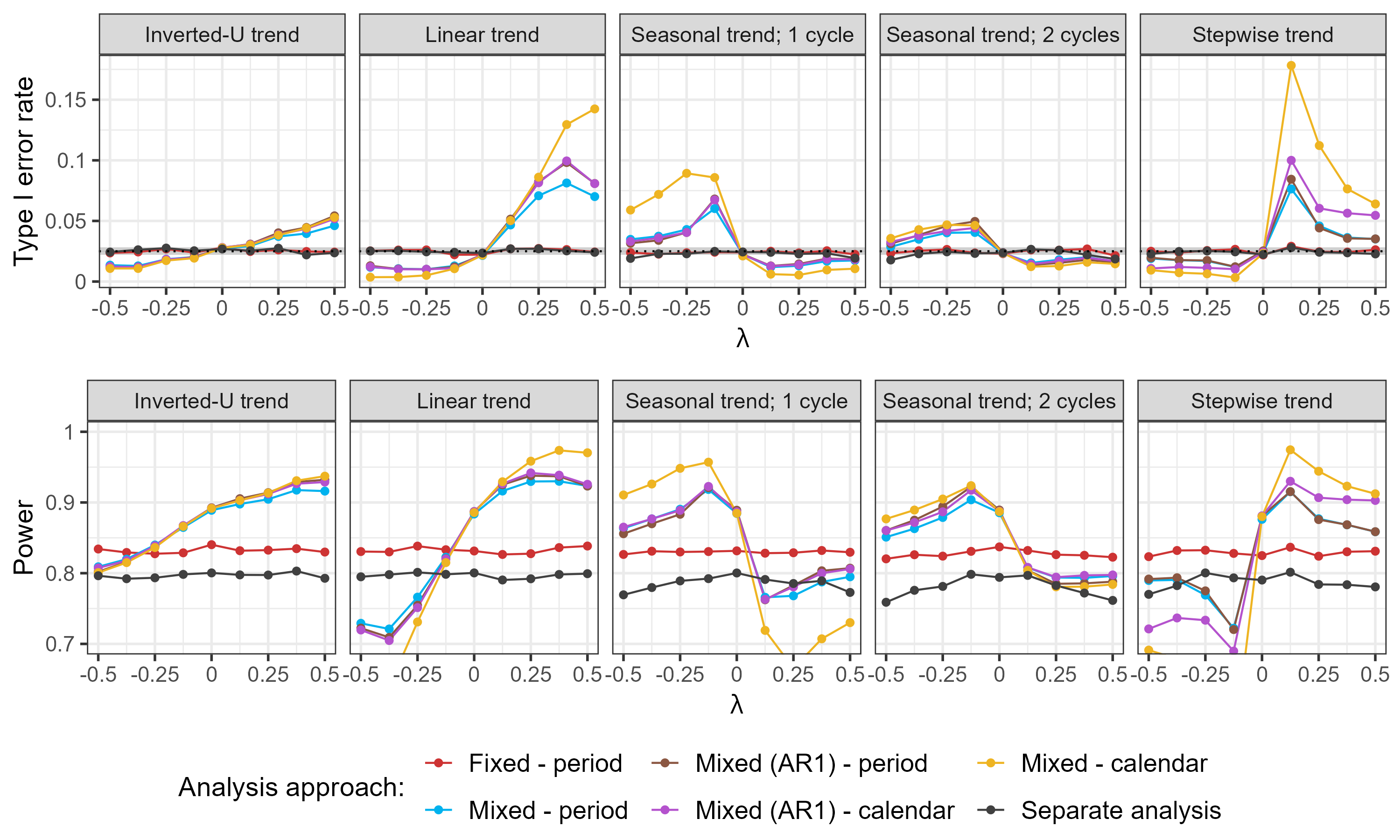

Supplement: Supplementary file 1 — Supporting Information [file BIMJ-67-e70059-s002.zip › simulations/figures/mixmodel_alpha_pow_lambda.tiff]

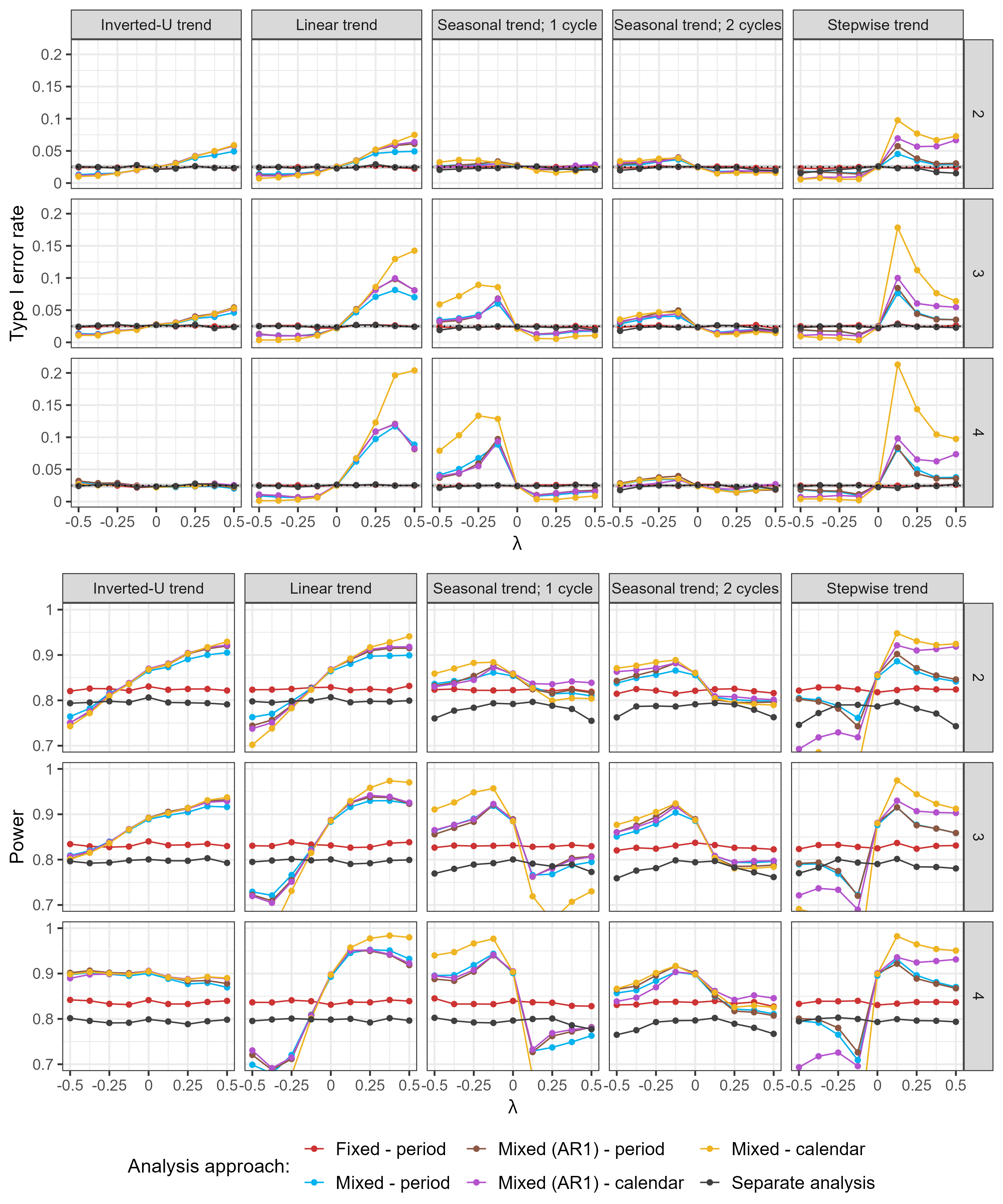

Supplement: Supplementary file 1 — Supporting Information [file BIMJ-67-e70059-s002.zip › simulations/figures/mixmodel_alpha_pow_lambda_all_arms.png]

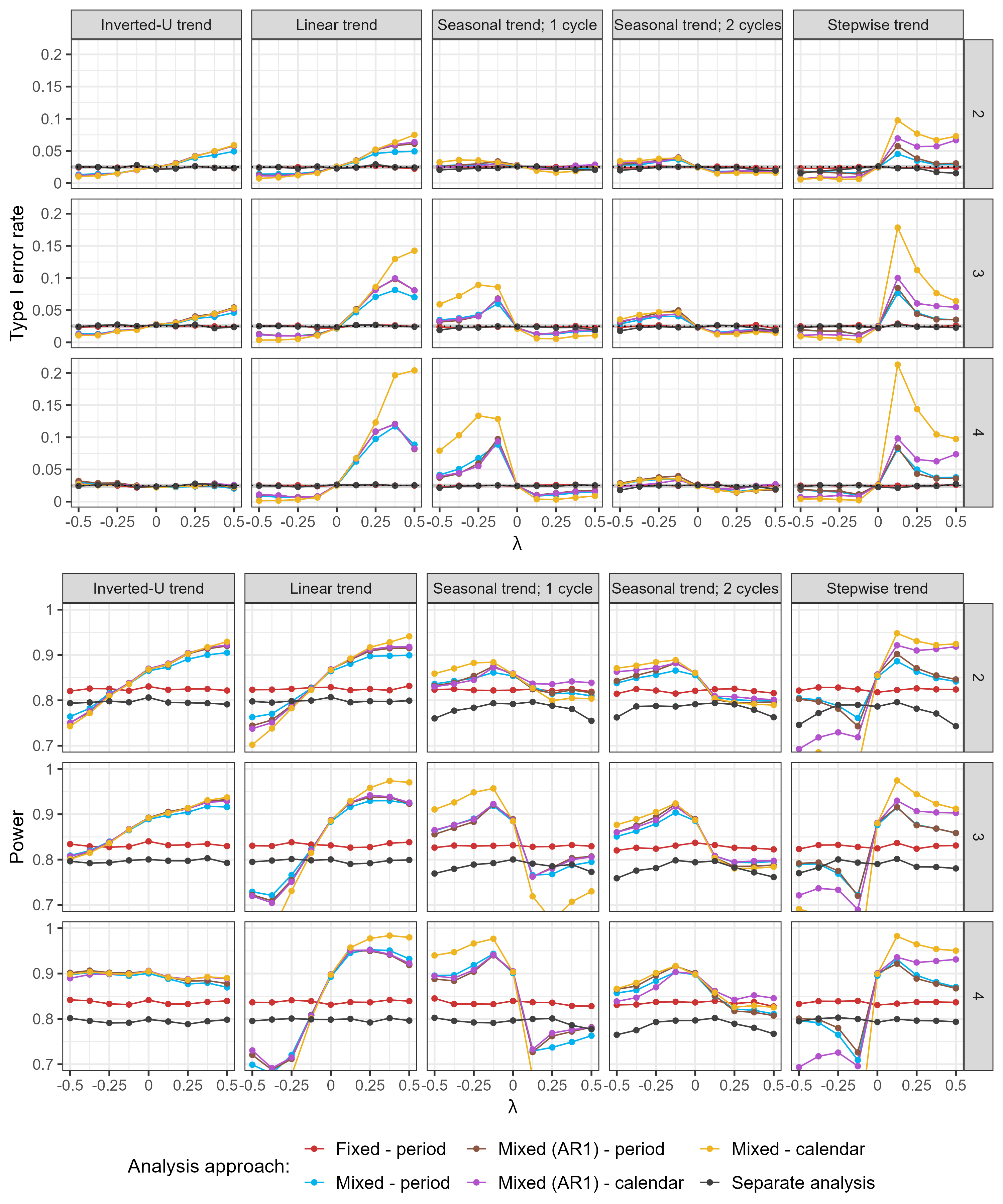

Supplement: Supplementary file 1 — Supporting Information [file BIMJ-67-e70059-s002.zip › simulations/figures/mixmodel_alpha_pow_lambda_all_arms.tiff]

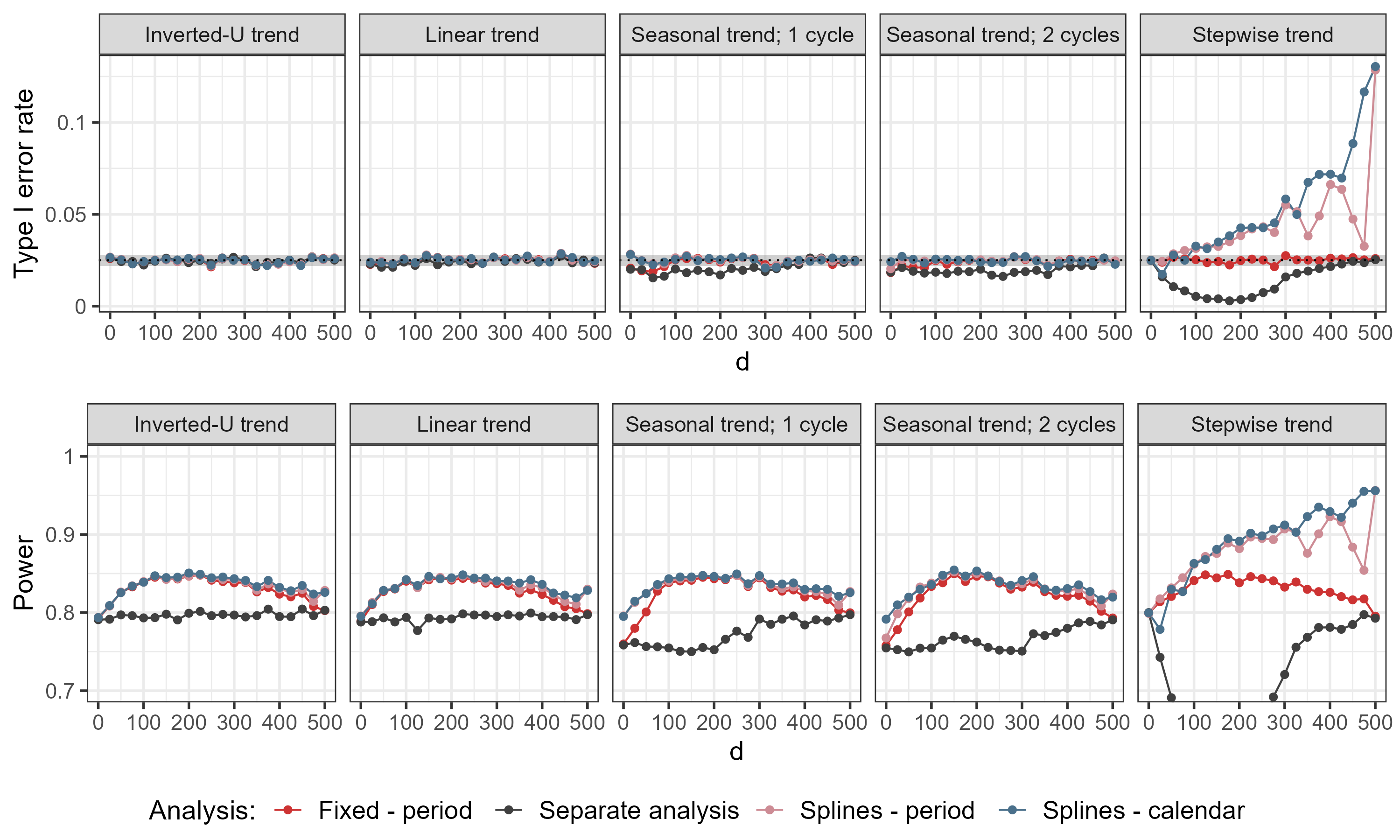

Supplement: Supplementary file 1 — Supporting Information [file BIMJ-67-e70059-s002.zip › simulations/figures/splines_alpha_pow_d_trend.png]

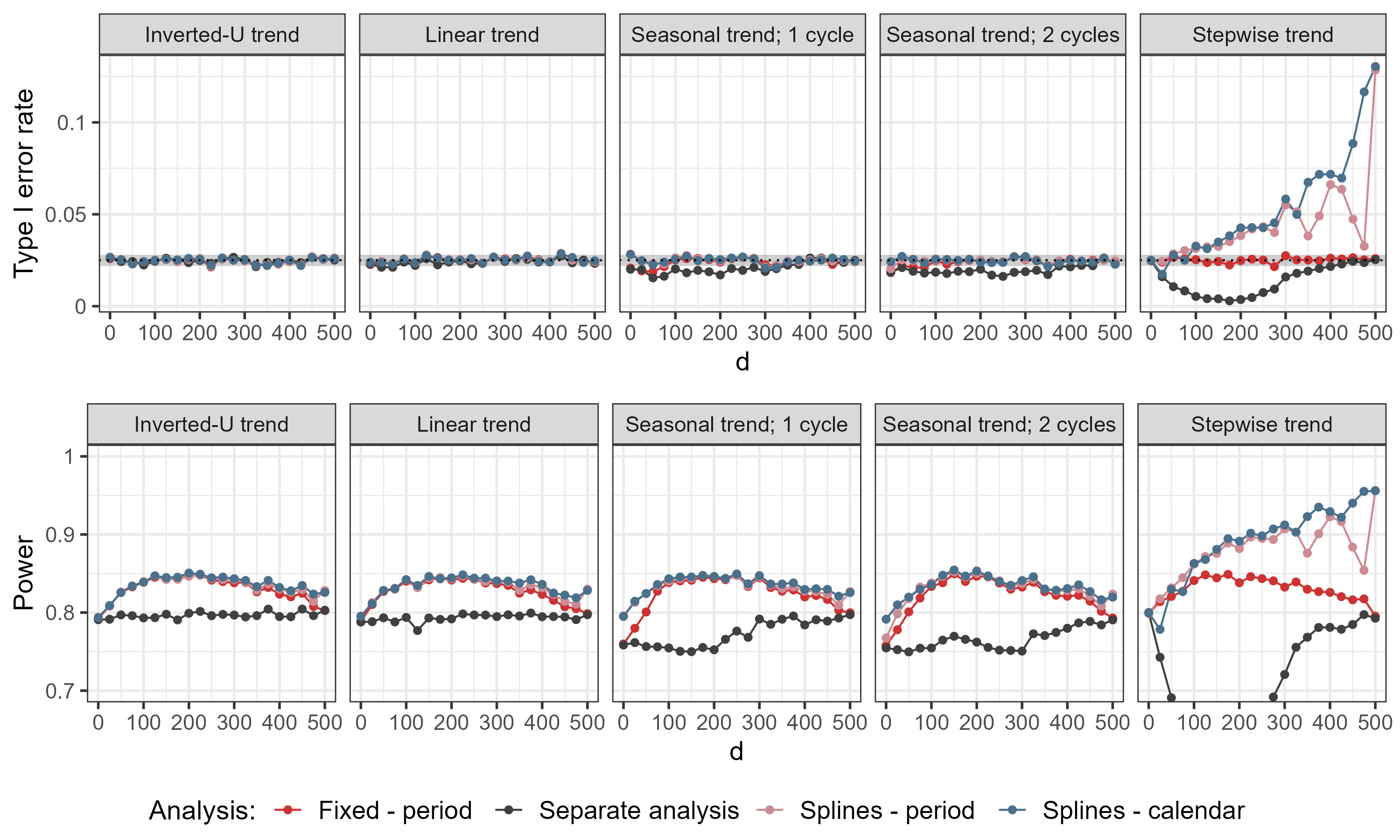

Supplement: Supplementary file 1 — Supporting Information [file BIMJ-67-e70059-s002.zip › simulations/figures/splines_alpha_pow_d_trend.tiff]

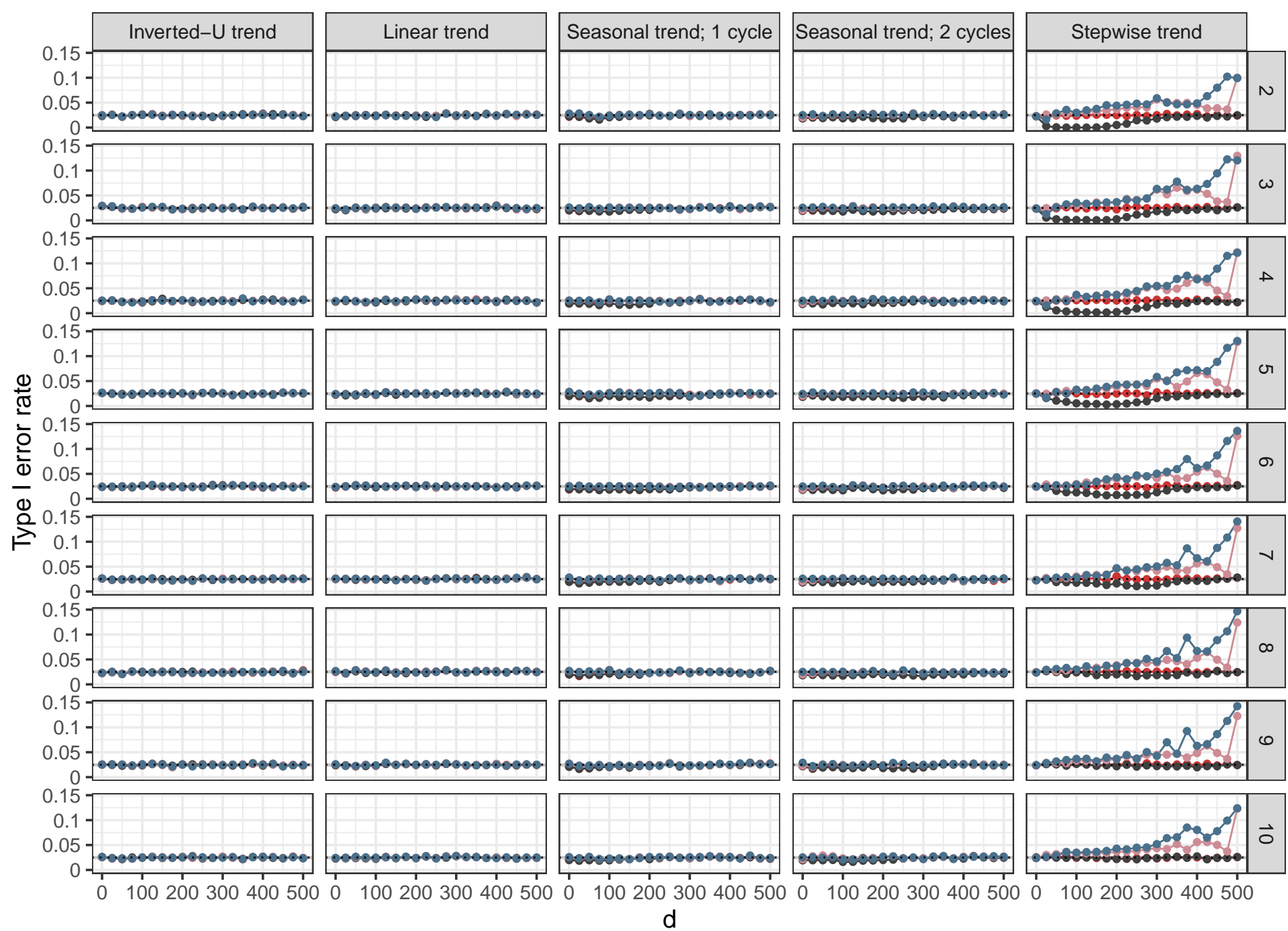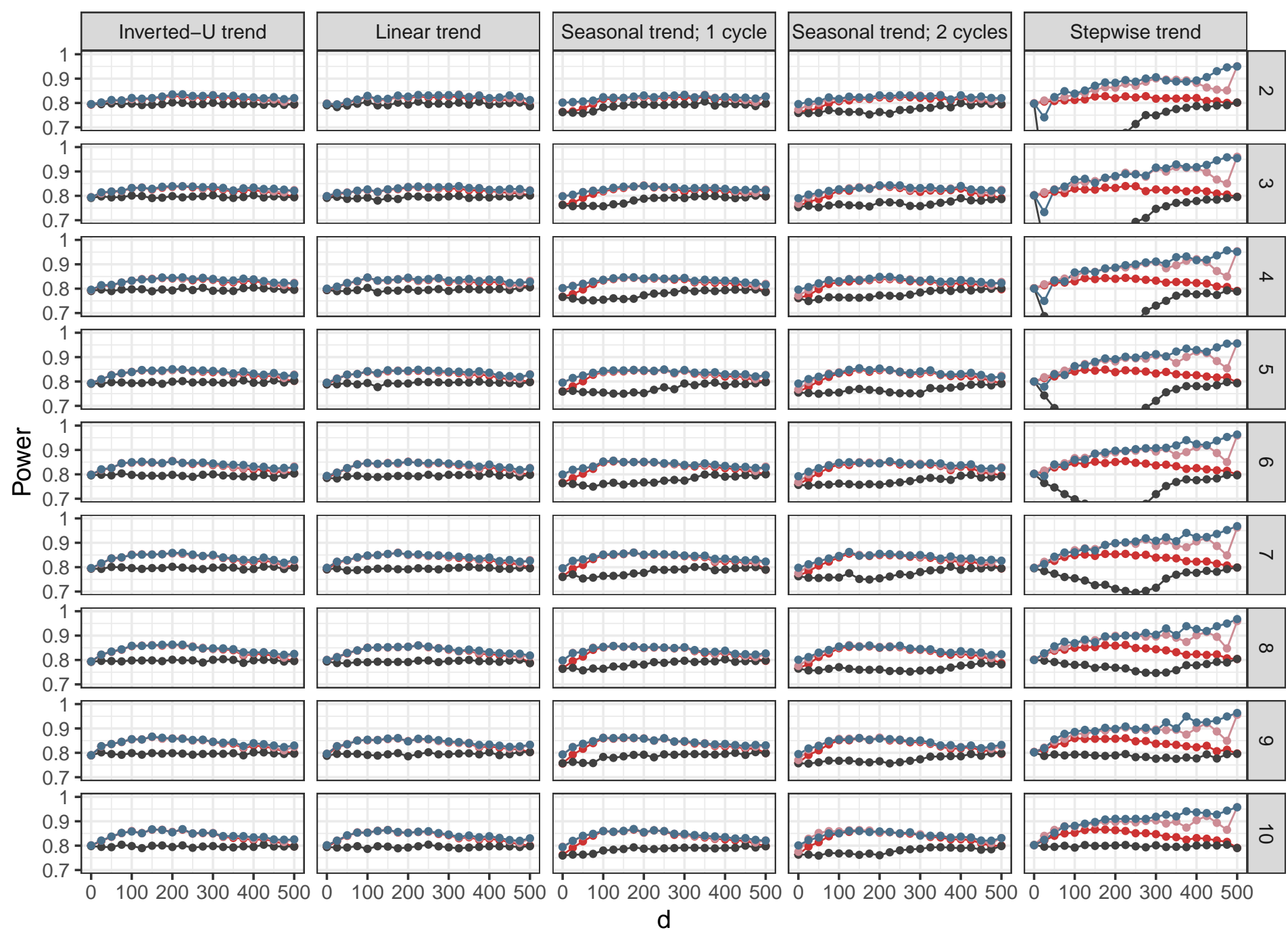

Analysis approach: Fixed – period Separate analysis Splines – period Splines – calendar

Supplement: Supplementary file 1 — Supporting Information [file BIMJ-67-e70059-s002.zip › simulations/figures/splines_alpha_pow_d_trend_all_arms.pdf]

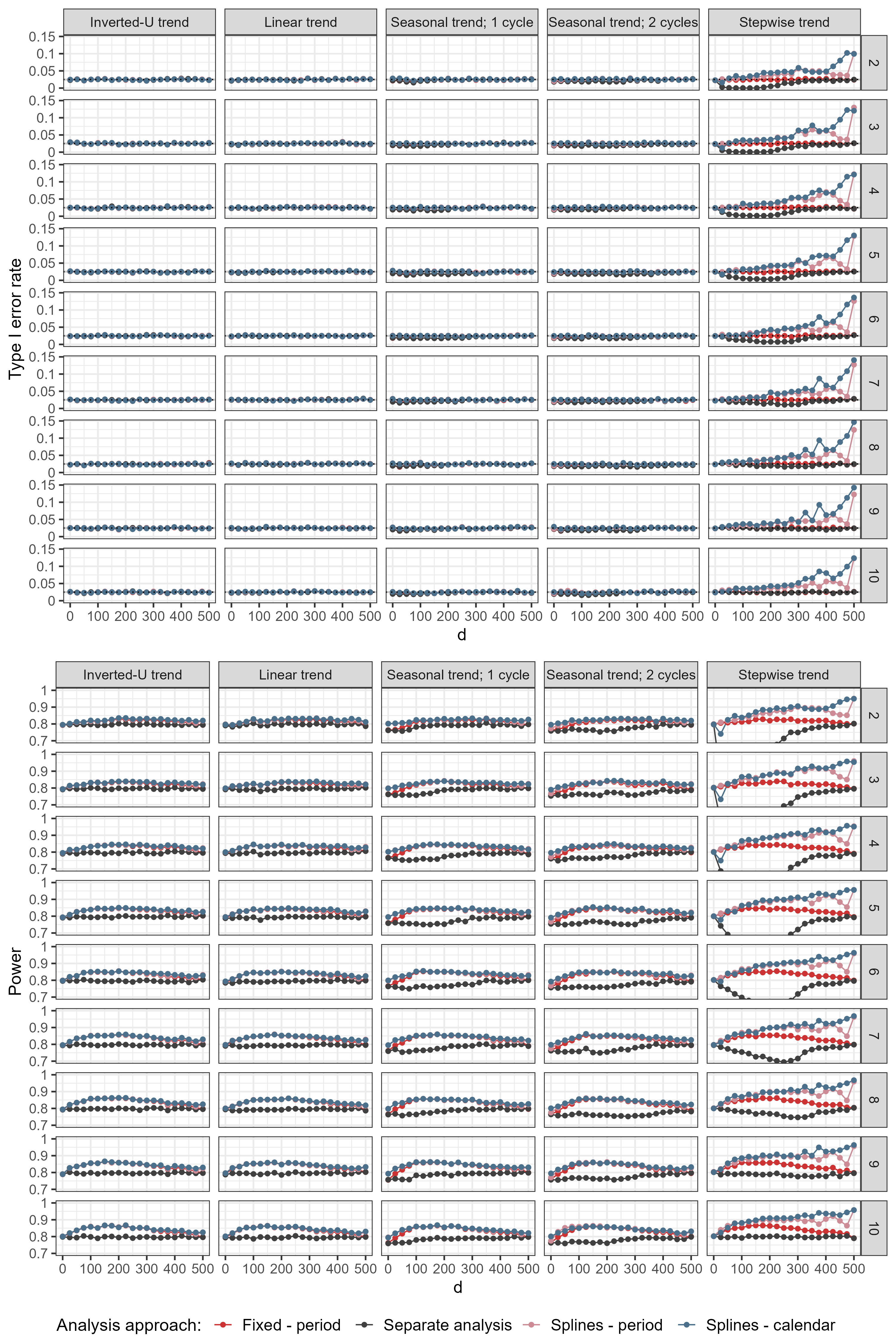

Supplement: Supplementary file 1 — Supporting Information [file BIMJ-67-e70059-s002.zip › simulations/figures/splines_alpha_pow_d_trend_all_arms.png]

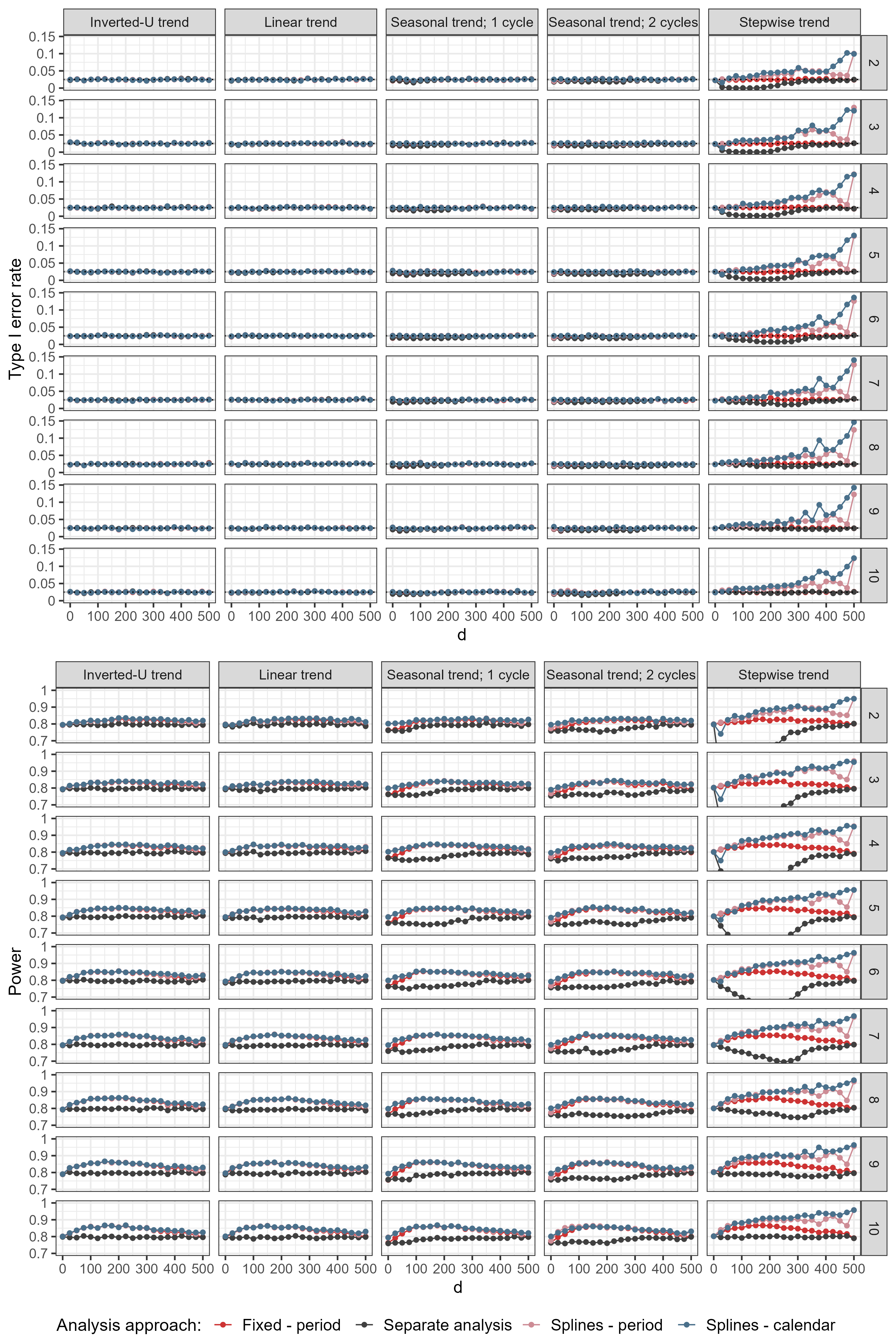

Supplement: Supplementary file 1 — Supporting Information [file BIMJ-67-e70059-s002.zip › simulations/figures/splines_alpha_pow_d_trend_all_arms.tiff]

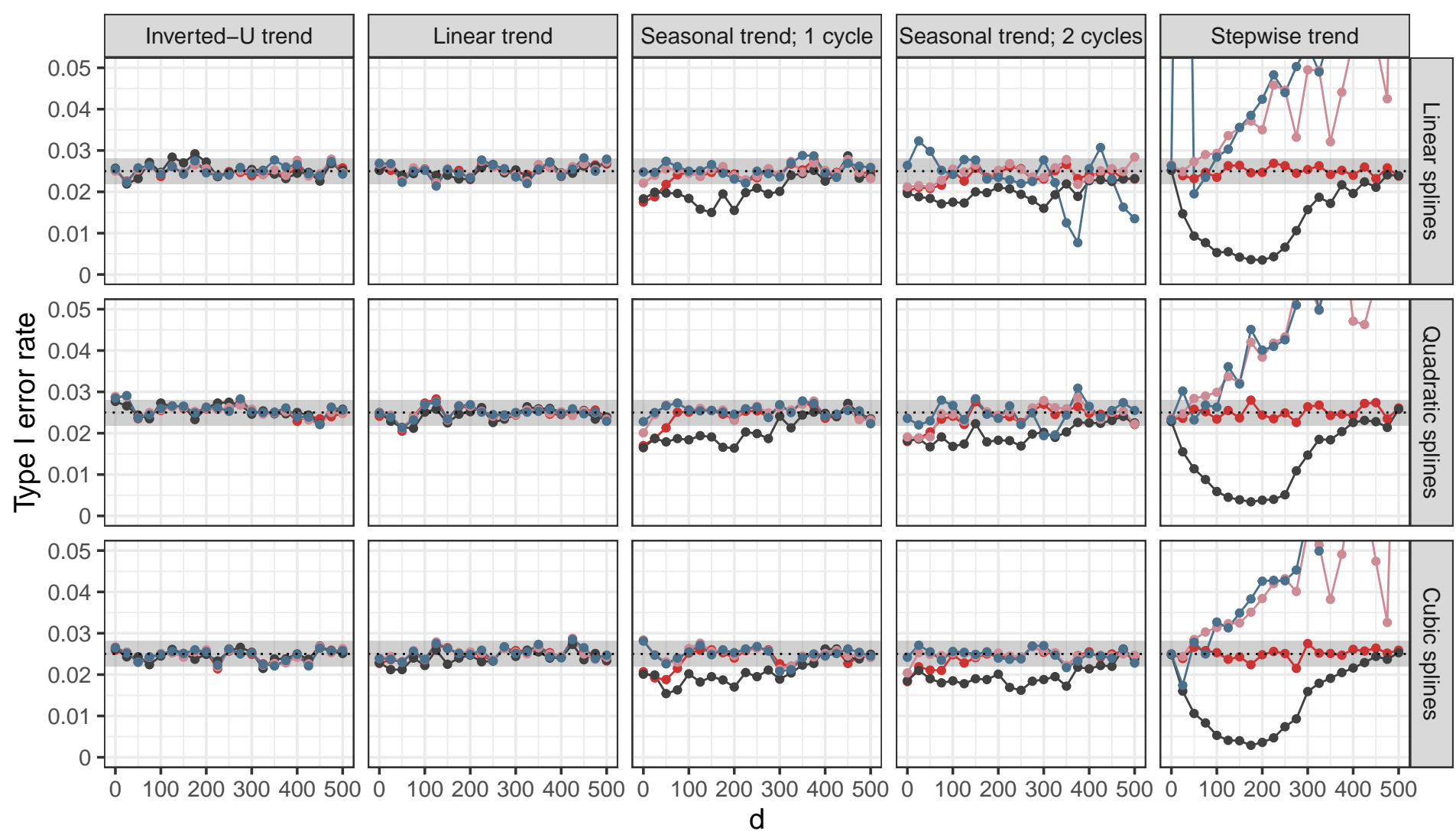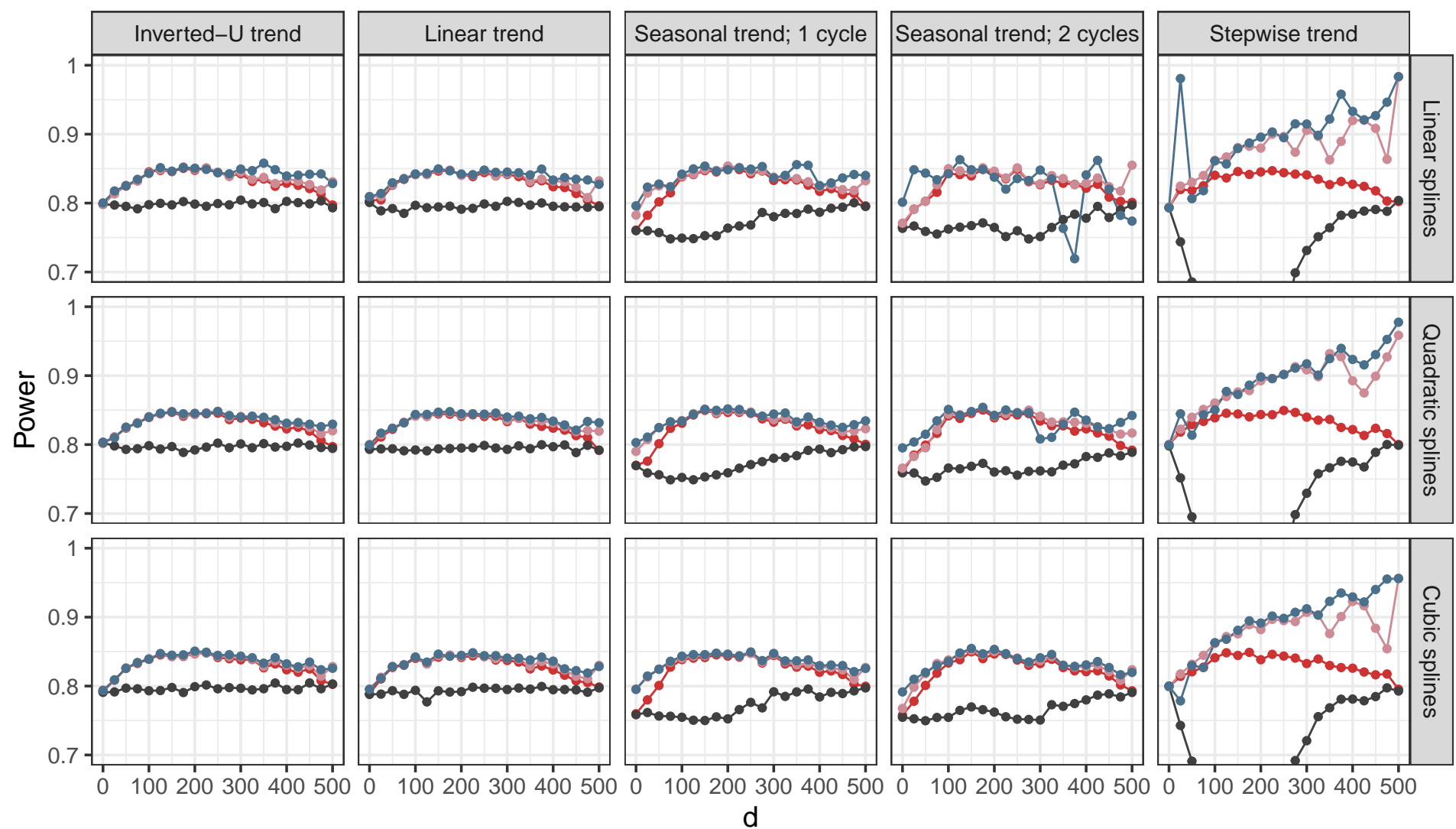

Analysis: —●— Fixed – period —●— Separate analysis —●— Splines – period —●— Splines – calendar

Supplement: Supplementary file 1 — Supporting Information [file BIMJ-67-e70059-s002.zip › simulations/figures/splines_alpha_pow_d_trend_all_degrees.pdf]

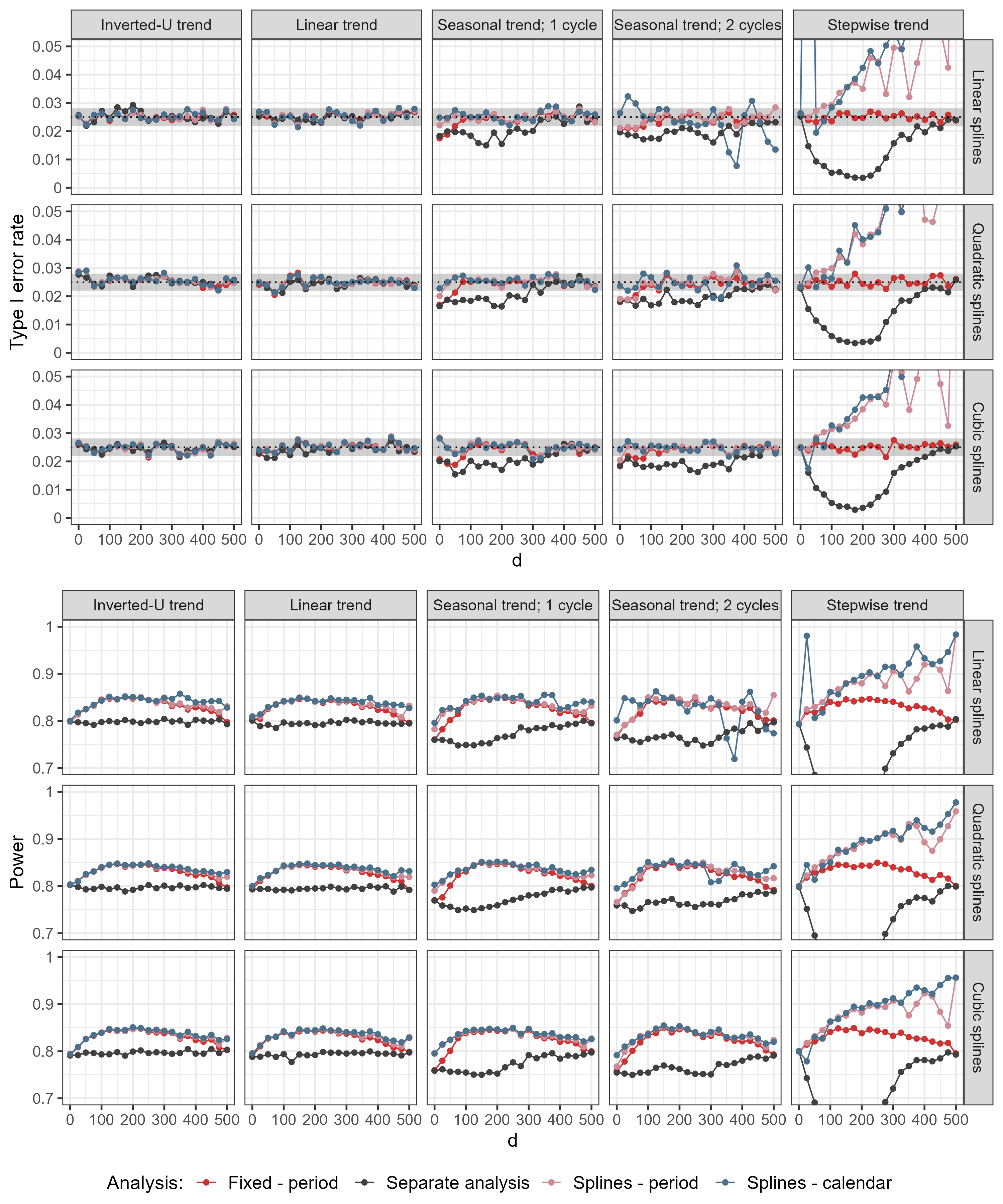

Supplement: Supplementary file 1 — Supporting Information [file BIMJ-67-e70059-s002.zip › simulations/figures/splines_alpha_pow_d_trend_all_degrees.png]

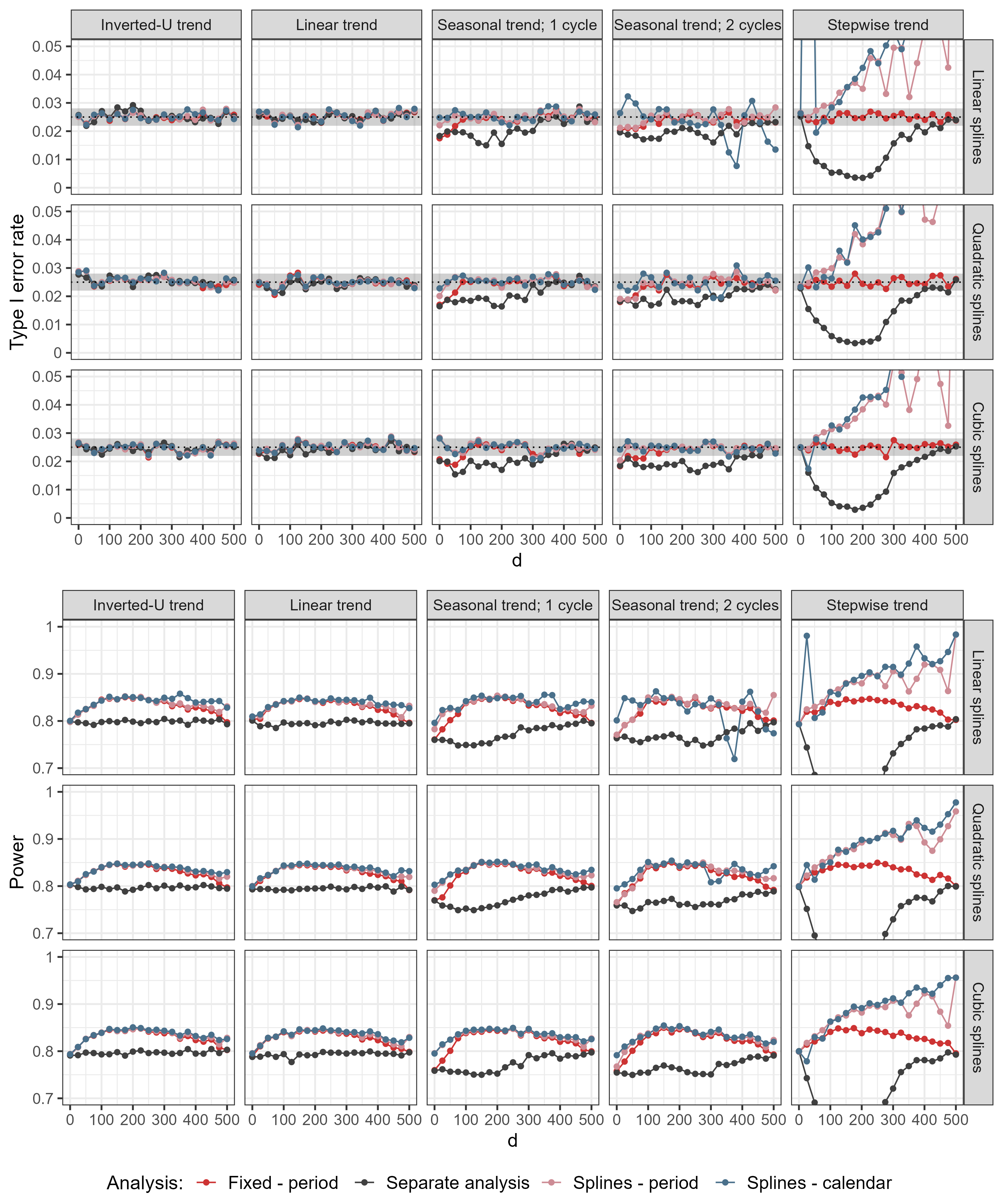

Supplement: Supplementary file 1 — Supporting Information [file BIMJ-67-e70059-s002.zip › simulations/figures/splines_alpha_pow_d_trend_all_degrees.tiff]

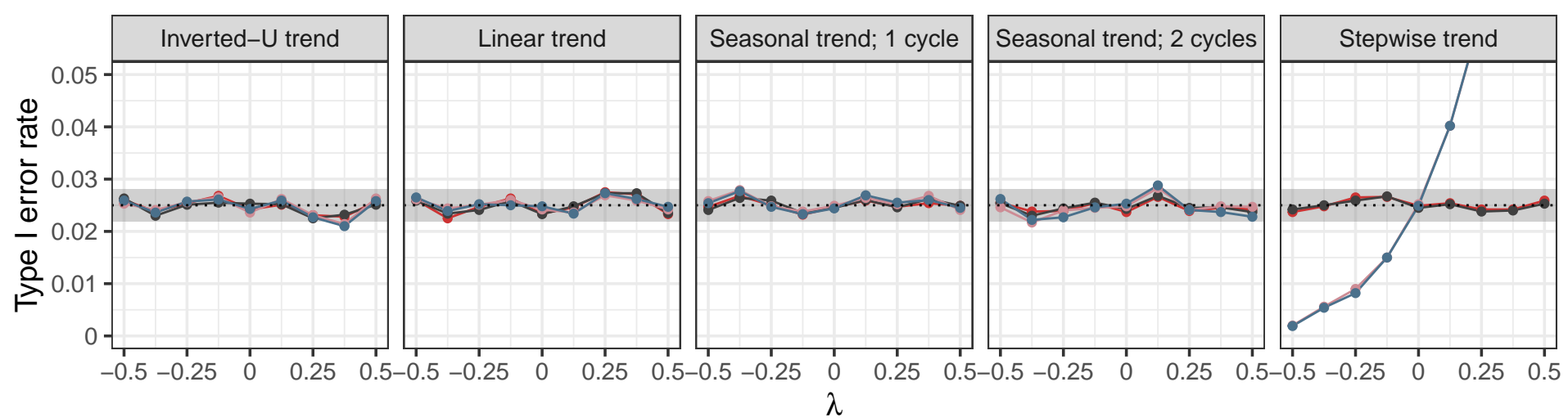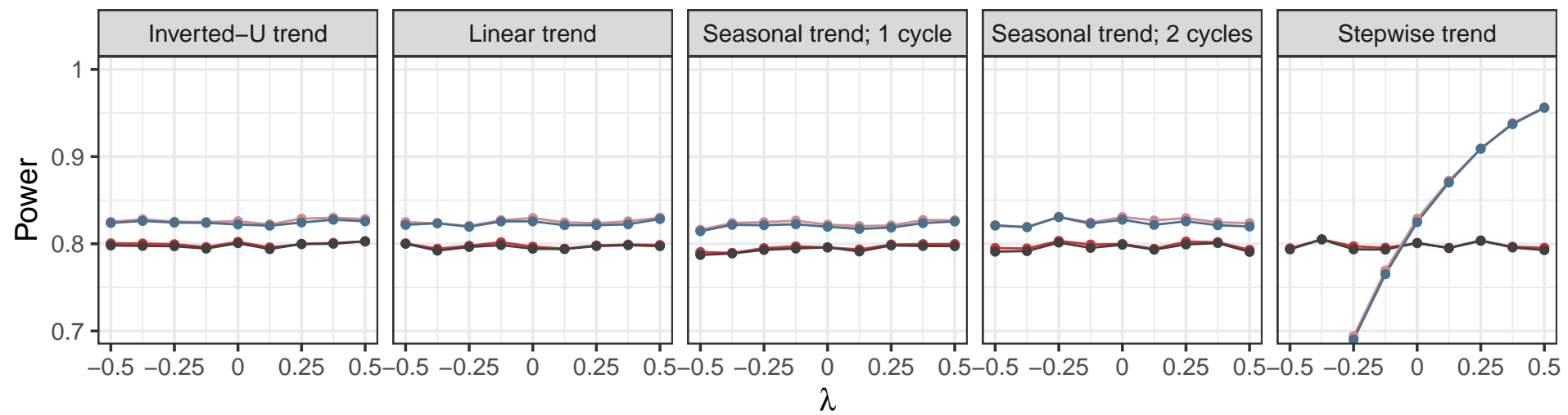

Analysis: —●— Fixed – period —●— Separate analysis —●— Splines – period —●— Splines – calendar

Supplement: Supplementary file 1 — Supporting Information [file BIMJ-67-e70059-s002.zip › simulations/figures/splines_alpha_pow_lambda_trend.pdf]

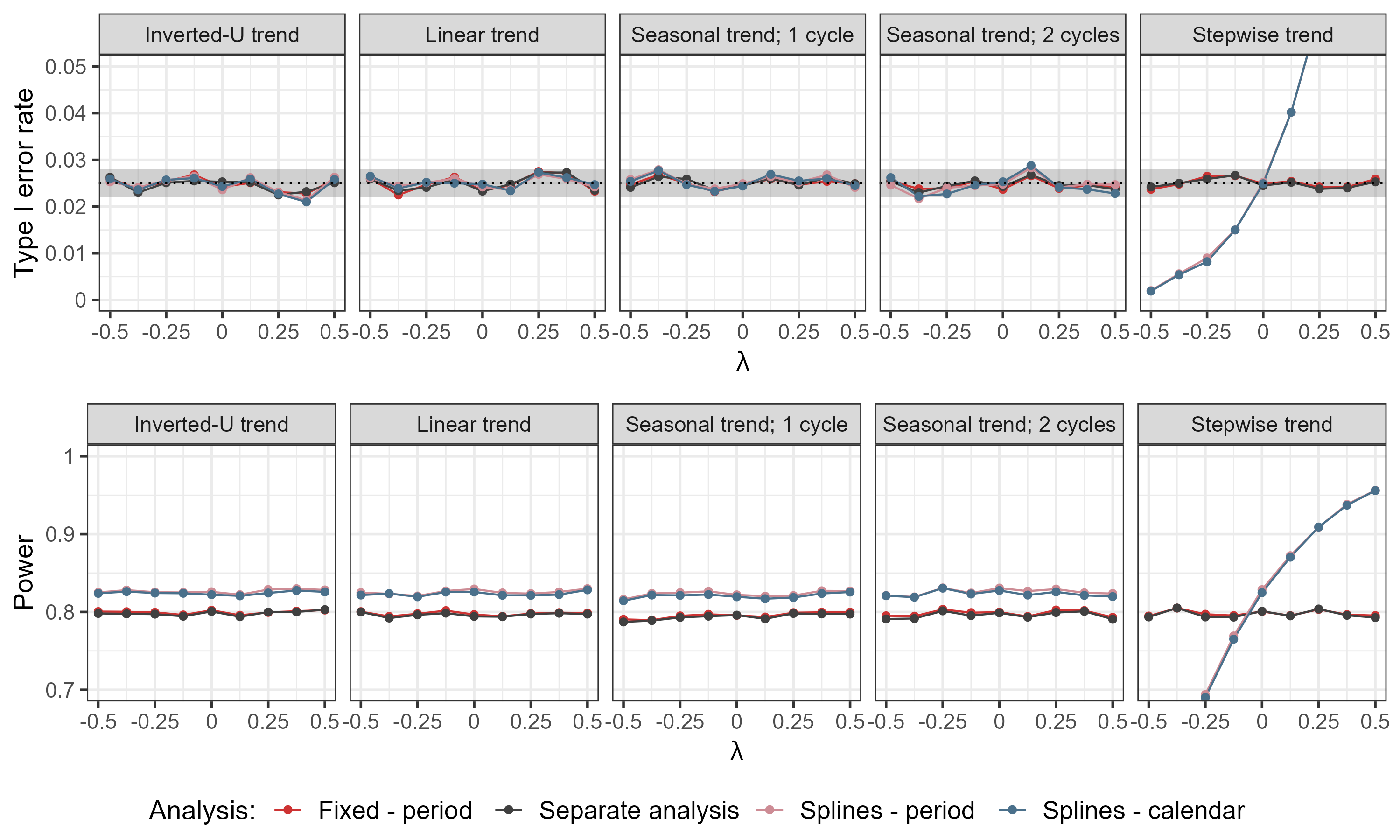

Supplement: Supplementary file 1 — Supporting Information [file BIMJ-67-e70059-s002.zip › simulations/figures/splines_alpha_pow_lambda_trend.png]

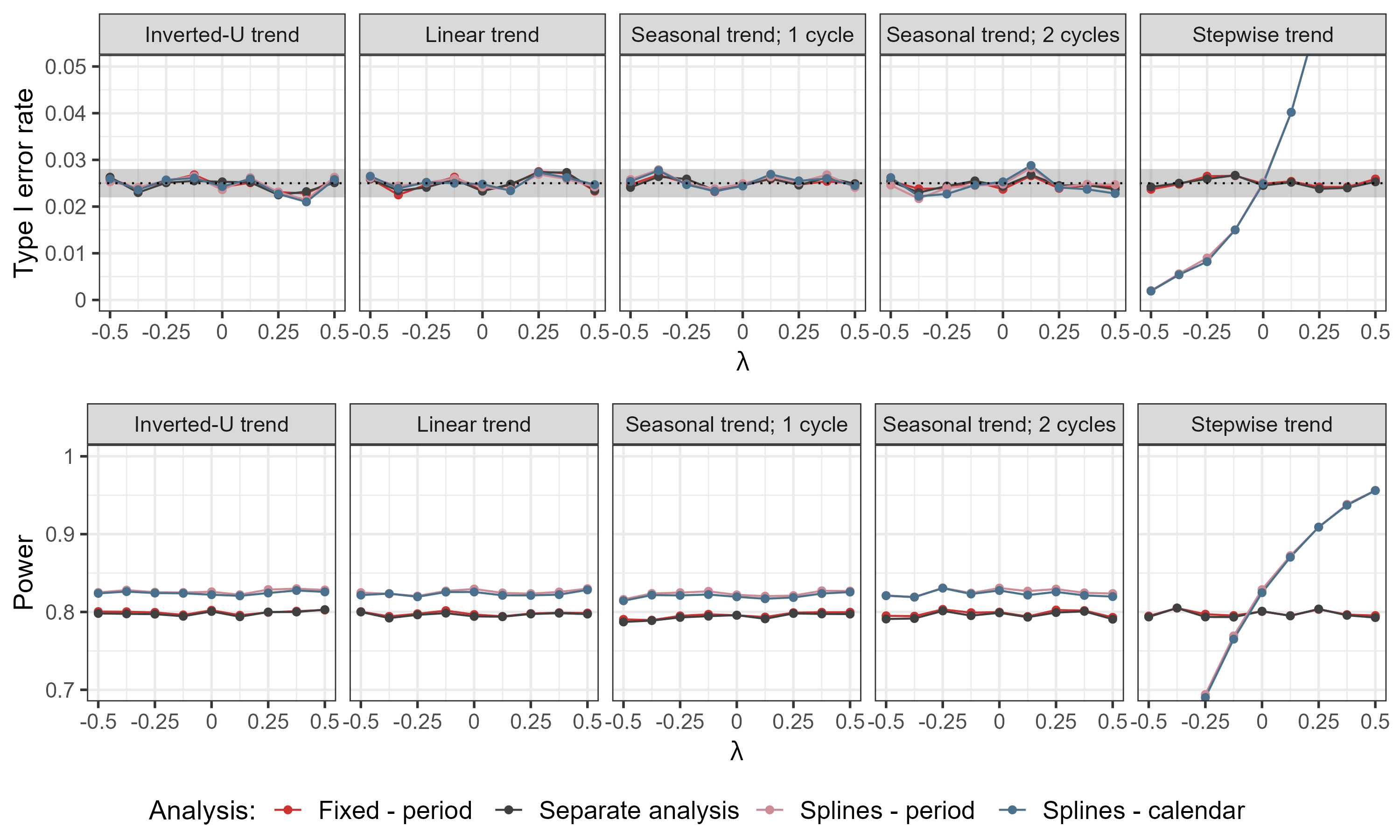

Supplement: Supplementary file 1 — Supporting Information [file BIMJ-67-e70059-s002.zip › simulations/figures/splines_alpha_pow_lambda_trend.tiff]

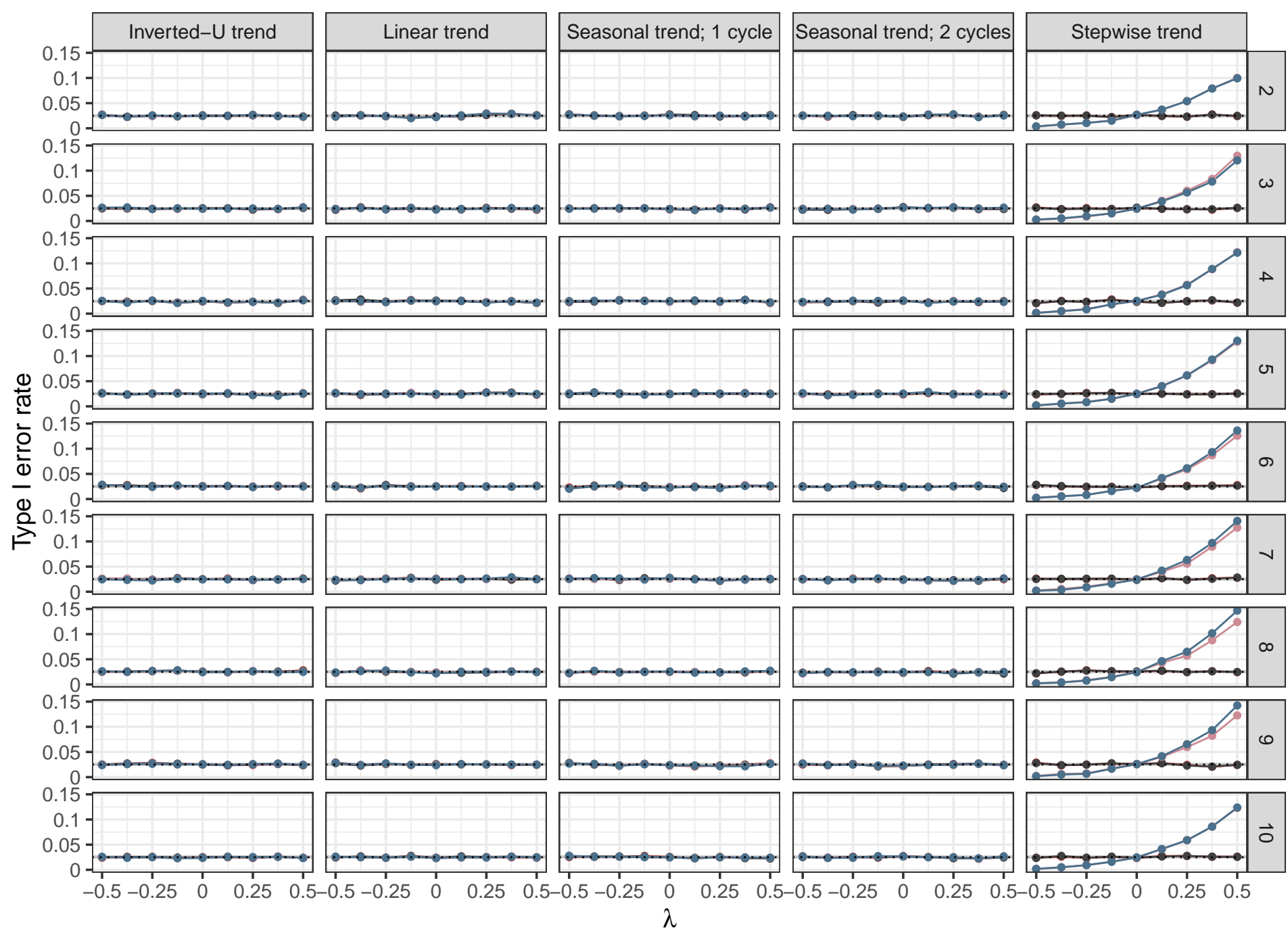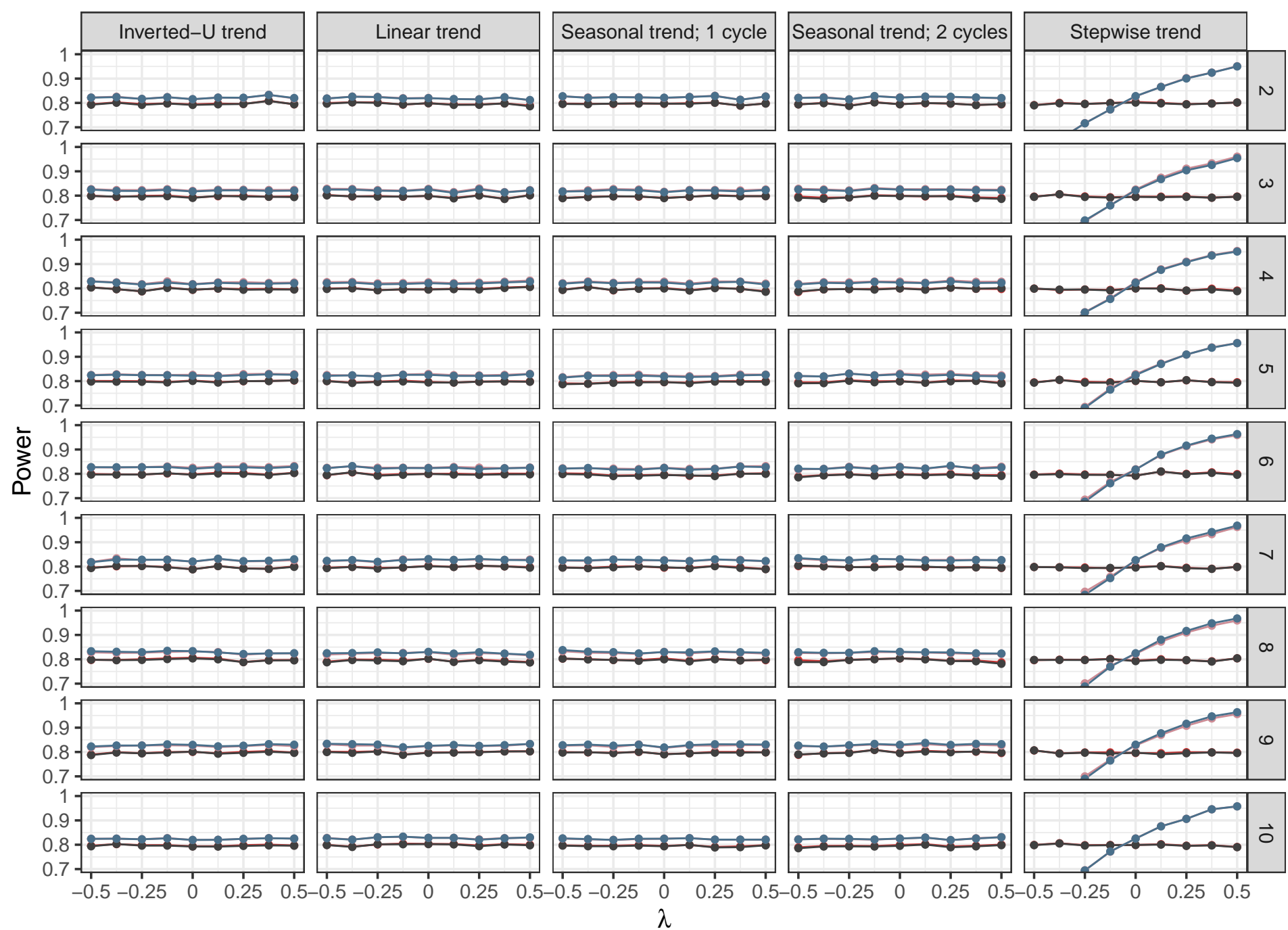

Analysis approach: Fixed – period Separate analysis Splines – period Splines – calendar

Supplement: Supplementary file 1 — Supporting Information [file BIMJ-67-e70059-s002.zip › simulations/figures/splines_alpha_pow_lambda_trend_all_arms.pdf]

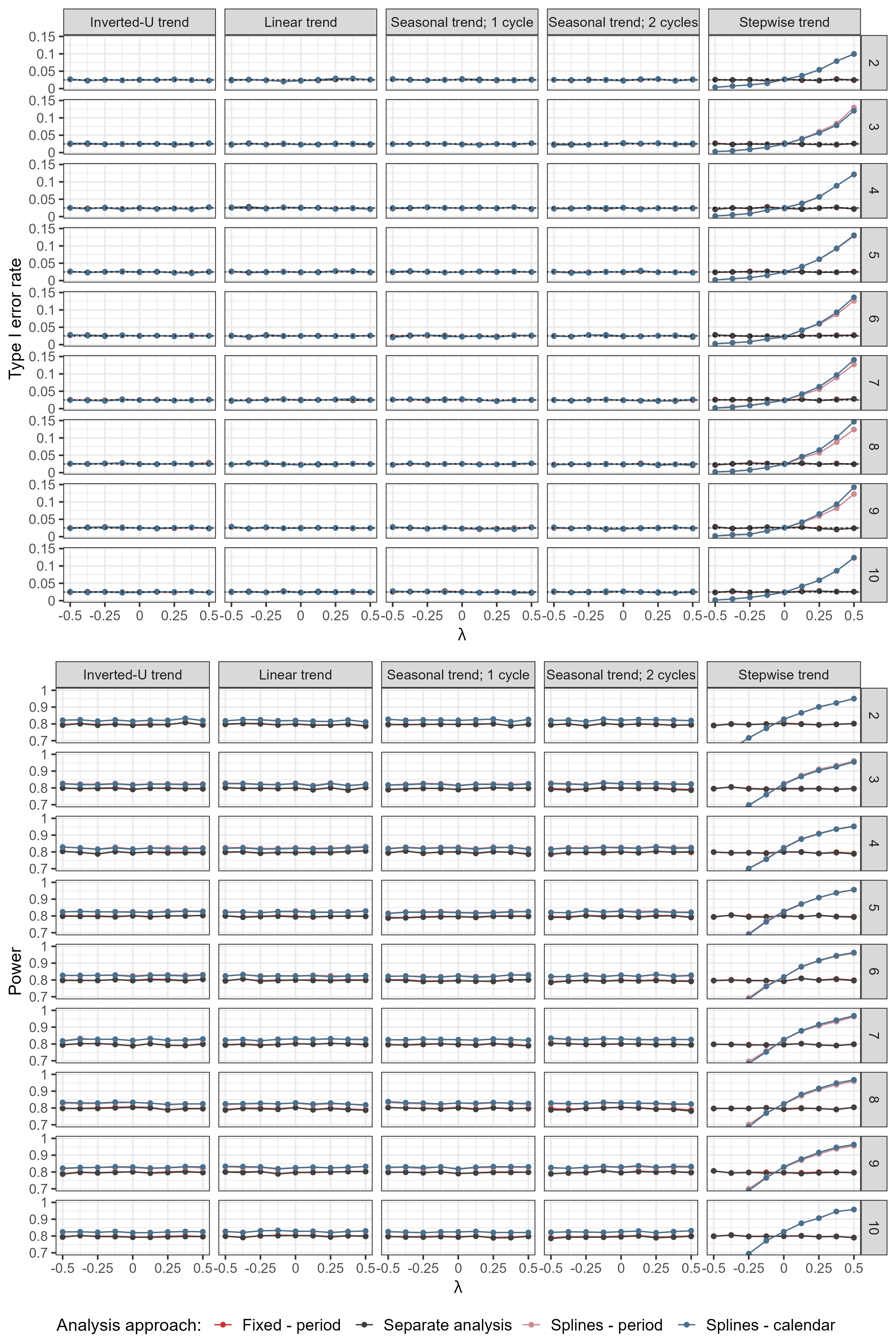

Supplement: Supplementary file 1 — Supporting Information [file BIMJ-67-e70059-s002.zip › simulations/figures/splines_alpha_pow_lambda_trend_all_arms.png]

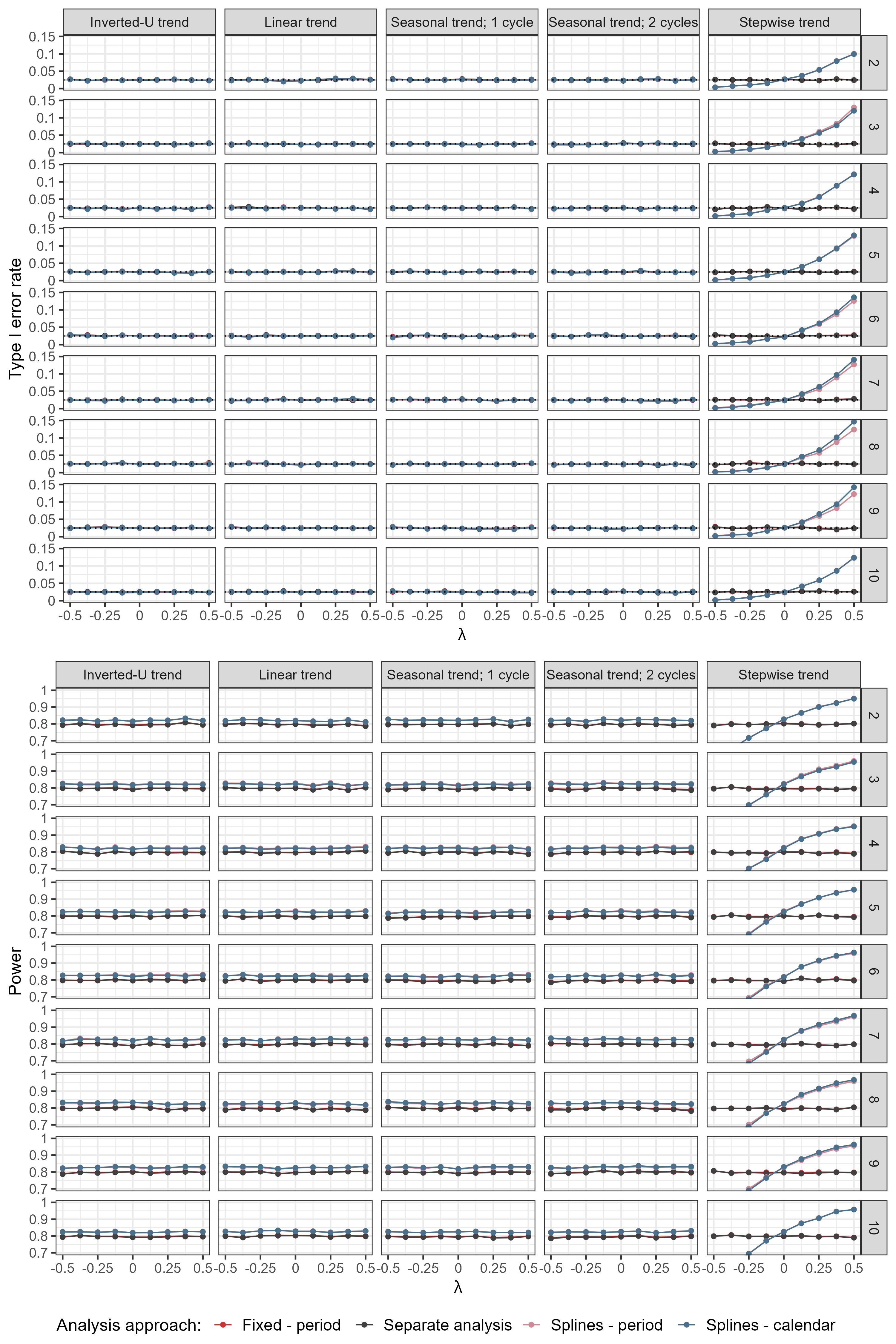

Supplement: Supplementary file 1 — Supporting Information [file BIMJ-67-e70059-s002.zip › simulations/figures/splines_alpha_pow_lambda_trend_all_arms.tiff]

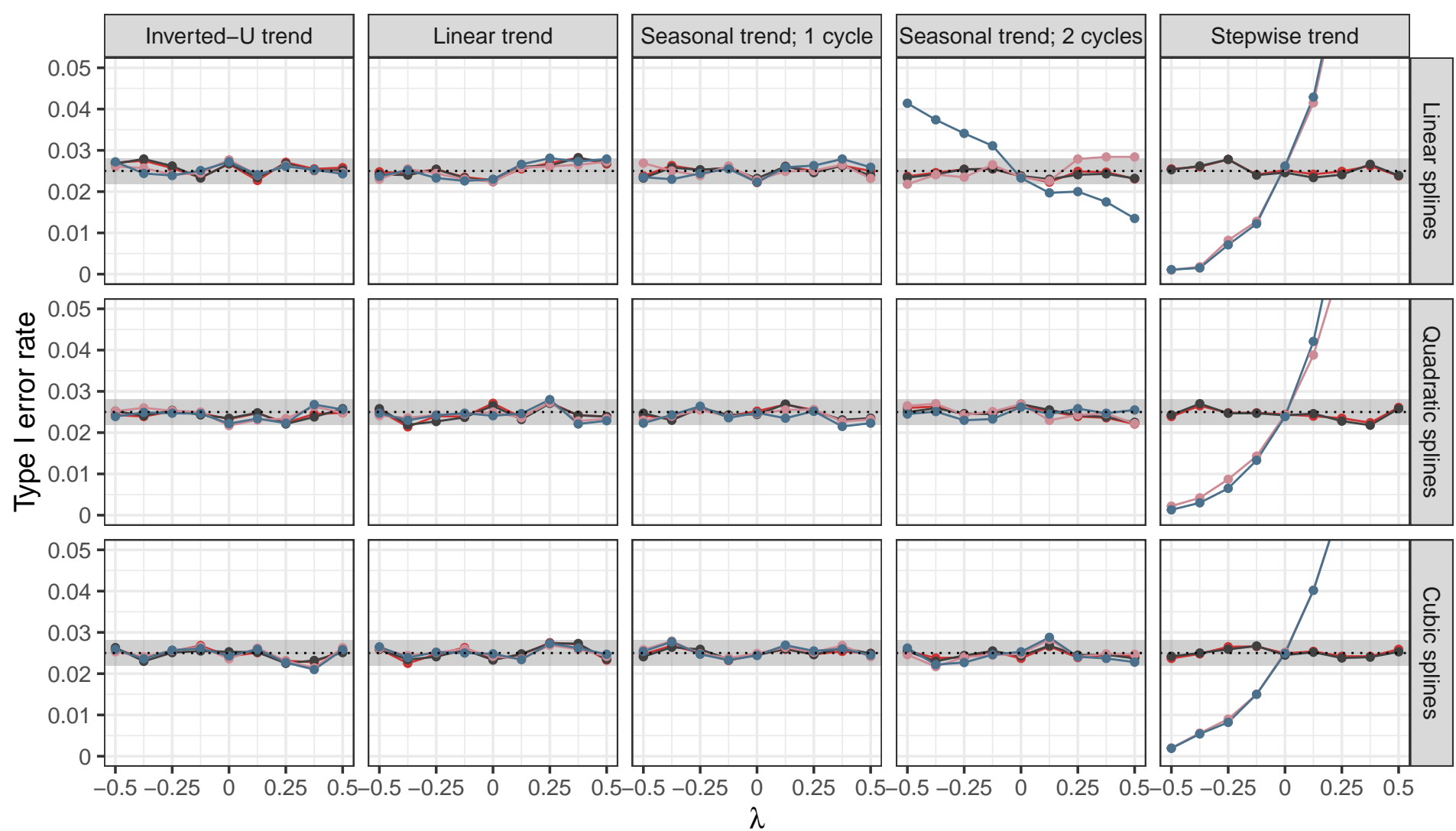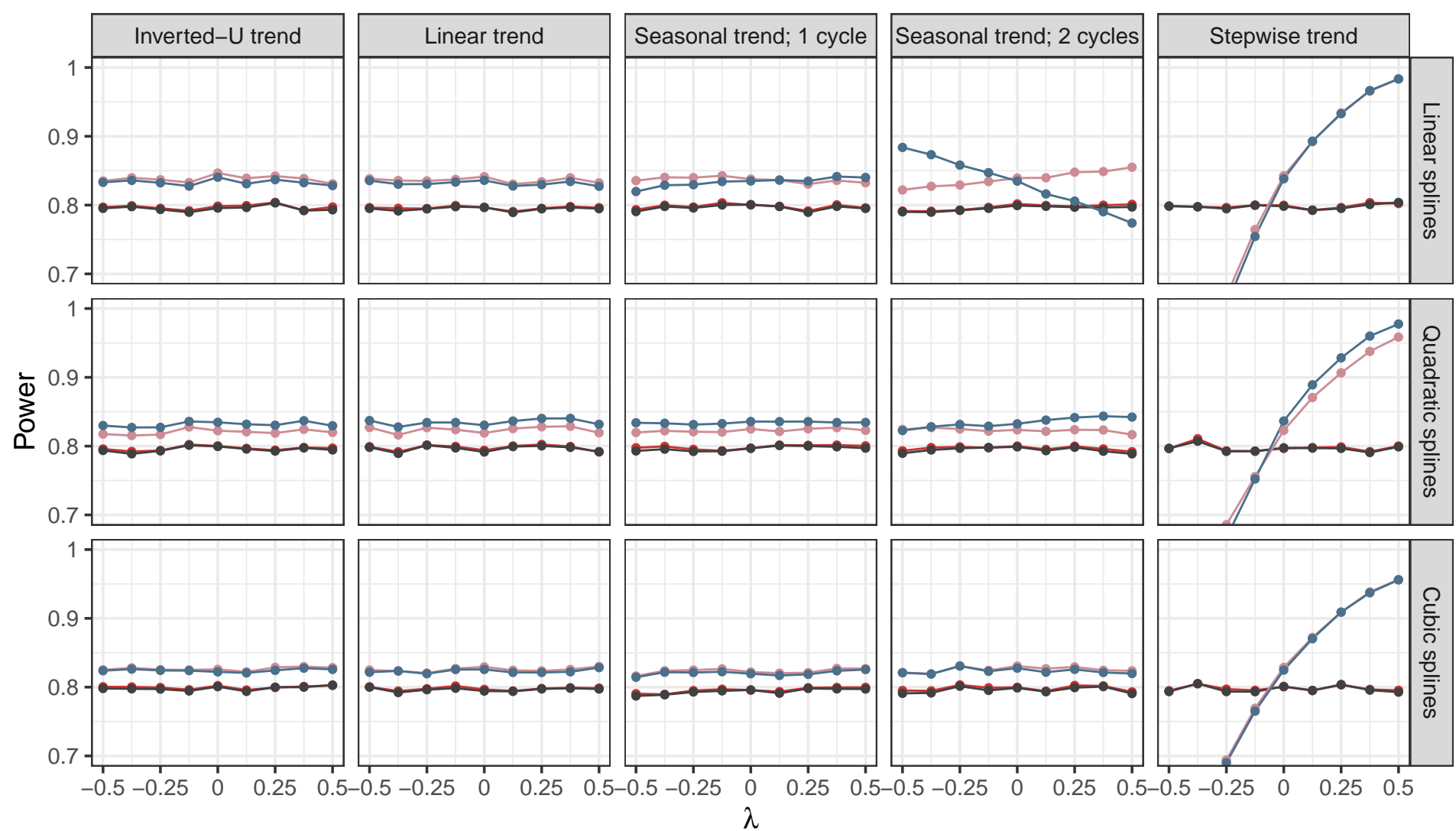

Analysis: —●— Fixed – period —●— Separate analysis —●— Splines – period —●— Splines – calendar

Supplement: Supplementary file 1 — Supporting Information [file BIMJ-67-e70059-s002.zip › simulations/figures/splines_alpha_pow_lambda_trend_all_degrees.pdf]

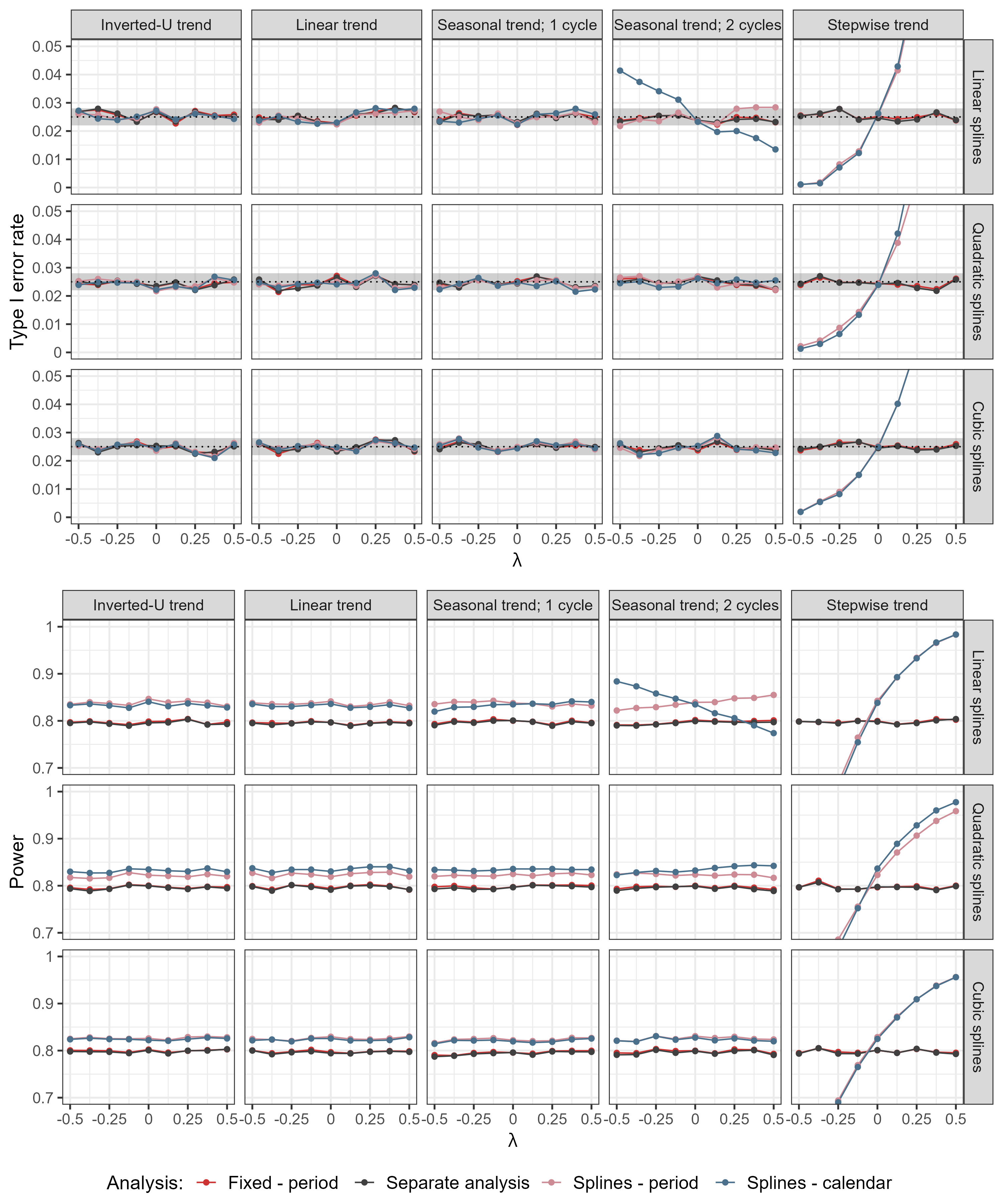

Supplement: Supplementary file 1 — Supporting Information [file BIMJ-67-e70059-s002.zip › simulations/figures/splines_alpha_pow_lambda_trend_all_degrees.png]

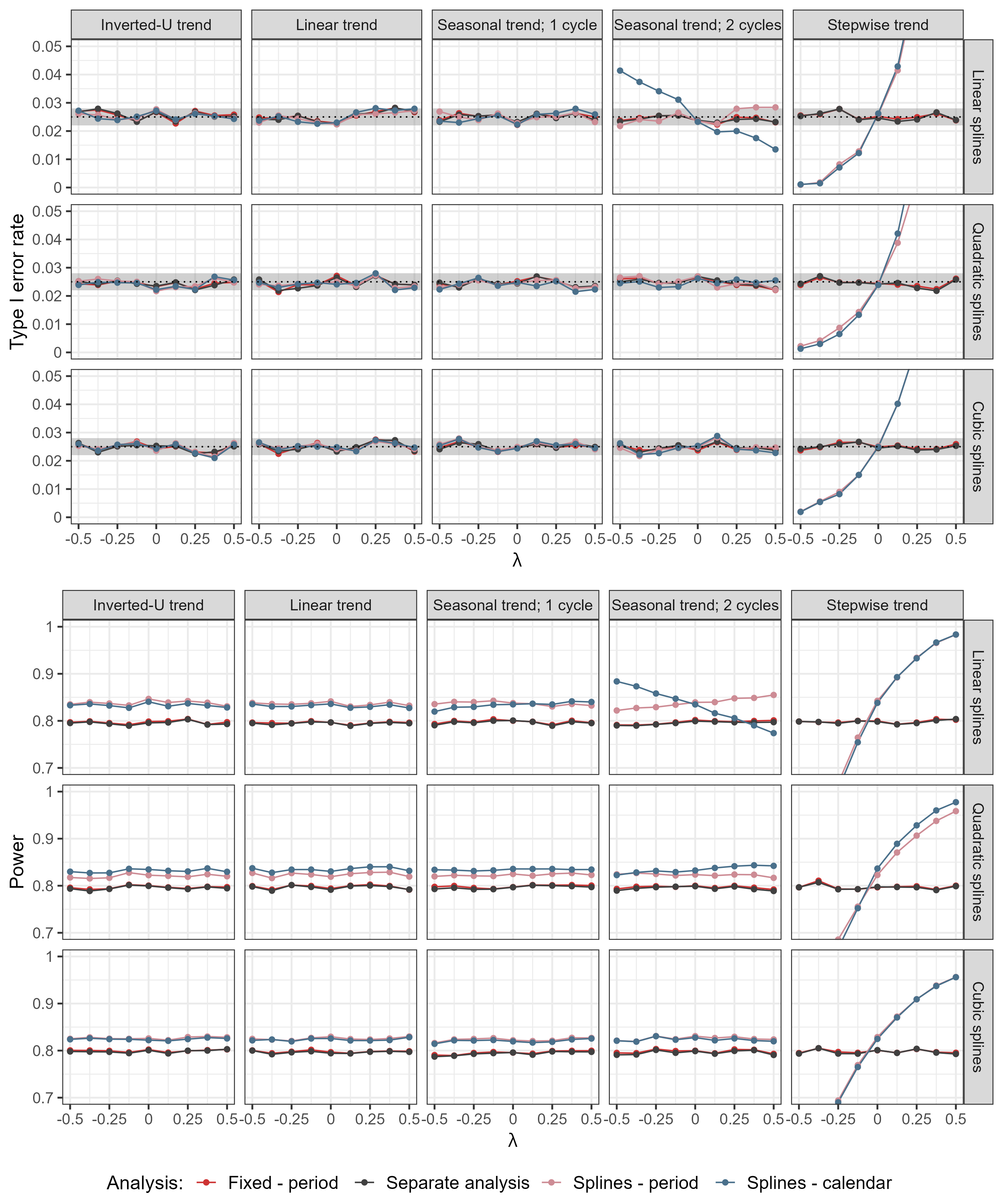

Supplement: Supplementary file 1 — Supporting Information [file BIMJ-67-e70059-s002.zip › simulations/figures/splines_alpha_pow_lambda_trend_all_degrees.tiff]

Mean response under  $H_0$

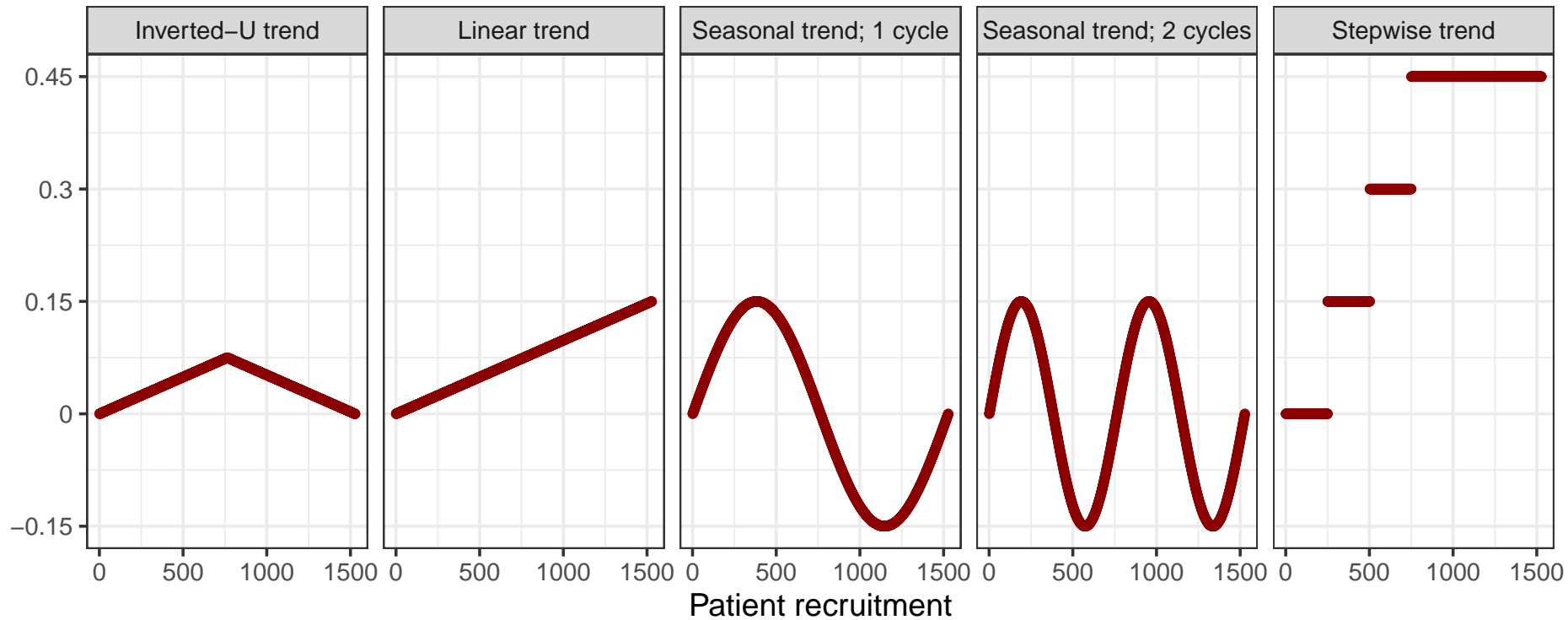

Supplement: Supplementary file 1 — Supporting Information [file BIMJ-67-e70059-s002.zip › simulations/figures/trend_patterns.pdf]

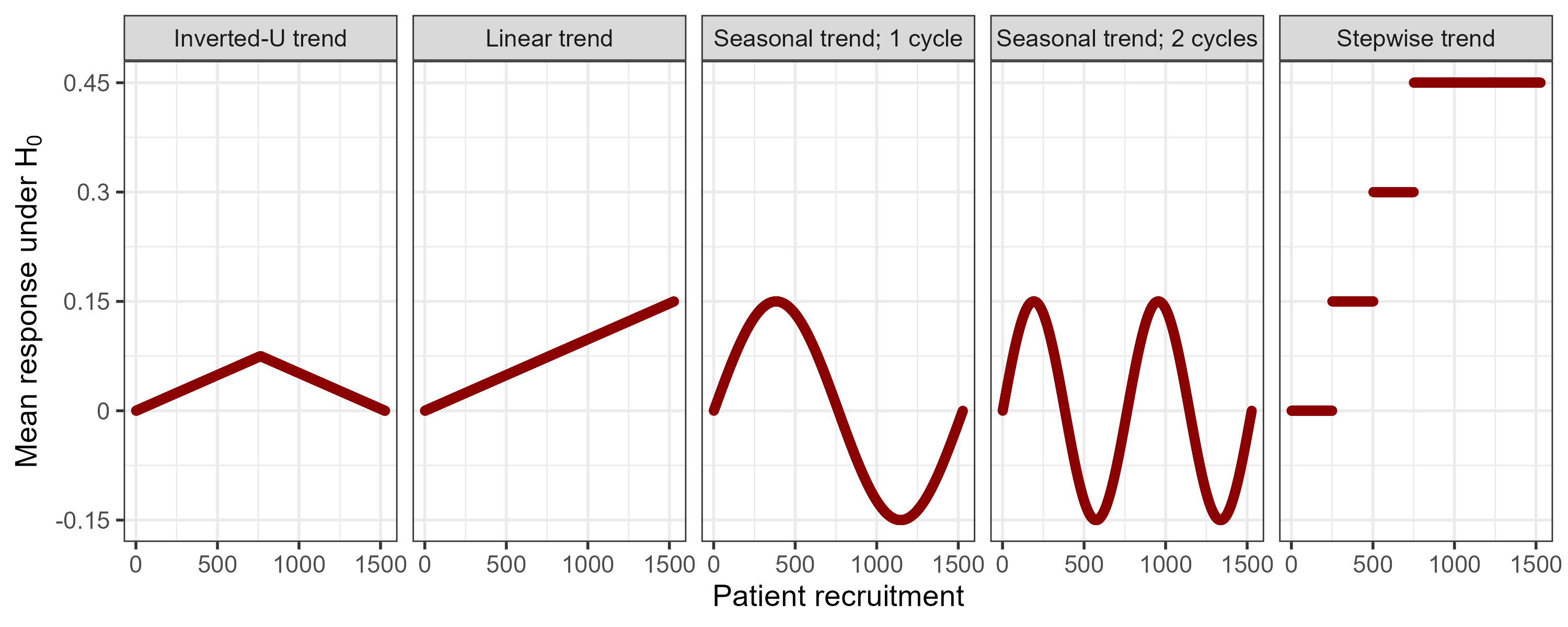

Supplement: Supplementary file 1 — Supporting Information [file BIMJ-67-e70059-s002.zip › simulations/figures/trend_patterns.png]

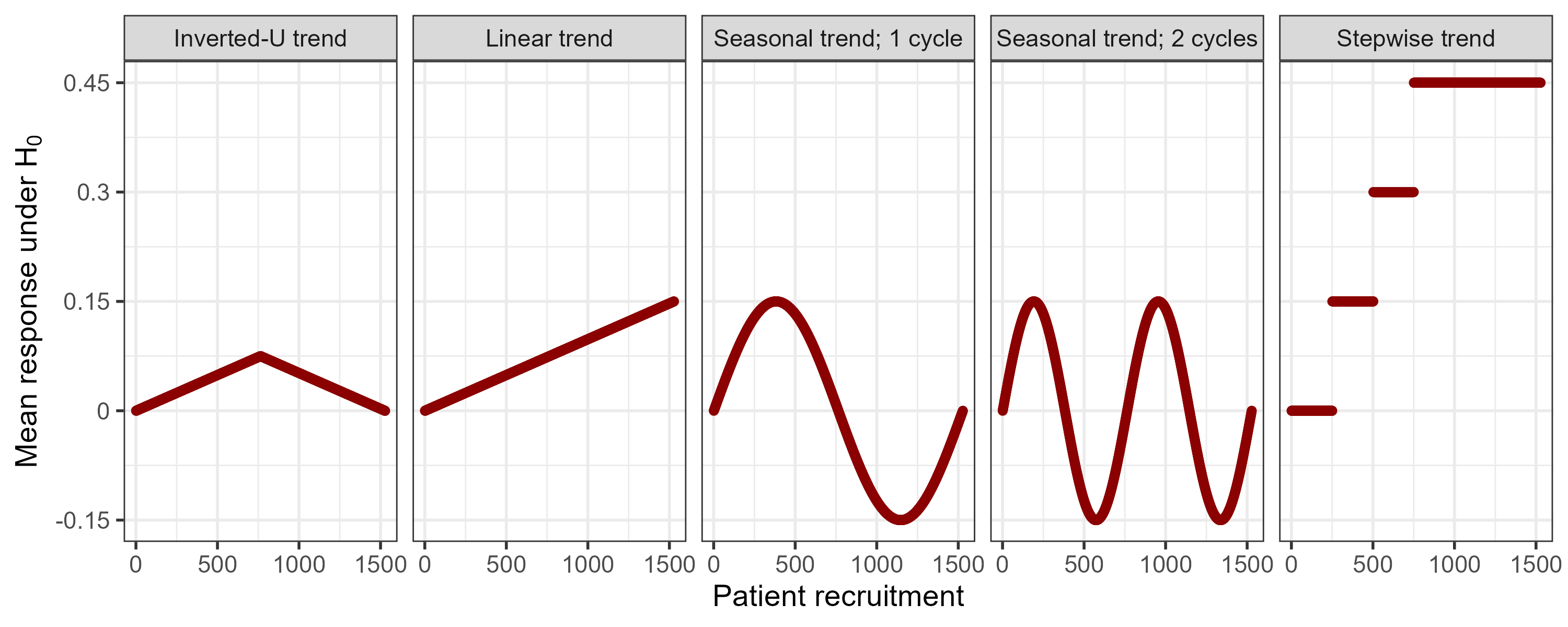

Supplement: Supplementary file 1 — Supporting Information [file BIMJ-67-e70059-s002.zip › simulations/figures/trend_patterns.tiff]
