# Supplementary material for: Statistical Modeling to Adjust for Time Trends in Adaptive Platform Trials Utilizing Non‐Concurrent Controls
Source: Biom J. 2025 Jun 10;67(3):e70059. doi: 10.1002/bimj.70059 (PMC12150008; doi:10.1002/bimj.70059)
Supplement: Supplementary file 2 — Supporting Information [file BIMJ-67-e70059-s001.pdf]

Supplementary material for "Statistical modeling to adjust for time trends in adaptive platform trials utilizing non-concurrent controls"

Pavla Krotka[1,2], Martin Posch[1], Mohamed Gewily[3], Günter Höglinger[4,5,6,7], and Marta Bofill Roig*[1,2]

[1] *Center for Medical Data Science, Medical University of Vienna, Vienna, Austria*
[2] *Department of Statistics and Operations Research, Universitat Politècnica de Catalunya, Barcelona, Spain*
[3] *Department of Pharmacy, Uppsala University, Uppsala, Sweden.*
[4] *Department of Neurology, LMU University Hospital, Ludwig-Maximilians-Universität (LMU) München, Munich, Germany.*
[5] *German Center for Neurodegenerative Diseases (DZNE), Munich, Germany.*
[6] *Munich Cluster for Systems Neurology (SyNergy), Munich, Germany.*
[7] *Department of Neurology, Hannover Medical School, Hanover, Germany.*

A Formal definition of the time variables

In this section, we present a formal definition of the considered periods, as well as units of calendar time in the platform trial.

We denote by k ($k \in \{1, \dots, K\}$) the indicator of the experimental treatment arm, and by s ($s \in \{1, \dots, S\}$) and c ($c \in \{1, \dots, C\}$) the period and calendar time unit indicator, respectively. For each experimental treatment arm k ($k \in \{1, \dots, K\}$), define $\mathbb{P}(k_j = k)$ as the probability that patient j is allocated to this arm. This probability is strictly positive when the arm is active in the trial (i.e. open for randomization when patient j is recruited) and 0 if the treatment arm is not yet or not anymore part of the platform, that is:

$$\mathbb{P}(k_j = k) = \begin{cases} \frac{1}{|\mathcal{K}_A(t_j)|+1} & \text{if } k \text{ is active} \\ 0 & \text{if } k \text{ is inactive} \end{cases}$$

where $\mathcal{K}_A(t) \subseteq \{1, \dots, K\}$ is the set of active experimental arms at time t. Note that formally, we say that treatment arm k is active at time t if $k \in \mathcal{K}_A(t)$ and inactive otherwise.

We define the entry and exit times of the treatment arm k by t_k^{entry} and t_k^{exit} such that:

$$k \in \mathcal{K}_A(t) \text{ if and only if } t \in [t_k^{entry}, t_k^{exit}]$$

Denoting by $\mathbf{t}^{entry,exit} = \{t_1^{entry}, t_1^{exit}, t_2^{entry}, t_2^{exit}, \dots t_K^{entry}, t_K^{exit}\}$ a vector containing the entry and exit times of all experimental arms, we define the end of a period s by t_s^{end}, considering:

$$t_1^{end} = \min\{t \in \mathbf{t}^{entry,exit} \mid t > 0\}$$
$$t_s^{end} = \min\{t \in \mathbf{t}^{entry,exit} \mid t > t_{s-1}^{end}\} \text{ for } s \in \{2, \dots, S\}$$

*marta.bofill.roig@upc.edu

where S is the total number of periods in the trial as before.

Finally, the time interval for a period s is given by:

$$T_1^S = [0, t_1^{end}]$$
$$T_s^S = (t_{s-1}^{end}, t_s^{end}] \text{ for } s \in \{2, \ldots, S\}$$

The trial can also be divided into equidistant units of calendar time. Given the length of the calendar time interval c_{length}, the trial consists of $C = \min\{x \in \mathbb{N} \mid x \geq t_S^{end}/c_{length}\}$ such intervals. The time interval for a calendar time unit c is then given by:

$$T_1^C = [0, c_{length}] \text{ for } c = 1$$
$$T_c^C = ((c-1) \cdot c_{length}, c \cdot c_{length}] \text{ for } c \in \{2, \ldots, C-1\}$$
$$T_C^C = ((C-1) \cdot c_{length}, t_S^{end}] \text{ for } c = C$$

B Further specification of the models for treatment-control comparisons

In this section, we provide technical details on the B-spline regression, as well as details on the structure of the covariance matrices in the mixed models with autocorrelated random effects.

B.1 Spline regression

In the spline regression described in Section 2.2, we consider the B-spline function to model the time trend. This function is composed of multiple polynomial functions of a given degree q, which are joined together at points called *knots*, such that the entire spline is continuously differentiable up to the $(q-1)$th derivative [1].

In our case, the knots are placed within the range of the patient entry times t_j. To define a B-spline function, we first define the knot sequence

$$\zeta_1 = \ldots = \zeta_q = \zeta_{q+1} < \zeta_{q+2} < \ldots < \zeta_{q+Z+1} < \zeta_{q+Z+2} = \zeta_{q+Z+3} = \ldots = \zeta_{2q+Z+2}$$

where the Z knots in the set $\{\zeta_{q+2}, \ldots, \zeta_{q+Z+1}\}$ are called *inner knots*, while ζ_{q+1} and ζ_{q+Z+2} are referred to as *boundary knots*. The additional knots $\{\zeta_1, \ldots, \zeta_q\}$, as well as $\{\zeta_{q+Z+3}, \ldots, \zeta_{Z+2q+2}\}$ are set equal to the boundary knots, even though their choice is essentially arbitrary and only needed because of the later recursive definition of the B-spline [2].

The function $f(t_j)$ can then be represented by a set of basis functions $B_i^q(t_j)$ as follows [3]:

$$f(t_j) = \sum_{i=1}^{q+Z+1} B_i^q(t_j)\beta_i$$

where β_i are the associated regression coefficients and the functions $B_i^q(t_j)$ are defined using the following formula, recursive in q, as follows:

$$B_i^q(t_j) = \frac{t_j - \zeta_i}{\zeta_{i+q} - \zeta_i} B_i^{q-1}(t_j) + \frac{\zeta_{i+q+1} - t_j}{\zeta_{i+q+1} - \zeta_{i+1}} B_{i+1}^{q-1}(t_j), \qquad i = 1, \ldots, q + Z + 1$$

with

$$B_i^0 = \begin{cases} 1 & \zeta_i \leq t_j < \zeta_{i+1} \\ 0 & \text{otherwise} \end{cases}$$

and

$$B_i^0 \equiv 0 \text{ if } \zeta_i = \zeta_{i+1}$$

As described in the main paper, we evaluate two strategies for the positions of the inner knots $\{\zeta_{q+2}, \ldots, \zeta_{q+Z+1}\}$. Firstly, we place the inner knots to the beginning of each period $s = 2, \ldots, S$, such that one polynomial of degree q is always fitted to each period. In this case, the number of inner knots $Z = S - 1$. Moreover, we consider placing the inner knots equidistantly, according to the length of the calendar time unit, and thus fitting a polynomial of degree q to each calendar time interval, which leads to $Z = C - 1$. The boundary knots ζ_{q+1} and ζ_{q+K+2} are always set to 1 and N, respectively, hence to the beginning and end of the trial.

B.2 Mixed-effect models with autocorrelated random effects

In the mixed-effect models with autocorrelated random effects, described in Section 2.3, the random effects u_s for individual periods are assumed to follow a normal distribution with mean 0, constant variance σ^2_{period} and an AR(1) correlation structure, i.e.:

$$\boldsymbol{u} \sim \mathcal{N}(0, \sigma^2_{period} \cdot \Sigma_{S_M \times S_M})$$

$$\Sigma_{S_M \times S_M} = \begin{bmatrix} 1 & \phi & \cdots & \phi^{S_M-1} \\ \phi & 1 & \cdots & \phi^{S_M-2} \\ \vdots & \vdots & \ddots & \vdots \\ \phi^{S_M-1} & \phi^{S_M-2} & \cdots & 1 \end{bmatrix}$$

An analogous distribution is assumed for the random effects u_c associated with different calendar times:

$$\boldsymbol{u} \sim \mathcal{N}(0, \sigma^2_{calendar} \cdot \Sigma_{C_M \times C_M})$$

$$\Sigma_{C_M \times C_M} = \begin{bmatrix} 1 & \phi & \cdots & \phi^{C_M-1} \\ \phi & 1 & \cdots & \phi^{C_M-2} \\ \vdots & \vdots & \ddots & \vdots \\ \phi^{C_M-1} & \phi^{C_M-2} & \cdots & 1 \end{bmatrix}$$

The parameter ϕ denotes the correlation between two adjacent periods or calendar time units. Note that ϕ can range from -1 to 1 and the correlation of periods that are w units apart is equal to ϕ^w, such that the correlation is weaker for periods or calendar time units that are further apart.

C Additional results from the simulation study

Figure S1: Setting 1A: Type I error rate and power of the fixed effect regression model with period adjustment compared to the pooled and separate analyses with respect to the strength of the time trend λ. In this example, $d = 400$ and $n = 250$ in each experimental arm and a linear shape of the time trend are used. Results for different experimental arms are shown in the rows.

Figure S2: Setting 1A: Type I error rate and power of the fixed effect regression model with period adjustment compared to the pooled and separate analyses with respect to the timing of adding the treatment arms. In this example, $n = 250$ in each experimental arm and a linear time trend with strength $\lambda = 0.5$ are considered. Results for different experimental arms are shown in the rows.

Figure S3: Setting 1A: Type I error rate and power of the regression model with period adjustment compared to the pooled and separate analyses with respect to the index of the evaluated arm. Here, $d = 200$, $n = 250$ in each experimental arm, and a linear time trend with strength $\lambda = 0.5$ are considered.

Figure S4: Setting 1B: Type I error rate and power for the spline regression model with knots according to periods or calendar time units compared to the regression model with period adjustment with respect to the timing of adding the treatment arms using different time trend patterns. In this example, $n = 250$ in each experimental arm, N_p placed to the middle of the trial in case of inverted-U trend, and $\lambda = 0.5$ are used. In case of calendar time adjustment, unit size of 450 patients is considered. Treatment arm 5 is being evaluated. Results for different degrees of the polynomial spline (linear, quadratic, cubic) are shown in the rows.

Figure S5: Setting 1B: Type I error rate and power for the spline regression model with knots according to periods or calendar time units compared to the regression model with period adjustment with respect to the strength of the time trend λ using different time trend patterns. In this example, $n = 250$ in each experimental arm and $N_p = 2500$ in case of inverted-U trend (corresponding to the middle of the trial) are used. In case of calendar time adjustment, unit size of 450 patients is considered. Treatment arm 5 is being evaluated. Results for different degrees of the polynomial spline (linear, quadratic, cubic) are shown in the rows.

Figure S6: Setting 1B: Type I error rate and power for the cubic spline regression model with knots according to periods or calendar time units compared to the regression model with period adjustment with respect to the timing of adding the treatment arms using different time trend patterns. In this example, $n = 250$ in each experimental arm, N_p placed to the middle of the trial in case of inverted-U trend, and $\lambda = 0.5$ are used. In case of calendar time adjustment, unit size of 450 patients is considered. Results for different experimental arms are shown in the rows.

Figure S7: Setting 1B: Type I error rate and power for the cubic spline regression model with knots according to periods or calendar time units compared to the regression model with period adjustment with respect to the strength of the time trend λ using different time trend patterns. In this example, $n = 250$ in each experimental arm, $N_p = 2500$ in case of inverted-U trend (corresponding to the middle of the trial) are used. In case of calendar time adjustment, unit size of 450 patients is considered. Results for different experimental arms are shown in the rows.

Figure S8: Setting 2A: Type I error rate and power of the regression model with calendar time adjustment compared to the regression model with period adjustment and separate analysis with respect to the size of the calendar time unit under different time trend patterns. In this example, $n = 250$ in each experimental arm, $\lambda = 0.125$ and $N_p = 750$ in case of inverted-U trend (corresponding approximately to the middle of the trial) are considered. Results for different experimental arms are shown in the rows.

Figure S9: Setting 2A: Type I error rate and power of the regression model with calendar time adjustment compared to the regression model with period adjustment and separate analysis with respect to the strength of the time trend λ under different time trend patterns. In this example, $n = 250$ in each experimental arm, $N_p = 750$ in case of inverted-U trend (corresponding approximately to the middle of the trial) and calendar time unit size of 100 are used. Results for different experimental arms are shown in the rows.

Figure S10: Setting 2B: Type I error rate and power of the mixed model with period and calendar time adjustments as uncorrelated and autocorrelated random effects, compared to the fixed effect regression model with period adjustment with respect to the pattern and strength of the time trend. Sample sizes of $n = 250$ in each experimental arm, $N_p = 750$ in case of inverted-U trend (corresponding approximately to the middle of the trial) are used. In case of calendar time adjustment, unit size of 100 patients is considered. Results for different experimental arms are shown in the rows.

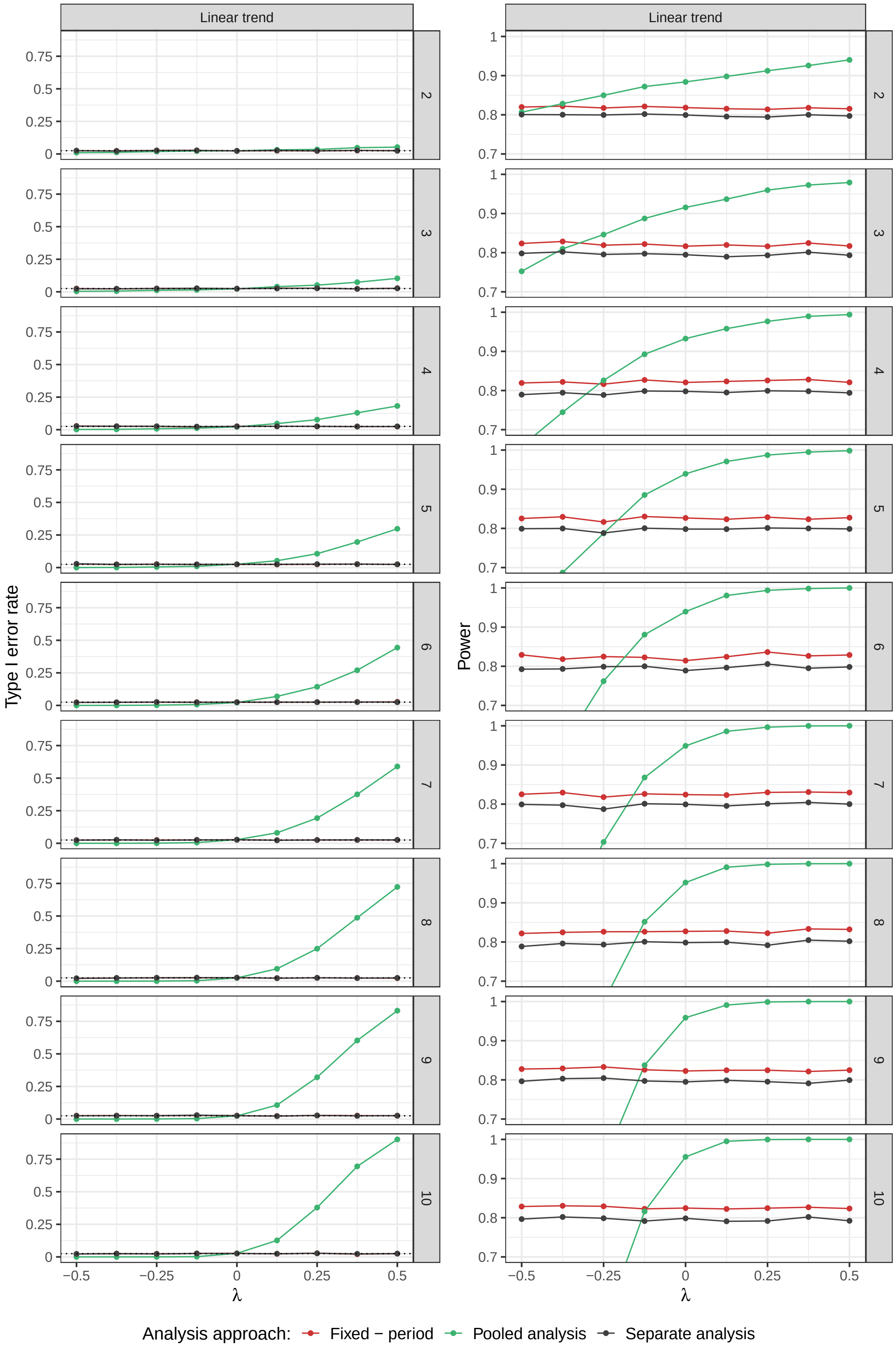

Linear trend
Linear trend
Type I error rate
Power
λ
λ
2
3
4
5
6
7
8
9
10
Analysis approach: Fixed − period Pooled analysis Separate analysis

Figure S1

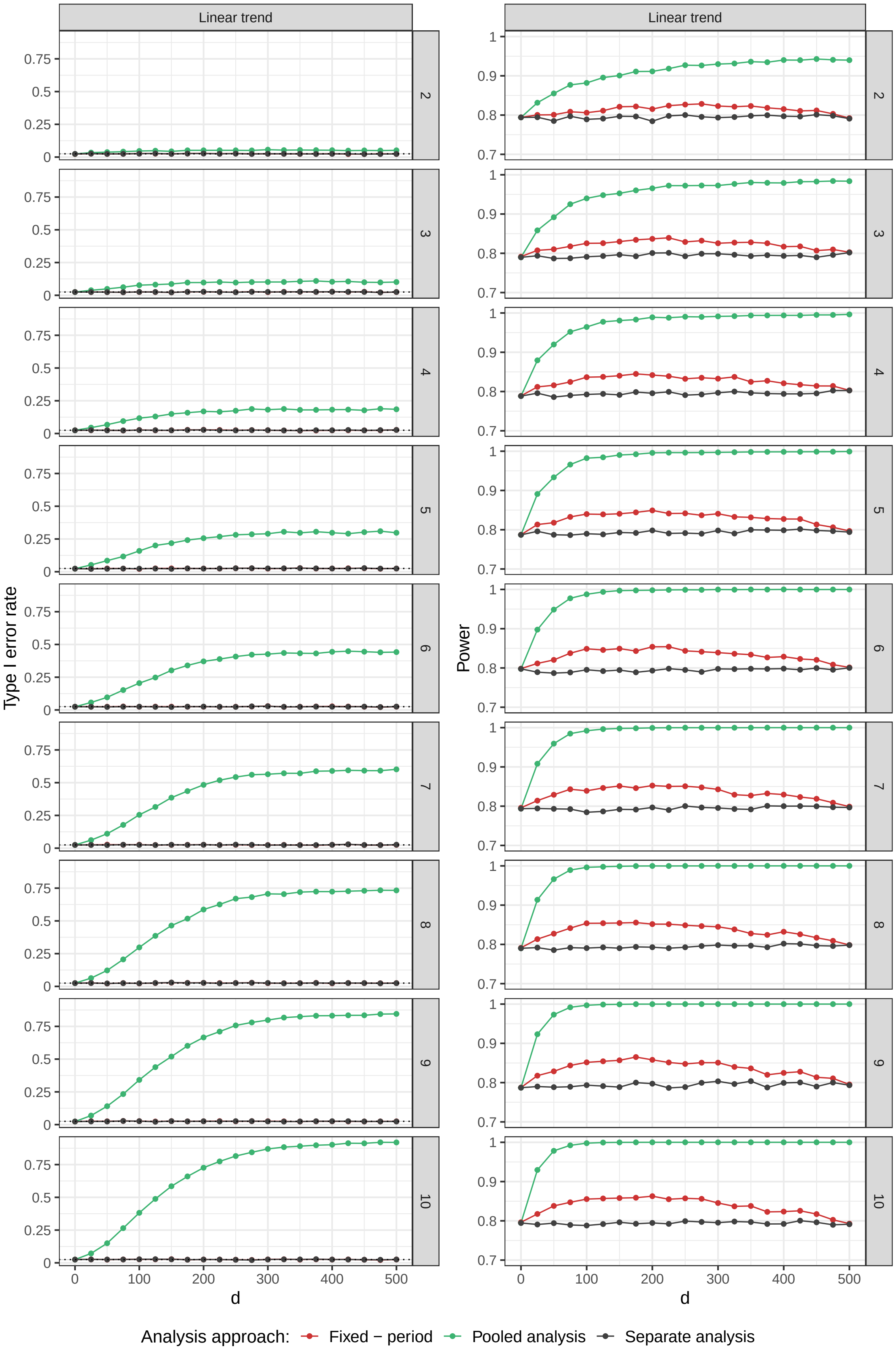
Linear trend
Linear trend
Type I error rate
Power
d
d
Analysis approach: Fixed − period Pooled analysis Separate analysis

Figure S2

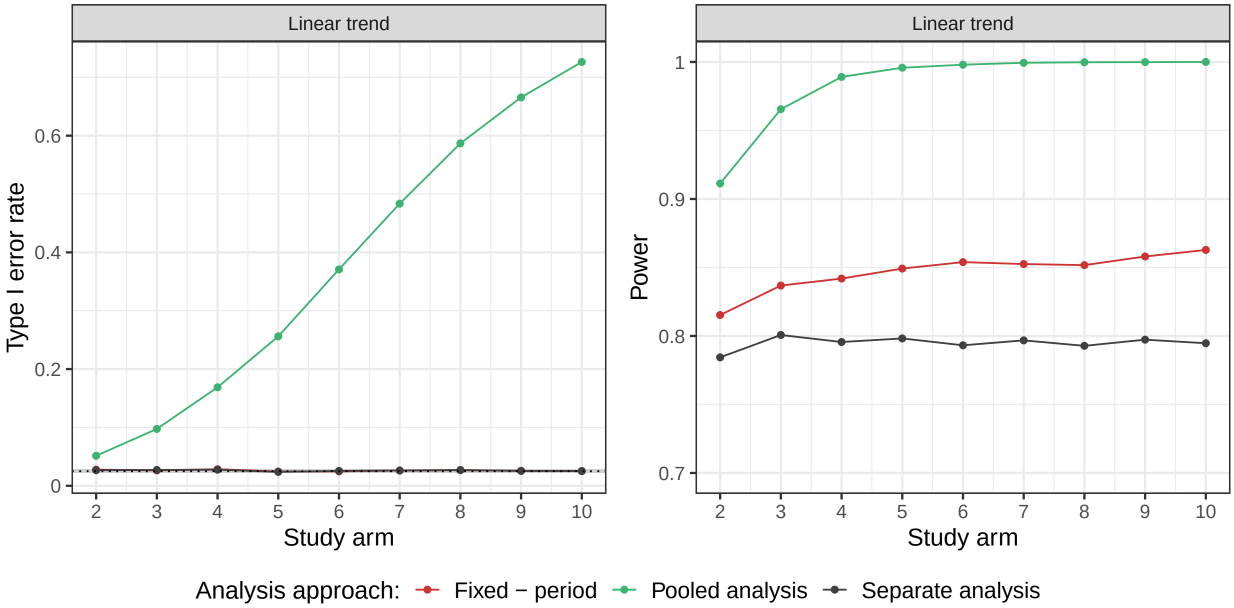
Linear trend
Type I error rate
0.6
0.4
0.2
0
2
3
4
5
6
7
8
9
10
Study arm
Linear trend
Power
1
0.9
0.8
0.7
Study arm
Analysis approach:
Fixed − period
Pooled analysis
Separate analysis

Figure S3

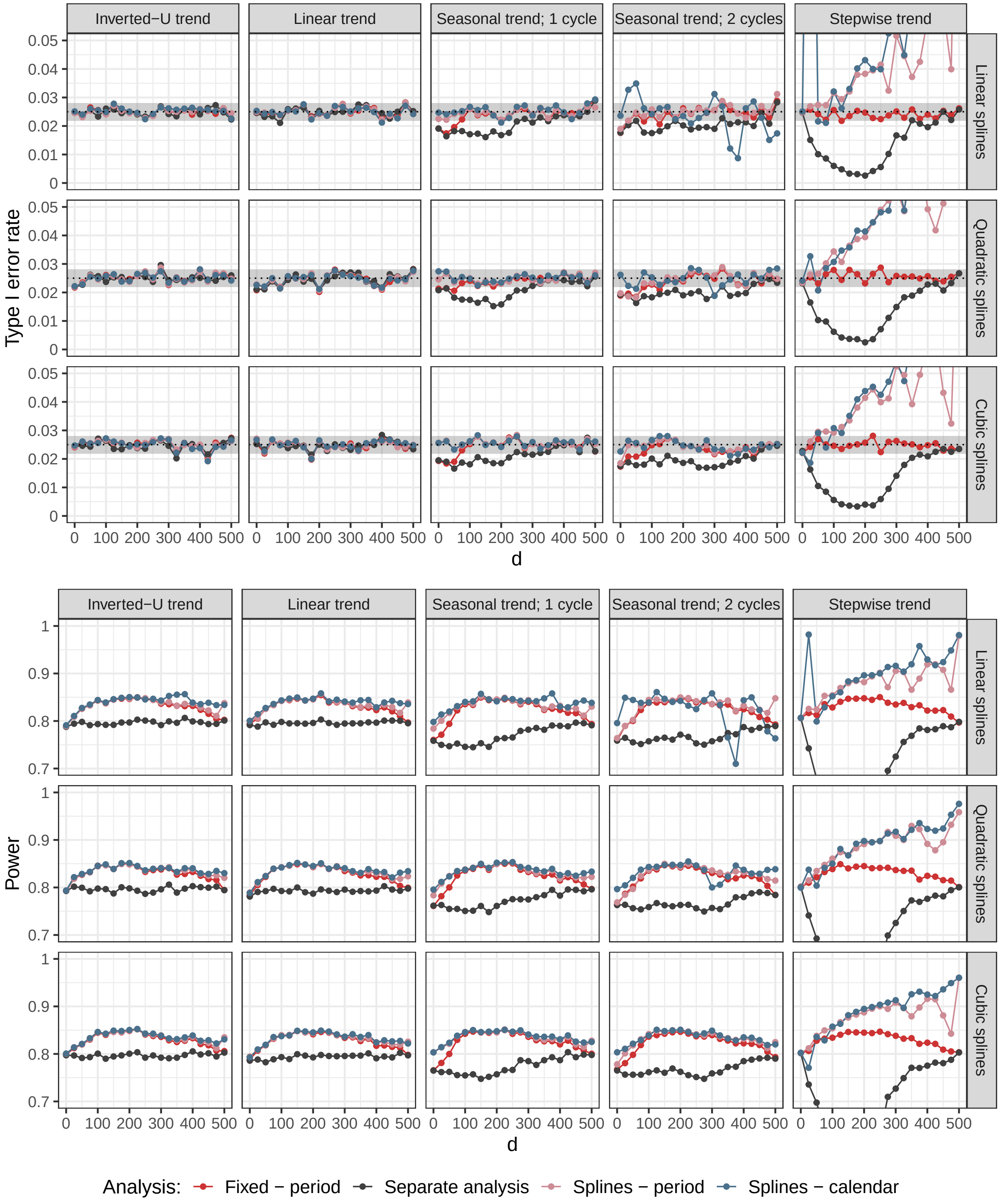
Inverted−U trend
Linear trend
Seasonal trend; 1 cycle
Seasonal trend; 2 cycles
Stepwise trend
Linear splines
Quadratic splines
Cubic splines
Type I error rate
Power
d
Analysis: Fixed − period Separate analysis Splines − period Splines − calendar

Figure S4

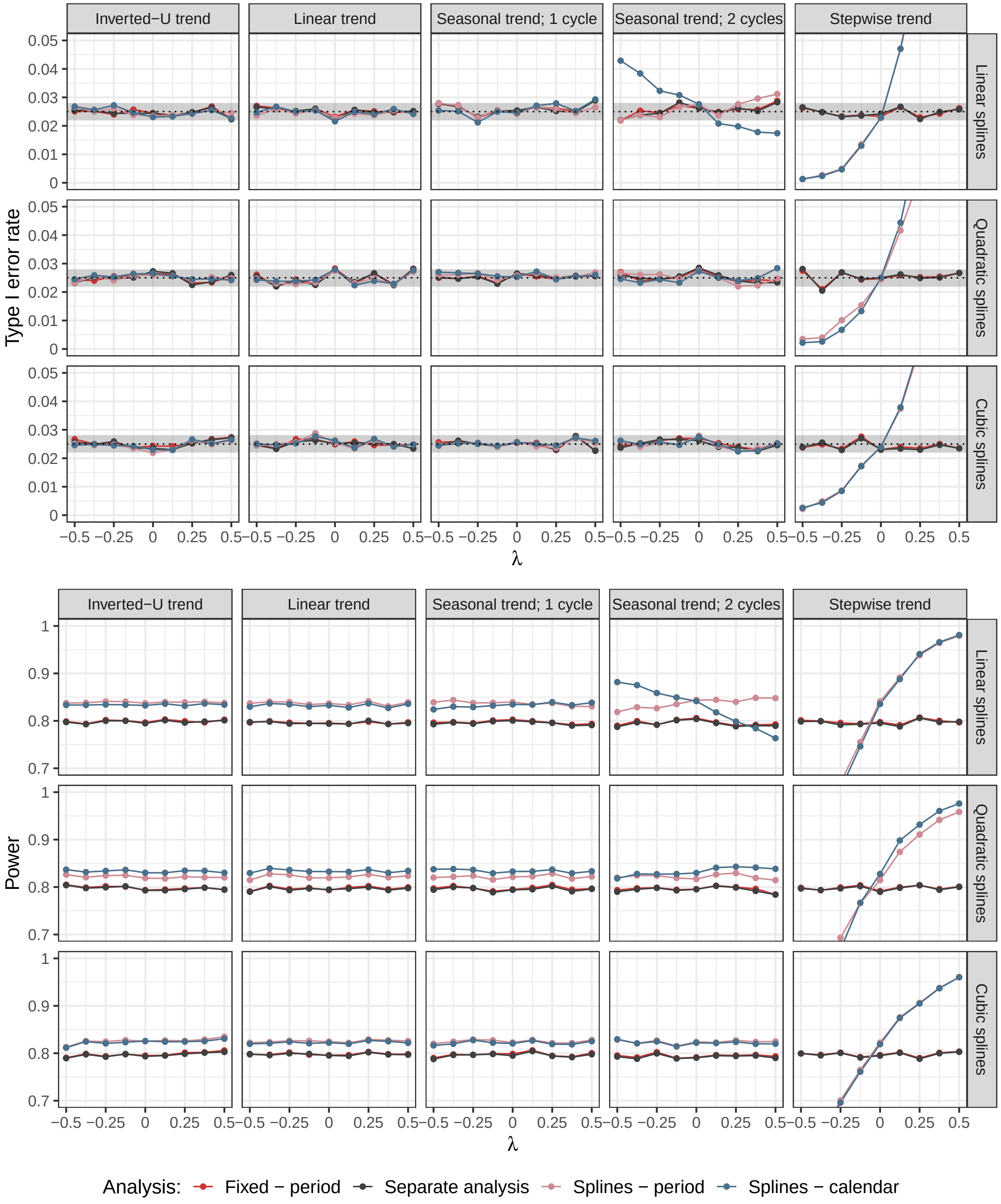

Inverted−U trend
Linear trend
Seasonal trend; 1 cycle
Seasonal trend; 2 cycles
Stepwise trend
Linear splines
Quadratic splines
Cubic splines
Type I error rate
Power
λ
Analysis: Fixed − period Separate analysis Splines − period Splines − calendar

Figure S5

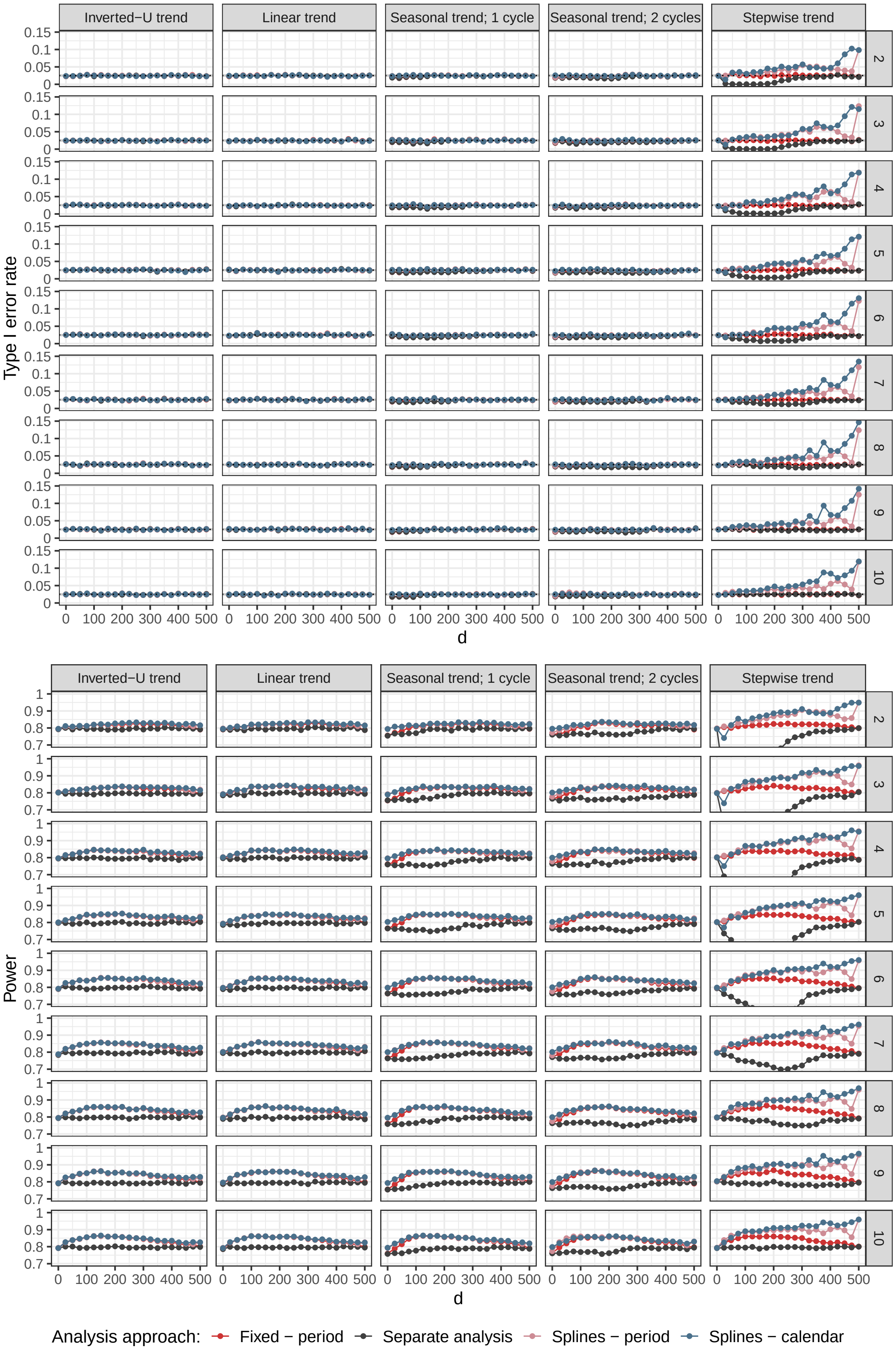
Inverted−U trend
Linear trend
Seasonal trend; 1 cycle
Seasonal trend; 2 cycles
Stepwise trend
Type I error rate
Power
d
Analysis approach: Fixed − period Separate analysis Splines − period Splines − calendar

Figure S6

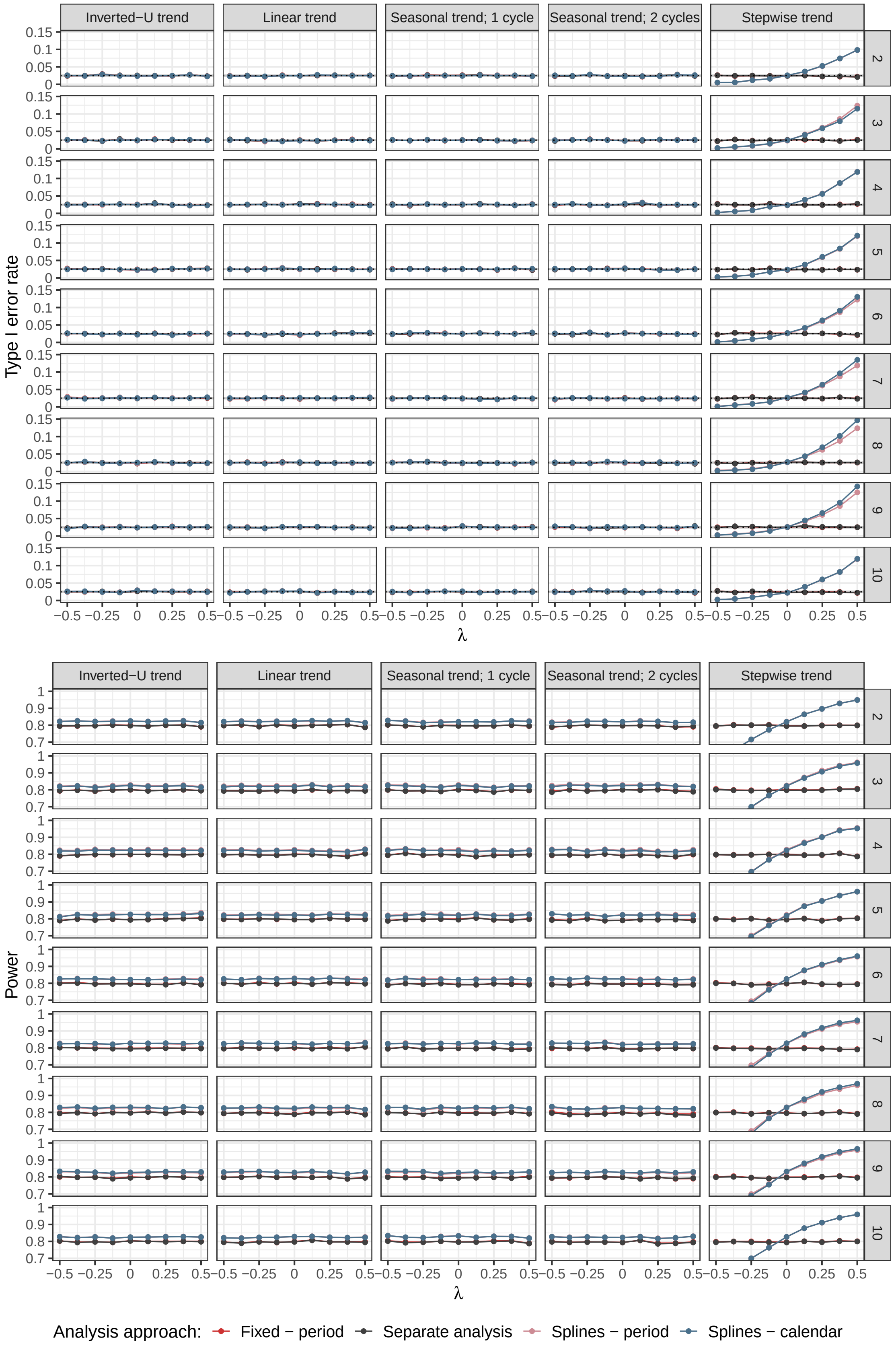
Inverted−U trend
Linear trend
Seasonal trend; 1 cycle
Seasonal trend; 2 cycles
Stepwise trend
Type I error rate
Power
λ
Analysis approach: Fixed − period Separate analysis Splines − period Splines − calendar

Figure S7

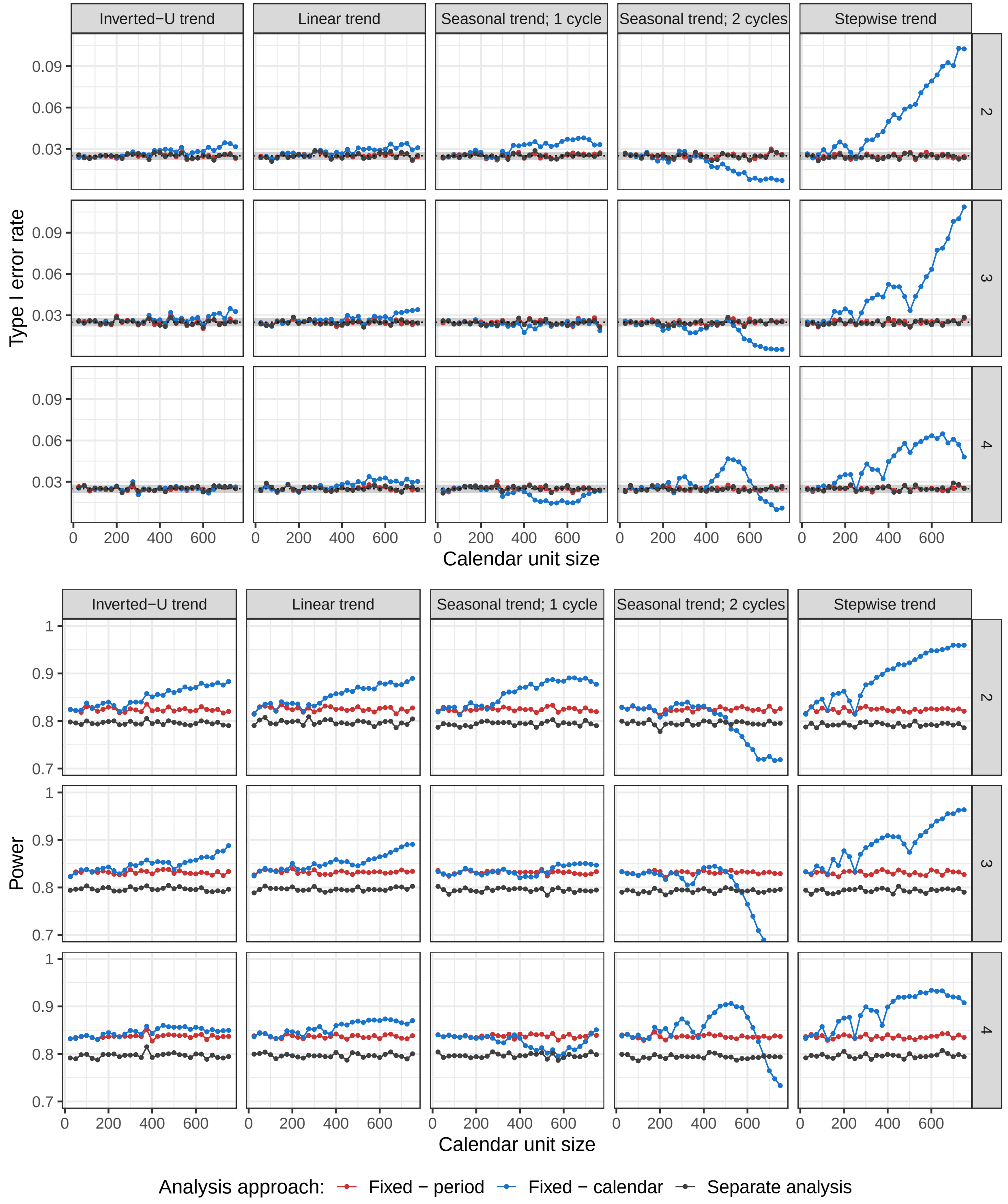
Inverted−U trend
Linear trend
Seasonal trend; 1 cycle
Seasonal trend; 2 cycles
Stepwise trend
Type I error rate
Power
Calendar unit size
2
3
4
Analysis approach: Fixed − period Fixed − calendar Separate analysis

Figure S8

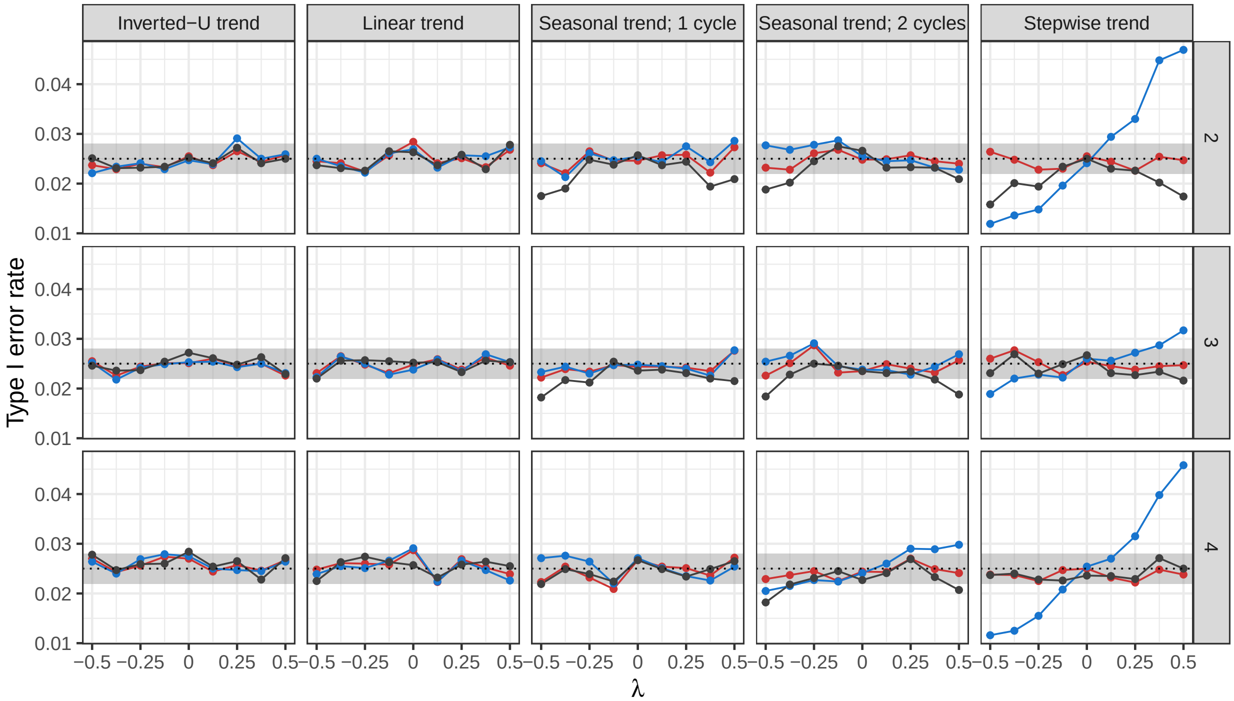
Inverted−U trend
Linear trend
Seasonal trend; 1 cycle
Seasonal trend; 2 cycles
Stepwise trend
2
3
4
Type I error rate
0.01
0.02
0.03
0.04
−0.5 −0.25 0 0.25 0.5
λ

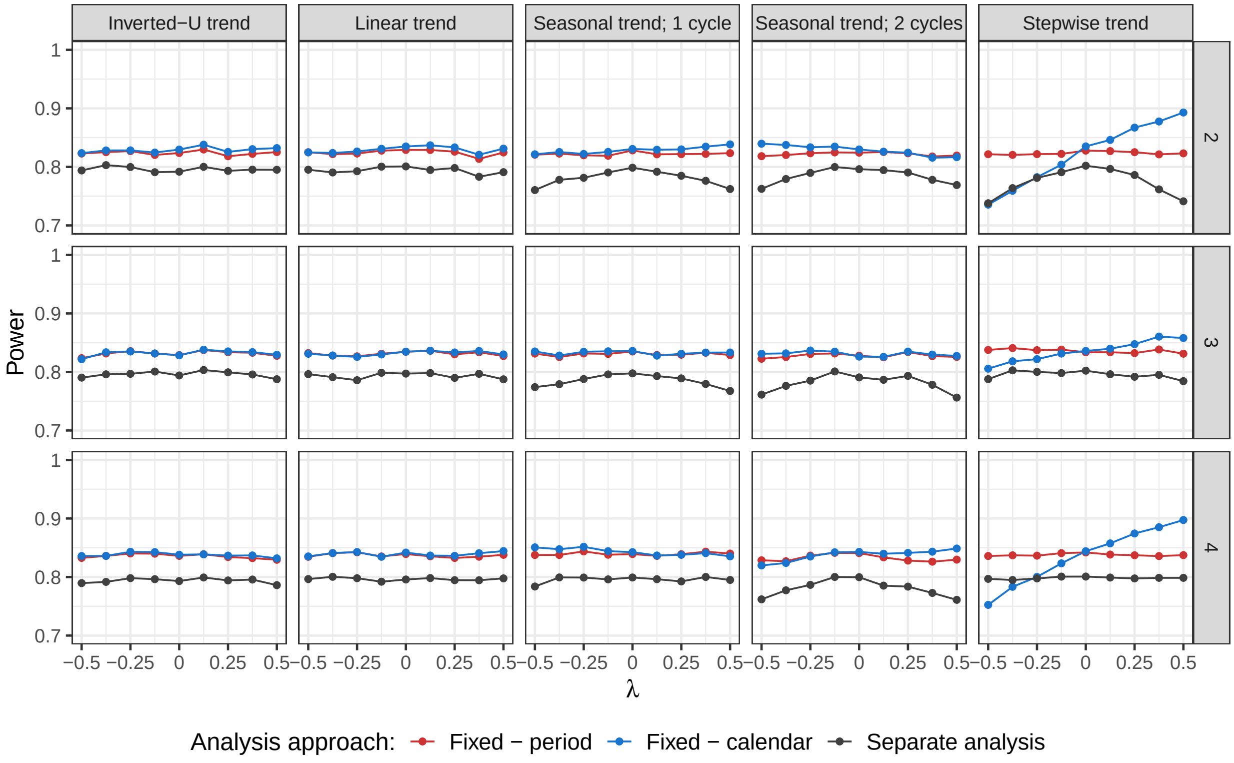
Inverted−U trend
Linear trend
Seasonal trend; 1 cycle
Seasonal trend; 2 cycles
Stepwise trend
2
3
4
Power
0.7
0.8
0.9
1
−0.5 −0.25 0 0.25 0.5
λ
Analysis approach: Fixed − period Fixed − calendar Separate analysis

Figure S9

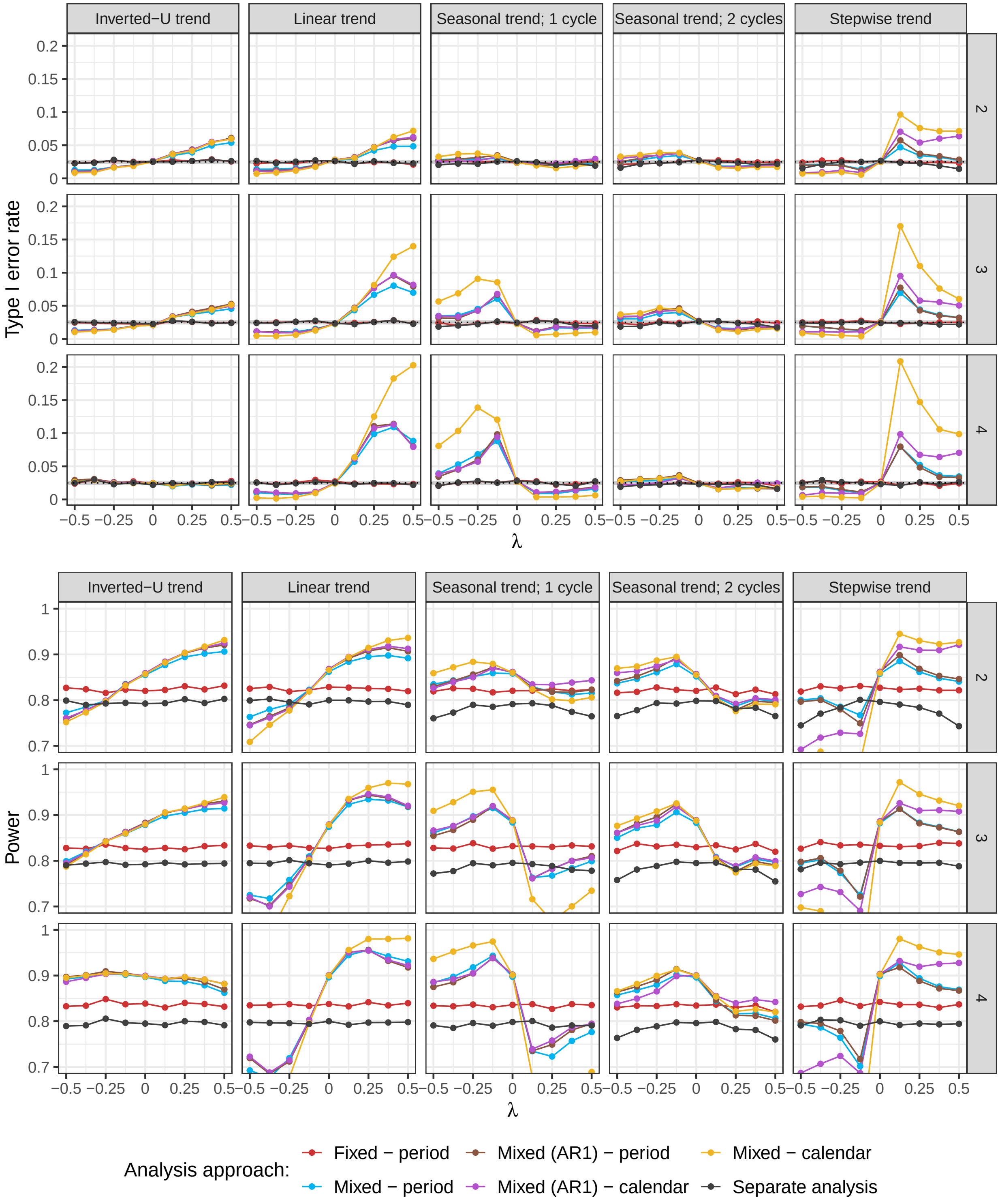
Inverted−U trend
Linear trend
Seasonal trend; 1 cycle
Seasonal trend; 2 cycles
Stepwise trend
Type I error rate
Power
λ
2
3
4
Analysis approach:
Fixed − period
Mixed − period
Mixed (AR1) − period
Mixed (AR1) − calendar
Mixed − calendar
Separate analysis

Figure S10

D Additional case study: FLAIR trial for chronic lymphocytic leukemia

We revisit the FLAIR trial, a recent platform trial for chronic lymphocytic leukemia [4, 5, 6]. The trial started in September 2014 and was originally designed to evaluate one experimental treatment arm (ibrutinib and rituximab (IR), denoted as arm 1) against a control arm (fludarabine, cyclophosphamide, and rituximab (FCR)). However, during the recruitment, new evidence emerged of another promising treatment (ibrutinib and venetoclax (IV), denoted as arm 2), which led to the addition of another experimental arm, together with an additional control arm (ibrutinib monotherapy (I)) in July 2017. Arm 1 was completed in June 2018, while the additional arm 2 continued recruitment. We consider the course of the trial until March 2021, where the experimental arm 2 was evaluated against the original control group (FCR), resulting in a 3-period platform trial. However, the actual trial continued to period 4, recruiting patients to further assess the efficacy of arm 2 against the additional control arm (I).

We consider a platform trial that imitates the design aspects of the FLAIR trial to show the use of the suggested analysis methods in a realistic scenario. We simulate a trial with 3 arms, emulating the arms FCR (shared control), IR (arm 1), and IV (arm 2) from the FLAIR trial, using the original sample sizes. The scheme of the simulated platform trial with the considered sample sizes is presented in Figure S11. The FLAIR trial considered a time-to-event endpoint (progression-free survival) as the primary outcome. The estimated hazard ratios for comparing the experimental arms 1 and 2 to control were 0.44 [5] and 0.13 [4], respectively. We assume a platform trial with continuous endpoints for illustrative purposes, and consider effect sizes of -log(HR) by analogy. Hence, we use treatment effects of $\theta_1 = 0.82$ and $\theta_2 = 2.04$. $\eta_0 = 0$ was assumed for the mean response of the control arm. The data was generated using full randomization and variance $\sigma^2 = 1$. Furthermore, for illustrative purposes, we introduced time trends of different patterns and strengths. In particular, we considered linear and stepwise time trends (as defined in Section 4.1.1 in the main paper), and strengths corresponding to moderate trends ($\lambda = 0.25$), large trends ($\lambda = 1.5$) and extreme trends ($\lambda = 5$).

We test the null hypothesis $H_{0,2} : \theta_2 = 0$ against the two-sided alternative, taking into account data from the whole platform trial.

Subsequently, we use the proposed modeling approaches to test the null hypothesis $H_{0,2}$. In particular, we consider the fixed effect models, as well as mixed effect models with uncorrelated and autocorrelated random effects, both with period and calendar time adjustment, and spline regression, placing the inner knots according to periods or calendar time units. In case of the calendar time adjustment, we use unit size of 100 patients, separating the trial into 13 units.

Note that, for this case study, we used a different data generation strategy compared to the simulation study in the main paper. In the main paper, independent simulations were conducted for each considered time trend (i.e., separate datasets were simulated from normal distributions with means $\eta_0 + \sum_{k=1,2} \theta_k \cdot I(k_j = k) + f(j)$ for each time trend f). For the case study, however, we first simulated a dataset without time trends (sampling from a normal distribution with means $\eta_0 + \sum_{k=1,2} \theta_k \cdot I(k_j = k)$ for patient j allocated to arm k_j, where $k_j = 0, 1, 2$) and subsequently shifted the simulated datasets by the different time trends f. Hence, the underlying means were the same in all considered scenarios, and the only difference was induced by adding the time trends of different patterns and strengths. This approach allows observed differences in estimates and hypothesis tests between time trends to be attributed more directly to the time trends themselves rather than to sampling error.

The resulting treatment effect estimates and standard errors for all scenarios are presented in Table S1.

All considered approaches resulted in the rejection of the null hypothesis $H_{0,2}$ with a p-value $p < 0.001$ in all scenarios. For this simulated data set, the fixed effect model with period adjustment gives the closest effect estimates to the separate analysis. Under linear time trends, the differences between all the model-based approaches are minimal. Under stepwise time trends, adjusting for calendar time units as a fixed or random

effect results in somewhat larger treatment effect estimates compared to the period adjustment in the same model. The pooled analysis gives notably larger estimates in all considered cases. The spline regression with period adjustment leads to larger estimation in settings with stepwise time trend compared to scenarios with linear time trend.

Since the treatment effect estimates presented in Table S1 are from a single simulated dataset, and it is difficult to see general tendencies, we have additionally evaluated the estimation error of the model-based approaches with period adjustment using 10,000 simulation runs. The resulting histograms of the estimation error for a scenario with a stepwise time trend with $\lambda = 0.25$ are shown in Figure S12. We can see that the mixed models, as well as the spline regression yield a slight bias in the treatment effect estimates, while the fixed effect model leads to unbiased estimation.

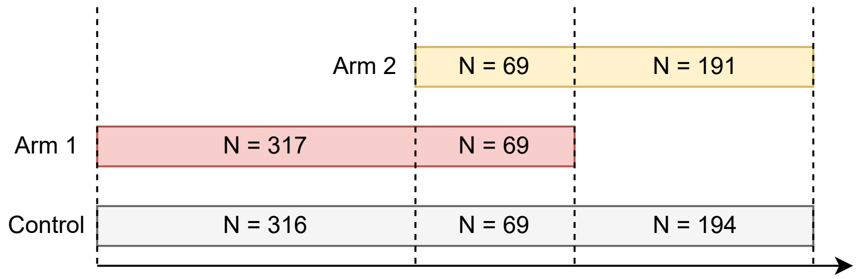

Figure S11: Scheme of the platform trials considered in the case study for chronic lymphocytic leukemia (FLAIR trial). Sample sizes per arm and period are shown.

Analysis approach	Adjustment	Linear time trend			Stepwise time trend		
		$\lambda = 0.25$	$\lambda = 1.5$	$\lambda = 5$	$\lambda = 0.25$	$\lambda = 1.5$	$\lambda = 5$
		**Effect est. (SE)**	**Effect est. (SE)**	**Effect. est. (SE)**	**Effect est. (SE)**	**Effect est. (SE)**	**Effect est. (SE)**
Fixed effect model	Periods	2.037 (0.085)	2.034 (0.087)	2.026 (0.100)	2.037 (0.085)	2.037 (0.085)	2.037 (0.085)
	Cal. time units	2.044 (0.085)	2.047 (0.085)	2.055 (0.086)	2.054 (0.085)	2.105 (0.087)	2.250 (0.102)
Mixed model	Periods	2.057 (0.083)	2.041 (0.087)	2.029 (0.100)	2.056 (0.084)	2.041 (0.085)	2.038 (0.085)
	Cal. time units	2.110 (0.078)	2.077 (0.084)	2.065 (0.085)	2.140 (0.080)	2.129 (0.086)	2.260 (0.102)
Mixed model (AR1)	Periods	2.071 (0.082)	2.044 (0.086)	2.031 (0.099)	2.066 (0.084)	2.043 (0.085)	2.039 (0.085)
	Cal. time units	2.078 (0.081)	2.057 (0.084)	2.061 (0.085)	2.093 (0.082)	2.122 (0.086)	2.258 (0.102)
Spline regression	Periods	2.036 (0.085)	2.036 (0.085)	2.036 (0.085)	2.048 (0.085)	2.109 (0.087)	2.280 (0.106)
	Cal. time units	2.037 (0.085)	2.037 (0.085)	2.037 (0.085)	2.042 (0.085)	2.064 (0.086)	2.128 (0.092)
Pooled analysis	-	2.134 (0.074)	2.488 (0.081)	3.479 (0.125)	2.200 (0.075)	2.882 (0.089)	4.792 (0.174)
Separate analysis	-	2.007 (0.089)	2.001 (0.092)	1.983 (0.110)	2.009 (0.089)	2.009 (0.089)	2.009 (0.089)

Table S1: FLAIR trial: Treatment effect estimates and estimated standard errors obtained when comparing the second treatment arm against the control using the proposed approaches.

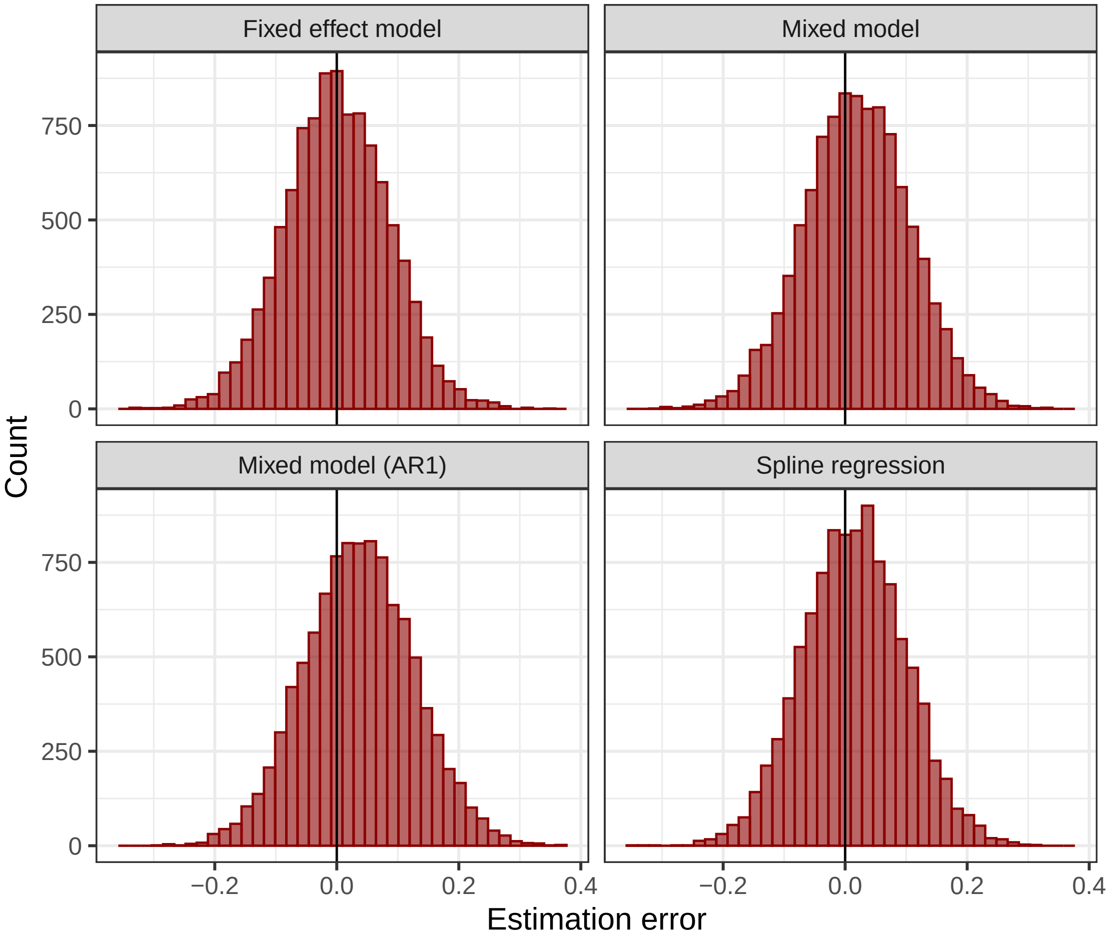

Figure S12: FLAIR trial: Histograms of the error in the treatment effect estimates in a scenario with stepwise time trend and $\lambda = 0.25$ using different models with period adjustment, estimated using 10,000 simulation runs. A reference line for the estimated error of 0 is included in each plot. The estimated means and 95% confidence intervals are: **fixed effect model:** 0.00027 [-0.00139; 0.00194]; **mixed model:** 0.01962 [0.01793; 0.02132]; **mixed model (AR1):** 0.03687 [0.03511; 0.03863]; **spline regression:** 0.01463 [0.01298; 0.01627]

References

[1] Paul H.C. Eilers and Brian D. Marx. Flexible smoothing with B-splines and penalties. *Statistical Science*, 11(2):89–121, 1996.

[2] Aris Perperoglou, Willi Sauerbrei, Michal Abrahamowicz, and Matthias Schmid. A review of spline function procedures in R. *BMC Medical Research Methodology*, 19(1):1–16, 2019.

[3] Simon N. Wood. *Generalized Additive Models: An Introduction with R*. Chapman and Hall/CRC, 2017.

[4] Talha Munir, David A Cairns, Adrian Bloor, David Allsup, Kate Cwynarski, Andrew Pettitt, Shankara Paneesha, Christopher P Fox, Toby A Eyre, Francesco Forconi, et al. Chronic lymphocytic leukemia therapy guided by measurable residual disease. *New England Journal of Medicine*, 390(4):326–337, 2024.

[5] Peter Hillmen, Alexandra Pitchford, Adrian Bloor, Angus Broom, Moya Young, Ben Kennedy, Renata Walewska, Michelle Furtado, Gavin Preston, Jeffrey R Neilson, et al. Ibrutinib and rituximab versus

fludarabine, cyclophosphamide, and rituximab for patients with previously untreated chronic lymphocytic leukaemia (flair): interim analysis of a multicentre, open-label, randomised, phase 3 trial. *The Lancet Oncology*, 24(5):535–552, 2023.

[6] Dena R Howard, Anna Hockaday, Julia M Brown, Walter M Gregory, Susan Todd, Tahla Munir, Jamie B Oughton, Claire Dimbleby, and Peter Hillmen. A platform trial in practice: adding a new experimental research arm to the ongoing confirmatory flair trial in chronic lymphocytic leukaemia. *Trials*, 22:1–13, 2021.
