# Supplementary material for: Statistical Modeling to Adjust for Time Trends in Adaptive Platform Trials Utilizing Non‐Concurrent Controls
Source: Biom J. 2025 Jun 10;67(3):e70059. doi: 10.1002/bimj.70059 (PMC12150008; doi:10.1002/bimj.70059)
Supplement: Supplementary file 1 — Supporting Information [file BIMJ-67-e70059-s002.zip › NCC.tar.gz!/NCC/inst/doc/datasim_bin.html]

How to simulate binary data


# How to simulate binary data

```
library(NCC)
```

## datasim\_bin()

The function `datasim_bin()` enables data simulation of a
platform trial with binary endpoint and an arbitrary number of treatment
arms entering at different time points.

## Assumptions

- equal sample sizes across all treatment arms, different sample size
  for the control group (resulting from allocation ratio 1:1:…:1 in each
  period)
- block randomization is used, a factor to multiply the number of
  active arms with in order to get the block size in each period can be
  specified as input argument (`period_blocks`, default=2)

## Notation

|  |  |
| --- | --- |
| **Paper** | **Software** |
| \(N\) | n\_total |
| \(K\) | num\_arms |
| \(d\) | d |
| \(n\) | n\_arm |
| \(\eta\_0\) | p0 |
| \(\lambda\) | lambda |
| \(N\_p\) | N\_peak |

## Usage

### Input

The user specifies the number of treatment arms in the trial, the
sample size per treatment arm (assumed equal) and the timing of adding
arms in terms of patients recruited to the trial so far.

- `num_arms` Number of treatment arms in the trial
- `n_arm` Sample size per arm (assumed equal)
- `d` Vector with timings of adding new arms in terms of
  number of patients recruited to the trial so far (of length
  `num_arms`)
- `period_blocks` - number to multiply the number of active
  arms with in order to get the block size per period (block size =
  `period_blocks` \(\cdot\)
  #active arms)
- `p0` - response in the control arm
- `OR`- vector with odds ratios for each treatment arm (of
  length `num_arms`)
- `lambda` - vector with strength of time trend in each arm
  (of length `num_arms+1`, as time trend in the control is also
  allowed)
- `trend` - indicates the time trend pattern (“linear”,
  “stepwise” or “inv\_u”)
- `N_peak` - point at which the inverted-u time trend
  switches direction in terms of overall sample size
- `full` - Boolean. Indicates whether the full dataset
  should be returned. Default=`FALSE`

### Output

Per default (using `full=FALSE`), the function outputs a
dataframe with simulated trial data needed for the analysis. If the
parameter `full` is set to `TRUE`, the output is a
list containing an extended version of the dataframe (also including
lambdas and underlying responses) and all input parameters.

### Examples

```
# Dataset with trial data only (default) 

head(datasim_bin(num_arms = 3, n_arm = 100, d = c(0, 100, 250),
                 p0 = 0.7, OR = rep(1.8, 3), 
                 lambda = rep(0.15, 4), trend="stepwise"))
```

```
  j response treatment period
1 1        1         1      1
2 2        1         1      1
3 3        0         0      1
4 4        1         0      1
5 5        0         1      1
6 6        1         0      1
```

```
# Full dataset

head(datasim_bin(num_arms = 3, n_arm = 100, d = c(0, 100, 250),
                 p0 = 0.7, OR = rep(1.8, 3), 
                 lambda = rep(0.15, 4), trend="stepwise", full = T)$Data)
```

```
  j response treatment period         p lambda0 lambda1 lambda2 lambda3
1 1        1         1      1 0.8076923    0.15    0.15    0.15    0.15
2 2        1         0      1 0.7000000    0.15    0.15    0.15    0.15
3 3        0         0      1 0.7000000    0.15    0.15    0.15    0.15
4 4        1         1      1 0.8076923    0.15    0.15    0.15    0.15
5 5        0         0      1 0.7000000    0.15    0.15    0.15    0.15
6 6        1         1      1 0.8076923    0.15    0.15    0.15    0.15
```
