# Supplementary material for: Statistical Modeling to Adjust for Time Trends in Adaptive Platform Trials Utilizing Non‐Concurrent Controls
Source: Biom J. 2025 Jun 10;67(3):e70059. doi: 10.1002/bimj.70059 (PMC12150008; doi:10.1002/bimj.70059)
Supplement: Supplementary file 1 — Supporting Information [file BIMJ-67-e70059-s002.zip › NCC.tar.gz!/NCC/inst/doc/how_to_run_sim_study.html]

How to run a simulation study


# How to run a simulation study

```
library(NCC)
library(ggplot2)
```

## Preparing scenarios

To perform a simulation study with the `NCC` package, we
first create a data frame with the desired scenarios that contains all
the parameters needed for data generation and analysis.

In this simple example, we consider a platform trial with 4
experimental treatment arms entering sequentially, where the null
hypothesis holds for all experimental arms. We vary the strength and
pattern of the time trend (parameters `lambda` and
`trend`) in order to investigate their impact on the type I
error, bias and MSE of the treatment effect estimates.

```
sim_scenarios <- data.frame(num_arms = 4, 
                            n_arm = 250, 
                            d1 = 250*0,
                            d2 = 250*1,
                            d3 = 250*2,
                            d4 = 250*3,
                            period_blocks = 2, 
                            mu0 = 0,
                            sigma = 1,
                            theta1 = 0,
                            theta2 = 0,
                            theta3 = 0,
                            theta4 = 0,
                            lambda0 = rep(seq(-0.15, 0.15, length.out = 9), 2),
                            lambda1 = rep(seq(-0.15, 0.15, length.out = 9), 2),
                            lambda2 = rep(seq(-0.15, 0.15, length.out = 9), 2),
                            lambda3 = rep(seq(-0.15, 0.15, length.out = 9), 2),
                            lambda4 = rep(seq(-0.15, 0.15, length.out = 9), 2),
                            trend = c(rep("linear", 9), rep("stepwise_2", 9)),
                            alpha = 0.025,
                            ncc = TRUE)

head(sim_scenarios)
```

```
  num_arms n_arm d1  d2  d3  d4 period_blocks mu0 sigma theta1 theta2 theta3
1        4   250  0 250 500 750             2   0     1      0      0      0
2        4   250  0 250 500 750             2   0     1      0      0      0
3        4   250  0 250 500 750             2   0     1      0      0      0
4        4   250  0 250 500 750             2   0     1      0      0      0
5        4   250  0 250 500 750             2   0     1      0      0      0
6        4   250  0 250 500 750             2   0     1      0      0      0
  theta4 lambda0 lambda1 lambda2 lambda3 lambda4  trend alpha  ncc
1      0 -0.1500 -0.1500 -0.1500 -0.1500 -0.1500 linear 0.025 TRUE
2      0 -0.1125 -0.1125 -0.1125 -0.1125 -0.1125 linear 0.025 TRUE
3      0 -0.0750 -0.0750 -0.0750 -0.0750 -0.0750 linear 0.025 TRUE
4      0 -0.0375 -0.0375 -0.0375 -0.0375 -0.0375 linear 0.025 TRUE
5      0  0.0000  0.0000  0.0000  0.0000  0.0000 linear 0.025 TRUE
6      0  0.0375  0.0375  0.0375  0.0375  0.0375 linear 0.025 TRUE
```

## Running simulations

We use the function `sim_study_par()` to perform a
simulation study with the created scenarios. Here we evaluate the 4th
experimental treatment arm using the regression model with period
adjustment, as well as the separate and pooled analyses. Each scenario
will be replicated 1000 times.

```
set.seed(1234)
sim_results <- sim_study_par(nsim = 1000, scenarios = sim_scenarios, arms = 4, 
                             models = c("fixmodel", "sepmodel", "poolmodel"), 
                             endpoint = "cont")
```

```
[1] "Starting the simulations. 18 scenarios will be simulated. Starting time: 2023-02-19 14:24:09"  
[1] "Scenario 1/18 done. Time: 2023-02-19 14:24:21"
[1] "Scenario 2/18 done. Time: 2023-02-19 14:24:26"  
[1] "Scenario 3/18 done. Time: 2023-02-19 14:24:32"  
[1] "Scenario 4/18 done. Time: 2023-02-19 14:24:37"
[1] "Scenario 5/18 done. Time: 2023-02-19 14:24:42"
[1] "Scenario 6/18 done. Time: 2023-02-19 14:24:47"
[1] "Scenario 7/18 done. Time: 2023-02-19 14:24:53"
[1] "Scenario 8/18 done. Time: 2023-02-19 14:24:58"
[1] "Scenario 9/18 done. Time: 2023-02-19 14:25:03"
[1] "Scenario 10/18 done. Time: 2023-02-19 14:25:08"
[1] "Scenario 11/18 done. Time: 2023-02-19 14:25:13"
[1] "Scenario 12/18 done. Time: 2023-02-19 14:25:19"
[1] "Scenario 13/18 done. Time: 2023-02-19 14:25:24"
[1] "Scenario 14/18 done. Time: 2023-02-19 14:25:30"
[1] "Scenario 15/18 done. Time: 2023-02-19 14:25:36"
[1] "Scenario 16/18 done. Time: 2023-02-19 14:25:41"
[1] "Scenario 17/18 done. Time: 2023-02-19 14:25:47"
[1] "Scenario 18/18 done. Time: 2023-02-19 14:25:52"
```

The function reports the system time after each scenario finishes in
order to track the progress of the simulations.

## Simulation results

The resulting data frame contains the considered scenarios, as well
as simulation results - probability to reject the null hypothesis, bias
and MSE of the treatment effect estimates.

```
head(sim_results)
```

```
  num_arms n_arm d1  d2  d3  d4 period_blocks mu0 sigma theta1 theta2 theta3
1        4   250  0 250 500 750             2   0     1      0      0      0
2        4   250  0 250 500 750             2   0     1      0      0      0
3        4   250  0 250 500 750             2   0     1      0      0      0
4        4   250  0 250 500 750             2   0     1      0      0      0
5        4   250  0 250 500 750             2   0     1      0      0      0
6        4   250  0 250 500 750             2   0     1      0      0      0
  theta4 lambda0 lambda1 lambda2 lambda3 lambda4  trend alpha  ncc study_arm
1      0 -0.1500 -0.1500 -0.1500 -0.1500 -0.1500 linear 0.025 TRUE         4
2      0 -0.1500 -0.1500 -0.1500 -0.1500 -0.1500 linear 0.025 TRUE         4
3      0 -0.1500 -0.1500 -0.1500 -0.1500 -0.1500 linear 0.025 TRUE         4
4      0 -0.1125 -0.1125 -0.1125 -0.1125 -0.1125 linear 0.025 TRUE         4
5      0 -0.1125 -0.1125 -0.1125 -0.1125 -0.1125 linear 0.025 TRUE         4
6      0 -0.1125 -0.1125 -0.1125 -0.1125 -0.1125 linear 0.025 TRUE         4
      model reject_h0          bias         MSE failed nsim
1  fixmodel     0.030 -0.0005506988 0.007717052      0 1000
2 poolmodel     0.007 -0.0438708399 0.008220890      0 1000
3  sepmodel     0.027  0.0005236461 0.008487647      0 1000
4  fixmodel     0.020 -0.0001912505 0.006601056      0 1000
5 poolmodel     0.008 -0.0332907139 0.006741297      0 1000
6  sepmodel     0.020 -0.0002745666 0.007570587      0 1000
```

We can now visualize the performance of the considered analysis
methods with respect to the strength and pattern of the time trend.

### Type I error

```
ggplot(sim_results, aes(x=lambda0, y=reject_h0, color=model)) +
  geom_point() +
  geom_line() +
  facet_grid(~ trend) +
  geom_hline(aes(yintercept = 0.025), linetype = "dotted") +
  labs(x="Strength of time trend", y="Type I error", color="Analysis approach") +
  theme_bw()
```

### Bias

```
ggplot(sim_results, aes(x=lambda0, y=bias, color=model)) +
  geom_point() +
  geom_line() +
  facet_grid(~ trend) +
  geom_hline(aes(yintercept = 0), linetype = "dotted") +
  labs(x="Strength of time trend", y="Bias", color="Analysis approach") +
  theme_bw()
```

### MSE

```
ggplot(sim_results, aes(x=lambda0, y=MSE, color=model)) +
  geom_point() +
  geom_line() +
  facet_grid(~ trend) +
  labs(x="Strength of time trend", y="MSE", color="Analysis approach") +
  theme_bw()
```
