# Supplementary material for: Statistical Modeling to Adjust for Time Trends in Adaptive Platform Trials Utilizing Non‐Concurrent Controls
Source: Biom J. 2025 Jun 10;67(3):e70059. doi: 10.1002/bimj.70059 (PMC12150008; doi:10.1002/bimj.70059)
Supplement: Supplementary file 1 — Supporting Information [file BIMJ-67-e70059-s002.zip › NCC.tar.gz!/NCC/inst/doc/installation.html]

How to install the NCC package


# How to install the NCC package

Below we describe how to install the `NCC` package in two
simple steps.

## 1) Installing JAGS

Prior to installing the `NCC` package, the JAGS library needs to be
installed on your computer, since the Bayesian analysis methods are
performed using the rjags R-package and
therefore require the installation of the JAGS library. You can download
the latest version of JAGS from here.

## 2) Installing the `NCC` package

### 2a) from CRAN

To install the stable version of the `NCC` package from CRAN, please run the
following code:

```
install.packages("NCC")
```

### 2b) from GitHub

To install the latest development version of the `NCC`
package from GitHub, please run the following code:

```
# install.packages("devtools") 
devtools::install_github("pavlakrotka/NCC", build_vignettes = TRUE)
```
