# Supplementary material for: Statistical Modeling to Adjust for Time Trends in Adaptive Platform Trials Utilizing Non‐Concurrent Controls
Source: Biom J. 2025 Jun 10;67(3):e70059. doi: 10.1002/bimj.70059 (PMC12150008; doi:10.1002/bimj.70059)
Supplement: Supplementary file 1 — Supporting Information [file BIMJ-67-e70059-s002.zip › NCC.tar.gz!/NCC/inst/doc/ncc_intro.html]

NCC Introduction


# NCC Introduction

## Non-concurrent controls in platform trials

Platform trials are multi-arm multi-stage clinical trials that aim at
evaluating the efficacy of several treatment arms within a single trial.
Experimental treatments are permitted to enter and exit the trial at
different times, as new treatments become available, and the total
number of newly added treatments is not pre-specified. Moreover,
treatment arms may be compared against a shared control group.

For treatments that enter the trial later, the control group is
divided into concurrent (CC) and non-concurrent controls (NCC), i.e.,
patients randomized before the given treatment arm entered the
trial.

While using NCC data can offer several benefits, such as reduced
sample size and increased statistical power, it may also introduce bias
in effect estimators if time trends are present in the trial.
